# Synonymous Genes Explore Different Evolutionary Landscapes

**DOI:** 10.1371/journal.pgen.1000256

**Published:** 2008-11-14

**Authors:** Guillaume Cambray, Didier Mazel

**Affiliations:** Unité Plasticité du Génome Bactérien, Institut Pasteur, CNRS URA 2171, Paris, France; Fred Hutchinson Cancer Research Center, United States of America

## Abstract

The evolutionary potential of a gene is constrained not only by the amino acid sequence of its product, but by its DNA sequence as well. The topology of the genetic code is such that half of the amino acids exhibit synonymous codons that can reach different subsets of amino acids from each other through single mutation. Thus, synonymous DNA sequences should access different regions of the protein sequence space through a limited number of mutations, and this may deeply influence the evolution of natural proteins. Here, we demonstrate that this feature can be of value for manipulating protein evolvability. We designed an algorithm that, starting from an input gene, constructs a synonymous sequence that systematically includes the codons with the most different evolutionary perspectives; i.e., codons that maximize accessibility to amino acids previously unreachable from the template by point mutation. A synonymous version of a bacterial antibiotic resistance gene was computed and synthesized. When concurrently submitted to identical directed evolution protocols, both the wild type and the recoded sequence led to the isolation of specific, advantageous phenotypic variants. Simulations based on a mutation isolated only from the synthetic gene libraries were conducted to assess the impact of sub-functional selective constraints, such as codon usage, on natural adaptation. Our data demonstrate that rational design of synonymous synthetic genes stands as an affordable improvement to any directed evolution protocol. We show that using two synonymous DNA sequences improves the overall yield of the procedure by increasing the diversity of mutants generated. These results provide conclusive evidence that synonymous coding sequences do experience different areas of the corresponding protein adaptive landscape, and that a sequence's codon usage effectively constrains the evolution of the encoded protein.

## Author Summary

Evolutionary processes largely rely on the production of diversity. Genetic robustness, by allowing the accumulation of neutral diversity within a population, has been associated with increase in evolutionary potential (evolvability). In this work, we propose to use a well-known source of robustness, the redundancy of the genetic code, to alter the evolvability of any protein. The topology of the code allows synonymous codons to sample different mutational neighborhoods. Using this property, we developed an algorithm to design synonymous sequences with maximally divergent evolutionary potentials relative to the input sequences. At the population level, each of these sequences expands the scope of the evolutionary landscape that can be explored by the encoded protein, and ultimately increase the odds of uncovering adaptive mutants. We applied this principle to evolve new antibiotic resistance phenotype variants. Fundamentally, our results provide an example of how neutral diversity may favor evolvability. Moreover, in light of the rapid development in nucleic acid synthesis, the use of rationally designed synonymous genes offers a profitable enhancement to any directed evolution procedure.

## Introduction

Natural proteins typically exhibit a range of weak promiscuous activities that require few mutations to be improved [1]. Such changes, however, may not be directly accessible but instead necessitate the stepwise accumulation of several mutations. Darwinian evolution generally relies on the existence of an adaptive pathway in which all intermediate steps provide a gradual improvement of protein function. Routes that comprise some deleterious or neutral intermediates can also be followed, but such mutations have to be fortuitously fixed by genetic drift. Importantly, evolutionary pathways passing through fitness valleys or neutral ridges may largely prevail among those available to evolution [2]. Apart from occasional situations of relaxed selection, best exemplified by gene duplication events, protein adaptation is thus a heavily constrained process.

Biological engineering often produces valuable approaches to bypass such natural constraints and provides insight into fundamental questions from a fresh point of view [3]. By implementing natural selection processes, directed evolution strategies were successfully developed to derive desired features from existing protein templates. Incidentally, they also greatly contributed to our improved understanding of protein evolution [4]. Following this line, the purpose of this work is twofold: to describe a new approach to artificially expand the explorable adaptive landscape of a given protein sequence and to exploit the data generated to highlight the role of synonymous codons in sequence evolution.

Directed evolution proceeds by incremental isolation of advantageous mutations through successive rounds of random variation and subsequent selection. The efficiency of such experiments relies on both a proper exploration of sequence space and the adequate design of selective pressure. While selective screening strategies are usually specific to the polypeptide being evolved, the generation of diversity is a general prerequisite to successful evolution. A comprehensive exploration of the sequence space around template proteins involves sampling the 19 alternative amino acids (aa) at each position of the polypeptides [5]. To deal with the resulting combinatorics, a common approach is to restrict the protein space to be searched [6]. This can be achieved by using pre-existing diversity as a substrate for PCR-mediated recombination [7], which permits wide jumps in the protein space and limits exploration to *a priori* interesting areas. Alternatively, when the structure/function relationships of a protein are well described, rational predictions can drive targeted mutagenesis of specific residues. *De novo* design of whole functions has even been reported [8]. However, most advantageous mutations are unpredictable and can only be unveiled by blind modification of large sequence segments [9]. Current studies mainly focus on semi-rational approaches in which bioinformatic inferences are used to optimize the generation of recombinant or mutant libraries, or to specify regions to mutate randomly [10],[11].

Despite these advances, our ability to explore adaptive landscapes *per se* remains limited, and the generation of variants requiring several mutational changes is still particularly challenging. As mentioned above, globally adaptive pathways that comprise proximally deleterious or neutral intermediates are unlikely to be explored in experimental evolution. Only few adaptive mutants are usually selected at each round of directed evolution, leaving little room for extensive genetic drift to occur. Variants containing deleterious mutations are irrevocably lost for subsequent rounds of selection, while neutral changes must be linked to a selected mutation to persist in the libraries over time. To circumvent these limitations, mutation rates can be increased *in vitro* to allow the simultaneous appearance of double or triple mutants, while avoiding the cost or absence of benefice of intermediates [12]. However, 30 to 40% of the non-synonymous mutations are usually expected to disrupt a protein's structure, thereby perturbing its activity [13]. Even if high mutation rates limit the redundancy of the generated libraries, optimal rates should not exceed few nucleotides per sequence [14]. Hence a gene-wide increase in mutation rate comes at a high cost, for much larger libraries are required to produce the beneficial combination of mutations without any other strongly deleterious mutations associated. Assuming 1/3 of lethal aa substitutions, a standard 1000 bp coding sequence and a mean mutation rate of 4 nucleotide substitutions, we estimated that a minimal 300 fold increase in library size is necessary to generate on average one given functional double mutant compared to a single one. This figure increases to more than 100,000 fold when considering a phenotype caused by three nucleotide mutations rather than one (see supporting Text S1).

While it is difficult to optimize artificial selection procedures to allow the usage of adaptive pathways with fitness valleys, much can be done to facilitate the passage through neutral intermediates. Particularly, increased protein robustness has been reported to promote evolvability [15], because it allows the protein to tolerate a wider range of mutations and thus permits more pathways to be explored. Recent experiments also championed extended periods of neutral drift as a valuable method to accumulate viable variations prior to directed evolution [4]. In any case, the amount of time necessary for drift stands as the major factor limiting the exploration of neutral sequence space. Here, we describe a strategy that builds on a universal aspect of mutational tolerance, the redundancy of the genetic code, to artificially introduce neutral and evolvable variability into coding sequences. Our approach widens the protein evolutionary landscape that can be explored, while avoiding both high mutation rates and the time required for extensive genetic drift to occur. We designed a synonymous version of the gene *aac(6′)-Ib* [16] that optimizes differential exploration of the genetic space relative to its wild type counterpart. The active site of the encoded aminoglycoside 6′-N acetyltransferase is known to display substantial plasticity, enabling the enzyme to adapt to new substrates with relatively few mutations [17].This synthetic gene provides a functional basis from which to access otherwise remote aa substitutions (i.e. requiring at least 2 mutations per codon). We experimentally demonstrate that each synonymous sequence effectively experiences different areas of the enzyme's adaptive landscape, ultimately leading to the isolation of radically different mutants from each other. Biotechnological and evolutionary implications of these results are discussed.

## Results

### The Evolutionary Landscape Painter Principle

**Figure 1:**
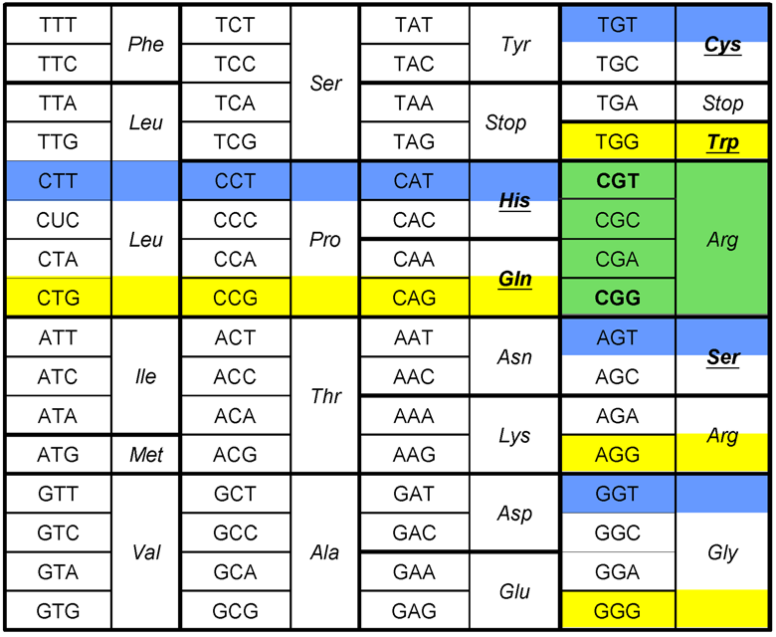
Standard genetic code and codon evolutionary landscapes. Every codon can access nine other codons by single mutation, which corresponds to only 5.8 proximal aa on average due to the code redundancy. These evolutionary perspectives are highlighted in yellow and blue and for the arginine codons CGG and CGT, respectively (green indicates codons accessible from both). Amino acids reachable by only one of the two codons are bold and underlined.

A given codon can only access nine proximal codons through single mutation, but only 65% of these codon changes effectively result in aa substitution, i.e. codons have only 5.8 proximal aa on average (see the genetic code, Figure 1). However, some synonymous codons display different sets of proximal aa. For example, let us consider the arginine codons CGG and CGT: the former can access the aa {Leu; Pro; Gly; Gln; Trp} through single point mutation, while the latter can reach {Leu; Pro; Gly; His; Ser; Cys}. So, a synonymous change of an initial CGG to a CGT in a coding sequence gives direct access to {His; Ser; Cys} while losing access to {Gln; Trp} (Figure 1). In terms of adaptive pathways, this suggests that if a transition from arginine to cysteine requires the passage through a neutral intermediate when the initial sequence is CGG, it is direct if switched to CGT.

This property relies on the interplay between the code redundancy and topology. Its evolutionary significance can be conceptualized as ‘traveling without moving’: the protein sequence does not change, retaining function and fitness, while the immediate protein adaptive landscape differs, which provide the protein with new opportunities to evolve. Based on this principle, synonymous versions of any coding sequence can be designed to decrease the number of mutations necessary to obtain given aa substitutions, thereby avoiding library size enlargements consecutive to increases in mutation rates and time consumption linked with random drift along neutral pathways. In addition, this strategy obviously permits to efficiently introduce several mutations in the same codon, a property that is statistically difficult to achieve with usual directed evolution procedures.

As there is generally no *a priori* evidence to favor specific aa substitutions relative to others, we will now describe how such synonymous sequence manipulations can conveniently be used to broaden any protein's evolutionary potential.

Based on a closely related analysis of the code redundancy, a “volatility” index was previously defined to allegedly detect selection [18]–[22]. This analysis was focused on past evolution of single sequences, while we need a prospective measure of the differences between two synonymous codons' evolutionary perspectives. We thus define the Relative Evolutionary Potential of codon XXX relative to its synonymous counterpart YYY (REP_XXX/YYY_) as the number of different aa reachable from XXX but not from YYY, through single mutation. In the example above, REP_CGG/CGT_ equals 2 and REP_CGT/CGG_ is 3 (Figure 1). Given the standard genetic code, half the twenty aa displays groups of synonymous codons with non null REP (see Figure S1). Considering ongoing improvements in gene synthesis, it is conceivable to extensively manipulate the evolutionary perspectives of any gene through systematic introduction of specifically chosen synonymous codons. To do so we implemented an algorithm, the Evolutionary Landscape Painter (ELP), to search synonymous DNA sequence space for alternative sequences showing a maximal number of proximal aa that are different from the initial sequence (*i.e.* a maximization of every codon's REP with respect to the input sequence). While encoding the same protein, an output sequence maximizes accessibility to aa that were not directly reachable from its template. When subjected to any directed evolution protocol, wild-type and ELP-designed synonymous sequences should directly access different areas of the protein space, thereby increasing the odds to isolate mutations of interest.

### Experimental Validation

To test if manipulating the synonymous codons within a sequence could indeed help artificial selection to follow new adaptive pathways, we chose the versatile bacterial aminoglycoside resistance gene *aac(6′)-Ib*. As it specifies antibiotic resistance phenotypes, this model provides a straightforward screening strategy. Moreover, this gene normally confers clinical levels of resistance to 3 aminoglycosides (amikacin, tobramycin, and netilmicin) but *aac(6′)-Ib* mutants coding for altered aminoglycoside resistance spectra have been naturally isolated in clinical bacteria (see for example [16]). We identified 129 different natural homologs (≥80% aa identity) of this gene in GenBank. These data can be used to compare the outcomes of our artificial selection method and natural selection in the wild.

**Figure 2:**
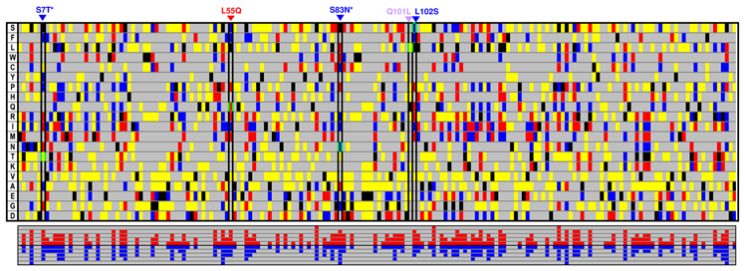
Evolutionary landscape comparison of the aac_WT_ and aac_ELP_ synonymous genes. aac_ELP_ has been designed by the ELP software to maximize evolutionary perspective divergence from aac_WT_ when subjected to single mutation. The 184 successive positions along AAC(6′) are represented as columns. Each row stands for an aa, as indicated by the one-letter code in first column. Intersection between aa and position are coloured according to the following code: *black*, aa encoded at that position; *blue*, aa specifically accessible by *aac_WT_*; *red*, aa specifically accessible by *aac_ELP_*; *yellow*, aa directly accessible from both *aac* genes; *grey*, aa inaccessible from either *aac* genes through single mutation. Blue and red patterns, denoting different evolutionary perspectives, are widely and evenly distributed along the sequence. The histogram on the bottom summarizes the number of aa directly accessible only by *aac_WT_* (blue squares) or *aac_ELP_* (red squares) for each position. Mutations isolated in this study are indicated above the diagram and corresponding positions are black lined. Mutations' outcomes are highlighted with bright green borders. Asterisks denote mutations isolated together.

We annealed long oligonucleotides (Table S1) to physically assemble *aac_ELP_*, a ELP-designed synthetic sequence of *aac(6′)-Ib*. While excluding the codons rarely used in *E. coli* (see material and methods), this synonymous gene harbored 119 (out of 184) codons different from the wild type sequence *aac_WT_*, which resulted in only 61% identity between both sequences at DNA level (Figure S2). As mentioned above, each codon can, on average, access 5.8 aa substitutions. Each codon in the *aac_ELP_* sequence can reach 1.6 proximal aa that were not accessible from the wild type, and this number raises up to 2.5 aa if we only consider codons that have been changed. Conversely, a codon from *aac_WT_* can reach 1.5 proximal aa that are not accessible from the synthetic sequence (2.4 aa when considering only those changed). Globally, parallel evolution of these two synonymous sequence would thus significantly expand the AAC(6′)-Ib protein space exploration. A graphical overview of the evolutionary perspectives differences between the two *aac* sequences is presented in Figure 2.

**Figure 3:**
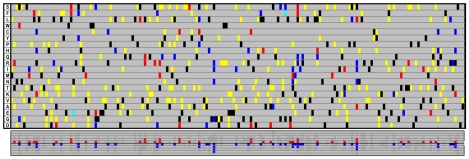
Comparison of the amino acid substitutions identified in alleles sampled from the mutant libraries before selection. This diagram is reminiscent of Figure 2. The 184 successive positions along the AAC(6′)-Ib protein are represented as columns. Each row stands for an aa, as indicated by the one-letter code in first column. Black cells show the WT sequence, other coloured cells highlight the substitutions identified by sequencing 297 aac_WT_ and 267 aac_ELP_ alleles, independent and randomly chosen before any selection. The color code is the following: *blue*, observed substitution only directly accessible from *aac_WT_*; *red*, observed substitution only directly accessible from *aac_ELP_*; *yellow*, observed substitution directly accessible from both genes; *green*, observed substitution resulting from a double mutation in the same codon. Overall, we identified 524 substitutions out of which 110 were only accessible from one of the version of the gene and not the other. The mutations specific of each gene versions were only observed from the cognate template. The histogram on the bottom summarizes the number of aa substitution specific to each version that were sampled at each position (*aac_WT_*, blue squares; *aac_ELP_*, red squares). The shaded area recalls the theoretical pattern as shown in Figure 4.

*aac_WT_* and *aac_ELP_* were concurrently subjected to an identical directed evolution protocol. Briefly, mutations were introduced by PCR using error-prone polymerase. For each version of the gene, we generated four independent *E.coli* libraries, with mutation rates ranging from 0.5 to 5.2 mutations/kb (Table S2). We estimated that each library contained more than 10^6^ clones. For each gene version, we randomly picked more than 250 clones from these libraries and sequenced their *aac* alleles. Among the 524 deduced aa substitutions identified from both gene templates, 110 (ca. 20%) were only directly accessible from one version but not from the other (see Figure 3). These data demonstrate that the two synonymous sequences readily explore different areas of the protein space.

Libraries were then screened on agar plates containing a gradient of the tested antibiotics for isolated colonies growing in the zone of concentrations preventing growth of wild type phenotype. We applied this screen for increased resistance phenotypes on the following aminoglycosides antibiotics: tobramycin, amikacin, gentamicin, isepamicin, neomycin and kanamycin. No mutants were isolated from tobramycin, neomycin or kanamycin based screens, suggesting local evolutionary dead ends with these antibiotics. This correlates with reports on the closely related gene *aac(6′)-Iaa* [23]. On the other antibiotics, three mutations caught our attention as they involved codons that were different in the two synonymous sequences (Table 1). The description of a natural *aac(6′)-Ib* double mutant associated with a modest increase in fluoroquinolone resistance [24], a different class of antibiotic, prompted us to further screen our libraries on such compounds. However, we did not select any mutant showing such a phenotype.

**Table 1:** Mutations isolated from the two synonymous versions of the gene *aac(6′)-Ib*.

		Mutations isolated^1^		
		*MIC* 3		
		N = number of isolates^2^		
Antibiotic^4^		Amikacin	Gentamycin	Isepamicin
WT MIC^3^		5 µg/mL	0.25 µg/mL	0.4 µg/mL
**Gene version**	***aac_WT_***	**Q101L**	**L102S** 5	**S7T+S83N**
		CAG→CTG	TTA→TCA	TCC→ACC+AGC→AAC
		*10 µg/mL*	*1.5 µg/mL*	*1.2 µg/mL*
		N = 3	N = 35	N = 1
	***aac_ELP_***	**Q101L**	ND^6^	**L55Q**
		CAA→CTA		CTG→CAG
		*10 µg/mL*		*1.6 µg/mL*
		N = 1		N = 25

1 Gene mutant libraries were independently screened for enhanced resistance phenotype on six different aminoglycoside antibiotics. Each aa substitution is directed by a single nucleotide difference. Three out of five substitutions (L102S, S83N and L55Q) are directly accessible by only one of the gene version, and were not isolated from any library of the alternative gene.

2 The number of independent colonies carrying the mutation isolates from the corresponding libraries.

3 The global improvement of resistance phenotype is measured by the Minimum Inhibitory Concentration (MIC). To avoid any influence of the sequence background on observed phenotypes, mutations isolated from *aac_ELP_* were reconstructed by site directed mutagenesis and tested in *aac_WT_*.

4 No mutant was selected on the aminoglycosides kanamycin, neomycin and tobramycin, nor on the fluoroquinolones ciprofloxacin, levofloxacin and ofloxacin.

5 The L102S mutation corresponds to the L83S mutation described by Lambert and col. The numbering difference is due to an error in the start codon choice in their original characterization [16].

6 ND: Not Detected.

As expected, the differences observed in the mutations selected from the two sequences reflected the number of nucleotide changes required to produce the selected aa change. Amikacin based screens led to isolation of a Q101L mutant from both *aac_WT_* and *aac_ELP_* derived libraries, which is logical as leucine at codon 101 of *aac_WT_* (CAG) or *aac_ELP_* (CAA) is equally accessible by mutation to CTG and CTA. Conversely, improvement of gentamicin resistance was only observed from the wild-type sequence: 35 independent colonies from the different WT libraries were found to carry a L102S mutation, in several cases associated with different other mutations that further proved to be neutral. L102S requires a single TTA to TCA transition from *aac_WT_*, while at least two mutations are required to encode a leucine from the CTG codon present in *aac_ELP_*. Similarly, screening on isepamicin, allowed for the selection of a single S7T+L12F+S83N mutant from *aac_WT_*. Further analysis of this latter mutant showed that only the S7T and S83N mutations participate in the enhanced resistance. L12F alone is associated with a slightly decreased resistance phenotype and must have been selected via hitchhiking. As previously observed for L102S, S83N is only directly accessible from *aac_WT_*, hence no such mutants were produced from the synthetic genes library. Finally, selection on isepamicin, also led to the isolation of a L55Q mutation that was recurrently isolated from the synthetic gene mutant library but not from wild-type (25 independent colonies, again linked with different neutral mutations), demonstrating that the manipulation of synonymous codons within the sequence could help find new beneficial mutations. The L55Q mutation resulted from a single CTG to CAG transversion in *aac_ELP_*, while a double or triple mutation would have been required to reach Gln (CAA or CAG) from *aac_WT_*'s Leu TTA codon.

### Simulation of Adaptive Landscape Exploration

**Figure 4:**
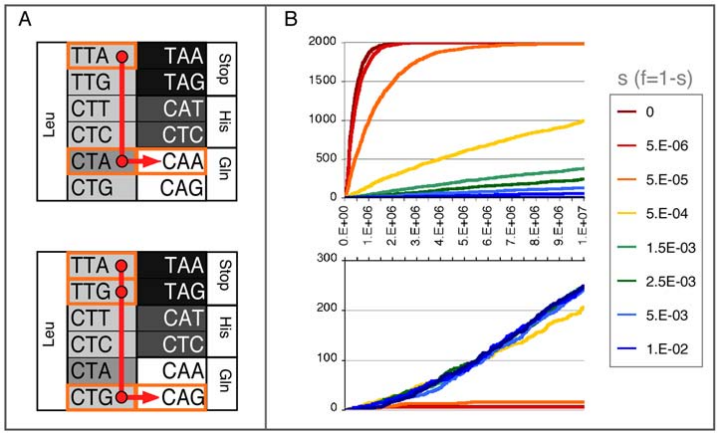
Impact of the low-usage leucine codon CTA on L55Q evolutionary pathways. The L55Q mutation has only been isolated from *aac_ELP_* libraries and has never been described in natural isolates. In *aac_WT_*, L55 is encoded by TTA. Panel A: two main pathways can be followed to access Gln under selection, one with a single CTA intermediate, which might be associated with a lower fitness (*top*, selective pathway) and the other with two neutral intermediates (*bottom*, neutral pathway). Every genotype presented in these schematic landscapes has been constructed and assessed for growth on isepamicin. The colouring scheme, with darker colours associated with lower fitness, derives from these results except for the weakly used codon CTA for which the putative fitness effect was not measurable. Panel B: Monte-Carlo simulations of the evolution of TTA alleles populations were carried out, assuming various fitness f of CTA. *s* denotes the selective coefficient associated with the CTA codon. The cumulative number of simulations where CAA (*top*, selective pathway) or CAG (*bottom*, neutral pathway) reached fixation is plotted over time in generations (2,000 repetitions). Even weak selective coefficient against CTA (e.g. 5 10^−4^) greatly affects the use of the selective pathway. Although longer, the neutral pathway is preferred over the selective one from selective coefficients as low as 2.5 10^−3^.

We observed a striking correlation between aa accessibility from *aac(6′)-Ib* wild-type sequence and occurrences in databases. As stated above, 129 different homologs were identified in GenBank (see supporting Dataset S1). Among them, 43 display the L102S mutation, which is associated with a switch in AAC(6′)-Ib resistance spectrum [16]. The other substitutions directly accessible from *aac_WT_*, Q101L and S83N, are represented twice and once respectively. On the contrary the L55Q mutation, which is only accessible from *aac_ELP_*, has never been described to our knowledge. This suggests that pathways leading to this specific aa substitution are poorly explored through natural evolution. Potential L55Q evolutionary pathways are TTA→TT**G**→**C**T**G**→**CAG** (Figure 4A, bottom), which involve two putatively neutral intermediates and TTA→**C**TA→**CA**A (Figure 4A, top) with only one putatively neutral intermediate. Although the latter route does not primarily seem beyond evolutionary scope, one must note that the CTA intermediate codon is rarely used in numerous host bacterial genomes (e.g. only 4% of the leucine codons in *E.coli*). Given that a growing body of evidence points to the existence of weak fitness differences related to codon usage (see discussion), this observation may be biologically relevant. Supporting this idea, we found that one *aac(6′)-Ib* homolog displays the synonymous Leu TTG in position 55 (sequences accession AY444814, DQ357197, EF514364; 85% identities at the nucleotide level) and two closely related homologs display Leu CTG (AM283490, AM283489, AJ640197 and EF514368; 82 and 85% identities, respectively). Both of these codons are part of the former three-stepped pathway, while no sequence exhibits Leu CTA, the only intermediate on the shorter route to glutamine at this position.

To investigate how subtle sub-functional constraints such as codon usage would affect the evolutionary pathway followed, we conducted simple Monte-Carlo simulations [25]. We qualitatively mapped the adaptive landscape relevant to the L55Q substitution and implemented it to follow the evolution of homogeneous Leu TTA allele populations until the fixation of Gln, assuming various values for the impact of Leu CTA allele on fitness (Figure 4). Results show that the number of generations necessary for Gln CAA fixation rises sharply with only slight decreases in Leu CTA associated fitness (Figure 4B, top). Fixation of Gln CAG does not require passing through CTA, but it does requires enough time to drift through two neutral intermediates according to the pathway TTA→TT**G**→**C**T**G**→**CAG** (Figure 4A, bottom). For Leu CTA fitness decreases larger than ca. 0.1%, this neutral pathway appears over time as a more probable outcome (Figure 4B, bottom). Hence, a faintly rugged adaptive landscape can favor longer/neutral versus shorter/weakly selective pathways over evolutionary time.

The difficulty, even for natural selection in the wild, in using adaptive pathways comprising some neutral or slightly deleterious mutations suggests that many natural proteins might not be optimal. The approach we have described is thus likely to allow unexplored adaptive pathways to be tested.

## Discussion

It has long been established that the DNA composition directly affects the amino acid composition of proteins. The bulk correlation between GC content and the aa composition has been early evidenced [26], while more subtle effects, such as positioning on the leading or lagging strand was more recently detected [27]. Our results demonstrate that in addition to these different effects, the coding sequence composition directly influences the future evolution of proteins. Moreover, we show that one can easily manipulate this principle to widen the short termed evolutionary perspectives of any given protein.

A single round of directed evolution of the two synonymous sequences *aac_WT_* and *aac_ELP_* led to the isolation of five mutations modifying the resistance spectrum of the encoded AAC(6′)-Ib enzyme. Among these mutations, three were accessible from only one of two synonymous sequences through single mutation, and were indeed only isolated from the corresponding mutant libraries. These results, together with the observed substitution pattern sketched in Figure 3, clearly show that parallel directed evolution of specifically designed synonymous sequences permits a wider exploration of the local protein landscape. In the framework of a serial directed evolution experiment, once a beneficial mutation has been identified in a given sequence, it can easily be introduced in other synonymous sequences by site-directed mutagenesis to proceed to the next round of evolution.

We emphasize that an ELP-designed sequence *per se* does not improve the encoded protein evolvability. The strategy we propose is rather a hypothesis-free approach to expand the evolutionary perspectives of existing proteins, so that parallel directed evolution of wild type plus synthetic sequences actually increases the overall odd to identify advantageous mutations. Indeed, what does matter from a biotechnological point of view is not the evolvability of a given DNA coding sequence, but the ability to extensively explore the corresponding protein sequence space. Conceptually, this dissociates the polypeptidic product of interest from the actual nucleic acid sequence from which it originates.

As no aa displays more than four codons with different REP, four synonymous sequences are sufficient to explore all the possibilities allowed by this principle, assuming independence between positions along the sequence (no epistasis). Obviously, a huge number of synonymous sequences would be needed to tackle the combinatorial association of codons between positions, but parallel evolution of four sequences seems a tractable alternative. The ELP software allows drawing of up to three alternative sequences with evolutionary perspectives as different as possible from each other and from the initial sequence at each codon. The use of such a set of sequences significantly reduces the number of mutations necessary for extensive landscape exploration (Figure S3), and consequently decreases the required library size by several orders of magnitude (see supporting Text S1). Effective mutational spectra vary markedly according to protocols, and in some cases it can be controlled for. This can be used to discriminate between otherwise equivalent alternative codons. An improved version of the ELP program will be developed to take such parameters into account.

Evolvability and robustness are tightly linked, with exploration of neutral networks potentially fuelling adaptive evolution [28]. Hence, methods that improve spreading along a protein neutral space result in promoting its evolvability [4],[15]. In this study, we chose the most open and straightforward approach to design the neutral alternative sequence. The REP calculation does not rely on any particular assumption about the chemistry of the protein: every aa accessible by single mutation is counted as one unit (Hamming metric) and only synonymous codons were considered as potential alternatives. However, any idiosyncratic knowledge of a protein structure/function relationships can be incorporated in the calculation, by applying different metrics to specific residues or regions of the protein. When available, *in silico* predictions might also be used to include non-synonymous, but nonetheless neutral mutations as potential alternatives in the REP calculations. This latter strategy, however, is risky because a single mispredicted substitution can flaw the enzyme activity and its derived library. Apart from improving directed evolution of proteins, synonymous codon replacement might alternatively be used to prevent the appearance of previously identified deleterious mutations, thereby favoring protein robustness in specific biotechnological applications.

Formally, the principle presented here plays with the exploration of synonymous sequence space. It is usually assumed that this exploration depends upon mutation rate and chance (neutral drift), in which case the use of synthetic sequences saves the time necessary for these processes to occur. However, some weak sub-functional forces may also structure synonymous space and constrain evolutionary pathways in many species [29]–[31]. The ELP strategy permits one to circumvent such constraints. As a case study, we focused on the L55Q substitution which was only isolated from the synthetic sequence *aac_ELP_* and was not directly accessible from the wild type sequence *aac_WT_*. Strikingly, it is the only mutation identified in this study which is not represented in the 129 different *aac6′-Ib* homologous sequences deposited in the NCBI database. We identified two possible evolutionary pathways for that substitution. The longer one comprises two synonymous intermediates and can be explored by extensive drift over long time scale. The shorter one comprises only one synonymous intermediate, but the corresponding codon is very weakly used throughout many of the gene's host genomes.

Over the last 20 years, experimental studies reported various phenotypic effects associated with modification of sequences codon usage: alteration of mRNA structure [32],[33], modification of translation efficiency [34],[35], and protein aggregation due to alteration of folding route and final tertiary structure [36]–[38]. Although a recent report has linked a phenotypic effect to the presence of two weakly used codons combined with a non-synonymous SNP [39], to the best of our knowledge no significant impact of a single rare codon has ever been described, most likely because it should involve a decrease in protein synthesis too weak to be measured accurately.

As we did not manage to measure any effect of the weakly used L55 CTA codon alone, we performed simulations of the L55Q adaptive landscape exploration in which we assumed various fitness values associated with this codon. Not surprisingly, drift toward Gln CAA requires a substantial amount of time, even when the CTA intermediate is considered neutral. Our results show that fitness decreases that are too faint to be detected *in vivo* can strongly affect the passage through weakly used codons. Eventually, longer and neutral pathways can rise as more probable outcomes over time. The hypothetic non-appearance of the adaptive L55Q substitution in nature would then be consistent with the relatively recent introduction of the antibiotic selective pressure. Nevertheless, we identified distant homologs in GenBank that display Leu TTG or CTG in position 55. These sequences can stand as natural intermediates along the longer pathway toward Gln CAG (see Figure 4A bottom).

If the adaptive landscape of proteins is indeed subtly structured by the codon preferences of the host genome, these constraints should be altered by higher-order evolutionary events such as horizontal gene transfer (HGT). At least in bacteria, HGT is a major factor of genome evolution [40], while phylogenetically distant species usually display markedly different codon usages. The introgression of a gene may compel its codon usage to conform to the new host [41], thus granting access to new adaptive pathways and offering chances to provide different mutants. Another intriguing issue, that should soon become feasible considering the current intensive efforts in synthetic biology [42], would be to recode the full set of genes encoding a metabolic pathway, and even a whole bacterial genome [43]. By relieving several constraints at the same time, this could unlock access to potentially adaptive solutions and give access to the study of evolutionary phenomena from an upper scale.

The codon composition of a coding sequence is the outcome of its history, whether it is selective or contingent. It has been suggested that natural selection might actively bias the codon usage of some proteins to modulate their robustness to mutation or mistranslation [18],[44]. Although this later possibility remains unclear, we demonstrated experimentally that an astute reorganization of the synonymous codons can be performed artificially to modify the evolvability of the encoded protein. This strategy allows wider exploration of the protein space while limiting both library sizes and amount of time usually required for genetic drift. Hence, it provides an inexpensive and powerful tool to enhance the efficiency of any directed evolution protocol.

## Methods

### Evolutionary Landscape Painter (ELP) Algorithm

ELP is a Perl implemented script accessible via a CGI web interface. It explores the synonymous DNA sequence space of any coding sequence for alternative sequences with highest evolutionary perspectives differences. Users are invited to enter an initial coding sequence and prompted to choose the maximum number of weakly used codons allowed to be introduced in the output sequence as well as its overall G+C content. Weakly used codons in *Escherichia coli* are defined as CTA, AGG, ATA and TAG (derived from the codon usage database, http://www.kazusa.or.jp/codon/). A synonymous sequence is generated at random by choosing a codon among the highest REP codons available at each position. This sequence is arbitrarily evolved in the maximum REP codon space until fitting user requirements. If the only available highest REP codon at a given position is a weakly used codon, the program may change it to an immediately lower REP codon. To further exploit the potentiality of the approach (see Discussion), the program can genetrate two additional alternative sequences so that each sequence maximizes its REP relative to each other. Note that only arginine is encoded by four codon groups with different REP (Figure S1), so the use of a fourth alternative sequence only improves exploration at arginine residues. The ELP program is accessible on line at the following address http://www.pasteur.fr/recherche/unites/pgb/ELP. We used ELP to design one alternative version (*aac_ELP_*) of the *aac(6′)-Ib* gene (*aac_WT_*), requiring a 50.1 G+C content and no weakly used codon. The sequences of both genes are shown in Figure S2.

### Synthetic Gene and Genetic System Construction

The synonymous sequence outputted by ELP (*aac_ELP_*) was physically constructed. A set of 17 overlapping 5′ phosphorylated oligonucleotides has been defined to cover the whole sequence of *aac_ELP_* without any gaps (see Table S1). Oligonucleotides were equimolarly mixed together in water. The solution was warmed up to 80°C and slowly cooled to room temperature. The resulting annealed fragments were ligated using T4 DNA ligase, resulting in *aac_ELP_* assemblage. *aac_WT_* was PCR amplified from pAT672. EcoRI and BamHI cleavage site were introduced 5′ and 3′ of the genes, respectively. Plasmid pAM238, a pSC101 derivative carrying a spectinomycin resistance marker [45], was used as low-copy number vector. Genes were cloned downstream a Plac promoter using EcoRI and BamHI. The RBS was modified to include the EcoRI cloning site.

### Parallel Directed Evolution of aac_WT_ and aac_ELP_

#### Error-Prone PCR

Both versions of the gene were separately amplified using the mutazyme II kit (Stratagene). PCR products were purified (PCR purification kit, Qiagen) and EcoRI/BamHI cloned into the pAM238. Resulting constructs were transformed into *Escherichia coli* TOP10 electrocompetent cells (Invitrogen) and selected on the vector marker, spectinomycin (50 µg/ml) and not for. Four PCRs were achieved for each gene with increasing amounts of template, resulting in decreasing mutation rate. This led to the construction of four independent libraries for each gene, covering different mutation rates (Table S2). As a preliminary evaluation of the mutation rate, 96 randomly chosen isolates were assessed for growth under low concentrations of tobramycin to evaluate the functionality of AAC mutants (data not shown). *aac* alleles of the same samples were then sequenced to evaluate mutation rate after amplification following the Templiphi procedure (Amersham Biosciences). Sequencing reactions were carried out using BigDye terminator v3.1 on template DNA and analyzed with a 3100 Genetic Analyzer sequencer (ABI). Complete sequences from this dataset were also used to compare exploration of evolutionary landscape before selection (Figure 3).

#### Selection

Aliquots of each library were grown overnight without imposing selective pressure on the aac genes. Dilutions (1/1000) were then homogeneously spread on culture plates. Plates were previously structured by letting aminoglycosides diffuse overnight from a disk, resulting in a smooth antibiotic gradient. This allowed clear isolation of colonies showing enhanced resistance phenotype. Six aminoglycoside antibiotics where separately used to screen the different mutant libraries: tobramycin (Tob), amikacin (Amk), Gentamycin (Gm), isepamycin (Isp), neomycin (Neo) and kanamycin (Kan). Three fluoroquinolone antibiotics, ciprofloxacin, levomycin and ofloxacin, were used to further screen the libraries.

#### Mutant Analysis

Alleles borne by the isolated and purified colonies were PCR amplified using error-safe polymerase (Pfu, Promega). Amplicons were systematically cloned back in a naïve pAM238 vector and transformed into *E.coli* DH5α chemically competent cells. Enhanced resistance phenotypes on the antibiotic of isolation were then assessed to discriminate false positives. Candidates were then sequenced and compared to the cognate original sequences. MICs were measured by e-test when available (Amk and Gm, AB Biodisk) or by standard liquid culture protocol with smoothly increasing antibiotic concentration. For Isp, the MIC is defined as the first concentration where growth is not detected after overnight culture in liquid ML broth containing Isp in concentration ranging from 0.2 to 2 µg/ml, with gradual increment of 0.1 µg/ml.

### Accession Numbers

The wild type aac_ELP_ and its L55Q derivative sequences have been deposited at GenBank under accession numbers FJ269241 and FJ269242, respectively.

### Database Comparison

A BlastP against the AAC(6′)-Ib protein sequence was performed on the NCBI's nr database. Only HSPs (High-scoring Segment Pair) displaying ≥80% identities were considered. Corresponding nucleotide sequences were fetched, sorted and aligned using a dedicated BioPerl script. 129 different naturally occurring homologs, corresponding to 297 different accessions, were identified (see supporting Dataset S1).

### Monte-Carlo Simulation

A simple C implemented Monte-Carlo model was used to stochastically evolve sub-population of alleles. At each generation, sub-populations are multiplied according to the relative fitness of the considered allele. Then mutation was introduced, with frequencies of double or triple mutations simply derived from the frequency of simple mutation. Resulting sub-populations were then sampled according to a Poisson law (or Binomial for low frequencies) to simulate genetic drift. Size of the overall population is held constant over generations. Simulations are stopped when a Glu allele (either CAA or CAG) is fixed (>99.99% of the population) or when the number of generation exceed 10^7^. The generation number and the identity of the fixed codon are then recorded. The results presented in Figure 4 derive from 2,000 repetitions with a population size of 10^8^ and a mutation rate of 10^−9^. Variations of these parameters do not qualitatively affect the results. The fitness values used in these simulations are derived from experimental data. All genotypes have been reconstructed by site-directed mutagenesis. Growth rate were measured on various concentration of isepamycin and fitness value drawn to qualitatively reflect these measures.

## Supporting Information

Figure S1 Relative Evolutionary Potentials of the different synonymous codons.
(0.04 MB DOC)

Figure S2 Alignment of aac_WT_ and aac_ELP_ sequences.
(0.04 MB DOC)

Figure S3 Number of mutations and protein space exploration.
(0.03 MB DOC)

Table S1 Oligonucleotides used in this study.
(0.04 MB DOC)

Table S2 Properties of the mutant libraries.
(0.03 MB DOC)

Dataset S1 Alignment of the aac(6′)-Ib homologs identified by BlastP.
(0.27 MB DOC)

Text S1 Modeling of the relationship between protein space exploration and library size.
(0.10 MB DOC)
