# Supplementary material for: Synonymous Genes Explore Different Evolutionary Landscapes
Source: PLoS Genet. 2008 Nov 14;4(11):e1000256. doi: 10.1371/journal.pgen.1000256 (PMC2575237; doi:10.1371/journal.pgen.1000256)
Supplement: Figure S1 — Relative Evolutionary Potentials of the different synonymous codons. (0.04 MB DOC) [file pgen.1000256.s001.doc]

# Figure S1: Relative Evolutionary Potentials of the different synonymous codons.

Ten amino-acids display synonymous codons with different evolutionary potential from each other. These include all 6-fold degenerate amino-acids: leucine, serine and arginine; five out of six 4-fold degenerate amino-acids: proline, threonine, valine, alanine and glycine; and finally isoleucine and lysine. These tables show the REP for every pair of synonymous codons corresponding to these amino-acids. We define the Relative Evolutionary Potential of codon XXX relative to its synonymous counterpart YYY (REP_XXX/YYY_) as the number of different amino-acids reachable from XXX but not from YYY through single mutation. Note that the REP is not a symmetrical index.
