# Supplementary material for: Synonymous Genes Explore Different Evolutionary Landscapes
Source: PLoS Genet. 2008 Nov 14;4(11):e1000256. doi: 10.1371/journal.pgen.1000256 (PMC2575237; doi:10.1371/journal.pgen.1000256)
Supplement: Figure S2 — Alignment of aacWT and aacELP sequences. (0.04 MB DOC) [file pgen.1000256.s002.doc]

# **Figure S2:** Alignment of aac_WT_ and aac_ELP_ sequences

#
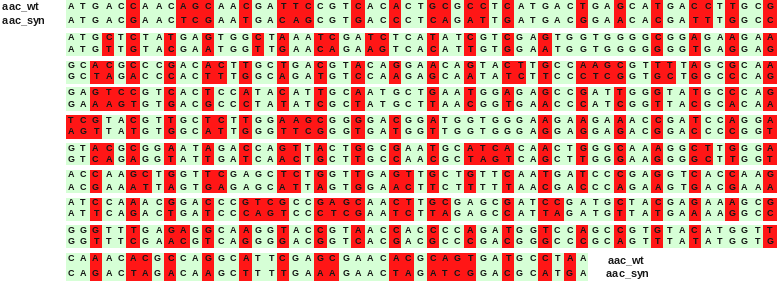


***aac _WT_***

***aac _ELP_***

While encoding identical proteins, *aac_WT_* and *aac_ELP_* only share 61% identity. In this figure, different bases are highlighted in red. Overall, 119 codons out of 184 are different between the two sequences.
