# Supplementary material for: Synonymous Genes Explore Different Evolutionary Landscapes
Source: PLoS Genet. 2008 Nov 14;4(11):e1000256. doi: 10.1371/journal.pgen.1000256 (PMC2575237; doi:10.1371/journal.pgen.1000256)
Supplement: Figure S3 — Number of mutations and protein space exploration. (0.03 MB DOC) [file pgen.1000256.s003.doc]

# Figure S3: Number of mutations and protein space exploration

|  | | ***Min. number of mutations*** | | |
| --- | --- | --- | --- | --- |
|  |  | ***1*** | ***2*** | ***3*** |
| ***Average % of aa accessible*** | ***From single codon*** | 30 | 51 | 19 |
|  | ***From all synonymous codons*** | 40 | 53 | 7 |

No amino acid shows more than four codons with different REP. Thus, at any position, a set of four ELP-designed sequences accesses the same evolutionary landscape as do all the synonymous codons corresponding to the position considered. We compared the minimum number of mutations necessary to reach the other 19 aa from either a single codon or all synonymous codons. This figure summarizes the percentage of amino acid accessible in 1, 2 or 3 mutations averaged over the 61 single codon or the 20 sets of synonymous codons. The use of four ELP-designed sequences, achieves a shift toward a lower number of mutations. It drastically decreases the number of substitutions requiring three mutations by codon.
