# Supplementary material for: Synonymous Genes Explore Different Evolutionary Landscapes
Source: PLoS Genet. 2008 Nov 14;4(11):e1000256. doi: 10.1371/journal.pgen.1000256 (PMC2575237; doi:10.1371/journal.pgen.1000256)
Supplement: Table S1 — Oligonucleotides used in this study. (0.04 MB DOC) [file pgen.1000256.s004.doc]

# Table S1: Oligonucleotides used in this study

| **Name** | **Sequence (5' 🡪 3')^3^** |
| --- | --- |
| AACelp_t1^1^ | pAATTCATATGACGAACTCGAATGACAGCGTGACCCTCAGATTGATGACGGAACACGATTTGGCCATGTTGTAC |
| AACelp_t2^1^ | pGAATGGTTGAACAGAAGTCACATTGTGGAATGGTGGGGGGGTGAGGAGGCTAGACCCACTTTGGCAGATG |
| AACelp_t3^1^ | pTCCAAGAGCAATATCTTCCCTCGGTGCTGGCCCAGGAAAGTGTGACGCCCTATATCGCTATGCTTAACGG |
| AACelp_t4^1^ | pTGAACCCATCGGTTACGCACAAAGTTATGTGGCATTGGGTTCGGGTGATGGTTGGTGGGAGGAGGAGACG |
| AACelp_t5^1^ | pGACCCCGGTGTCAGAGGTATTGATCAACTGCTTGCCAGGTTCGGGTGATGGTTGGTGGGAGGAGGAGACG |
| AACelp_t6^1^ | pGACCCCGGTGTCAGAGGTATTGATCAACTGCTTGCCACCCAGAAGTGACGAAAATTCAGACTGATCCCAG |
| AACelp_t7^1^ | pTCCCTCGAATCTTAGAGCCATTAGATGTTATGAAAAGGCCGGTTTCGAACGTCAGGGGACGGTCACGACG |
| AACelp_t8^1^ | pCCCGACGGGCCCGCAGTTTATATGGTGCAGACTAGACAAGCTTTTGAAAGAACTAGATCGGACGCATGAG |
| AACelp_b1^1^ | pCAATCTGAGGGTCACGCTGTCATTCGAGTTCGTCATATG |
| AACelp_b2^1^ | pCCCACCATTCCACAATGTGACTTCTGTTCAACCATTCGTACAACATGGCCAAATCGTGTTCCGTCATATG |
| AACelp_b3^1^ | pTCCTGGGCCAGCACCGAGGGAAGATATTGCTCTTGGACATCTGCCAAAGTGGGTCTAGCCTCCTCACCCC |
| AACelp_b4^1^ | pCAATGCCACATAACTTTGTGCGTAACCGATGGGTTCACCGTTAAGCATAGCGATATAGGGCGTCACACTT |
| AACelp_b5^1^ | pTGGCAAGCAGTTGATCAATACCTCTGACACCGGGGTCCGTCTCCTCCTCCCACCAACCATCACCCGAACC |
| AACelp_b6^1^ | pTGGCAAGCAGTTGATCAATACCTCTGACACCGGGGTCCGTCTCCTCCTCCCACCAACCATCACCCGAACC |
| AACelp_b7^1^ | pCTTTTCATAACATCTAATGGCTCTAAGATTCGAGGGACTGGGATCAGTCTGAATTTTCGTCACTTCTGGG |
| AACelp_b8^1^ | pGTCTAGTCTGCACCATATAAACTGCGGGCCCGTCGGGCGTCGTGACCGTCCCCTGACGTTCGAAACCGGC |
| AACelp_b9^1^ | pGATCCTCATGCGTCCGATCTAGTTCTTTCAAAAGCTT |
| AACmut_Rw^2^ | TGTGAGCGGATAACAATTTCACACAGgaattcAT |
| AACmut_Fw^2^ | GCATGCCTGCAGGTCGACTCTAGAggatcc |

^1^ AACelps were designed to construct the synthetic gene *aac_ELP_*

^2^ AACmuts were used to amplify cloned genes

^3^ The letter *p* indicates phosphorylation
