# Supplementary material for: Synonymous Genes Explore Different Evolutionary Landscapes
Source: PLoS Genet. 2008 Nov 14;4(11):e1000256. doi: 10.1371/journal.pgen.1000256 (PMC2575237; doi:10.1371/journal.pgen.1000256)
Supplement: Table S2 — Properties of the mutant libraries. (0.03 MB DOC) [file pgen.1000256.s005.doc]

# Table S2: Properties of the mutant libraries

|  | **#** | **Size^2^** | **Mut. Rate^3^** |
| --- | --- | --- | --- |
|  |  |  |  |
| ***aac_WT_*^1^** | 1 | >10^6^ | 0.5 |
|  | 2 | >10^6^ | 1.3 |
|  | 3 | >10^6^ | 3.1 |
|  | 4 | >10^6^ | 5.2 |
| ***aac_ELP_*^1^** | 1 | >10^6^ | 1.3 |
|  | 2 | >10^6^ | 0.9 |
|  | 3 | >10^6^ | 2.5 |
|  | 4 | >10^6^ | 3.2 |

^1^ Libraries were generated by error-prone PCR from each version of the gene *aac(6’)-Ib*.

^2^ Each pool contained approximately the same number of clones, as estimated on plates before selection.

^3^ The mean mutation rate is 2.5 mut./kb for *aac_WT_* and 2 mut./kb for *aac_ELP_*
