# Supplementary material for: Synonymous Genes Explore Different Evolutionary Landscapes
Source: PLoS Genet. 2008 Nov 14;4(11):e1000256. doi: 10.1371/journal.pgen.1000256 (PMC2575237; doi:10.1371/journal.pgen.1000256)
Supplement: Dataset S1 — Alignment of the aac(6′)-Ib homologs identified by BlastP. (0.27 MB DOC) [file pgen.1000256.s006.doc]

**Data S1:** Alignment of the aac(6')-Ib homologs identified by BlastP

The protein sequence AAC(6')-Ib was blasted again the NCBI nr protein database as of 2007/08/26. Corresponding nucleotide sequences were fetched, sorted and aligned using a dedicated BioPerl script.

>AF355189 Pseudomonas aeruginosa integron In60 integrase IntI1 (intI1), aminoglycoside 3-N-acetyltransferase/aminoglycoside 6'-N-acetyltransferase fusion protein (aac(3)-Ib/aac(6')-Ib), and beta-lactamase GES-1 (blages-1) genes, complete cds.

TTGACCAACAGC---AACGATTCCGTCACACTGCGCCTCATGACTGAGCATGACCTTGCGATGCTCTATGAGTGGGTAAATCGATCTCATATCGTCGAGTGGTGGGGCGGAGAAGAAGCACGCCCGACACTTGCTGACGTACAGGAACAGTACTTGCCAAGCGTTTTAGCGCAAGAGTCCGTCACTCCATACATTGCAATGCTGAATGGAGAGCCGATTGGGTATGCCCAGTCGTACGTTGCTCTTGGAGGCGGGGACGGATGGTGGGAAGAAGAAACCGATCCAGGAGTACGCGGAATAGACCAGTCACTGGCGAATGCATCACAACTGGGCAAAGGCTTGGGAACCAAGCTGGTTCGAGCTCTGGTTGAGTTGCTGTTCAATGATCCCGAGGTCACCAAGATCCAAACGGACCCGTCGCCGAGCAACTTGCGAGCGATCCGATGCTACGAGAAAGCGGGGTTTGAGAGGCAAGGTACCGTAACCACCCCAGATGGTCCAGCCGTGTACATGGTTCAAACACGCCAGGCATTCGAGCGAACACGCAGTGATGCCTAA

>AY370764 Klebsiella pneumoniae plasmid pKP31 sulI-type integron integrase (intI1) gene, partial cds, aminoglycoside 6-N-acetyltransferase (aac(6')-Ib) gene, complete cds, and QacEdelta1 (qacEdelta1) gene, partial cds.

GTGACCAACAGC---AACGATTCCGTCACACTGCGCCTCATGACTGAGCATGACCTTGCGATGCTCTATGAGTGGCTAAATCGATCTCATATCGTCGAGTGGTGGGGCGGAGAAGAAGCACGCCCGACACTTGCTGACGTACAGGAACAGTACTTGCCAAGCGTTTTAGCGCAAGAGTCCGTCACTCCATACATTGCAATGCTGAATGGAGAGCCGATTGGGTATGCCCAGTCGTACGTTGCTCTTGGAAGCGGGGACGGATGGTGGGAAGAAGAAACCGATCCAGGAGTACGCGGAATAGACCAGTTACTGGCGAATGCATCACAACTGGGCAAAGGCTTGGGAACCAAGCTGGTTCGAGCTCTGGTTGAGTTGCTGTTCAATGATCCCGAGGTCACCAAGATCCAAACGGACCCGTCGCCGAGCAACTTGCGAGCGATCCGATGCTACGAGAAAGCGGGGTTTGAGAGGCAAGGTACCGTAACCACCCCAGATGGTCCAGCCGTGTACATGGTTCAAACACGCCAGGCATTCGAGCGAACACGCAGTGATGCCTAA

>PAU59183 Pseudomonas aeruginosa integrase (int) gene, partial cds; and aminoglycoside 6'-N-acetyltransferase (aac(6')-Ib) and beta-lactamase (bla oxa-13) genes, complete cds.

GTGACCAACAGC---AACGATTCCGTCACACTGCGCCTCATGACTGAGCATGACCTTGCGATGCTCTATGAGTGGCTAAATCGATCTCATATCGTCGAGTGGTGGGGCGGAGAAGAAGCACGCCCGACACTTGCTGACGTACAGGAACAGTACTTGCCAAGCGTTTTAGCGCAAGAGTCCGTCACTCCATACATTGCAATGCTGAATGGAGAGCCGATTGGGTATGCCCAGTCGTACGTTGCTCTTGGAAGCGGGGACGGATGGTGGGAAGAAGAAACCGATCCAGGAGTACGCGGAATAGACCAGTTACTGGCGAATGCATCACAACTGGGCAAAGGCTTGGGAACCAAGCTGGTTCGAGCTCTGGTTGAGTTGCTGTTCAATGATCCCGAGGTCACCAAGATCCAAACGGACCCGTCGCCGAGCAACTTGCGAGCGATCCGATGCTACGAGAAAGCGGGGTTTGAGAGGCAAGGTACCGTAACCACCCCAGATGGTCCAGCCGTGTACATGGTTCAAACACGCCAGGCATTCGAGCGAACACGCAGTGATGCCTAA

>AF207065 Klebsiella pneumoniae DNA integrase (int) gene, partial cds; aminoglycoside 6'-N-acetyltransferase (aacC4) and aminoglycoside 3'-N-acetyltransferase (aac(3')-I) genes, complete cds; and ethidium bromide-resistance protein (qacdelta1) gene, partial cds.

GTGACCAACAGC---AACGATTCCGTCACACTGCGCCTCATGACTGAGCATGACCTTGCGATGCTCTATGAGTGGCTAAATCGATCTCATATCGTCGAGTGGTGGGGCGGAGAAGAAGCACGCCCGACACTTGCTGACGTACAGGAACAGTACTTGCCAAGCGTTTTAGCGCAAGAGTCCGTCACTCCATACATTGCAATGCTGAATGGAGAGCCGATTGGGTATGCCCAGTCGTACGTTGCTCTTGGAAGCGGGGACGGATGGTGGGAAGAAGAAACCGATCCAGGAGTACGCGGAATAGACCAGTTACTGGCGAATGCATCACAACTGGGCAAAGGCTTGGGAACCAAGCTGGTTCGAGCTCTGGTTGAGTTGCTGTTCAATGATCCCGAGGTCACCAAGATCCAAACGGACCCGTCGCCGAGCAACTTGCGAGCGATCCGATGCTACGAGAAAGCGGGGTTTGAGAGGCAAGGTACCGTAACCACCCCAGATGGTCCAGCCGTGTACATGGTTCAAACACGCCAGGCATTCGAGCGAACACGCAGTGATGCCTAA

>AF458080 Klebsiella pneumoniae strain ILT-3 class I integron, Tn1721 transposon, and ISEcp1B and IS903D insertion sequences.

GTGACCAACAGC---AACGATTCCGTCACACTGCGCCTCATGACTGAGCATGACCTTGCGATGCTCTATGAGTGGCTAAATCGATCTCATATCGTCGAGTGGTGGGGCGGAGAAGAAGCACGCCCGACACTTGCTGACGTACAGGAACAGTACTTGCCAAGCGTTTTAGCGCAAGAGTCCGTCACTCCATACATTGCAATGCTGAATGGAGAGCCGATTGGGTATGCCCAGTCGTACGTTGCTCTTGGAAGCGGGGACGGATGGTGGGAAGAAGAAACCGATCCAGGAGTACGCGGAATAGACCAGTTACTGGCGAATGCATCACAACTGGGCAAAGGCTTGGGAACCAAGCTGGTTCGAGCTCTGGTTGAGTTGCTGTTCAATGATCCCGAGGTCACCAAGATCCAAACGGACCCGTCGCCGAGCAACTTGCGAGCGATCCGATGCTACGAGAAAGCGGGGTTTGAGAGGCAAGGTACCGTAACCACCCCAGATGGTCCAGCCGTGTACATGGTTCAAACACGCCAGGCATTCGAGCGAACACGCAGTGATGCCTAA

>X60321 Pseudomonas aeruginosa plasmid pCFF04 aacA4 gene for 6'-N-aminoglycoside acetyltransferase.

GTGACCAACAGC---AACGATTCCGTCACACTGCGCCTCATGACTGAGCATGACCTTGCGATGCTCTATGAGTGGCTAAATCGATCTCATATCGTCGAGTGGTGGGGCGGAGAAGAAGCACGCCCGACACTTGCTGACGTACAGGAACAGTACTTGCCAAGCGTTTTAGCGCAAGAGTCCGTCACTCCATACATTGCAATGCTGAATGGAGAGCCGATTGGGTATGCCCAGTCGTACGTTGCTCTTGGAAGCGGGGACGGATGGTGGGAAGAAGAAACCGATCCAGGAGTACGCGGAATAGACCAGTTACTGGCGAATGCATCACAACTGGGCAAAGGCTTGGGAACCAAGCTGGTTCGAGCTCTGGTTGAGTTGCTGTTCAATGATCCCGAGGTCACCAAGATCCAAACGGACCCGTCGCCGAGCAACTTGCGAGCGATCCGATGCTACGAGAAAGCGGGGTTTGAGAGGCAAGGTACCGTAACCACCCCAGATGGTCCAGCCGTGTACATGGTTCAAACACGCCAGGCATTCGAGCGAACACGCAGTGATGCCTAA

>AB212941 Escherichia coli aacA4, cmlA1 genes for aminoglycoside acetyltrans, chloramphenicol resistance, complete cds.

GTGACCAACAGC---AACGATTCCGTCACACTGCGCCTCATGACTGAGCATGACCTTGCGATGCTCTATGAGTGGCTAAATCGATCTCATATCGTCGAGTGGTGGGGCGGAGAAGAAGCACGCCCGACACTTGCTGACGTACAGGAACAGTACTTGCCAAGCGTTTTAGCGCAAGAGTCCGTCACTCCATACATTGCAATGCTGAATGGAGAGCCGATTGGGTATGCCCAGTCGTACGTTGCTCTTGGAAGCGGGGACGGATGGTGGGAAGAAGAAACCGATCCAGGAGTACGCGGAATAGACCAGTTACTGGCGAATGCATCACAACTGGGCAAAGGCTTGGGAACCAAGCTGGTTCGAGCTCTGGTTGAGTTGCTGTTCAATGATCCCGAGGTCACCAAGATCCAAACGGACCCGTCGCCGAGCAACTTGCGAGCGATCCGATGCTACGAGAAAGCGGGGTTTGAGAGGCAAGGTACCGTAACCACCCCAGATGGTCCAGCCGTGTACATGGTTCAAACACGCCAGGCATTCGAGCGAACACGCAGTGATGCCTAA

>AB214531 Pseudomonas aeruginosa class I integron aacA4 and mutated-cmlA1 genes, complete cds.

GTGACCAACAGC---AACGATTCCGTCACACTGCGCCTCATGACTGAGCATGACCTTGCGATGCTCTATGAGTGGCTAAATCGATCTCATATCGTCGAGTGGTGGGGCGGAGAAGAAGCACGCCCGACACTTGCTGACGTACAGGAACAGTACTTGCCAAGCGTTTTAGCGCAAGAGTCCGTCACTCCATACATTGCAATGCTGAATGGAGAGCCGATTGGGTATGCCCAGTCGTACGTTGCTCTTGGAAGCGGGGACGGATGGTGGGAAGAAGAAACCGATCCAGGAGTACGCGGAATAGACCAGTTACTGGCGAATGCATCACAACTGGGCAAAGGCTTGGGAACCAAGCTGGTTCGAGCTCTGGTTGAGTTGCTGTTCAATGATCCCGAGGTCACCAAGATCCAAACGGACCCGTCGCCGAGCAACTTGCGAGCGATCCGATGCTACGAGAAAGCGGGGTTTGAGAGGCAAGGTACCGTAACCACCCCAGATGGTCCAGCCGTGTACATGGTTCAAACACGCCAGGCATTCGAGCGAACACGCAGTGATGCCTAA

>AY912492 Escherichia coli class I integron chloramphenicol resistance protein cmlA1-variant-like gene, complete sequence, and aminoglycoside acetyltransferase AAC(6')-Ib (aacA4) gene, complete cds.

GTGACCAACAGC---AACGATTCCGTCACACTGCGCCTCATGACTGAGCATGACCTTGCGATGCTCTATGAGTGGCTAAATCGATCTCATATCGTCGAGTGGTGGGGCGGAGAAGAAGCACGCCCGACACTTGCTGACGTACAGGAACAGTACTTGCCAAGCGTTTTAGCGCAAGAGTCCGTCACTCCATACATTGCAATGCTGAATGGAGAGCCGATTGGGTATGCCCAGTCGTACGTTGCTCTTGGAAGCGGGGACGGATGGTGGGAAGAAGAAACCGATCCAGGAGTACGCGGAATAGACCAGTTACTGGCGAATGCATCACAACTGGGCAAAGGCTTGGGAACCAAGCTGGTTCGAGCTCTGGTTGAGTTGCTGTTCAATGATCCCGAGGTCACCAAGATCCAAACGGACCCGTCGCCGAGCAACTTGCGAGCGATCCGATGCTACGAGAAAGCGGGGTTTGAGAGGCAAGGTACCGTAACCACCCCAGATGGTCCAGCCGTGTACATGGTTCAAACACGCCAGGCATTCGAGCGAACACGCAGTGATGCCTAA

>EF690695 Acinetobacter baumannii class I integron In-Ab10a integrase (intI) gene, partial cds, ORF11 (orf11), ACC (6')-Ib (aac (6')-Ib), and VIM-1 (blaVIM-1) genes, complete cds, and QacEdelta1 (qacEdelta1) gene, partial cds.

GTGACCAACAGC---AACGATTCCGTCACACTGCGCCTCATGACTGAGCATGACCTTGCGATGCTCTATGAGTGGCTAAATCGATCTCATATCGTCGAGTGGTGGGGCGGAGAAGAAGCACGCCCGACACTTGCTGACGTACAGGAACAGTACTTGCCAAGCGTTTTAGCGCAAGAGTCCGTCACTCCATACATTGCAATGCTGAATGGAGAGCCGATTGGGTATGCCCAGTCGTACGTTGCTCTTGGAAGCGGGGACGGATGGTGGGAAGAAGAAACCGATCCAGGAGTACGCGGAATAGACCAGTTACTGGCGAATGCATCACAACTGGGCAAAGGCTTGGGAACCAAGCTGGTTCGAGCTCTGGTTGAGTTGCTGTTCAATGATCCCGAGGTCACCAAGATCCAAACGGACCCGTCGCCGAGCAACTTGCGAGCGATCCGATGCTACGAGAAAGCGGGGTTTGAGAGGCAAGGTACCGTAACCACCCCAGATGGTCCAGCCGTGTACATGGTTCAAACACGCCAGGCATTCGAGCGAACACGCAGTGATGCCTAA

>AB291064 Staphylococcus epidermidis class 1 integron aacA4, cmlA1 genes for aminoglycoside acetyltrans, chloramphenicol resistance, complete cds.

GTGACCAACAGC---AACGATTCCGTCACACTGCGCCTCATGACTGAGCATGACCTTGCGATGCTCTATGAGTGGCTAAATCGATCTCATATCGTCGAGTGGTGGGGCGGAGAAGAAGCACGCCCGACACTTGCTGACGTACAGGAACAGTACTTGCCAAGCGTTTTAGCGCAAGAGTCCGTCACTCCATACATTGCAATGCTGAATGGAGAGCCGATTGGGTATGCCCAGTCGTACGTTGCTCTTGGAAGCGGGGACGGATGGTGGGAAGAAGAAACCGATCCAGGAGTACGCGGAATAGACCAGTTACTGGCGAATGCATCACAACTGGGCAAAGGCTTGGGAACCAAGCTGGTTCGAGCTCTGGTTGAGTTGCTGTTCAATGATCCCGAGGTCACCAAGATCCAAACGGACCCGTCGCCGAGCAACTTGCGAGCGATCCGATGCTACGAGAAAGCGGGGTTTGAGAGGCAAGGTACCGTAACCACCCCAGATGGTCCAGCCGTGTACATGGTTCAAACACGCCAGGCATTCGAGCGAACACGCAGTGATGCCTAA

>AB291065 Staphylococcus hominis class 1 integron aacA4, cmlA1 genes for aminoglycoside acetyltrans, chloramphenicol resistance, complete cds.

GTGACCAACAGC---AACGATTCCGTCACACTGCGCCTCATGACTGAGCATGACCTTGCGATGCTCTATGAGTGGCTAAATCGATCTCATATCGTCGAGTGGTGGGGCGGAGAAGAAGCACGCCCGACACTTGCTGACGTACAGGAACAGTACTTGCCAAGCGTTTTAGCGCAAGAGTCCGTCACTCCATACATTGCAATGCTGAATGGAGAGCCGATTGGGTATGCCCAGTCGTACGTTGCTCTTGGAAGCGGGGACGGATGGTGGGAAGAAGAAACCGATCCAGGAGTACGCGGAATAGACCAGTTACTGGCGAATGCATCACAACTGGGCAAAGGCTTGGGAACCAAGCTGGTTCGAGCTCTGGTTGAGTTGCTGTTCAATGATCCCGAGGTCACCAAGATCCAAACGGACCCGTCGCCGAGCAACTTGCGAGCGATCCGATGCTACGAGAAAGCGGGGTTTGAGAGGCAAGGTACCGTAACCACCCCAGATGGTCCAGCCGTGTACATGGTTCAAACACGCCAGGCATTCGAGCGAACACGCAGTGATGCCTAA

>AF550679 Escherichia coli plasmid p1658/97, complete sequence.

GTGACCAACAGC---AACGATTCCGTCACACTGCGCCTCATGACTGAGCATGACCTTGCGATGCTCTATGAGTGGCTAAATCGATCTCATATCGTCGAGTGGTGGGGCGGAGAAGAAGCACGCCCGACACTTGCTGACGTACAGGAACAGTACTTGCCAAGCGTTTTAGCGCAAGAGTCCGTCACTCCATACATTGCAATGCTGAATGGAGAGCCGATTGGGTATGCCCAGTCGTACGTTGCTCTTGGAAGCGGGGACGGATGGTGGGAAGAAGAAACCGATCCAGGAGTACGCGGAATAGACCAGTTACTGGCGAATGCATCACAACTGGGCAAAGGCTTGGGAACCAAGCTGGTTCGAGCTCTGGTTGAGTTGCTGTTCAATGATCCCGAGGTCACCAAGATCCAAACGGACCCGTCGCCGAGCAACTTGCGAGCGATCCGATGCTACGAGAAAGCGGGGTTTGAGAGGCAAGGTACCGTAACCACCCCAGATGGTCCAGCCGTGTACATGGTTCAAACACGCCAGGCATTCGAGCGAACACGCAGTGATGCCTAA

>AF034958 Enterobacter aerogenes plasmid pIP833 integron In40 6'-N-aminoglycoside acetyltransferase (aac(6')-Ib), quarternary ammonium determinant (qacF), chloramphenicol resistance determinant (cmlB), oxacillinase (oxa9), and sulfonamides resistance determinant (sul1) genes, complete cds; and unknown gene.

GTGACCAACAGC---AACGATTCCGTCACACTGCGCCTCATGACTGAGCATGACCTTGCGATGCTCTATGAGTGGCTAAATCGATCTCATATCGTCGAGTGGTGGGGCGGAGAAGAAGCACGCCCGACACTTGCTGACGTACAGGAACAGTACTTGCCAAGCGTTTTAGCGCAAGAGTCCGTCACTCCATACATTGCAATGCTGAATGGAGAGCCGATTGGGTATGCCCAGTCGTACGTTGCTCTTGGAAGCGGGGACGGATGGTGGGAAGAAGAAACCGATCCAGGAGTACGCGGAATAGACCAGTTACTGGCGAATGCATCACAACTGGGCAAAGGCTTGGGAACCAAGCTGGTTCGAGCTCTGGTTGAGTTGCTGTTCAATGATCCCGAGGTCACCAAGATCCAAACGGACCCGTCGCCGAGCAACTTGCGAGCGATCCGATGCTACGAGAAAGCGGGGTTTGAGAGGCAAGGTACCGTAACCACCCCAGATGGTCCAGCCGTGTACATGGTTCAAACACGCCAGGCATTCGAGCGAACACGCAGTGATGCCTAA

>AF282595 Klebsiella pneumoniae integron INTCZD37 DNA integrase gene, partial cds; 6'-N-aminoglycoside acetyltransferase (aac(6')-Ib), 3-N-aminoglycoside acetyltransferase (aac(3)-Ia), aminoglycoside adenyltransferase AAD(3') (aadA1a), and quaternary ammonium compound-resistance protein (qacEdelta1) genes, complete cds; and unknown genes.

GTGACCAACAGC---AACGATTCCGTCACACTGCGCCTCATGACTGAGCATGACCTTGCGATGCTCTATGAGTGGCTAAATCGATCTCATATCGTCGAGTGGTGGGGCGGAGAAGAAGCACGCCCGACACTTGCTGACGTACAGGAACAGTACTTGCCAAGCGTTTTAGCGCAAGAGTCCGTCACTCCATACATTGCAATGCTGAATGGAGAGCCGATTGGGTATGCCCAGTCGTACGTTGCTCTTGGAAGCGGGGACGGATGGTGGGAAGAAGAAACCGATCCAGGAGTACGCGGAATAGACCAGTTACTGGCGAATGCATCACAACTGGGCAAAGGCTTGGGAACCAAGCTGGTTCGAGCTCTGGTTGAGTTGCTGTTCAATGATCCCGAGGTCACCAAGATCCAAACGGACCCGTCGCCGAGCAACTTGCGAGCGATCCGATGCTACGAGAAAGCGGGGTTTGAGAGGCAAGGTACCGTAACCACCCCAGATGGTCCAGCCGTGTACATGGTTCAAACACGCCAGGCATTCGAGCGAACACGCAGTGATGCCTAA

>KOU90945 Klebsiella oxytoca plasmid pACM1 quaternary ammonium compound transporter protein (qacE delta 1) gene, partial cds; 3''-0-adenyltransferase (ant(3'')-Ia), 3-N-aminoglycoside acetyltransferase (aac(3)-Ia), and 6'-N-aminoglycoside acetyltransferase (aac(6')-Ib) genes, complete cds; DNA integrase (intI1) gene, partial cds; and unknown genes.

GTGACCAACAGC---AACGATTCCGTCACACTGCGCCTCATGACTGAGCATGACCTTGCGATGCTCTATGAGTGGCTAAATCGATCTCATATCGTCGAGTGGTGGGGCGGAGAAGAAGCACGCCCGACACTTGCTGACGTACAGGAACAGTACTTGCCAAGCGTTTTAGCGCAAGAGTCCGTCACTCCATACATTGCAATGCTGAATGGAGAGCCGATTGGGTATGCCCAGTCGTACGTTGCTCTTGGAAGCGGGGACGGATGGTGGGAAGAAGAAACCGATCCAGGAGTACGCGGAATAGACCAGTTACTGGCGAATGCATCACAACTGGGCAAAGGCTTGGGAACCAAGCTGGTTCGAGCTCTGGTTGAGTTGCTGTTCAATGATCCCGAGGTCACCAAGATCCAAACGGACCCGTCGCCGAGCAACTTGCGAGCGATCCGATGCTACGAGAAAGCGGGGTTTGAGAGGCAAGGTACCGTAACCACCCCAGATGGTCCAGCCGTGTACATGGTTCAAACACGCCAGGCATTCGAGCGAACACGCAGTGATGCCTAA

>AJ313334 Acinetobacter baumannii partial intI1 gene for class I integron DNA integrase and aacA4 gene for aminoglycoside 6'-N-acetyltransferase.

GTGACCAACAGC---AACGATTCCGTCACACTGCGCCTCATGACTGAGCATGACCTTGCGATGCTCTATGAGTGGCTAAATCGATCTCATATCGTCGAGTGGTGGGGCGGAGAAGAAGCACGCCCGACACTTGCTGACGTACAGGAACAGTACTTGCCAAGCGTTTTAGCGCAAGAGTCCGTCACTCCATACATTGCAATGCTGAATGGAGAGCCGATTGGGTATGCCCAGTCGTACGTTGCTCTTGGAAGCGGGGACGGATGGTGGGAAGAAGAAACCGATCCAGGAGTACGCGGAATAGACCAGTTACTGGCGAATGCATCACAACTGGGCAAAGGCTTGGGAACCAAGCTGGTTCGAGCTCTGGTTGAGTTGCTGTTCAATGATCCCGAGGTCACCAAGATCCAAACGGACCCGTCGCCGAGCAACTTGCGAGCGATCCGATGCTACGAGAAAGCGGGGTTTGAGAGGCAAGGTACCGTAACCACCCCAGATGGTCCAGCCGTGTACATGGTTCAAACACGCCAGGCATTCGAGCGAACACGCAGTGATGCCTAA

>AJ319747 Acinetobacter baumannii intI1 gene (partial), aacA4 gene, ORF, oxa-20 gene and qacEdelta1 gene (partial).

GTGACCAACAGC---AACGATTCCGTCACACTGCGCCTCATGACTGAGCATGACCTTGCGATGCTCTATGAGTGGCTAAATCGATCTCATATCGTCGAGTGGTGGGGCGGAGAAGAAGCACGCCCGACACTTGCTGACGTACAGGAACAGTACTTGCCAAGCGTTTTAGCGCAAGAGTCCGTCACTCCATACATTGCAATGCTGAATGGAGAGCCGATTGGGTATGCCCAGTCGTACGTTGCTCTTGGAAGCGGGGACGGATGGTGGGAAGAAGAAACCGATCCAGGAGTACGCGGAATAGACCAGTTACTGGCGAATGCATCACAACTGGGCAAAGGCTTGGGAACCAAGCTGGTTCGAGCTCTGGTTGAGTTGCTGTTCAATGATCCCGAGGTCACCAAGATCCAAACGGACCCGTCGCCGAGCAACTTGCGAGCGATCCGATGCTACGAGAAAGCGGGGTTTGAGAGGCAAGGTACCGTAACCACCCCAGATGGTCCAGCCGTGTACATGGTTCAAACACGCCAGGCATTCGAGCGAACACGCAGTGATGCCTAA

>AY152821 Escherichia coli class I integron integrase (intI) gene, partial cds; 6'-N-aminoglycoside acetyltransferase aac(6')-Ib (aac 6' Ib), blaVIM-1 (vim-1), and quartenary ammonium resistance protein (qacEdelta1) genes, complete cds; and sulfonamide resistance protein (sulI) gene, partial cds.

GTGACCAACAGC---AACGATTCCGTCACACTGCGCCTCATGACTGAGCATGACCTTGCGATGCTCTATGAGTGGCTAAATCGATCTCATATCGTCGAGTGGTGGGGCGGAGAAGAAGCACGCCCGACACTTGCTGACGTACAGGAACAGTACTTGCCAAGCGTTTTAGCGCAAGAGTCCGTCACTCCATACATTGCAATGCTGAATGGAGAGCCGATTGGGTATGCCCAGTCGTACGTTGCTCTTGGAAGCGGGGACGGATGGTGGGAAGAAGAAACCGATCCAGGAGTACGCGGAATAGACCAGTTACTGGCGAATGCATCACAACTGGGCAAAGGCTTGGGAACCAAGCTGGTTCGAGCTCTGGTTGAGTTGCTGTTCAATGATCCCGAGGTCACCAAGATCCAAACGGACCCGTCGCCGAGCAACTTGCGAGCGATCCGATGCTACGAGAAAGCGGGGTTTGAGAGGCAAGGTACCGTAACCACCCCAGATGGTCCAGCCGTGTACATGGTTCAAACACGCCAGGCATTCGAGCGAACACGCAGTGATGCCTAA

>AY307114 Acinetobacter baumannii class 1 integron 6'-N-acetyltransferase (aacA4), hypothetical protein, and OXA-20 (oxa-20) genes, complete cds.

GTGACCAACAGC---AACGATTCCGTCACACTGCGCCTCATGACTGAGCATGACCTTGCGATGCTCTATGAGTGGCTAAATCGATCTCATATCGTCGAGTGGTGGGGCGGAGAAGAAGCACGCCCGACACTTGCTGACGTACAGGAACAGTACTTGCCAAGCGTTTTAGCGCAAGAGTCCGTCACTCCATACATTGCAATGCTGAATGGAGAGCCGATTGGGTATGCCCAGTCGTACGTTGCTCTTGGAAGCGGGGACGGATGGTGGGAAGAAGAAACCGATCCAGGAGTACGCGGAATAGACCAGTTACTGGCGAATGCATCACAACTGGGCAAAGGCTTGGGAACCAAGCTGGTTCGAGCTCTGGTTGAGTTGCTGTTCAATGATCCCGAGGTCACCAAGATCCAAACGGACCCGTCGCCGAGCAACTTGCGAGCGATCCGATGCTACGAGAAAGCGGGGTTTGAGAGGCAAGGTACCGTAACCACCCCAGATGGTCCAGCCGTGTACATGGTTCAAACACGCCAGGCATTCGAGCGAACACGCAGTGATGCCTAA

>AJ585042 Pseudomonas aeruginosa partial int1 gene, aacA4 gene, blaVIM-4 gene and partial qacEdelta1 gene.

GTGACCAACAGC---AACGATTCCGTCACACTGCGCCTCATGACTGAGCATGACCTTGCGATGCTCTATGAGTGGCTAAATCGATCTCATATCGTCGAGTGGTGGGGCGGAGAAGAAGCACGCCCGACACTTGCTGACGTACAGGAACAGTACTTGCCAAGCGTTTTAGCGCAAGAGTCCGTCACTCCATACATTGCAATGCTGAATGGAGAGCCGATTGGGTATGCCCAGTCGTACGTTGCTCTTGGAAGCGGGGACGGATGGTGGGAAGAAGAAACCGATCCAGGAGTACGCGGAATAGACCAGTTACTGGCGAATGCATCACAACTGGGCAAAGGCTTGGGAACCAAGCTGGTTCGAGCTCTGGTTGAGTTGCTGTTCAATGATCCCGAGGTCACCAAGATCCAAACGGACCCGTCGCCGAGCAACTTGCGAGCGATCCGATGCTACGAGAAAGCGGGGTTTGAGAGGCAAGGTACCGTAACCACCCCAGATGGTCCAGCCGTGTACATGGTTCAAACACGCCAGGCATTCGAGCGAACACGCAGTGATGCCTAA

>AY509609 Pseudomonas aeruginosa class I integron OXA-like protein (blaOXA), 6'-N-aminoglycoside acetyltransferase (aacA4), and VIM-4 (blaVIM-4) genes, complete cds.

GTGACCAACAGC---AACGATTCCGTCACACTGCGCCTCATGACTGAGCATGACCTTGCGATGCTCTATGAGTGGCTAAATCGATCTCATATCGTCGAGTGGTGGGGCGGAGAAGAAGCACGCCCGACACTTGCTGACGTACAGGAACAGTACTTGCCAAGCGTTTTAGCGCAAGAGTCCGTCACTCCATACATTGCAATGCTGAATGGAGAGCCGATTGGGTATGCCCAGTCGTACGTTGCTCTTGGAAGCGGGGACGGATGGTGGGAAGAAGAAACCGATCCAGGAGTACGCGGAATAGACCAGTTACTGGCGAATGCATCACAACTGGGCAAAGGCTTGGGAACCAAGCTGGTTCGAGCTCTGGTTGAGTTGCTGTTCAATGATCCCGAGGTCACCAAGATCCAAACGGACCCGTCGCCGAGCAACTTGCGAGCGATCCGATGCTACGAGAAAGCGGGGTTTGAGAGGCAAGGTACCGTAACCACCCCAGATGGTCCAGCCGTGTACATGGTTCAAACACGCCAGGCATTCGAGCGAACACGCAGTGATGCCTAA

>AY702100 Pseudomonas aeruginosa class I integron 6'-N-aminoglycoside acetyltransferase (aacA4) and VIM-4 (blaVIM-4) genes, complete cds.

GTGACCAACAGC---AACGATTCCGTCACACTGCGCCTCATGACTGAGCATGACCTTGCGATGCTCTATGAGTGGCTAAATCGATCTCATATCGTCGAGTGGTGGGGCGGAGAAGAAGCACGCCCGACACTTGCTGACGTACAGGAACAGTACTTGCCAAGCGTTTTAGCGCAAGAGTCCGTCACTCCATACATTGCAATGCTGAATGGAGAGCCGATTGGGTATGCCCAGTCGTACGTTGCTCTTGGAAGCGGGGACGGATGGTGGGAAGAAGAAACCGATCCAGGAGTACGCGGAATAGACCAGTTACTGGCGAATGCATCACAACTGGGCAAAGGCTTGGGAACCAAGCTGGTTCGAGCTCTGGTTGAGTTGCTGTTCAATGATCCCGAGGTCACCAAGATCCAAACGGACCCGTCGCCGAGCAACTTGCGAGCGATCCGATGCTACGAGAAAGCGGGGTTTGAGAGGCAAGGTACCGTAACCACCCCAGATGGTCCAGCCGTGTACATGGTTCAAACACGCCAGGCATTCGAGCGAACACGCAGTGATGCCTAA

>AY866525 Pseudomonas aeruginosa class I integron integrase I (intI) gene, partial cds, metallo-beta-lactamase VIM-4 (blaVIM-4), 6'-N-aminoglycoside acetyltransferase (aacA4), and beta-lactamase OXA-35 (blaOXA-35) genes, complete cds, and QacEdelta1 (qacEdelta1) gene, partial cds.

GTGACCAACAGC---AACGATTCCGTCACACTGCGCCTCATGACTGAGCATGACCTTGCGATGCTCTATGAGTGGCTAAATCGATCTCATATCGTCGAGTGGTGGGGCGGAGAAGAAGCACGCCCGACACTTGCTGACGTACAGGAACAGTACTTGCCAAGCGTTTTAGCGCAAGAGTCCGTCACTCCATACATTGCAATGCTGAATGGAGAGCCGATTGGGTATGCCCAGTCGTACGTTGCTCTTGGAAGCGGGGACGGATGGTGGGAAGAAGAAACCGATCCAGGAGTACGCGGAATAGACCAGTTACTGGCGAATGCATCACAACTGGGCAAAGGCTTGGGAACCAAGCTGGTTCGAGCTCTGGTTGAGTTGCTGTTCAATGATCCCGAGGTCACCAAGATCCAAACGGACCCGTCGCCGAGCAACTTGCGAGCGATCCGATGCTACGAGAAAGCGGGGTTTGAGAGGCAAGGTACCGTAACCACCCCAGATGGTCCAGCCGTGTACATGGTTCAAACACGCCAGGCATTCGAGCGAACACGCAGTGATGCCTAA

>DQ089809 Pseudomonas aeruginosa class I integron In120 Aac6'-Ib (aacA4), Bel-1 (bla(BEL1)), and Smr2 (smr2) genes, complete cds, and AadA5 (aadA5) gene, partial cds.

GTGACCAACAGC---AACGATTCCGTCACACTGCGCCTCATGACTGAGCATGACCTTGCGATGCTCTATGAGTGGCTAAATCGATCTCATATCGTCGAGTGGTGGGGCGGAGAAGAAGCACGCCCGACACTTGCTGACGTACAGGAACAGTACTTGCCAAGCGTTTTAGCGCAAGAGTCCGTCACTCCATACATTGCAATGCTGAATGGAGAGCCGATTGGGTATGCCCAGTCGTACGTTGCTCTTGGAAGCGGGGACGGATGGTGGGAAGAAGAAACCGATCCAGGAGTACGCGGAATAGACCAGTTACTGGCGAATGCATCACAACTGGGCAAAGGCTTGGGAACCAAGCTGGTTCGAGCTCTGGTTGAGTTGCTGTTCAATGATCCCGAGGTCACCAAGATCCAAACGGACCCGTCGCCGAGCAACTTGCGAGCGATCCGATGCTACGAGAAAGCGGGGTTTGAGAGGCAAGGTACCGTAACCACCCCAGATGGTCCAGCCGTGTACATGGTTCAAACACGCCAGGCATTCGAGCGAACACGCAGTGATGCCTAA

>DQ278189 Salmonella enterica subsp. enterica serovar Agona class I integron Aac 6' Ib (aac 6' Ib) gene, complete cds, disrupted beta-lactamase Oxa-30 (oxa-30) gene, partial sequence, insertion sequence IS1 element, complete sequence, AadA1 (aadA1) gene, complete cds, and unknown gene.

GTGACCAACAGC---AACGATTCCGTCACACTGCGCCTCATGACTGAGCATGACCTTGCGATGCTCTATGAGTGGCTAAATCGATCTCATATCGTCGAGTGGTGGGGCGGAGAAGAAGCACGCCCGACACTTGCTGACGTACAGGAACAGTACTTGCCAAGCGTTTTAGCGCAAGAGTCCGTCACTCCATACATTGCAATGCTGAATGGAGAGCCGATTGGGTATGCCCAGTCGTACGTTGCTCTTGGAAGCGGGGACGGATGGTGGGAAGAAGAAACCGATCCAGGAGTACGCGGAATAGACCAGTTACTGGCGAATGCATCACAACTGGGCAAAGGCTTGGGAACCAAGCTGGTTCGAGCTCTGGTTGAGTTGCTGTTCAATGATCCCGAGGTCACCAAGATCCAAACGGACCCGTCGCCGAGCAACTTGCGAGCGATCCGATGCTACGAGAAAGCGGGGTTTGAGAGGCAAGGTACCGTAACCACCCCAGATGGTCCAGCCGTGTACATGGTTCAAACACGCCAGGCATTCGAGCGAACACGCAGTGATGCCTAA

>DQ370505 Acinetobacter baumannii strain P17 class 1 integron 6'-N-aminoglycoside acetyltransferase (aacA4), 3-n-aminoglycoside acetyltransferase (aac(3)-Ia), and aminoglycoside adenyltransferase AAD(3') (aadA1a) genes, complete cds, and unknown genes.

GTGACCAACAGC---AACGATTCCGTCACACTGCGCCTCATGACTGAGCATGACCTTGCGATGCTCTATGAGTGGCTAAATCGATCTCATATCGTCGAGTGGTGGGGCGGAGAAGAAGCACGCCCGACACTTGCTGACGTACAGGAACAGTACTTGCCAAGCGTTTTAGCGCAAGAGTCCGTCACTCCATACATTGCAATGCTGAATGGAGAGCCGATTGGGTATGCCCAGTCGTACGTTGCTCTTGGAAGCGGGGACGGATGGTGGGAAGAAGAAACCGATCCAGGAGTACGCGGAATAGACCAGTTACTGGCGAATGCATCACAACTGGGCAAAGGCTTGGGAACCAAGCTGGTTCGAGCTCTGGTTGAGTTGCTGTTCAATGATCCCGAGGTCACCAAGATCCAAACGGACCCGTCGCCGAGCAACTTGCGAGCGATCCGATGCTACGAGAAAGCGGGGTTTGAGAGGCAAGGTACCGTAACCACCCCAGATGGTCCAGCCGTGTACATGGTTCAAACACGCCAGGCATTCGAGCGAACACGCAGTGATGCCTAA

>EF522838 Pseudomonas putida class I integron integrase gene, partial cds, metallo-beta-lactamase VIM-6 (blaVIM-6), oxacillinase (blaOXA-10), aminoglycoside 6'-N-acetyltransferase (aacA4), and aminoglycoside adenyltransferase (aadA1) genes, complete cds, and quaternary ammonium compound-resistance (qacEdelta1) gene, partial cds.

GTGACCAACAGC---AACGATTCCGTCACACTGCGCCTCATGACTGAGCATGACCTTGCGATGCTCTATGAGTGGCTAAATCGATCTCATATCGTCGAGTGGTGGGGCGGAGAAGAAGCACGCCCGACACTTGCTGACGTACAGGAACAGTACTTGCCAAGCGTTTTAGCGCAAGAGTCCGTCACTCCATACATTGCAATGCTGAATGGAGAGCCGATTGGGTATGCCCAGTCGTACGTTGCTCTTGGAAGCGGGGACGGATGGTGGGAAGAAGAAACCGATCCAGGAGTACGCGGAATAGACCAGTTACTGGCGAATGCATCACAACTGGGCAAAGGCTTGGGAACCAAGCTGGTTCGAGCTCTGGTTGAGTTGCTGTTCAATGATCCCGAGGTCACCAAGATCCAAACGGACCCGTCGCCGAGCAACTTGCGAGCGATCCGATGCTACGAGAAAGCGGGGTTTGAGAGGCAAGGTACCGTAACCACCCCAGATGGTCCAGCCGTGTACATGGTTCAAACACGCCAGGCATTCGAGCGAACACGCAGTGATGCCTAA

>EU091084 Enterobacter cloacae strain E701 complex class I In132::ISCR1::qnrB10 integron, partial sequence.

GTGACCAACAGC---AACGATTCCGTCACACTGCGCCTCATGACTGAGCATGACCTTGCGATGCTCTATGAGTGGCTAAATCGATCTCATATCGTCGAGTGGTGGGGCGGAGAAGAAGCACGCCCGACACTTGCTGACGTACAGGAACAGTACTTGCCAAGCGTTTTAGCGCAAGAGTCCGTCACTCCATACATTGCAATGCTGAATGGAGAGCCGATTGGGTATGCCCAGTCGTACGTTGCTCTTGGAAGCGGGGACGGATGGTGGGAAGAAGAAACCGATCCAGGAGTACGCGGAATAGACCAGTTACTGGCGAATGCATCACAACTGGGCAAAGGCTTGGGAACCAAGCTGGTTCGAGCTCTGGTTGAGTTGCTGTTCAATGATCCCGAGGTCACCAAGATCCAAACGGACCCGTCGCCGAGCAACTTGCGAGCGATCCGATGCTACGAGAAAGCGGGGTTTGAGAGGCAAGGTACCGTAACCACCCCAGATGGTCCAGCCGTGTACATGGTTCAAACACGCCAGGCATTCGAGCGAACACGCAGTGATGCCTAA

>AF453998 Serratia marcescens integron 2 aminoglycoside (2') adenylyltransferase (aadB), maturase-related protein (smtr), fused aminoglycoside (3'') adenylyltransferase-aminoglycoside (6') acetyltransferase ANT(3'')-Ih-AAC(6')-IId (ant(3'')-Ih-aac(6')-IId), and hypothetical protein (orfO) genes, complete cds; bla(oxa10) pseudogene, complete sequence; and IS1, complete sequence.

GTGACCAACAGC---AACGATTCCGTCACACTGCGCCTCATGACTGAGCATGACCTTGCGATGCTCTATGAGTGGCTAAATCGATCTCATATCGTCGAGTGGTGGGGCGGAGAAGAAGCACGCCCGACACTTGCTGACGTACAGGAACAGTACTTGCCAAGCGTTTTAGCGCAAGAGTCCGTCACTCCATACATTGCAATGCTGAATGGAGAGCCGATTGGGTATGCCCAGTCGTACGTTGCTCTTGGAAGCGGGGACGGATGGTGGGAAGAAGAAACCGATCCAGGAGTACGCGGAATAGACCAGTCACTGGCGAATGCATCACAACTGGGCAAAGGCTTGGGAACCAAGCTGGTTCGAGCTCTGGTTGAGTTGCTGTTCAATGATCCCGAGGTCACCAAGATCCAAACGGACCCGTCGCCGAGCAACTTGCGAGCGATCCGATGCTACGAGAAAGCGGGGTTTGAGAAGCAAGGTACCGTAACCACCCCAGATGGTCCAGCCGTGTACATGGTTCAAACACGCCAGGCATTCGAGCGAACACGCAGTGATGCCTAA

>AY740681 Aeromonas punctata class 1 integron In39, partial sequence.

GTGACCAACAGC---ACCGATTCCGTCACACTGCGCCTCATGACTGAGCATGACCTTGCGATGCTCTATGAGTGGCTAAATCGATCTCATATCGTCGAGTGGTGGGGCGGAGAAGAAGCACGCCCGACACTTGCTGACGTACAGGAACAGTACTTGCCAAGCGTTTTAGCGCAAGAGTCCGTCACTCCATACATTGCAATGCTGAATGGAGAGCCGATTGGGTATGCCCAGTCGTACGTTGCTCTTGGAAGCGGGGACGGATGGTGGGAAGAAGAAACCGATCCAGGAGTACGCGGAATAGACCAGTCACTGGCGAATGCATCACAACTGGGCAAAGGCTTGGGAACCAAGCTGGTTCGAGCTCTGGTTGAGTTGCTGTTCAATGATCCCGAGGTCACCAAGATCCAAACGGACCCGTCGCCGAGCAACTTGCGAGCGATCCGATGCTACGAGAAAGCGGGGTTTGAGAGGCAAGGTACCGTAACCACCCCAGATGGTCCAGCCGTGTACATGGTTCAAACACGCCAGGCATTCGAGCGAACACGCAGTGATGCCTAA

>AB070224 Serratia marcescens tniC, intI3, blaIMP, aac6'-Ib, parA, blaTEM genes, partial and complete cds.

GTGACCAACAGC---ACCGATTCCGTCACACTGCGCCTCATGACTGAGCATGACCTTGCGATGCTCTATGAGTGGCTAAATCGATCTCATATCGTCGAGTGGTGGGGCGGAGAAGAAGCACGCCCGACACTTGCTGACGTACAGGAACAGTACTTGCCAAGCGTTTTAGCGCAAGAGTCCGTCACTCCATACATTGCAATGCTGAATGGAGAGCCGATTGGGTATGCCCAGTCGTACGTTGCTCTTGGAAGCGGGGACGGATGGTGGGAAGAAGAAACCGATCCAGGAGTACGCGGAATAGACCAGTCACTGGCGAATGCATCACAACTGGGCAAAGGCTTGGGAACCAAGCTGGTTCGAGCTCTGGTTGAGTTGCTGTTCAATGATCCCGAGGTCACCAAGATCCAAACGGACCCGTCGCCGAGCAACTTGCGAGCGATCCGATGCTACGAGAAAGCGGGGTTTGAGAGGCAAGGTACCGTAACCACCCCAGATGGTCCAGCCGTGTACATGGTTCAAACACGCCAGGCATTCGAGCGAACACGCAGTGATGCCTAA

>PLPDHOS Escherichia coli plasmid pSa, partial sequence.

GTGACCAACAGC---ACCGATTCCGTCACACTGCGCCTCATGACTGAGCATGACCTTGCGATGCTCTATGAGTGGCTAAATCGATCTCATATCGTCGAGTGGTGGGGCGGAGAAGAAGCACGCCCGACACTTGCTGACGTACAGGAACAGTACTTGCCAAGCGTTTTAGCGCAAGAGTCCGTCACTCCATACATTGCAATGCTGAATGGAGAGCCGATTGGGTATGCCCAGTCGTACGTTGCTCTTGGAAGCGGGGACGGATGGTGGGAAGAAGAAACCGATCCAGGAGTACGCGGAATAGACCAGTCACTGGCGAATGCATCACAACTGGGCAAAGGCTTGGGAACCAAGCTGGTTCGAGCTCTGGTTGAGTTGCTGTTCAATGATCCCGAGGTCACCAAGATCCAAACGGACCCGTCGCCGAGCAACTTGCGAGCGATCCGATGCTACGAGAAAGCGGGGTTTGAGAGGCAAGGTACCGTAACCACCCCAGATGGTCCAGCCGTGTACATGGTTCAAACACGCCAGGCATTCGAGCGAACACGCAGTGATGCCTAA

>DQ393781 Pseudomonas aeruginosa strain N2-15 class I integron, partial sequence.

GTGACCAACAGC---ACCGATTCCGTCACACTGCGCCTCATGACTGAGCATGACCTTGCGATGCTCTATGAGTGGCTAAATCGATCTCATATCGTCGAGTGGTGGGGCGGAGAAGAAGCACGCCCGACACTTGCTGACGTACAGGAACAGTACTTGCCAAGCGTTTTAGCGCAAGAGTCCGTCACTCCATACATTGCAATGCTGAATGGAGAGCCGATTGGGTATGCCCAGTCGTACGTTGCTCTTGGAAGCGGGGACGGATGGTGGGAAGAAGAAACCGATCCAGGAGTACGCGGAATAGACCAGTCACTGGCGAATGCATCACAACTGGGCAAAGGCTTGGGAACCAAGCTGGTTCGAGCTCTGGTTGAGTTGCTGTTCAATGATCCCGAGGTCACCAAGATCCAAACGGACCCGTCGCCGAGCAACTTGCGAGCGATCCGATGCTACGAGAAAGCGGGGTTTGAGAGGCAAGGTACCGTAACCACCCCAGATGGTCCAGCCGTGTACATGGTTCAAACACGCCAGGCATTCGAGCGAACACGCAGTGATGCCTAA

>AF416297 Serratia marcescens class 3 integron, partial sequence.

GTGACCAACAGC---ACCGATTCCGTCACACTGCGCCTCATGACTGAGCATGACCTTGCGATGCTCTATGAGTGGCTAAATCGATCTCATATCGTCGAGTGGTGGGGCGGAGAAGAAGCACGCCCGACACTTGCTGACGTACAGGAACAGTACTTGCCAAGCGTTTTAGCGCAAGAGTCCGTCACTCCATACATTGCAATGCTGAATGGAGAGCCGATTGGGTATGCCCAGTCGTACGTTGCTCTTGGAAGCGGGGACGGATGGTGGGAAGAAGAAACCGATCCAGGAGTACGCGGAATAGACCAGTCACTGGCGAATGCATCACAACTGGGCAAAGGCTTGGGAACCAAGCTGGTTCGAGCTCTGGTTGAGTTGCTGTTCAATGATCCCGAGGTCACCAAGATCCAAACGGACCCGTCGCCGAGCAACTTGCGAGCGATCCGATGCTACGAGAAAGCGGGGTTTGAGAGGCAAGGTACCGTAACCACCCCAGATGGTCCAGCCGTGTACATGGTTCAAACACGCCAGGCATTCGAGCGAACACGCAGTGATGCCTAA

>AF322577 Klebsiella pneumoniae plasmid pEKP0787-1 integron In73 DNA integrase form delta 1 (intI1delta1), metallo-beta-lactamase IMP-8 (blaIMP-8), aminoglycoside 6'-N-acetyltransferase (aac6'-Ib), chloramphenicol acetyltransferase (catB4), and ethidium bromide resistance protein qacE form delta1 (qacEdelta1) genes, complete cds; and dihydropteroate synthase Sul1 (sul1) gene, partial cds.

GTGACCAACAGC---ACCGATTCCGTCACACTGCGCCTCATGACTGAGCATGACCTTGCGATGCTCTATGAGTGGCTAAATCGATCTCATATCGTCGAGTGGTGGGGCGGAGAAGAAGCACGCCCGACACTTGCTGACGTACAGGAACAGTACTTGCCAAGCGTTTTAGCGCAAGAGTCCGTCACTCCATACATTGCAATGCTGAATGGAGAGCCGATTGGGTATGCCCAGTCGTACGTTGCTCTTGGAAGCGGGGACGGATGGTGGGAAGAAGAAACCGATCCAGGAGTACGCGGAATAGACCAGTCACTGGCGAATGCATCACAACTGGGCAAAGGCTTGGGAACCAAGCTGGTTCGAGCTCTGGTTGAGTTGCTGTTCAATGATCCCGAGGTCACCAAGATCCAAACGGACCCGTCGCCGAGCAACTTGCGAGCGATCCGATGCTACGAGAAAGCGGGGTTTGAGAGGCAAGGTACCGTAACCACCCCAGATGGTCCAGCCGTGTACATGGTTCAAACACGCCAGGCATTCGAGCGAACACGCAGTGATGCCTAA

>AY220520 Salmonella enterica subsp. enterica class 1 integron DNA integrase IntI1 (intI1) gene, partial cds; AACA4 (aacA6'-Ib) and blaOXA-1-like genes, complete cds; QacEdelta1 (qacEdelta1) gene, partial cds; and unknown gene.

GTGACCAACAGC---ACCGATTCCGTCACACTGCGCCTCATGACTGAGCATGACCTTGCGATGCTCTATGAGTGGCTAAATCGATCTCATATCGTCGAGTGGTGGGGCGGAGAAGAAGCACGCCCGACACTTGCTGACGTACAGGAACAGTACTTGCCAAGCGTTTTAGCGCAAGAGTCCGTCACTCCATACATTGCAATGCTGAATGGAGAGCCGATTGGGTATGCCCAGTCGTACGTTGCTCTTGGAAGCGGGGACGGATGGTGGGAAGAAGAAACCGATCCAGGAGTACGCGGAATAGACCAGTCACTGGCGAATGCATCACAACTGGGCAAAGGCTTGGGAACCAAGCTGGTTCGAGCTCTGGTTGAGTTGCTGTTCAATGATCCCGAGGTCACCAAGATCCAAACGGACCCGTCGCCGAGCAACTTGCGAGCGATCCGATGCTACGAGAAAGCGGGGTTTGAGAGGCAAGGTACCGTAACCACCCCAGATGGTCCAGCCGTGTACATGGTTCAAACACGCCAGGCATTCGAGCGAACACGCAGTGATGCCTAA

>AY348316 Salmonella enterica subsp. enterica serovar Typhi class I integron DNA integrase (intI1) gene, partial cds; aminoglycoside acetyltransferase (aacA6'-Ib) and oxacillinase (blaOXA-1-like) genes, complete cds; quaternary ammonium compound (qacEdelta1) gene, partial cds; and unknown gene.

GTGACCAACAGC---ACCGATTCCGTCACACTGCGCCTCATGACTGAGCATGACCTTGCGATGCTCTATGAGTGGCTAAATCGATCTCATATCGTCGAGTGGTGGGGCGGAGAAGAAGCACGCCCGACACTTGCTGACGTACAGGAACAGTACTTGCCAAGCGTTTTAGCGCAAGAGTCCGTCACTCCATACATTGCAATGCTGAATGGAGAGCCGATTGGGTATGCCCAGTCGTACGTTGCTCTTGGAAGCGGGGACGGATGGTGGGAAGAAGAAACCGATCCAGGAGTACGCGGAATAGACCAGTCACTGGCGAATGCATCACAACTGGGCAAAGGCTTGGGAACCAAGCTGGTTCGAGCTCTGGTTGAGTTGCTGTTCAATGATCCCGAGGTCACCAAGATCCAAACGGACCCGTCGCCGAGCAACTTGCGAGCGATCCGATGCTACGAGAAAGCGGGGTTTGAGAGGCAAGGTACCGTAACCACCCCAGATGGTCCAGCCGTGTACATGGTTCAAACACGCCAGGCATTCGAGCGAACACGCAGTGATGCCTAA

>AY623046 Cloning vector pUCD615 aminoglycoside 6'-N-acetyltransferase (aac(6')-Ib) gene, complete cds.

GTGACCAACAGC---ACCGATTCCGTCACACTGCGCCTCATGACTGAGCATGACCTTGCGATGCTCTATGAGTGGCTAAATCGATCTCATATCGTCGAGTGGTGGGGCGGAGAAGAAGCACGCCCGACACTTGCTGACGTACAGGAACAGTACTTGCCAAGCGTTTTAGCGCAAGAGTCCGTCACTCCATACATTGCAATGCTGAATGGAGAGCCGATTGGGTATGCCCAGTCGTACGTTGCTCTTGGAAGCGGGGACGGATGGTGGGAAGAAGAAACCGATCCAGGAGTACGCGGAATAGACCAGTCACTGGCGAATGCATCACAACTGGGCAAAGGCTTGGGAACCAAGCTGGTTCGAGCTCTGGTTGAGTTGCTGTTCAATGATCCCGAGGTCACCAAGATCCAAACGGACCCGTCGCCGAGCAACTTGCGAGCGATCCGATGCTACGAGAAAGCGGGGTTTGAGAGGCAAGGTACCGTAACCACCCCAGATGGTCCAGCCGTGTACATGGTTCAAACACGCCAGGCATTCGAGCGAACACGCAGTGATGCCTAA

>AY907717 Pseudomonas putida isolate YMC 98/2/665 class I integron DNA integrase (intI1) gene, partial cds, aminoglycoside 6'-acetyltransferase type I (aacA6'-I), metallo-beta-lactamase VIM-2 (blaVIM-2), aminoglycoside 6'-acetyltransferase type Ib (aacA6'-Ib), oxacillinase (blaOXA-2), and quaternary ammonium compound-resistance protein (qac) genes, complete cds, quaternary ammonium compound-resistance protein (qacEdelta1) gene, partial cds, and unknown genes.

GTGACCAACAGC---ACCGATTCCGTCACACTGCGCCTCATGACTGAGCATGACCTTGCGATGCTCTATGAGTGGCTAAATCGATCTCATATCGTCGAGTGGTGGGGCGGAGAAGAAGCACGCCCGACACTTGCTGACGTACAGGAACAGTACTTGCCAAGCGTTTTAGCGCAAGAGTCCGTCACTCCATACATTGCAATGCTGAATGGAGAGCCGATTGGGTATGCCCAGTCGTACGTTGCTCTTGGAAGCGGGGACGGATGGTGGGAAGAAGAAACCGATCCAGGAGTACGCGGAATAGACCAGTCACTGGCGAATGCATCACAACTGGGCAAAGGCTTGGGAACCAAGCTGGTTCGAGCTCTGGTTGAGTTGCTGTTCAATGATCCCGAGGTCACCAAGATCCAAACGGACCCGTCGCCGAGCAACTTGCGAGCGATCCGATGCTACGAGAAAGCGGGGTTTGAGAGGCAAGGTACCGTAACCACCCCAGATGGTCCAGCCGTGTACATGGTTCAAACACGCCAGGCATTCGAGCGAACACGCAGTGATGCCTAA

>AY907717 Pseudomonas putida isolate YMC 98/2/665 class I integron DNA integrase (intI1) gene, partial cds, aminoglycoside 6'-acetyltransferase type I (aacA6'-I), metallo-beta-lactamase VIM-2 (blaVIM-2), aminoglycoside 6'-acetyltransferase type Ib (aacA6'-Ib), oxacillinase (blaOXA-2), and quaternary ammonium compound-resistance protein (qac) genes, complete cds, quaternary ammonium compound-resistance protein (qacEdelta1) gene, partial cds, and unknown genes.

GTGACCAACAGC---ACCGATTCCGTCACACTGCGCCTCATGACTGAGCATGACCTTGCGATGCTCTATGAGTGGCTAAATCGATCTCATATCGTCGAGTGGTGGGGCGGAGAAGAAGCACGCCCGACACTTGCTGACGTACAGGAACAGTACTTGCCAAGCGTTTTAGCGCAAGAGTCCGTCACTCCATACATTGCAATGCTGAATGGAGAGCCGATTGGGTATGCCCAGTCGTACGTTGCTCTTGGAAGCGGGGACGGATGGTGGGAAGAAGAAACCGATCCAGGAGTACGCGGAATAGACCAGTCACTGGCGAATGCATCACAACTGGGCAAAGGCTTGGGAACCAAGCTGGTTCGAGCTCTGGTTGAGTTGCTGTTCAATGATCCCGAGGTCACCAAGATCCAAACGGACCCGTCGCCGAGCAACTTGCGAGCGATCCGATGCTACGAGAAAGCGGGGTTTGAGAGGCAAGGTACCGTAACCACCCCAGATGGTCCAGCCGTGTACATGGTTCAAACACGCCAGGCATTCGAGCGAACACGCAGTGATGCCTAA

>DQ112222 Achromobacter denitrificans isolate YMC/HMC 233516 class I integron, partial sequence.

GTGACCAACAGC---ACCGATTCCGTCACACTGCGCCTCATGACTGAGCATGACCTTGCGATGCTCTATGAGTGGCTAAATCGATCTCATATCGTCGAGTGGTGGGGCGGAGAAGAAGCACGCCCGACACTTGCTGACGTACAGGAACAGTACTTGCCAAGCGTTTTAGCGCAAGAGTCCGTCACTCCATACATTGCAATGCTGAATGGAGAGCCGATTGGGTATGCCCAGTCGTACGTTGCTCTTGGAAGCGGGGACGGATGGTGGGAAGAAGAAACCGATCCAGGAGTACGCGGAATAGACCAGTCACTGGCGAATGCATCACAACTGGGCAAAGGCTTGGGAACCAAGCTGGTTCGAGCTCTGGTTGAGTTGCTGTTCAATGATCCCGAGGTCACCAAGATCCAAACGGACCCGTCGCCGAGCAACTTGCGAGCGATCCGATGCTACGAGAAAGCGGGGTTTGAGAGGCAAGGTACCGTAACCACCCCAGATGGTCCAGCCGTGTACATGGTTCAAACACGCCAGGCATTCGAGCGAACACGCAGTGATGCCTAA

>DQ112222 Achromobacter denitrificans isolate YMC/HMC 233516 class I integron, partial sequence.

GTGACCAACAGC---ACCGATTCCGTCACACTGCGCCTCATGACTGAGCATGACCTTGCGATGCTCTATGAGTGGCTAAATCGATCTCATATCGTCGAGTGGTGGGGCGGAGAAGAAGCACGCCCGACACTTGCTGACGTACAGGAACAGTACTTGCCAAGCGTTTTAGCGCAAGAGTCCGTCACTCCATACATTGCAATGCTGAATGGAGAGCCGATTGGGTATGCCCAGTCGTACGTTGCTCTTGGAAGCGGGGACGGATGGTGGGAAGAAGAAACCGATCCAGGAGTACGCGGAATAGACCAGTCACTGGCGAATGCATCACAACTGGGCAAAGGCTTGGGAACCAAGCTGGTTCGAGCTCTGGTTGAGTTGCTGTTCAATGATCCCGAGGTCACCAAGATCCAAACGGACCCGTCGCCGAGCAACTTGCGAGCGATCCGATGCTACGAGAAAGCGGGGTTTGAGAGGCAAGGTACCGTAACCACCCCAGATGGTCCAGCCGTGTACATGGTTCAAACACGCCAGGCATTCGAGCGAACACGCAGTGATGCCTAA

>DQ287356 Pseudomonas aeruginosa class I integron metallo-beta-lactamase VIM-2 (blaVIM-2), quaternary ammonium compound-resistance protein (qac), aminoglycoside (6') N-acetyltransferase (aacA4), chloramphenicol acetyltransferase (catB3), oxacillinase (blaOXA-30), and 3'-aminoglycoside adenyltransferase (aadA1) genes, complete cds.

GTGACCAACAGC---ACCGATTCCGTCACACTGCGCCTCATGACTGAGCATGACCTTGCGATGCTCTATGAGTGGCTAAATCGATCTCATATCGTCGAGTGGTGGGGCGGAGAAGAAGCACGCCCGACACTTGCTGACGTACAGGAACAGTACTTGCCAAGCGTTTTAGCGCAAGAGTCCGTCACTCCATACATTGCAATGCTGAATGGAGAGCCGATTGGGTATGCCCAGTCGTACGTTGCTCTTGGAAGCGGGGACGGATGGTGGGAAGAAGAAACCGATCCAGGAGTACGCGGAATAGACCAGTCACTGGCGAATGCATCACAACTGGGCAAAGGCTTGGGAACCAAGCTGGTTCGAGCTCTGGTTGAGTTGCTGTTCAATGATCCCGAGGTCACCAAGATCCAAACGGACCCGTCGCCGAGCAACTTGCGAGCGATCCGATGCTACGAGAAAGCGGGGTTTGAGAGGCAAGGTACCGTAACCACCCCAGATGGTCCAGCCGTGTACATGGTTCAAACACGCCAGGCATTCGAGCGAACACGCAGTGATGCCTAA

>DQ342344 Pseudomonas aeruginosa class 1 integron integrase IntI1 (intI1) gene, partial cds, VIM-3 metallo-beta-lactamase (blaVIM-3) and aminoglycoside (6')-N-acetyltransferase (aacA4) genes, complete cds, and unknown gene.

GTGACCAACAGC---ACCGATTCCGTCACACTGCGCCTCATGACTGAGCATGACCTTGCGATGCTCTATGAGTGGCTAAATCGATCTCATATCGTCGAGTGGTGGGGCGGAGAAGAAGCACGCCCGACACTTGCTGACGTACAGGAACAGTACTTGCCAAGCGTTTTAGCGCAAGAGTCCGTCACTCCATACATTGCAATGCTGAATGGAGAGCCGATTGGGTATGCCCAGTCGTACGTTGCTCTTGGAAGCGGGGACGGATGGTGGGAAGAAGAAACCGATCCAGGAGTACGCGGAATAGACCAGTCACTGGCGAATGCATCACAACTGGGCAAAGGCTTGGGAACCAAGCTGGTTCGAGCTCTGGTTGAGTTGCTGTTCAATGATCCCGAGGTCACCAAGATCCAAACGGACCCGTCGCCGAGCAACTTGCGAGCGATCCGATGCTACGAGAAAGCGGGGTTTGAGAGGCAAGGTACCGTAACCACCCCAGATGGTCCAGCCGTGTACATGGTTCAAACACGCCAGGCATTCGAGCGAACACGCAGTGATGCCTAA

>DQ845788 Acinetobacter baumannii class I integron metallo-beta-lactamase IMP-8 (blaIMP-8) and aminoglycoside 6`-N-acetyltransferase (aac6`-Ib) genes, complete cds.

GTGACCAACAGC---ACCGATTCCGTCACACTGCGCCTCATGACTGAGCATGACCTTGCGATGCTCTATGAGTGGCTAAATCGATCTCATATCGTCGAGTGGTGGGGCGGAGAAGAAGCACGCCCGACACTTGCTGACGTACAGGAACAGTACTTGCCAAGCGTTTTAGCGCAAGAGTCCGTCACTCCATACATTGCAATGCTGAATGGAGAGCCGATTGGGTATGCCCAGTCGTACGTTGCTCTTGGAAGCGGGGACGGATGGTGGGAAGAAGAAACCGATCCAGGAGTACGCGGAATAGACCAGTCACTGGCGAATGCATCACAACTGGGCAAAGGCTTGGGAACCAAGCTGGTTCGAGCTCTGGTTGAGTTGCTGTTCAATGATCCCGAGGTCACCAAGATCCAAACGGACCCGTCGCCGAGCAACTTGCGAGCGATCCGATGCTACGAGAAAGCGGGGTTTGAGAGGCAAGGTACCGTAACCACCCCAGATGGTCCAGCCGTGTACATGGTTCAAACACGCCAGGCATTCGAGCGAACACGCAGTGATGCCTAA

>EF027105 Achromobacter xylosoxidans strain MB-156 class I integron IntI1 (intI1) gene, partial cds, Imp-1 (blaIMP-1), AacA4 (aacA4), and AadA5 (aadA5) genes, complete cds, and QacEdelta1 (qacEdelta1) gene, partial cds.

GTGACCAACAGC---ACCGATTCCGTCACACTGCGCCTCATGACTGAGCATGACCTTGCGATGCTCTATGAGTGGCTAAATCGATCTCATATCGTCGAGTGGTGGGGCGGAGAAGAAGCACGCCCGACACTTGCTGACGTACAGGAACAGTACTTGCCAAGCGTTTTAGCGCAAGAGTCCGTCACTCCATACATTGCAATGCTGAATGGAGAGCCGATTGGGTATGCCCAGTCGTACGTTGCTCTTGGAAGCGGGGACGGATGGTGGGAAGAAGAAACCGATCCAGGAGTACGCGGAATAGACCAGTCACTGGCGAATGCATCACAACTGGGCAAAGGCTTGGGAACCAAGCTGGTTCGAGCTCTGGTTGAGTTGCTGTTCAATGATCCCGAGGTCACCAAGATCCAAACGGACCCGTCGCCGAGCAACTTGCGAGCGATCCGATGCTACGAGAAAGCGGGGTTTGAGAGGCAAGGTACCGTAACCACCCCAGATGGTCCAGCCGTGTACATGGTTCAAACACGCCAGGCATTCGAGCGAACACGCAGTGATGCCTAA

>EF206349 Pseudomonas aeruginosa strain YMC 05/5/U1361 class I integron, partial sequence.

GTGACCAACAGC---ACCGATTCCGTCACACTGCGCCTCATGACTGAGCATGACCTTGCGATGCTCTATGAGTGGCTAAATCGATCTCATATCGTCGAGTGGTGGGGCGGAGAAGAAGCACGCCCGACACTTGCTGACGTACAGGAACAGTACTTGCCAAGCGTTTTAGCGCAAGAGTCCGTCACTCCATACATTGCAATGCTGAATGGAGAGCCGATTGGGTATGCCCAGTCGTACGTTGCTCTTGGAAGCGGGGACGGATGGTGGGAAGAAGAAACCGATCCAGGAGTACGCGGAATAGACCAGTCACTGGCGAATGCATCACAACTGGGCAAAGGCTTGGGAACCAAGCTGGTTCGAGCTCTGGTTGAGTTGCTGTTCAATGATCCCGAGGTCACCAAGATCCAAACGGACCCGTCGCCGAGCAACTTGCGAGCGATCCGATGCTACGAGAAAGCGGGGTTTGAGAGGCAAGGTACCGTAACCACCCCAGATGGTCCAGCCGTGTACATGGTTCAAACACGCCAGGCATTCGAGCGAACACGCAGTGATGCCTAA

>EF207719 Pseudomonas aeruginosa strain YMC 05/5/2030 class 1 integron DNA integrase (intI1) gene, partial cds, metallo-beta-lactamase VIM-2 (blaVIM-2), aminoglycoside 6'-N-acetyltransferase (aacA(6')-Ib), unknown orf/DNA integrase fusion protein, and erythromycin esterase type I (ereA) genes, complete cds, and quaternary ammonium compound-resistance protein (qacEdelta1) gene, partial cds.

GTGACCAACAGC---ACCGATTCCGTCACACTGCGCCTCATGACTGAGCATGACCTTGCGATGCTCTATGAGTGGCTAAATCGATCTCATATCGTCGAGTGGTGGGGCGGAGAAGAAGCACGCCCGACACTTGCTGACGTACAGGAACAGTACTTGCCAAGCGTTTTAGCGCAAGAGTCCGTCACTCCATACATTGCAATGCTGAATGGAGAGCCGATTGGGTATGCCCAGTCGTACGTTGCTCTTGGAAGCGGGGACGGATGGTGGGAAGAAGAAACCGATCCAGGAGTACGCGGAATAGACCAGTCACTGGCGAATGCATCACAACTGGGCAAAGGCTTGGGAACCAAGCTGGTTCGAGCTCTGGTTGAGTTGCTGTTCAATGATCCCGAGGTCACCAAGATCCAAACGGACCCGTCGCCGAGCAACTTGCGAGCGATCCGATGCTACGAGAAAGCGGGGTTTGAGAGGCAAGGTACCGTAACCACCCCAGATGGTCCAGCCGTGTACATGGTTCAAACACGCCAGGCATTCGAGCGAACACGCAGTGATGCCTAA

>EF184216 Pseudomonas aeruginosa isolate 4677 class I integron In95, partial sequence.

GTGACCAACAGC---ACCGATTCCGTCACACTGCGCCTCATGACTGAGCATGACCTTGCGATGCTCTATGAGTGGCTAAATCGATCTCATATCGTCGAGTGGTGGGGCGGAGAAGAAGCACGCCCGACACTTGCTGACGTACAGGAACAGTACTTGCCAAGCGTTTTAGCGCAAGAGTCCGTCACTCCATACATTGCAATGCTGAATGGAGAGCCGATTGGGTATGCCCAGTCGTACGTTGCTCTTGGAAGCGGGGACGGATGGTGGGAAGAAGAAACCGATCCAGGAGTACGCGGAATAGACCAGTCACTGGCGAATGCATCACAACTGGGCAAAGGCTTGGGAACCAAGCTGGTTCGAGCTCTGGTTGAGTTGCTGTTCAATGATCCCGAGGTCACCAAGATCCAAACGGACCCGTCGCCGAGCAACTTGCGAGCGATCCGATGCTACGAGAAAGCGGGGTTTGAGAGGCAAGGTACCGTAACCACCCCAGATGGTCCAGCCGTGTACATGGTTCAAACACGCCAGGCATTCGAGCGAACACGCAGTGATGCCTAA

>DQ310703 Vibrio cholerae plasmid pAS1 insertion sequence IS4321R and transposon Tn1696, complete sequence; and class I integron InVC117, partial sequence.

GTGACCAACAGC---AACGATTCCGTCACACTGCGCCTCATGACTGAGCATGACCTTGCGATGCTCTATGAGTGGCTAAATCGATCTCATATCGTCGAGTGGTGGGGCGGAGAAGAAGCACGCCCGACACTTGCTGACGTACAGGAACAGTACTTGCCAAGCGTTTTAGCGCAAGAGTCCGTCACTCCATACATTGCAATGCTGAATGGAGAGCCGATTGGGTATGCCCAGTCGTACGTTGCTCTTGGAAGCGGGGACGGATGGTGGGAAGAAGAAACCGATCCAGGAGTACGCGGAATAGACCAGTTACTGGCGAATGCATCACAACTGGGCAAAGGCTTGGGAACCAAGCTGGTCCGAGCTCTGGTTGAGTTGCTGTTCAATGATCCCGAGGTCACCAAGATCCAAACGGACCCGTCGCCGAGCAACTTGCGAGCGATCCGATGCTACGAGAAAGCGGGGTTTGAGAGGCAAGGTACCGTAACCACCCCAGATGGTCCAGCCGTGTACATGGTTCAAACACGCCAGGCATTCGAGCGAACACGCAGTGATGCCTAA

>AJ311891 Salmonella enterica subsp. enterica serovar Infantis plasmid pS21 class I sulI-type integron (InS21).

GTGACCAACAGC---AACGATTCCGTCACACTGCGCCTCATGACTGAGCATGACCTTGCGATGCTCTATGAGTGGCTAAATCGATCTCATATCGTCGAGTGGTGGGGCGGAGAAGAAGCACGCCCGACACTTGCTGACGTACAGGAACAGTACTTGCCAAGCGTTTTAGCGCAAGAGTCCGTCACTCCATACATTGCAATGCTGAATGGAGAGCCGATTGGGTATGCCCAGTCGTACGTTGCTCTTGGAAGCGGGGACGGATGGTGGGAAGAAGAAACCGATCCAGGAGTACGCGGAATAGACCAGTTACTGGCGAATGCATCACAACTGGGCAAAGGCTTGGGAACCAAGCTGGTCCGAGCTCTGGTTGAGTTGCTGTTCAATGATCCCGAGGTCACCAAGATCCAAACGGACCCGTCGCCGAGCAACTTGCGAGCGATCCGATGCTACGAGAAAGCGGGGTTTGAGAGGCAAGGTACCGTAACCACCCCAGATGGTCCAGCCGTGTACATGGTTCAAACACGCCAGGCATTCGAGCGAACACGCAGTGATGCCTAA

>AJ621187 Morganella morganii subsp. morganii class 1 integron (In116).

GTGACCAACAGC---AACGATTCCGTCACACTGCGCCTCATGACTGAGCATGACCTTGCGATGCTCTATGAGTGGCTAAATCGATCTCATATCGTCGAGTGGTGGGGCGGAGAAGAAGCACGCCCGACACTTGCTGACGTACAGGAACAGTACTTGCCAAGCGTTTTAGCGCAAGAGTCCGTCACTCCATACATTGCAATGCTGAATGGAGAGCCGATTGGGTATGCCCAGTCGTACGTTGCTCTTGGAAGCGGGGACGGATGGTGGGAAGAAGAAACCGATCCAGGAGTACGCGGAATAGACCAGTTACTGGCGAATGCATCACAACTGGGCAAAGGCTTGGGAACCAAGCTGGTCCGAGCTCTGGTTGAGTTGCTGTTCAATGATCCCGAGGTCACCAAGATCCAAACGGACCCGTCGCCGAGCAACTTGCGAGCGATCCGATGCTACGAGAAAGCGGGGTTTGAGAGGCAAGGTACCGTAACCACCCCAGATGGTCCAGCCGTGTACATGGTTCAAACACGCCAGGCATTCGAGCGAACACGCAGTGATGCCTAA

>AM040449 Klebsiella pneumoniae partial integron InK13.

GTGACCAACAGC---AACGATTCCGTCACACTGCGCCTCATGACTGAGCATGACCTTGCGATGCTCTATGAGTGGCTAAATCGATCTCATATCGTCGAGTGGTGGGGCGGAGAAGAAGCACGCCCGACACTTGCTGACGTACAGGAACAGTACTTGCCAAGCGTTTTAGCGCAAGAGTCCGTCACTCCATACATTGCAATGCTGAATGGAGAGCCGATTGGGTATGCCCAGTCGTACGTTGCTCTTGGAAGCGGGGACGGATGGTGGGAAGAAGAAACCGATCCAGGAGTACGCGGAATAGACCAGTTACTGGCGAATGCATCACAACTGGGCAAAGGCTTGGGAACCAAGCTGGTCCGAGCTCTGGTTGAGTTGCTGTTCAATGATCCCGAGGTCACCAAGATCCAAACGGACCCGTCGCCGAGCAACTTGCGAGCGATCCGATGCTACGAGAAAGCGGGGTTTGAGAGGCAAGGTACCGTAACCACCCCAGATGGTCCAGCCGTGTACATGGTTCAAACACGCCAGGCATTCGAGCGAACACGCAGTGATGCCTAA

>AM040709 Escherichia coli intI1 gene (partial), aac(6')1b gene, blaOXA-2 gene, ORFD, qacEdelta1 gene, sul1 gene, ORF513, blaCTX-M-2 gene, ORF3 (partial), qacEdelta1 gene and sul1 gene (partial).

GTGACCAACAGC---AACGATTCCGTCACACTGCGCCTCATGACTGAGCATGACCTTGCGATGCTCTATGAGTGGCTAAATCGATCTCATATCGTCGAGTGGTGGGGCGGAGAAGAAGCACGCCCGACACTTGCTGACGTACAGGAACAGTACTTGCCAAGCGTTTTAGCGCAAGAGTCCGTCACTCCATACATTGCAATGCTGAATGGAGAGCCGATTGGGTATGCCCAGTCGTACGTTGCTCTTGGAAGCGGGGACGGATGGTGGGAAGAAGAAACCGATCCAGGAGTACGCGGAATAGACCAGTTACTGGCGAATGCATCACAACTGGGCAAAGGCTTGGGAACCAAGCTGGTCCGAGCTCTGGTTGAGTTGCTGTTCAATGATCCCGAGGTCACCAAGATCCAAACGGACCCGTCGCCGAGCAACTTGCGAGCGATCCGATGCTACGAGAAAGCGGGGTTTGAGAGGCAAGGTACCGTAACCACCCCAGATGGTCCAGCCGTGTACATGGTTCAAACACGCCAGGCATTCGAGCGAACACGCAGTGATGCCTAA

>DQ139276 Pseudomonas aeruginosa isolate PS109 class 1 integron, partial sequence.

GTGACCAACAGC---AACGATTCCGTCACACTGCGCCTCATGACTGAGCATGACCTTGCGATGCTCTATGAGTGGCTAAATCGATCTCATATCGTCGAGTGGTGGGGCGGAGAAGAAGCACGCCCGACACTTGCTGACGTACAGGAACAGTACTTGCCAAGCGTTTTAGCGCAAGAGTCCGTCACTCCATACATTGCAATGCTGAATGGAGAGCCGATTGGGTATGCCCAGTCGTACGTTGCTCTTGGAAGCGGGGACGGATGGTGGGAAGAAGAAACCGATCCAGGAGTACGCGGAATAGACCAGTTACTGGCGAATGCATCACAACTGGGCAAAGGCTTGGGAACCAAGCTGGTCCGAGCTCTGGTTGAGTTGCTGTTCAATGATCCCGAGGTCACCAAGATCCAAACGGACCCGTCGCCGAGCAACTTGCGAGCGATCCGATGCTACGAGAAAGCGGGGTTTGAGAGGCAAGGTACCGTAACCACCCCAGATGGTCCAGCCGTGTACATGGTTCAAACACGCCAGGCATTCGAGCGAACACGCAGTGATGCCTAA

>AF318077 Pseudomonas aeruginosa integron InAB1 aminoglycoside 6'-N-acetyltransferase (aacC4), metallo-beta-lactamase (blaIMP-7), and aminoglycoside 3-N-acetyltransferase (aacC1) genes, complete cds.

GTGACCAACAGC---AACGATTCCGTCACACTGCGCCTCATGACTGAGCATGACCTTGCGATGCTCTATGAGTGGCTAAATCGATCTCATATCGTCGAGTGGTGGGGCGGAGAAGAAGCACGCCCGACACTTGCTGACGTACAGGAACAGTACTTGCCAAGCGTTTTAGCGCAAGAGTCCGTCACTCCATACATTGCAATGCTGAATGGAGAGCCGATTGGGTATGCCCAGTCGTACGTTGCTCTTGGAAGCGGGGACGGATGGTGGGAAGAAGAAACCGATCCAGGAGTACGCGGAATAGACCAGTTACTGGCGAATGCGTCACAACTGGGCAAAGGCTTGGGAACCAAGCTGGTTCGAGCTCTGGTTGAGTTGCTGTTCAATGATCCCGAGGTCACCAAGATCCAAACGGACCCGTCGCCGAGCAACTTGCGAGCGATCCGATGCTACGAGAAAGCGGGGTTTGAGAGGCAAGGCACCGTAACCACCCCAGATGGTCCAGCCGTGTACATGGTTCAAACACGCCAGGCATTCGAGCGAACACGCAGTGATGCCTAA

>AY007784 Acinetobacter baumannii transposon IntAb74 aminoglycoside 6'-N-acetyltransferase Ib (aacA4) and oxacillinase (oxa-37) genes, complete cds, and unknown gene.

GTGACCAACAGC---AACGATTCCGTCACACTGCGCCTCATGACTGAGCATGACCTTGCGATGCTCTATGAGTGGCTAAATCGATCTCATATCGTCGAGTGGTGGGGCGGAGAAGAAGCACGCCCGACACTTGCTGACGTACAGGAACAGTACTTGCCAAGCGTTTTAGCGCAAGAGTCCGTCACTCCATACATTGCAATGCTGAATGGAGAGCCGATTGGGTATGCCCAGTCGTACGTTGCTCTTGGAAGCGGGGACGGATGGTGGGAAGAAGAAACCGATCCAGGAGTACGCGGAATAGACCAGTTACTGGCGAATGCATCACAACTGGGCAAAGGCTTGGGAACCAAGCTCGTTCGAGCTCTGGTTGAGTTGCTGTTCAATGATCCCGAGGTCACCAAGATCCAAACGGACCCGTCGCCGAGCAACTTGCGAGCGATCCGATGCTACGAGAAAGCGGGGTTTGAGAGGCAAGGTACCGTAACCACCCCAGATGGTCCAGCCGTGTACATGGTTCAAACACGCCAGGCATTCGAGCGAACACGCAGTGATGCCTAA

>AJ584652 Pseudomonas aeruginosa inti1 gene (partial), blaIMP-16 gene, aac(6')-30/aac(6')-Ib' fused gene, aacA4 gene, aadA1 gene, qacEdelta1 gene and sul1 gene (partial).

GTGACCAACAGC---AACGATTCCGTCACACTGCGCCTCATGACTGAGCATGACCTTGCGATGCTCTATGAGTGGCTAAATCGATCTCATATCGTCGAGTGGTGGGGCGGAGAAGAAGCACGCCCGACACTTGCTGACGTACAGGAACAGTACTTGCCAAGCGTTTTAGCGCAAGAGTCCGTCACTCCATACATTGCAATGCTGAATGGAGAGCCGATTGGGTATGCCCAGTCGTACGTTGCTCTTGGAAGCGGGGACGGATGGTGGGAAGAAGAAACCGATCCAGGAGTACGCGGAATAGACCAGTCACTGGCGAATGCATCACAACTGGGCAAAGGCTTGGGAACCAAGCTGGTTCGAGCTCTGGTTGAGTTGCTGTTCAATGATCCCGAGGTCACCAAGATCCAAACGGACCCGTCGCCGAGCAACTTGCGAGCGATCCGATGCTACGAGAAAGCGGGGTTTGAGAGGCAAGGTACCGTAACCACCCCAGATGGTCCAGCCGTGTACATGGTTCAAACACGCCAGGCATTCGAGCGAACACGCAGTGTTGCCTAA

>AY887109 Providencia rettgeri class 1 integron, complete sequence.

GTGACCAACAGC---AACGATTCCGTCACACTGCGCCTCATGACTGAGCATGACCTTGCGATGCTCTATGAGTGGCTAAATCGATCTCATATCGTCGAGTGGTGGGGCGGAGAAGAAGCACGCCCGACACTTGCTGACGTACAGGAACAGTACTTGCCAAGCGTTTTAGCGCAAGAGTCCGTCACTCCATACATTGCAATGCTGAATGGAGAGCCGATTGGGTATGCCCAGTCGTACGTTGCTCTTGGAAGCGGGGACGGATGGTGGGAAGAAGAAACCGATCCAGGAGTACGCGGAATAGACCAGTCACTGGCGAATGCATCACAACTGGGCAAAGGCTTGGGAACCAAGCTGGTTCGAGCTCTGGTTGAGTTGCTGTTCAATGATCCCGAGGTCACCAAGATCCAAACGGACCCGTCGCCGAGCAACTTGCGAGCGATCCGATGCTACGAGAAAGCGGGGTTTGAGAGGCAAGGTACCGTAACCACCCCAGATGGTCCAGCCGTGTACATGGTTCAAACACGCCAGGCATTCGAGCGAACACGCAGTGTTGCCTAA

>DQ153218 Klebsiella pneumoniae isolate YMC 03/10/U1702 class I integron DNA integrase IntI1 (intI1) gene, partial cds, AACA4 (aac6'-I) and metallo-beta-lactamase VIM-2 (blaVIM-2) genes, complete cds, and unknown genes.

GTGACCAACAGC---AACGATTCCGTCACACTGCGCCTCATGACTGAGCATGACCTTGCGATGCTCTATGAGTGGCTAAATCGATCTCATATCGTCGAGTGGTGGGGCGGAGAAGAAGCACGCCCGACACTTGCTGACGTACAGGAACAGTACTTGCCAAGCGTTTTAGCGCAAGAGTCCGTCACTCCATACATTGCAATGCTGAATGGAGAGCCGATTGGGTATGCCCAGTCGTACGTTGCTCTTGGAAGCGGGGACGGATGGTGGGAAGAAGAAACCGATCCAGGAGTACGCGGAATAGACCAGTCACTGGCGAATGCATCACAACTGGGCAAAGGCTTGGGAACCAAGCTGGTTCGAGCTCTGGTTGAGTTGCTGTTCAATGATCCCGAGGTCACCAAGATCCAAACGGACCCGTCGCCGAGCAACTTGCGAGCGATCCGATGCTACGAGAAAGCGGGGTTTGAGAGGCAAGGTACCGTAACCACCCCAGATGGTCCAGCCGTGTACATGGTTCAAACACGCCAGGCATTCGAGCGAACACGCAGTGTTGCCTAA

>AJ584652 Pseudomonas aeruginosa inti1 gene (partial), blaIMP-16 gene, aac(6')-30/aac(6')-Ib' fused gene, aacA4 gene, aadA1 gene, qacEdelta1 gene and sul1 gene (partial).

GTGACCAACAGC---AACGATTCCGTCACACTGCGCCTCATGACTGAGCATGACCTTGCGATGCTCTATGAGTGGCTAAATCGATCTCATATCGTCGAGTGGTGGGGCGGAGAAGAAGCACGCCCGACACTTGCTGACGTACAGGAACAGTACTTGCCAAGCGTTTTAGCGCAAGAGTCCGTCACTCCATACATTGCAATGCTGAATGGAGAGCCGATTGGGTATGCCCAGTCGTACGTTGCTCTTGGAAGCGGGGACGGATGGTGGGAAGAAGAAACCGATCCAGGAGTACGCGGAATAGACCAGTCACTGGCGAATGCATCACAACTGGGCAAAGGCTTGGGAACCAAGCTGGTTCGAGCTCTGGTTGAGTTGCTGTTCAATGATCCCGAGGTCACCAAGATCCAAACGGACCCGTCGCCGAGCAACTTGCGAGCGATCCGATGCTACGAGAAAGCGGGGTTTGAGAGGCAAGGTACCGTAACCACCCCAGATGGTCCAGCCGTGTACATGGTTCAAACACGCCAGGCATTCGAGCGAACACGCAGTGTTGCCTAA

>AY660529 Pseudomonas aeruginosa integron In163, complete sequence.

GTGACCAACAGC---AACGATTCCGTCACACTGCGCCTCATGACTGAGCATGACCTTGCGATGCTCTATGAGTGGCTAAATCGATCTCATATCGTCGAGTGGTGGGGCGGAGAAGAAGCACGCCCGACACTTGCTGACGTACAGGAACAGTACTTGCCAAGCGTTTTAGCGCAAGAGTCCGTCACTCCATACATTGCAATGCTGAATGGAGAGCCGATTGGGTATGCCCAGTCGTACGTTGCTCTTGGAAGCGGGGACGGATGGTGGGAAGAAGAAACCGATCCAGGAGTACGCGGAATAGACCAGTCACTGGCGAATGCATCACAACTGGGCAAAGGCTTGGGAACCAAGCTGGTTCGAGCTCTGGTTGAGTTGCTGTTCAATGATCCCGAGGTCACCAAGATCCAAACGGACCCGTCGCCGAGCAACTTGCGAGCGATCCGATGCTACGAGAAAGCGGGGTTTGAGAGGCAAGGTACCGTAACCACCCCAGATGGTCCAGCCGTGTACATGGTTCAAACACGCCAGGCATTCGAGCGAACACGCAGTGTTGCCTAA

>AF227505 Klebsiella pneumoniae plasmid pY2 class 1 integron aminoglycoside 6'-N-acetyltransferase (aac(6')-Ib), beta-lactamase (oxa-1), and streptomycin 3''-adenyltransferase (aadA2) genes, complete cds.

GTGACCAACAGC---AACGATTCCGTCACACTGCGCCTCATGACTGAGCATGACCTTGCGATGCTCTATGAGTGGCTAAATCGATCTCATATCGTCGAGTGGTGGGGCGGAGAAGAAGCACGCCCGACACTTGCTGACGTACAGGAACAGTACTTGCCAAGCGTTTTAGCGCAAGAGTCCGTCACTCCATACATTGCAATGCTGAATGGAGAGCCGATTGGGTATGCCCAGTCGTACGTTGCTCTTGGAAGCGGGGACGGATGGTGGGAAGAAGAAACCGATCCAGGAGTACGCGGAATAGACCAGTCACTGGCGAATGCATCACAACTGGGCAAAGGCTTGGGAACCAAGCTGGTTCGAGCTCTGGTTGAGTTGCTGTTCAATGATCCCGAGGTCACCAAGATCCAAACGGACCCGTCGCCGAGCAACTTGCGAGCGATCCGATGCTACGAGAAAGCGGGGTTTGAGAGGCAAGGTACCGTAACCACCCCAGATGGTCCAGCCGTGTACATGGTTCAAACACGCCAGGCATTCGAGCGAACACGCAGTGTTGCCTAA

>AY029772 Pseudomonas aeruginosa isolate YMC704 class I integron, partial sequence.

GTGACCAACAGC---AACGATTCCGTCACACTGCGCCTCATGACTGAGCATGACCTTGCGATGCTCTATGAGTGGCTAAATCGATCTCATATCGTCGAGTGGTGGGGCGGAGAAGAAGCACGCCCGACACTTGCTGACGTACAGGAACAGTACTTGCCAAGCGTTTTAGCGCAAGAGTCCGTCACTCCATACATTGCAATGCTGAATGGAGAGCCGATTGGGTATGCCCAGTCGTACGTTGCTCTTGGAAGCGGGGACGGATGGTGGGAAGAAGAAACCGATCCAGGAGTACGCGGAATAGACCAGTCACTGGCGAATGCATCACAACTGGGCAAAGGCTTGGGAACCAAGCTGGTTCGAGCTCTGGTTGAGTTGCTGTTCAATGATCCCGAGGTCACCAAGATCCAAACGGACCCGTCGCCGAGCAACTTGCGAGCGATCCGATGCTACGAGAAAGCGGGGTTTGAGAGGCAAGGTACCGTAACCACCCCAGATGGTCCAGCCGTGTACATGGTTCAAACACGCCAGGCATTCGAGCGAACACGCAGTGTTGCCTAA

>AY065966 Pseudomonas putida class I integron DNA integrase (intI1) gene, partial cds; aminoglycoside acetyltransferase (aacA6'-Ib) and metallo-beta-lactamase (blaVIM-2) genes, complete cds; and unknown genes.

GTGACCAACAGC---AACGATTCCGTCACACTGCGCCTCATGACTGAGCATGACCTTGCGATGCTCTATGAGTGGCTAAATCGATCTCATATCGTCGAGTGGTGGGGCGGAGAAGAAGCACGCCCGACACTTGCTGACGTACAGGAACAGTACTTGCCAAGCGTTTTAGCGCAAGAGTCCGTCACTCCATACATTGCAATGCTGAATGGAGAGCCGATTGGGTATGCCCAGTCGTACGTTGCTCTTGGAAGCGGGGACGGATGGTGGGAAGAAGAAACCGATCCAGGAGTACGCGGAATAGACCAGTCACTGGCGAATGCATCACAACTGGGCAAAGGCTTGGGAACCAAGCTGGTTCGAGCTCTGGTTGAGTTGCTGTTCAATGATCCCGAGGTCACCAAGATCCAAACGGACCCGTCGCCGAGCAACTTGCGAGCGATCCGATGCTACGAGAAAGCGGGGTTTGAGAGGCAAGGTACCGTAACCACCCCAGATGGTCCAGCCGTGTACATGGTTCAAACACGCCAGGCATTCGAGCGAACACGCAGTGTTGCCTAA

>AY123251 Salmonella enterica subsp. enterica serovar Typhi R-plasmid pST2301 class I integron aminoglycoside 6'-N-acetyltransferase (aacA4), chloramphenicol acetyltransferase (catB8), aminoglycoside 3'-adenyltransferase (aadA1), dihydrofolate reductase type I (dhfr1), aminoglycoside 6'-n-acetyltransferase (aac6-II), and CARB-8 beta-lactamase (blaCARB-8) genes, complete cds.

GTGACCAACAGC---AACGATTCCGTCACACTGCGCCTCATGACTGAGCATGACCTTGCGATGCTCTATGAGTGGCTAAATCGATCTCATATCGTCGAGTGGTGGGGCGGAGAAGAAGCACGCCCGACACTTGCTGACGTACAGGAACAGTACTTGCCAAGCGTTTTAGCGCAAGAGTCCGTCACTCCATACATTGCAATGCTGAATGGAGAGCCGATTGGGTATGCCCAGTCGTACGTTGCTCTTGGAAGCGGGGACGGATGGTGGGAAGAAGAAACCGATCCAGGAGTACGCGGAATAGACCAGTCACTGGCGAATGCATCACAACTGGGCAAAGGCTTGGGAACCAAGCTGGTTCGAGCTCTGGTTGAGTTGCTGTTCAATGATCCCGAGGTCACCAAGATCCAAACGGACCCGTCGCCGAGCAACTTGCGAGCGATCCGATGCTACGAGAAAGCGGGGTTTGAGAGGCAAGGTACCGTAACCACCCCAGATGGTCCAGCCGTGTACATGGTTCAAACACGCCAGGCATTCGAGCGAACACGCAGTGTTGCCTAA

>AY560837 Pseudomonas aeruginosa class 1 integron In100, partial sequence.

GTGACCAACAGC---AACGATTCCGTCACACTGCGCCTCATGACTGAGCATGACCTTGCGATGCTCTATGAGTGGCTAAATCGATCTCATATCGTCGAGTGGTGGGGCGGAGAAGAAGCACGCCCGACACTTGCTGACGTACAGGAACAGTACTTGCCAAGCGTTTTAGCGCAAGAGTCCGTCACTCCATACATTGCAATGCTGAATGGAGAGCCGATTGGGTATGCCCAGTCGTACGTTGCTCTTGGAAGCGGGGACGGATGGTGGGAAGAAGAAACCGATCCAGGAGTACGCGGAATAGACCAGTCACTGGCGAATGCATCACAACTGGGCAAAGGCTTGGGAACCAAGCTGGTTCGAGCTCTGGTTGAGTTGCTGTTCAATGATCCCGAGGTCACCAAGATCCAAACGGACCCGTCGCCGAGCAACTTGCGAGCGATCCGATGCTACGAGAAAGCGGGGTTTGAGAGGCAAGGTACCGTAACCACCCCAGATGGTCCAGCCGTGTACATGGTTCAAACACGCCAGGCATTCGAGCGAACACGCAGTGTTGCCTAA

>AB188812 Pseudomonas aeruginosa class 1 integron intI1, blaIMP-6, aacA4, aadA1, blaOXA-2 genes for DNA integrase, metallo-beta-lactamase IMP-6, aminoglycoside modifying enzyme, aminoglycoside modifying enzyme, beta-lactamase OXA-2, partial and complete cds.

GTGACCAACAGC---AACGATTCCGTCACACTGCGCCTCATGACTGAGCATGACCTTGCGATGCTCTATGAGTGGCTAAATCGATCTCATATCGTCGAGTGGTGGGGCGGAGAAGAAGCACGCCCGACACTTGCTGACGTACAGGAACAGTACTTGCCAAGCGTTTTAGCGCAAGAGTCCGTCACTCCATACATTGCAATGCTGAATGGAGAGCCGATTGGGTATGCCCAGTCGTACGTTGCTCTTGGAAGCGGGGACGGATGGTGGGAAGAAGAAACCGATCCAGGAGTACGCGGAATAGACCAGTCACTGGCGAATGCATCACAACTGGGCAAAGGCTTGGGAACCAAGCTGGTTCGAGCTCTGGTTGAGTTGCTGTTCAATGATCCCGAGGTCACCAAGATCCAAACGGACCCGTCGCCGAGCAACTTGCGAGCGATCCGATGCTACGAGAAAGCGGGGTTTGAGAGGCAAGGTACCGTAACCACCCCAGATGGTCCAGCCGTGTACATGGTTCAAACACGCCAGGCATTCGAGCGAACACGCAGTGTTGCCTAA

>AJ784256 Pseudomonas aeruginosa partial intI1 gene, blaVIM-1 gene, smr-2 gene, ORF1, aacA4 gene, pse-1 gene and partial qacEdelta1 gene.

GTGACCAACAGC---AACGATTCCGTCACACTGCGCCTCATGACTGAGCATGACCTTGCGATGCTCTATGAGTGGCTAAATCGATCTCATATCGTCGAGTGGTGGGGCGGAGAAGAAGCACGCCCGACACTTGCTGACGTACAGGAACAGTACTTGCCAAGCGTTTTAGCGCAAGAGTCCGTCACTCCATACATTGCAATGCTGAATGGAGAGCCGATTGGGTATGCCCAGTCGTACGTTGCTCTTGGAAGCGGGGACGGATGGTGGGAAGAAGAAACCGATCCAGGAGTACGCGGAATAGACCAGTCACTGGCGAATGCATCACAACTGGGCAAAGGCTTGGGAACCAAGCTGGTTCGAGCTCTGGTTGAGTTGCTGTTCAATGATCCCGAGGTCACCAAGATCCAAACGGACCCGTCGCCGAGCAACTTGCGAGCGATCCGATGCTACGAGAAAGCGGGGTTTGAGAGGCAAGGTACCGTAACCACCCCAGATGGTCCAGCCGTGTACATGGTTCAAACACGCCAGGCATTCGAGCGAACACGCAGTGTTGCCTAA

>AY922989 Acinetobacter baumannii isolate 6B(P) class I integron aminoglycoside 6'-N-acetyltransferase (aacA4), chloramphenicol acetyltransferase (catB8), and aminoglycoside 3'-adenyltransferase (aadA1) genes, complete cds.

GTGACCAACAGC---AACGATTCCGTCACACTGCGCCTCATGACTGAGCATGACCTTGCGATGCTCTATGAGTGGCTAAATCGATCTCATATCGTCGAGTGGTGGGGCGGAGAAGAAGCACGCCCGACACTTGCTGACGTACAGGAACAGTACTTGCCAAGCGTTTTAGCGCAAGAGTCCGTCACTCCATACATTGCAATGCTGAATGGAGAGCCGATTGGGTATGCCCAGTCGTACGTTGCTCTTGGAAGCGGGGACGGATGGTGGGAAGAAGAAACCGATCCAGGAGTACGCGGAATAGACCAGTCACTGGCGAATGCATCACAACTGGGCAAAGGCTTGGGAACCAAGCTGGTTCGAGCTCTGGTTGAGTTGCTGTTCAATGATCCCGAGGTCACCAAGATCCAAACGGACCCGTCGCCGAGCAACTTGCGAGCGATCCGATGCTACGAGAAAGCGGGGTTTGAGAGGCAAGGTACCGTAACCACCCCAGATGGTCCAGCCGTGTACATGGTTCAAACACGCCAGGCATTCGAGCGAACACGCAGTGTTGCCTAA

>AY913771 Pseudomonas aeruginosa isolate PS 83 class 1 integron, partial sequence.

GTGACCAACAGC---AACGATTCCGTCACACTGCGCCTCATGACTGAGCATGACCTTGCGATGCTCTATGAGTGGCTAAATCGATCTCATATCGTCGAGTGGTGGGGCGGAGAAGAAGCACGCCCGACACTTGCTGACGTACAGGAACAGTACTTGCCAAGCGTTTTAGCGCAAGAGTCCGTCACTCCATACATTGCAATGCTGAATGGAGAGCCGATTGGGTATGCCCAGTCGTACGTTGCTCTTGGAAGCGGGGACGGATGGTGGGAAGAAGAAACCGATCCAGGAGTACGCGGAATAGACCAGTCACTGGCGAATGCATCACAACTGGGCAAAGGCTTGGGAACCAAGCTGGTTCGAGCTCTGGTTGAGTTGCTGTTCAATGATCCCGAGGTCACCAAGATCCAAACGGACCCGTCGCCGAGCAACTTGCGAGCGATCCGATGCTACGAGAAAGCGGGGTTTGAGAGGCAAGGTACCGTAACCACCCCAGATGGTCCAGCCGTGTACATGGTTCAAACACGCCAGGCATTCGAGCGAACACGCAGTGTTGCCTAA

>AY913772 Pseudomonas aeruginosa isolate PS 158 class 1 integron, partial sequence.

GTGACCAACAGC---AACGATTCCGTCACACTGCGCCTCATGACTGAGCATGACCTTGCGATGCTCTATGAGTGGCTAAATCGATCTCATATCGTCGAGTGGTGGGGCGGAGAAGAAGCACGCCCGACACTTGCTGACGTACAGGAACAGTACTTGCCAAGCGTTTTAGCGCAAGAGTCCGTCACTCCATACATTGCAATGCTGAATGGAGAGCCGATTGGGTATGCCCAGTCGTACGTTGCTCTTGGAAGCGGGGACGGATGGTGGGAAGAAGAAACCGATCCAGGAGTACGCGGAATAGACCAGTCACTGGCGAATGCATCACAACTGGGCAAAGGCTTGGGAACCAAGCTGGTTCGAGCTCTGGTTGAGTTGCTGTTCAATGATCCCGAGGTCACCAAGATCCAAACGGACCCGTCGCCGAGCAACTTGCGAGCGATCCGATGCTACGAGAAAGCGGGGTTTGAGAGGCAAGGTACCGTAACCACCCCAGATGGTCCAGCCGTGTACATGGTTCAAACACGCCAGGCATTCGAGCGAACACGCAGTGTTGCCTAA

>DQ141317 Klebsiella pneumoniae class I integron aminoglycoside 6'-N-acetyltransferase (aacA4), chloramphenicol acetyltransferase (catB8), and aminoglycoside 3'-adenyltransferase (aadA1) genes, complete cds.

GTGACCAACAGC---AACGATTCCGTCACACTGCGCCTCATGACTGAGCATGACCTTGCGATGCTCTATGAGTGGCTAAATCGATCTCATATCGTCGAGTGGTGGGGCGGAGAAGAAGCACGCCCGACACTTGCTGACGTACAGGAACAGTACTTGCCAAGCGTTTTAGCGCAAGAGTCCGTCACTCCATACATTGCAATGCTGAATGGAGAGCCGATTGGGTATGCCCAGTCGTACGTTGCTCTTGGAAGCGGGGACGGATGGTGGGAAGAAGAAACCGATCCAGGAGTACGCGGAATAGACCAGTCACTGGCGAATGCATCACAACTGGGCAAAGGCTTGGGAACCAAGCTGGTTCGAGCTCTGGTTGAGTTGCTGTTCAATGATCCCGAGGTCACCAAGATCCAAACGGACCCGTCGCCGAGCAACTTGCGAGCGATCCGATGCTACGAGAAAGCGGGGTTTGAGAGGCAAGGTACCGTAACCACCCCAGATGGTCCAGCCGTGTACATGGTTCAAACACGCCAGGCATTCGAGCGAACACGCAGTGTTGCCTAA

>DQ139278 Pseudomonas aeruginosa isolate PS408 class 1 integron, partial sequence.

GTGACCAACAGC---AACGATTCCGTCACACTGCGCCTCATGACTGAGCATGACCTTGCGATGCTCTATGAGTGGCTAAATCGATCTCATATCGTCGAGTGGTGGGGCGGAGAAGAAGCACGCCCGACACTTGCTGACGTACAGGAACAGTACTTGCCAAGCGTTTTAGCGCAAGAGTCCGTCACTCCATACATTGCAATGCTGAATGGAGAGCCGATTGGGTATGCCCAGTCGTACGTTGCTCTTGGAAGCGGGGACGGATGGTGGGAAGAAGAAACCGATCCAGGAGTACGCGGAATAGACCAGTCACTGGCGAATGCATCACAACTGGGCAAAGGCTTGGGAACCAAGCTGGTTCGAGCTCTGGTTGAGTTGCTGTTCAATGATCCCGAGGTCACCAAGATCCAAACGGACCCGTCGCCGAGCAACTTGCGAGCGATCCGATGCTACGAGAAAGCGGGGTTTGAGAGGCAAGGTACCGTAACCACCCCAGATGGTCCAGCCGTGTACATGGTTCAAACACGCCAGGCATTCGAGCGAACACGCAGTGTTGCCTAA

>DQ139279 Pseudomonas aeruginosa isolate PS99 class 1 integron, partial sequence.

GTGACCAACAGC---AACGATTCCGTCACACTGCGCCTCATGACTGAGCATGACCTTGCGATGCTCTATGAGTGGCTAAATCGATCTCATATCGTCGAGTGGTGGGGCGGAGAAGAAGCACGCCCGACACTTGCTGACGTACAGGAACAGTACTTGCCAAGCGTTTTAGCGCAAGAGTCCGTCACTCCATACATTGCAATGCTGAATGGAGAGCCGATTGGGTATGCCCAGTCGTACGTTGCTCTTGGAAGCGGGGACGGATGGTGGGAAGAAGAAACCGATCCAGGAGTACGCGGAATAGACCAGTCACTGGCGAATGCATCACAACTGGGCAAAGGCTTGGGAACCAAGCTGGTTCGAGCTCTGGTTGAGTTGCTGTTCAATGATCCCGAGGTCACCAAGATCCAAACGGACCCGTCGCCGAGCAACTTGCGAGCGATCCGATGCTACGAGAAAGCGGGGTTTGAGAGGCAAGGTACCGTAACCACCCCAGATGGTCCAGCCGTGTACATGGTTCAAACACGCCAGGCATTCGAGCGAACACGCAGTGTTGCCTAA

>DQ139280 Pseudomonas aeruginosa isolate PS2588 class 1 integron, partial sequence.

GTGACCAACAGC---AACGATTCCGTCACACTGCGCCTCATGACTGAGCATGACCTTGCGATGCTCTATGAGTGGCTAAATCGATCTCATATCGTCGAGTGGTGGGGCGGAGAAGAAGCACGCCCGACACTTGCTGACGTACAGGAACAGTACTTGCCAAGCGTTTTAGCGCAAGAGTCCGTCACTCCATACATTGCAATGCTGAATGGAGAGCCGATTGGGTATGCCCAGTCGTACGTTGCTCTTGGAAGCGGGGACGGATGGTGGGAAGAAGAAACCGATCCAGGAGTACGCGGAATAGACCAGTCACTGGCGAATGCATCACAACTGGGCAAAGGCTTGGGAACCAAGCTGGTTCGAGCTCTGGTTGAGTTGCTGTTCAATGATCCCGAGGTCACCAAGATCCAAACGGACCCGTCGCCGAGCAACTTGCGAGCGATCCGATGCTACGAGAAAGCGGGGTTTGAGAGGCAAGGTACCGTAACCACCCCAGATGGTCCAGCCGTGTACATGGTTCAAACACGCCAGGCATTCGAGCGAACACGCAGTGTTGCCTAA

>DQ219465 Pseudomonas aeruginosa In99 class 1 integron, partial sequence.

GTGACCAACAGC---AACGATTCCGTCACACTGCGCCTCATGACTGAGCATGACCTTGCGATGCTCTATGAGTGGCTAAATCGATCTCATATCGTCGAGTGGTGGGGCGGAGAAGAAGCACGCCCGACACTTGCTGACGTACAGGAACAGTACTTGCCAAGCGTTTTAGCGCAAGAGTCCGTCACTCCATACATTGCAATGCTGAATGGAGAGCCGATTGGGTATGCCCAGTCGTACGTTGCTCTTGGAAGCGGGGACGGATGGTGGGAAGAAGAAACCGATCCAGGAGTACGCGGAATAGACCAGTCACTGGCGAATGCATCACAACTGGGCAAAGGCTTGGGAACCAAGCTGGTTCGAGCTCTGGTTGAGTTGCTGTTCAATGATCCCGAGGTCACCAAGATCCAAACGGACCCGTCGCCGAGCAACTTGCGAGCGATCCGATGCTACGAGAAAGCGGGGTTTGAGAGGCAAGGTACCGTAACCACCCCAGATGGTCCAGCCGTGTACATGGTTCAAACACGCCAGGCATTCGAGCGAACACGCAGTGTTGCCTAA

>AJ634050 Pseudomonas aeruginosa partial Tn5051-like transposon and class 1 integron In85, strain 85MG.

GTGACCAACAGC---AACGATTCCGTCACACTGCGCCTCATGACTGAGCATGACCTTGCGATGCTCTATGAGTGGCTAAATCGATCTCATATCGTCGAGTGGTGGGGCGGAGAAGAAGCACGCCCGACACTTGCTGACGTACAGGAACAGTACTTGCCAAGCGTTTTAGCGCAAGAGTCCGTCACTCCATACATTGCAATGCTGAATGGAGAGCCGATTGGGTATGCCCAGTCGTACGTTGCTCTTGGAAGCGGGGACGGATGGTGGGAAGAAGAAACCGATCCAGGAGTACGCGGAATAGACCAGTCACTGGCGAATGCATCACAACTGGGCAAAGGCTTGGGAACCAAGCTGGTTCGAGCTCTGGTTGAGTTGCTGTTCAATGATCCCGAGGTCACCAAGATCCAAACGGACCCGTCGCCGAGCAACTTGCGAGCGATCCGATGCTACGAGAAAGCGGGGTTTGAGAGGCAAGGTACCGTAACCACCCCAGATGGTCCAGCCGTGTACATGGTTCAAACACGCCAGGCATTCGAGCGAACACGCAGTGTTGCCTAA

>EF031554 Salmonella enterica subsp. enterica serovar Senftenberg aminoglycoside 6'-N-acetyltansferase (aacA4) gene, complete cds.

GTGACCAACAGC---AACGATTCCGTCACACTGCGCCTCATGACTGAGCATGACCTTGCGATGCTCTATGAGTGGCTAAATCGATCTCATATCGTCGAGTGGTGGGGCGGAGAAGAAGCACGCCCGACACTTGCTGACGTACAGGAACAGTACTTGCCAAGCGTTTTAGCGCAAGAGTCCGTCACTCCATACATTGCAATGCTGAATGGAGAGCCGATTGGGTATGCCCAGTCGTACGTTGCTCTTGGAAGCGGGGACGGATGGTGGGAAGAAGAAACCGATCCAGGAGTACGCGGAATAGACCAGTCACTGGCGAATGCATCACAACTGGGCAAAGGCTTGGGAACCAAGCTGGTTCGAGCTCTGGTTGAGTTGCTGTTCAATGATCCCGAGGTCACCAAGATCCAAACGGACCCGTCGCCGAGCAACTTGCGAGCGATCCGATGCTACGAGAAAGCGGGGTTTGAGAGGCAAGGTACCGTAACCACCCCAGATGGTCCAGCCGTGTACATGGTTCAAACACGCCAGGCATTCGAGCGAACACGCAGTGTTGCCTAA

>AM392427 Pseudomonas aeruginosa partial class I integron InCRO.

GTGACCAACAGC---AACGATTCCGTCACACTGCGCCTCATGACTGAGCATGACCTTGCGATGCTCTATGAGTGGCTAAATCGATCTCATATCGTCGAGTGGTGGGGCGGAGAAGAAGCACGCCCGACACTTGCTGACGTACAGGAACAGTACTTGCCAAGCGTTTTAGCGCAAGAGTCCGTCACTCCATACATTGCAATGCTGAATGGAGAGCCGATTGGGTATGCCCAGTCGTACGTTGCTCTTGGAAGCGGGGACGGATGGTGGGAAGAAGAAACCGATCCAGGAGTACGCGGAATAGACCAGTCACTGGCGAATGCATCACAACTGGGCAAAGGCTTGGGAACCAAGCTGGTTCGAGCTCTGGTTGAGTTGCTGTTCAATGATCCCGAGGTCACCAAGATCCAAACGGACCCGTCGCCGAGCAACTTGCGAGCGATCCGATGCTACGAGAAAGCGGGGTTTGAGAGGCAAGGTACCGTAACCACCCCAGATGGTCCAGCCGTGTACATGGTTCAAACACGCCAGGCATTCGAGCGAACACGCAGTGTTGCCTAA

>EF370423 Escherichia coli plasmid pUMB-9, partial sequence.

GTGACCAACAGC---AACGATTCCGTCACACTGCGCCTCATGACTGAGCATGACCTTGCGATGCTCTATGAGTGGCTAAATCGATCTCATATCGTCGAGTGGTGGGGCGGAGAAGAAGCACGCCCGACACTTGCTGACGTACAGGAACAGTACTTGCCAAGCGTTTTAGCGCAAGAGTCCGTCACTCCATACATTGCAATGCTGAATGGAGAGCCGATTGGGTATGCCCAGTCGTACGTTGCTCTTGGAAGCGGGGACGGATGGTGGGAAGAAGAAACCGATCCAGGAGTACGCGGAATAGACCAGTCACTGGCGAATGCATCACAACTGGGCAAAGGCTTGGGAACCAAGCTGGTTCGAGCTCTGGTTGAGTTGCTGTTCAATGATCCCGAGGTCACCAAGATCCAAACGGACCCGTCGCCGAGCAACTTGCGAGCGATCCGATGCTACGAGAAAGCGGGGTTTGAGAGGCAAGGTACCGTAACCACCCCAGATGGTCCAGCCGTGTACATGGTTCAAACACGCCAGGCATTCGAGCGAACACGCAGTGTTGCCTAA

>AM087410 Pseudomonas aeruginosa class 1 integron containing blaVIM-4 and aacA4 gene cassettes.

GTGACCAACAGC---AACGATTCCGTCACACTGCGCCTCATGACTGAGCATGACCTTGCGATGCTCTATGAGTGGCTAAATCGATCTCATATCGTCGAGTGGTGGGGCGGAGAAGAAGCACGCCCGACACTTGCTGACGTACAGGAACAGTACTTGCCAAGCGTTTTAGCGCAAGAGTCCGTCACTCCATACATTGCAATGCTGAATGGAGAGCCGATTGGGTATGCCCAGTCGTACGTTGCTCTTGGAAGCGGGGACGGATGGTGGGAAGAAGAAACCGATCCAGGAGTACGCGGAATAGACCAGTCACTGGCGAATGCATCACAACTGGGCAAAGGCTTGGGAACCAAGCTGGTTCGAGCTCTGGTTGAGTTGCTGTTCAATGATCCCGAGGTCACCAAGATCCAAACGGACCCGTCGCCGAGCAACTTGCGAGCGATCCGATGCTACGAGAAAGCGGGGTTTGAGAGGCAAGGTACCGTAACCACCCCAGATGGTCCAGCCGTGTACATGGTTCAAACACGCCAGGCATTCGAGCGAACACGCAGTGTTGCCTAA

>AF479774 Klebsiella pneumoniae plasmid pJHCMW1, complete sequence.

GTGACCAACAGC---AACGATTCCGTCACACTGCGCCTCATGACTGAGCATGACCTTGCGATGCTCTATGAGTGGCTAAATCGATCTCATATCGTCGAGTGGTGGGGCGGAGAAGAAGCACGCCCGACACTTGCTGACGTACAGGAACAGTACTTGCCAAGCGTTTTAGCGCAAGAGTCCGTCACTCCATACATTGCAATGCTGAATGGAGAGCCGATTGGGTATGCCCAGTCGTACGTTGCTCTTGGAAGCGGGGACGGATGGTGGGAAGAAGAAACCGATCCAGGAGTACGCGGAATAGACCAGTTACTGGCGAATGCATCACAACTGGGCAAAGGCTTGGGAACCAAGCTGGTTCGAGCTCTGGTTGAGTTGCTGTTCAATGATCCCGAGGTCACCAAGATCCAAACGGACCCGTCGCCGAGCAACTTGCGAGCGATCCGATGCTACGAGAAAGCGGGGTTTGAGAGGCAAGGTACCGTAACCACCCCAGATGGTCCAGCCGTGTACATGGTTCAAACACGCCAGGCATTCGAGCGAACACGCAGTGTTGCCTAA

>KPNAACA4 Plasmid pJHC-MW1 aminoglycoside 6'-N-acetyltransferase (aacA4) gene, complete cds, and streptomycin-spectinomycin resistance (aadA) gene, 5' end.

GTGACCAACAGC---AACGATTCCGTCACACTGCGCCTCATGACTGAGCATGACCTTGCGATGCTCTATGAGTGGCTAAATCGATCTCATATCGTCGAGTGGTGGGGCGGAGAAGAAGCACGCCCGACACTTGCTGACGTACAGGAACAGTACTTGCCAAGCGTTTTAGCGCAAGAGTCCGTCACTCCATACATTGCAATGCTGAATGGAGAGCCGATTGGGTATGCCCAGTCGTACGTTGCTCTTGGAAGCGGGGACGGATGGTGGGAAGAAGAAACCGATCCAGGAGTACGCGGAATAGACCAGTTACTGGCGAATGCATCACAACTGGGCAAAGGCTTGGGAACCAAGCTGGTTCGAGCTCTGGTTGAGTTGCTGTTCAATGATCCCGAGGTCACCAAGATCCAAACGGACCCGTCGCCGAGCAACTTGCGAGCGATCCGATGCTACGAGAAAGCGGGGTTTGAGAGGCAAGGTACCGTAACCACCCCAGATGGTCCAGCCGTGTACATGGTTCAAACACGCCAGGCATTCGAGCGAACACGCAGTGTTGCCTAA

>TRNTAAB Klebsiella pneumoniae transposon Tn1331, complete sequence.

GTGACCAACAGC---AACGATTCCGTCACACTGCGCCTCATGACTGAGCATGACCTTGCGATGCTCTATGAGTGGCTAAATCGATCTCATATCGTCGAGTGGTGGGGCGGAGAAGAAGCACGCCCGACACTTGCTGACGTACAGGAACAGTACTTGCCAAGCGTTTTAGCGCAAGAGTCCGTCACTCCATACATTGCAATGCTGAATGGAGAGCCGATTGGGTATGCCCAGTCGTACGTTGCTCTTGGAAGCGGGGACGGATGGTGGGAAGAAGAAACCGATCCAGGAGTACGCGGAATAGACCAGTTACTGGCGAATGCATCACAACTGGGCAAAGGCTTGGGAACCAAGCTGGTTCGAGCTCTGGTTGAGTTGCTGTTCAATGATCCCGAGGTCACCAAGATCCAAACGGACCCGTCGCCGAGCAACTTGCGAGCGATCCGATGCTACGAGAAAGCGGGGTTTGAGAGGCAAGGTACCGTAACCACCCCAGATGGTCCAGCCGTGTACATGGTTCAAACACGCCAGGCATTCGAGCGAACACGCAGTGTTGCCTAA

>DQ174113 Pseudomonas putida transposon Tn1332, complete sequence.

GTGACCAACAGC---AACGATTCCGTCACACTGCGCCTCATGACTGAGCATGACCTTGCGATGCTCTATGAGTGGCTAAATCGATCTCATATCGTCGAGTGGTGGGGCGGAGAAGAAGCACGCCCGACACTTGCTGACGTACAGGAACAGTACTTGCCAAGCGTTTTAGCGCAAGAGTCCGTCACTCCATACATTGCAATGCTGAATGGAGAGCCGATTGGGTATGCCCAGTCGTACGTTGCTCTTGGAAGCGGGGACGGATGGTGGGAAGAAGAAACCGATCCAGGAGTACGCGGAATAGACCAGTTACTGGCGAATGCATCACAACTGGGCAAAGGCTTGGGAACCAAGCTGGTTCGAGCTCTGGTTGAGTTGCTGTTCAATGATCCCGAGGTCACCAAGATCCAAACGGACCCGTCGCCGAGCAACTTGCGAGCGATCCGATGCTACGAGAAAGCGGGGTTTGAGAGGCAAGGTACCGTAACCACCCCAGATGGTCCAGCCGTGTACATGGTTCAAACACGCCAGGCATTCGAGCGAACACGCAGTGTTGCCTAA

>CP000649 Klebsiella pneumoniae subsp. pneumoniae MGH 78578 plasmid pKPN4, complete sequence.

GTGACCAACAGC---AACGATTCCGTCACACTGCGCCTCATGACTGAGCATGACCTTGCGATGCTCTATGAGTGGCTAAATCGATCTCATATCGTCGAGTGGTGGGGCGGAGAAGAAGCACGCCCGACACTTGCTGACGTACAGGAACAGTACTTGCCAAGCGTTTTAGCGCAAGAGTCCGTCACTCCATACATTGCAATGCTGAATGGAGAGCCGATTGGGTATGCCCAGTCGTACGTTGCTCTTGGAAGCGGGGACGGATGGTGGGAAGAAGAAACCGATCCAGGAGTACGCGGAATAGACCAGTTACTGGCGAATGCATCACAACTGGGCAAAGGCTTGGGAACCAAGCTGGTTCGAGCTCTGGTTGAGTTGCTGTTCAATGATCCCGAGGTCACCAAGATCCAAACGGACCCGTCGCCGAGCAACTTGCGAGCGATCCGATGCTACGAGAAAGCGGGGTTTGAGAGGCAAGGTACCGTAACCACCCCAGATGGTCCAGCCGTGTACATGGTTCAAACACGCCAGGCATTCGAGCGAACACGCAGTGTTGCCTAA

>AY878717 Escherichia coli plasmid pKO97, partial sequence.

GTGACCAACAGC---AACGATTCCGTCACACTGCGCCTCATGACTGAGCATGACCTTGCGATGCTCTATGAGTGGCTAAATCGATCTCATATCGTCGAGTGGTGGGGCGGAGAAGAAGCACGCCCGACACTTGCTGACGTACAGGAACAGTACTTGCCAAGCGTTTTAGCGCAAGAGTCCGTCACTCCATACATTGCAATGCTGAATGGAGAGCCGATTGGGTATGCCCAGTCGTACGTTGCTCTTGGAAGCGGGGACGGATGGTGGGAAGAAGAAACCGATCCAGGAGTACGCGGAATAGACCAGTTACTGGCGAATGCATCACAACTGGGCAAAGGCTTGGGAACCAAGCTGGTTCGAGCTCTGGTTGAGTTGCTGTTCAATGATCCCGAGGTCACCAAGATCCAAACGGACCCGTCGCCGAGCAACTTGCGAGCGATCCGATGCTACGAGAAAGCGGGGTTTGAGAGGCAAGGTACCGTAACCACCCCAGATGGTCCAGCCGTGTACATGGTTCAAACACGCCAGGCATTCGAGCGAACACGCAGTGTTGCCTAA

>KPNAACA4 Plasmid pJHC-MW1 aminoglycoside 6'-N-acetyltransferase (aacA4) gene, complete cds, and streptomycin-spectinomycin resistance (aadA) gene, 5' end.

GTGACCAACAGC---AACGATTCCGTCACACTGCGCCTCATGACTGAGCATGACCTTGCGATGCTCTATGAGTGGCTAAATCGATCTCATATCGTCGAGTGGTGGGGCGGAGAAGAAGCACGCCCGACACTTGCTGACGTACAGGAACAGTACTTGCCAAGCGTTTTAGCGCAAGAGTCCGTCACTCCATACATTGCAATGCTGAATGGAGAGCCGATTGGGTATGCCCAGTCGTACGTTGCTCTTGGAAGCGGGGACGGATGGTGGGAAGAAGAAACCGATCCAGGAGTACGCGGAATAGACCAGTTACTGGCGAATGCATCACAACTGGGCAAAGGCTTGGGAACCAAGCTGGTTCGAGCTCTGGTTGAGTTGCTGTTCAATGATCCCGAGGTCACCAAGATCCAAACGGACCCGTCGCCGAGCAACTTGCGAGCGATCCGATGCTACGAGAAAGCGGGGTTTGAGAGGCAAGGTACCGTAACCACCCCAGATGGTCCAGCCGTGTACATGGTTCAAACACGCCAGGCATTCGAGCGAACACGCAGTGTTGCCTAA

>AB219236 Vibrio cholerae O1 aar-3, aacA4, dfrA1 genes, complete cds.

GTGACCAACAGC---AACGATTCCGTCACACTGCGCCTCATGACTGAGCATGACCTTGCGATGCTCTATGAGTGGCTAAATCGATCTCATATCGTCGAGTGGTGGGGCGGAGAAGAAGCACGCCCGACACTTGCTGACGTACAGGAACAGTACTTGCCAAGCGTTTTAGCGCAAGAGTCCGTCACTCCATACATTGCAATGCTGAATGGAGAGCCGATTGGGTATGCCCAGTCGTACGTTGCTCTTGGAAGCGGGGACGGATGGTGGGAAGAAGAAACCGATCCAGGAGTACGCGGAATAGACCAGTCACTGGCGAATGCATCACAACTGGGCAAAGGCTTGGGAACCAAGCTGGTTCGAGCTCTGGTTGAGTTGCTGTTCAATGATCCCGAGGTCACCAAGATCCAAACGGACCCGTCGCCGAGCAACTTGCGAGCGATCCGATGCTACGAGAAAGCGGGGTTTGAGAGGCAAGGTACCGTAACCACCCCAGATGGTCCAGCCGTGTACATGGTTCAAACACGCCAGGCATTCGAGCGAACACGCAGTGATGCCTAA

>AY103455 Vibrio cholerae class I integron aminoglycoside 6'-N-acetyl transferase gene, complete cds.

GTGACCAACAGC---AACGATTCCGTCACACTGCGCCTCATGACTGAGCATGACCTTGCGATGCTCTATGAGTGGCTAAATCGATCTCATATCGTCGAGTGGTGGGGCGGAGAAGAAGCACGCCCGACACTTGCTGACGTACAGGAACAGTACTTGCCAAGCGTTTTAGCGCAAGAGTCCGTCACTCCATACATTGCAATGCTGAATGGAGAGCCGATTGGGTATGCCCAGTCGTACGTTGCTCTTGGAAGCGGGGACGGATGGTGGGAAGAAGAAACCGATCCAGGAGTACGCGGAATAGACCAGTCACTGGCGAATGCATCACAACTGGGCAAAGGCTTGGGAACCAAGCTGGTTCGAGCTCTGGTTGAGTTGCTGTTCAATGATCCCGAGGTCACCAAGATCCAAACGGACCCGTCGCCGAGCAACTTGCGAGCGATCCGATGCTACGAGAAAGCGGGGTTTGAGAGGCAAGGTACCGTAACCACCCCAGATGGTCCAGCCGTGTACATGGTTCAAACACGCCAGGCATTCGAGCGAACACGCAGTGATGCCTAA

>AF043381 Pseudomonas aeruginosa integrase (intI) gene, partial cds; aminoglycoside 6'-N-acetyltransferase (aac(6')-Ib9) and beta-lactamase (bla oxa-19) genes, complete cds; and qacEdelta1 gene, partial cds.

GTGACCAACAGC---AACGATTCCGTCACACTGCGCCTCATGACTGAGCATGACCTTGCGATGCTCTATGAGTGGCTAAATCGATCTCATATCGTCGAGTGGTGGGGCGGAGAAGAAGCACGCCCGACACTTGCTGACGTACAGGAACAGTACTTGCCAAGCGTTTTAGCGCAAGAGTCCGTCACTCCATACATTGCAATGCTGAATGGAGAGCCGATTGGGTATGCCCAGTCGTACGTTGCTCTTGGAAGCGGGGACGGATGGTGGGAAGAAGAAACCGATCCAGGAGTACGCGGAATAGACCAGTCACTGGCGAATGCATCACAACTGGGCAAAGGCTTGGGAACCAAGCTGGTTCGAGCTCTGGTTGAGTTGCTGTTCAATGATCCCGAGGTCACCAAGATCCAAACGGACCCGTCGCCGAGCAACTTGCGAGCGATCCGATGCTACGAGAAAGCGGGGTTTGAGAGGCAAGGTACCGTAACCACCCCAGATGGTCCAGCCGTGTACATGGTTCAAACACGCCAGGCATTCGAGCGAACACGCAGTGATGCCTAA

>AF231133 Pseudomonas aeruginosa INTI1 (intI1) gene, partial cds; and AAC(6')-Ib (aac(6')-Ib) and OXA-28 (oxa-28) genes, complete cds.

GTGACCAACAGC---AACGATTCCGTCACACTGCGCCTCATGACTGAGCATGACCTTGCGATGCTCTATGAGTGGCTAAATCGATCTCATATCGTCGAGTGGTGGGGCGGAGAAGAAGCACGCCCGACACTTGCTGACGTACAGGAACAGTACTTGCCAAGCGTTTTAGCGCAAGAGTCCGTCACTCCATACATTGCAATGCTGAATGGAGAGCCGATTGGGTATGCCCAGTCGTACGTTGCTCTTGGAAGCGGGGACGGATGGTGGGAAGAAGAAACCGATCCAGGAGTACGCGGAATAGACCAGTCACTGGCGAATGCATCACAACTGGGCAAAGGCTTGGGAACCAAGCTGGTTCGAGCTCTGGTTGAGTTGCTGTTCAATGATCCCGAGGTCACCAAGATCCAAACGGACCCGTCGCCGAGCAACTTGCGAGCGATCCGATGCTACGAGAAAGCGGGGTTTGAGAGGCAAGGTACCGTAACCACCCCAGATGGTCCAGCCGTGTACATGGTTCAAACACGCCAGGCATTCGAGCGAACACGCAGTGATGCCTAA

>AF263520 Pseudomonas aeruginosa integron In58, partial sequence.

GTGACCAACAGC---AACGATTCCGTCACACTGCGCCTCATGACTGAGCATGACCTTGCGATGCTCTATGAGTGGCTAAATCGATCTCATATCGTCGAGTGGTGGGGCGGAGAAGAAGCACGCCCGACACTTGCTGACGTACAGGAACAGTACTTGCCAAGCGTTTTAGCGCAAGAGTCCGTCACTCCATACATTGCAATGCTGAATGGAGAGCCGATTGGGTATGCCCAGTCGTACGTTGCTCTTGGAAGCGGGGACGGATGGTGGGAAGAAGAAACCGATCCAGGAGTACGCGGAATAGACCAGTCACTGGCGAATGCATCACAACTGGGCAAAGGCTTGGGAACCAAGCTGGTTCGAGCTCTGGTTGAGTTGCTGTTCAATGATCCCGAGGTCACCAAGATCCAAACGGACCCGTCGCCGAGCAACTTGCGAGCGATCCGATGCTACGAGAAAGCGGGGTTTGAGAGGCAAGGTACCGTAACCACCCCAGATGGTCCAGCCGTGTACATGGTTCAAACACGCCAGGCATTCGAGCGAACACGCAGTGATGCCTAA

>AF445082 Acinetobacter baumannii integron class I integrase (intI), carbapenem-hydrolyzing beta-lactamase (blaIMP-4), quaternary ammonium compound resistance protein (qacG), aminoglycoside 6'-N-acetyltransferase (aacA4), and chloramphenicol acetyltransferase variant (catB3) genes, complete cds.

GTGACCAACAGC---AACGATTCCGTCACACTGCGCCTCATGACTGAGCATGACCTTGCGATGCTCTATGAGTGGCTAAATCGATCTCATATCGTCGAGTGGTGGGGCGGAGAAGAAGCACGCCCGACACTTGCTGACGTACAGGAACAGTACTTGCCAAGCGTTTTAGCGCAAGAGTCCGTCACTCCATACATTGCAATGCTGAATGGAGAGCCGATTGGGTATGCCCAGTCGTACGTTGCTCTTGGAAGCGGGGACGGATGGTGGGAAGAAGAAACCGATCCAGGAGTACGCGGAATAGACCAGTCACTGGCGAATGCATCACAACTGGGCAAAGGCTTGGGAACCAAGCTGGTTCGAGCTCTGGTTGAGTTGCTGTTCAATGATCCCGAGGTCACCAAGATCCAAACGGACCCGTCGCCGAGCAACTTGCGAGCGATCCGATGCTACGAGAAAGCGGGGTTTGAGAGGCAAGGTACCGTAACCACCCCAGATGGTCCAGCCGTGTACATGGTTCAAACACGCCAGGCATTCGAGCGAACACGCAGTGATGCCTAA

>AF439785 Campylobacter jejuni isolate B6W4 integron INTI1 IntI1 gene, partial cds; aminoglycoside 6'-N-acetyltransferase (aacA4) gene, aac-(6')-Ib allele, complete cds; and unknown gene.

GTGACCAACAGC---AACGATTCCGTCACACTGCGCCTCATGACTGAGCATGACCTTGCGATGCTCTATGAGTGGCTAAATCGATCTCATATCGTCGAGTGGTGGGGCGGAGAAGAAGCACGCCCGACACTTGCTGACGTACAGGAACAGTACTTGCCAAGCGTTTTAGCGCAAGAGTCCGTCACTCCATACATTGCAATGCTGAATGGAGAGCCGATTGGGTATGCCCAGTCGTACGTTGCTCTTGGAAGCGGGGACGGATGGTGGGAAGAAGAAACCGATCCAGGAGTACGCGGAATAGACCAGTCACTGGCGAATGCATCACAACTGGGCAAAGGCTTGGGAACCAAGCTGGTTCGAGCTCTGGTTGAGTTGCTGTTCAATGATCCCGAGGTCACCAAGATCCAAACGGACCCGTCGCCGAGCAACTTGCGAGCGATCCGATGCTACGAGAAAGCGGGGTTTGAGAGGCAAGGTACCGTAACCACCCCAGATGGTCCAGCCGTGTACATGGTTCAAACACGCCAGGCATTCGAGCGAACACGCAGTGATGCCTAA

>AF439786 Campylobacter jejuni isolate B7W3 integron INTI1 IntI1 gene, partial cds; aminoglycoside 6'-N-acetyltransferase (aacA4) gene, aac-(6')-Ib allele, complete cds; and unknown gene.

GTGACCAACAGC---AACGATTCCGTCACACTGCGCCTCATGACTGAGCATGACCTTGCGATGCTCTATGAGTGGCTAAATCGATCTCATATCGTCGAGTGGTGGGGCGGAGAAGAAGCACGCCCGACACTTGCTGACGTACAGGAACAGTACTTGCCAAGCGTTTTAGCGCAAGAGTCCGTCACTCCATACATTGCAATGCTGAATGGAGAGCCGATTGGGTATGCCCAGTCGTACGTTGCTCTTGGAAGCGGGGACGGATGGTGGGAAGAAGAAACCGATCCAGGAGTACGCGGAATAGACCAGTCACTGGCGAATGCATCACAACTGGGCAAAGGCTTGGGAACCAAGCTGGTTCGAGCTCTGGTTGAGTTGCTGTTCAATGATCCCGAGGTCACCAAGATCCAAACGGACCCGTCGCCGAGCAACTTGCGAGCGATCCGATGCTACGAGAAAGCGGGGTTTGAGAGGCAAGGTACCGTAACCACCCCAGATGGTCCAGCCGTGTACATGGTTCAAACACGCCAGGCATTCGAGCGAACACGCAGTGATGCCTAA

>S49888 aminoglycoside 6'-N-acetyltransferase [Serratia, 45, Genomic, 1169 nt].

GTGACCAACAGC---AACGATTCCGTCACACTGCGCCTCATGACTGAGCATGACCTTGCGATGCTCTATGAGTGGCTAAATCGATCTCATATCGTCGAGTGGTGGGGCGGAGAAGAAGCACGCCCGACACTTGCTGACGTACAGGAACAGTACTTGCCAAGCGTTTTAGCGCAAGAGTCCGTCACTCCATACATTGCAATGCTGAATGGAGAGCCGATTGGGTATGCCCAGTCGTACGTTGCTCTTGGAAGCGGGGACGGATGGTGGGAAGAAGAAACCGATCCAGGAGTACGCGGAATAGACCAGTCACTGGCGAATGCATCACAACTGGGCAAAGGCTTGGGAACCAAGCTGGTTCGAGCTCTGGTTGAGTTGCTGTTCAATGATCCCGAGGTCACCAAGATCCAAACGGACCCGTCGCCGAGCAACTTGCGAGCGATCCGATGCTACGAGAAAGCGGGGTTTGAGAGGCAAGGTACCGTAACCACCCCAGATGGTCCAGCCGTGTACATGGTTCAAACACGCCAGGCATTCGAGCGAACACGCAGTGATGCCTAA

>AY038837 Acinetobacter baumannii type 1 integron IntI gene, partial cds; rifampin ADP-ribosylating transferase (arr-3), aminoglycoside 6'-N-acetyltransferase (aacA4), quaternary ammonium-compound resistance protein (qacEdelta1), and diphydropterorate synthase (sul1) genes, complete cds; and unknown gene.

GTGACCAACAGC---AACGATTCCGTCACACTGCGCCTCATGACTGAGCATGACCTTGCGATGCTCTATGAGTGGCTAAATCGATCTCATATCGTCGAGTGGTGGGGCGGAGAAGAAGCACGCCCGACACTTGCTGACGTACAGGAACAGTACTTGCCAAGCGTTTTAGCGCAAGAGTCCGTCACTCCATACATTGCAATGCTGAATGGAGAGCCGATTGGGTATGCCCAGTCGTACGTTGCTCTTGGAAGCGGGGACGGATGGTGGGAAGAAGAAACCGATCCAGGAGTACGCGGAATAGACCAGTCACTGGCGAATGCATCACAACTGGGCAAAGGCTTGGGAACCAAGCTGGTTCGAGCTCTGGTTGAGTTGCTGTTCAATGATCCCGAGGTCACCAAGATCCAAACGGACCCGTCGCCGAGCAACTTGCGAGCGATCCGATGCTACGAGAAAGCGGGGTTTGAGAGGCAAGGTACCGTAACCACCCCAGATGGTCCAGCCGTGTACATGGTTCAAACACGCCAGGCATTCGAGCGAACACGCAGTGATGCCTAA

>AY139593 Uncultured bacterium plasmid-encoded class I integron gene cassette array aminoglycoside-3'N-acetyltransferase (aacA4) gene, complete cds.

GTGACCAACAGC---AACGATTCCGTCACACTGCGCCTCATGACTGAGCATGACCTTGCGATGCTCTATGAGTGGCTAAATCGATCTCATATCGTCGAGTGGTGGGGCGGAGAAGAAGCACGCCCGACACTTGCTGACGTACAGGAACAGTACTTGCCAAGCGTTTTAGCGCAAGAGTCCGTCACTCCATACATTGCAATGCTGAATGGAGAGCCGATTGGGTATGCCCAGTCGTACGTTGCTCTTGGAAGCGGGGACGGATGGTGGGAAGAAGAAACCGATCCAGGAGTACGCGGAATAGACCAGTCACTGGCGAATGCATCACAACTGGGCAAAGGCTTGGGAACCAAGCTGGTTCGAGCTCTGGTTGAGTTGCTGTTCAATGATCCCGAGGTCACCAAGATCCAAACGGACCCGTCGCCGAGCAACTTGCGAGCGATCCGATGCTACGAGAAAGCGGGGTTTGAGAGGCAAGGTACCGTAACCACCCCAGATGGTCCAGCCGTGTACATGGTTCAAACACGCCAGGCATTCGAGCGAACACGCAGTGATGCCTAA

>AY775051 Pseudomonas aeruginosa class 1 integron In102 integrase (IntI1) gene, partial cds, aminoglycoside (6') N-acetyltransferase (aacA4), aminoglycoside (3') N-acetyltransferase (aacC1), and VIM-2 metallo beta-lactamase (blaVIM-2) genes, complete cds, and unknown gene.

GTGACCAACAGC---AACGATTCCGTCACACTGCGCCTCATGACTGAGCATGACCTTGCGATGCTCTATGAGTGGCTAAATCGATCTCATATCGTCGAGTGGTGGGGCGGAGAAGAAGCACGCCCGACACTTGCTGACGTACAGGAACAGTACTTGCCAAGCGTTTTAGCGCAAGAGTCCGTCACTCCATACATTGCAATGCTGAATGGAGAGCCGATTGGGTATGCCCAGTCGTACGTTGCTCTTGGAAGCGGGGACGGATGGTGGGAAGAAGAAACCGATCCAGGAGTACGCGGAATAGACCAGTCACTGGCGAATGCATCACAACTGGGCAAAGGCTTGGGAACCAAGCTGGTTCGAGCTCTGGTTGAGTTGCTGTTCAATGATCCCGAGGTCACCAAGATCCAAACGGACCCGTCGCCGAGCAACTTGCGAGCGATCCGATGCTACGAGAAAGCGGGGTTTGAGAGGCAAGGTACCGTAACCACCCCAGATGGTCCAGCCGTGTACATGGTTCAAACACGCCAGGCATTCGAGCGAACACGCAGTGATGCCTAA

>AY954726 Pseudomonas aeruginosa transposon class 1 integron IN103 integrase (intI1) gene, partial cds, and 6'-N-aminoglycoside acetyltransferase Il (aacA7), VIM-2 metallo-beta-lactamase (blaVIM-2), and 6'-N-aminoglycoside acetyltransferase Ib' (aacA4) genes, complete cds.

GTGACCAACAGC---AACGATTCCGTCACACTGCGCCTCATGACTGAGCATGACCTTGCGATGCTCTATGAGTGGCTAAATCGATCTCATATCGTCGAGTGGTGGGGCGGAGAAGAAGCACGCCCGACACTTGCTGACGTACAGGAACAGTACTTGCCAAGCGTTTTAGCGCAAGAGTCCGTCACTCCATACATTGCAATGCTGAATGGAGAGCCGATTGGGTATGCCCAGTCGTACGTTGCTCTTGGAAGCGGGGACGGATGGTGGGAAGAAGAAACCGATCCAGGAGTACGCGGAATAGACCAGTCACTGGCGAATGCATCACAACTGGGCAAAGGCTTGGGAACCAAGCTGGTTCGAGCTCTGGTTGAGTTGCTGTTCAATGATCCCGAGGTCACCAAGATCCAAACGGACCCGTCGCCGAGCAACTTGCGAGCGATCCGATGCTACGAGAAAGCGGGGTTTGAGAGGCAAGGTACCGTAACCACCCCAGATGGTCCAGCCGTGTACATGGTTCAAACACGCCAGGCATTCGAGCGAACACGCAGTGATGCCTAA

>AJ609296 Klebsiella pneumoniae intI1 gene (partial), blaIMP-4 gene, qacG2 gene, aacA4 gene and catB3 gene.

GTGACCAACAGC---AACGATTCCGTCACACTGCGCCTCATGACTGAGCATGACCTTGCGATGCTCTATGAGTGGCTAAATCGATCTCATATCGTCGAGTGGTGGGGCGGAGAAGAAGCACGCCCGACACTTGCTGACGTACAGGAACAGTACTTGCCAAGCGTTTTAGCGCAAGAGTCCGTCACTCCATACATTGCAATGCTGAATGGAGAGCCGATTGGGTATGCCCAGTCGTACGTTGCTCTTGGAAGCGGGGACGGATGGTGGGAAGAAGAAACCGATCCAGGAGTACGCGGAATAGACCAGTCACTGGCGAATGCATCACAACTGGGCAAAGGCTTGGGAACCAAGCTGGTTCGAGCTCTGGTTGAGTTGCTGTTCAATGATCCCGAGGTCACCAAGATCCAAACGGACCCGTCGCCGAGCAACTTGCGAGCGATCCGATGCTACGAGAAAGCGGGGTTTGAGAGGCAAGGTACCGTAACCACCCCAGATGGTCCAGCCGTGTACATGGTTCAAACACGCCAGGCATTCGAGCGAACACGCAGTGATGCCTAA

>AJ628135 Pseudomonas aeruginosa Tn5051-like transposon and class 1 integron InPSG, strain MV461.

GTGACCAACAGC---AACGATTCCGTCACACTGCGCCTCATGACTGAGCATGACCTTGCGATGCTCTATGAGTGGCTAAATCGATCTCATATCGTCGAGTGGTGGGGCGGAGAAGAAGCACGCCCGACACTTGCTGACGTACAGGAACAGTACTTGCCAAGCGTTTTAGCGCAAGAGTCCGTCACTCCATACATTGCAATGCTGAATGGAGAGCCGATTGGGTATGCCCAGTCGTACGTTGCTCTTGGAAGCGGGGACGGATGGTGGGAAGAAGAAACCGATCCAGGAGTACGCGGAATAGACCAGTCACTGGCGAATGCATCACAACTGGGCAAAGGCTTGGGAACCAAGCTGGTTCGAGCTCTGGTTGAGTTGCTGTTCAATGATCCCGAGGTCACCAAGATCCAAACGGACCCGTCGCCGAGCAACTTGCGAGCGATCCGATGCTACGAGAAAGCGGGGTTTGAGAGGCAAGGTACCGTAACCACCCCAGATGGTCCAGCCGTGTACATGGTTCAAACACGCCAGGCATTCGAGCGAACACGCAGTGATGCCTAA

>DQ174291 Acinetobacter haemolyticus strain 99-24854 type 1 integron rifampin ADP-ribosylating transferase (arr-3) and aminoglycoside 6'-N-acetyltransferase (aacA4) genes, complete cds.

GTGACCAACAGC---AACGATTCCGTCACACTGCGCCTCATGACTGAGCATGACCTTGCGATGCTCTATGAGTGGCTAAATCGATCTCATATCGTCGAGTGGTGGGGCGGAGAAGAAGCACGCCCGACACTTGCTGACGTACAGGAACAGTACTTGCCAAGCGTTTTAGCGCAAGAGTCCGTCACTCCATACATTGCAATGCTGAATGGAGAGCCGATTGGGTATGCCCAGTCGTACGTTGCTCTTGGAAGCGGGGACGGATGGTGGGAAGAAGAAACCGATCCAGGAGTACGCGGAATAGACCAGTCACTGGCGAATGCATCACAACTGGGCAAAGGCTTGGGAACCAAGCTGGTTCGAGCTCTGGTTGAGTTGCTGTTCAATGATCCCGAGGTCACCAAGATCCAAACGGACCCGTCGCCGAGCAACTTGCGAGCGATCCGATGCTACGAGAAAGCGGGGTTTGAGAGGCAAGGTACCGTAACCACCCCAGATGGTCCAGCCGTGTACATGGTTCAAACACGCCAGGCATTCGAGCGAACACGCAGTGATGCCTAA

>DQ532122 Acinetobacter baumannii strain DB60079/01 class I integron metallo-beta-lactamase IMP-4 (blaIMP-4), quarternary ammonium compound resistance protein (qacG2), aminoglycoside 6'-N-acetyltransferase (aacA4), and chloramphenicol acetyltransferase (catB3) genes, complete cds.

GTGACCAACAGC---AACGATTCCGTCACACTGCGCCTCATGACTGAGCATGACCTTGCGATGCTCTATGAGTGGCTAAATCGATCTCATATCGTCGAGTGGTGGGGCGGAGAAGAAGCACGCCCGACACTTGCTGACGTACAGGAACAGTACTTGCCAAGCGTTTTAGCGCAAGAGTCCGTCACTCCATACATTGCAATGCTGAATGGAGAGCCGATTGGGTATGCCCAGTCGTACGTTGCTCTTGGAAGCGGGGACGGATGGTGGGAAGAAGAAACCGATCCAGGAGTACGCGGAATAGACCAGTCACTGGCGAATGCATCACAACTGGGCAAAGGCTTGGGAACCAAGCTGGTTCGAGCTCTGGTTGAGTTGCTGTTCAATGATCCCGAGGTCACCAAGATCCAAACGGACCCGTCGCCGAGCAACTTGCGAGCGATCCGATGCTACGAGAAAGCGGGGTTTGAGAGGCAAGGTACCGTAACCACCCCAGATGGTCCAGCCGTGTACATGGTTCAAACACGCCAGGCATTCGAGCGAACACGCAGTGATGCCTAA

>DQ984668 Pseudomonas aeruginosa strain PA255 class I integron beta-lactamase Oxa10 (oxa10), aminoglycoside acetyltranferase Aac(6')-Ib (aacA4), and chloramphenicol resistance protein CmlA5 (cmlA5) genes, complete cds.

GTGACCAACAGC---AACGATTCCGTCACACTGCGCCTCATGACTGAGCATGACCTTGCGATGCTCTATGAGTGGCTAAATCGATCTCATATCGTCGAGTGGTGGGGCGGAGAAGAAGCACGCCCGACACTTGCTGACGTACAGGAACAGTACTTGCCAAGCGTTTTAGCGCAAGAGTCCGTCACTCCATACATTGCAATGCTGAATGGAGAGCCGATTGGGTATGCCCAGTCGTACGTTGCTCTTGGAAGCGGGGACGGATGGTGGGAAGAAGAAACCGATCCAGGAGTACGCGGAATAGACCAGTCACTGGCGAATGCATCACAACTGGGCAAAGGCTTGGGAACCAAGCTGGTTCGAGCTCTGGTTGAGTTGCTGTTCAATGATCCCGAGGTCACCAAGATCCAAACGGACCCGTCGCCGAGCAACTTGCGAGCGATCCGATGCTACGAGAAAGCGGGGTTTGAGAGGCAAGGTACCGTAACCACCCCAGATGGTCCAGCCGTGTACATGGTTCAAACACGCCAGGCATTCGAGCGAACACGCAGTGATGCCTAA

>AM412777 Citrobacter freundii partial class 1 integron In14, isolate CF14.

GTGACCAACAGC---AACGATTCCGTCACACTGCGCCTCATGACTGAGCATGACCTTGCGATGCTCTATGAGTGGCTAAATCGATCTCATATCGTCGAGTGGTGGGGCGGAGAAGAAGCACGCCCGACACTTGCTGACGTACAGGAACAGTACTTGCCAAGCGTTTTAGCGCAAGAGTCCGTCACTCCATACATTGCAATGCTGAATGGAGAGCCGATTGGGTATGCCCAGTCGTACGTTGCTCTTGGAAGCGGGGACGGATGGTGGGAAGAAGAAACCGATCCAGGAGTACGCGGAATAGACCAGTCACTGGCGAATGCATCACAACTGGGCAAAGGCTTGGGAACCAAGCTGGTTCGAGCTCTGGTTGAGTTGCTGTTCAATGATCCCGAGGTCACCAAGATCCAAACGGACCCGTCGCCGAGCAACTTGCGAGCGATCCGATGCTACGAGAAAGCGGGGTTTGAGAGGCAAGGTACCGTAACCACCCCAGATGGTCCAGCCGTGTACATGGTTCAAACACGCCAGGCATTCGAGCGAACACGCAGTGATGCCTAA

>DQ767903 Pseudomonas aeruginosa strain PA13 class I integron integrase (intI1) gene, partial cds, and aminoglycoside 6'-N-acetyltransferase (aac'61b) and oxcillinase (oxa) genes, complete cds.

GTGACCAACAGC---AACGATTCCGTCACACTGCGCCTCATGACTGAGCATGACCTTGCGATGCTCTATGAGTGGCTAAATCGATCTCATATCGTCGAGTGGTGGGGCGGAGAAGAAGCACGCCCGACACTTGCTGACGTACAGGAACAGTACTTGCCAAGCGTTTTAGCGCAAGAGTCCGTCACTCCATACATTGCAATGCTGAATGGAGAGCCGATTGGGTATGCCCAGTCGTACGTTGCTCTTGGAAGCGGGGACGGATGGTGGGAAGAAGAAACCGATCCAGGAGTACGCGGAATAGACCAGTCACTGGCGAATGCATCACAACTGGGCAAAGGCTTGGGAACCAAGCTGGTTCGAGCTCTGGTTGAGTTGCTGTTCAATGATCCCGAGGTCACCAAGATCCAAACGGACCCGTCGCCGAGCAACTTGCGAGCGATCCGATGCTACGAGAAAGCGGGGTTTGAGAGGCAAGGTACCGTAACCACCCCAGATGGTCCAGCCGTGTACATGGTTCAAACACGCCAGGCATTCGAGCGAACACGCAGTGATGCCTAA

>AF156486 Klebsiella pneumoniae integron integrase INTI1 (intI1), beta-lactamase GES-1 (ges-1), aminoglycoside 6'-N-acetyltransferase type II AAC6'Ib (aac(6')-Ib'), dihydrofolate reductase DHFRXVb (dhfrXVb), chloramphenicol transporter CMLA4 (cmlA4), and streptomycin 3''-adenylyltransferase AADA2 (aadA2) genes, complete cds.

GTGACCAACAGC---AACGATTCCGTCACACTGCGCCTCATGACTGAGCATGACCTTGCGATGCTCTATGAGTGGCTAAATCGATCTCATATCGTCGAGTGGTGGGGCGGAGAAGAAGCACGCCCGACACTTGCTGACGTACAGGAACAGTACTTGCCAAGCGTTTTAGCGCAAGAGTCCGTCACTCCATACATTGCAATGCTGAATGGAGAGCCGATTGGGTATGCCCAGTCGTACGTTGCTCTTGGAAGCGGGGACGGATGGTGGGAAGAAGAAACCGATCCAGGAGTACGCGGAATAGACCAGTCACTGGCGAATGCATCACAACTGGGCAAAGGCTTGGGAACCAAGCTGGTTCGAGCTCTGGTTGAGTTGCTGTTCAATGATCCCGAGGTCACCAAGATCCAAACGGACCCGTCGCCGAGCAACTTGCGAGCGATCCGATGCTACGAGAAAGCGGGGTTTGAGAGGCAAGGTACCGTAACCACCCCAGATGGTCCAGCCGTGTACATGGTTCAAACACGCCAGGCATTCGAGCGAACACGCAGTGATGCCTAA

>AF202035 Enterobacter cloacae integron In14pr44/16 DNA integrase (int) gene, partial cds; aminoglycoside 6'-N-acetyltransferase (aacC4) gene, complete cds; and quaternary ammonium compound-resistance protein (qacEdelta1) gene, partial cds.

GTGACCAACAGC---AACGATTCCGTCACACTGCGCCTCATGACTGAGCATGACCTTGCGATGCTCTATGAGTGGCTAAATCGATCTCATATCGTCGAGTGGTGGGGCGGAGAAGAAGCACGCCCGACACTTGCTGACGTACAGGAACAGTACTTGCCAAGCGTTTTAGCGCAAGAGTCCGTCACTCCATACATTGCAATGCTGAATGGAGAGCCGATTGGGTATGCCCAGTCGTACGTTGCTCTTGGAAGCGGGGACGGATGGTGGGAAGAAGAAACCGATCCAGGAGTACGCGGAATAGACCAGTCACTGGCGAATGCATCACAACTGGGCAAAGGCTTGGGAACCAAGCTGGTTCGAGCTCTGGTTGAGTTGCTGTTCAATGATCCCGAGGTCACCAAGATCCAAACGGACCCGTCGCCGAGCAACTTGCGAGCGATCCGATGCTACGAGAAAGCGGGGTTTGAGAGGCAAGGTACCGTAACCACCCCAGATGGTCCAGCCGTGTACATGGTTCAAACACGCCAGGCATTCGAGCGAACACGCAGTGATGCCTAA

>AF371964 Burkholderia cepacia class I integron beta-lactamase OXA family and aminoglycoside 6'-N-acetyltransferase type 1b genes, complete cds.

GTGACCAACAGC---AACGATTCCGTCACACTGCGCCTCATGACTGAGCATGACCTTGCGATGCTCTATGAGTGGCTAAATCGATCTCATATCGTCGAGTGGTGGGGCGGAGAAGAAGCACGCCCGACACTTGCTGACGTACAGGAACAGTACTTGCCAAGCGTTTTAGCGCAAGAGTCCGTCACTCCATACATTGCAATGCTGAATGGAGAGCCGATTGGGTATGCCCAGTCGTACGTTGCTCTTGGAAGCGGGGACGGATGGTGGGAAGAAGAAACCGATCCAGGAGTACGCGGAATAGACCAGTCACTGGCGAATGCATCACAACTGGGCAAAGGCTTGGGAACCAAGCTGGTTCGAGCTCTGGTTGAGTTGCTGTTCAATGATCCCGAGGTCACCAAGATCCAAACGGACCCGTCGCCGAGCAACTTGCGAGCGATCCGATGCTACGAGAAAGCGGGGTTTGAGAGGCAAGGTACCGTAACCACCCCAGATGGTCCAGCCGTGTACATGGTTCAAACACGCCAGGCATTCGAGCGAACACGCAGTGATGCCTAA

>PSEAAC6IB Pseudomonas fluorescens aminoglycoside 6'-N-acetyltransferase type Ib (aac(6')-Ib gene, complete cds, and integrase and aadA2 genes, 5' end.

GTGACCAACAGC---AACGATTCCGTCACACTGCGCCTCATGACTGAGCATGACCTTGCGATGCTCTATGAGTGGCTAAATCGATCTCATATCGTCGAGTGGTGGGGCGGAGAAGAAGCACGCCCGACACTTGCTGACGTACAGGAACAGTACTTGCCAAGCGTTTTAGCGCAAGAGTCCGTCACTCCATACATTGCAATGCTGAATGGAGAGCCGATTGGGTATGCCCAGTCGTACGTTGCTCTTGGAAGCGGGGACGGATGGTGGGAAGAAGAAACCGATCCAGGAGTACGCGGAATAGACCAGTCACTGGCGAATGCATCACAACTGGGCAAAGGCTTGGGAACCAAGCTGGTTCGAGCTCTGGTTGAGTTGCTGTTCAATGATCCCGAGGTCACCAAGATCCAAACGGACCCGTCGCCGAGCAACTTGCGAGCGATCCGATGCTACGAGAAAGCGGGGTTTGAGAGGCAAGGTACCGTAACCACCCCAGATGGTCCAGCCGTGTACATGGTTCAAACACGCCAGGCATTCGAGCGAACACGCAGTGATGCCTAA

>PSEAACDINT Pseudomonas fluorescens aminoglycoside 6-N-acetyltransferase (aac(6')-Ib') gene, complete cds; integrase gene, 5' end; aadA2 gene, 5' end.

GTGACCAACAGC---AACGATTCCGTCACACTGCGCCTCATGACTGAGCATGACCTTGCGATGCTCTATGAGTGGCTAAATCGATCTCATATCGTCGAGTGGTGGGGCGGAGAAGAAGCACGCCCGACACTTGCTGACGTACAGGAACAGTACTTGCCAAGCGTTTTAGCGCAAGAGTCCGTCACTCCATACATTGCAATGCTGAATGGAGAGCCGATTGGGTATGCCCAGTCGTACGTTGCTCTTGGAAGCGGGGACGGATGGTGGGAAGAAGAAACCGATCCAGGAGTACGCGGAATAGACCAGTCACTGGCGAATGCATCACAACTGGGCAAAGGCTTGGGAACCAAGCTGGTTCGAGCTCTGGTTGAGTTGCTGTTCAATGATCCCGAGGTCACCAAGATCCAAACGGACCCGTCGCCGAGCAACTTGCGAGCGATCCGATGCTACGAGAAAGCGGGGTTTGAGAGGCAAGGTACCGTAACCACCCCAGATGGTCCAGCCGTGTACATGGTTCAAACACGCCAGGCATTCGAGCGAACACGCAGTGATGCCTAA

>AY139595 Uncultured bacterium plasmid-encoded class I integron gene cassette array quarternary ammonium compound resistance protein (qacH), aminoglycoside-6'N-acetyltransferase (aacA4), and OrfE-like (orfE-like) genes, complete cds.

GTGACCAACAGC---AACGATTCCGTCACACTGCGCCTCATGACTGAGCATGACCTTGCGATGCTCTATGAGTGGCTAAATCGATCTCATATCGTCGAGTGGTGGGGCGGAGAAGAAGCACGCCCGACACTTGCTGACGTACAGGAACAGTACTTGCCAAGCGTTTTAGCGCAAGAGTCCGTCACTCCATACATTGCAATGCTGAATGGAGAGCCGATTGGGTATGCCCAGTCGTACGTTGCTCTTGGAAGCGGGGACGGATGGTGGGAAGAAGAAACCGATCCAGGAGTACGCGGAATAGACCAGTCACTGGCGAATGCATCACAACTGGGCAAAGGCTTGGGAACCAAGCTGGTTCGAGCTCTGGTTGAGTTGCTGTTCAATGATCCCGAGGTCACCAAGATCCAAACGGACCCGTCGCCGAGCAACTTGCGAGCGATCCGATGCTACGAGAAAGCGGGGTTTGAGAGGCAAGGTACCGTAACCACCCCAGATGGTCCAGCCGTGTACATGGTTCAAACACGCCAGGCATTCGAGCGAACACGCAGTGATGCCTAA

>AJ515707 Pseudomonas aeruginosa tnpR gene (partial), urf2 pseudogene, intI1 gene, blaVIM-2 gene, aacA4 gene, qacEdelta1 gene and sul1 gene (partial).

GTGACCAACAGC---AACGATTCCGTCACACTGCGCCTCATGACTGAGCATGACCTTGCGATGCTCTATGAGTGGCTAAATCGATCTCATATCGTCGAGTGGTGGGGCGGAGAAGAAGCACGCCCGACACTTGCTGACGTACAGGAACAGTACTTGCCAAGCGTTTTAGCGCAAGAGTCCGTCACTCCATACATTGCAATGCTGAATGGAGAGCCGATTGGGTATGCCCAGTCGTACGTTGCTCTTGGAAGCGGGGACGGATGGTGGGAAGAAGAAACCGATCCAGGAGTACGCGGAATAGACCAGTCACTGGCGAATGCATCACAACTGGGCAAAGGCTTGGGAACCAAGCTGGTTCGAGCTCTGGTTGAGTTGCTGTTCAATGATCCCGAGGTCACCAAGATCCAAACGGACCCGTCGCCGAGCAACTTGCGAGCGATCCGATGCTACGAGAAAGCGGGGTTTGAGAGGCAAGGTACCGTAACCACCCCAGATGGTCCAGCCGTGTACATGGTTCAAACACGCCAGGCATTCGAGCGAACACGCAGTGATGCCTAA

>AY294333 Pseudomonas aeruginosa strain MB-7 class I integron, partial sequence.

GTGACCAACAGC---AACGATTCCGTCACACTGCGCCTCATGACTGAGCATGACCTTGCGATGCTCTATGAGTGGCTAAATCGATCTCATATCGTCGAGTGGTGGGGCGGAGAAGAAGCACGCCCGACACTTGCTGACGTACAGGAACAGTACTTGCCAAGCGTTTTAGCGCAAGAGTCCGTCACTCCATACATTGCAATGCTGAATGGAGAGCCGATTGGGTATGCCCAGTCGTACGTTGCTCTTGGAAGCGGGGACGGATGGTGGGAAGAAGAAACCGATCCAGGAGTACGCGGAATAGACCAGTCACTGGCGAATGCATCACAACTGGGCAAAGGCTTGGGAACCAAGCTGGTTCGAGCTCTGGTTGAGTTGCTGTTCAATGATCCCGAGGTCACCAAGATCCAAACGGACCCGTCGCCGAGCAACTTGCGAGCGATCCGATGCTACGAGAAAGCGGGGTTTGAGAGGCAAGGTACCGTAACCACCCCAGATGGTCCAGCCGTGTACATGGTTCAAACACGCCAGGCATTCGAGCGAACACGCAGTGATGCCTAA

>AJ744860 uncultured bacterium pTB11 plasmid complete genome.

GTGACCAACAGC---AACGATTCCGTCACACTGCGCCTCATGACTGAGCATGACCTTGCGATGCTCTATGAGTGGCTAAATCGATCTCATATCGTCGAGTGGTGGGGCGGAGAAGAAGCACGCCCGACACTTGCTGACGTACAGGAACAGTACTTGCCAAGCGTTTTAGCGCAAGAGTCCGTCACTCCATACATTGCAATGCTGAATGGAGAGCCGATTGGGTATGCCCAGTCGTACGTTGCTCTTGGAAGCGGGGACGGATGGTGGGAAGAAGAAACCGATCCAGGAGTACGCGGAATAGACCAGTCACTGGCGAATGCATCACAACTGGGCAAAGGCTTGGGAACCAAGCTGGTTCGAGCTCTGGTTGAGTTGCTGTTCAATGATCCCGAGGTCACCAAGATCCAAACGGACCCGTCGCCGAGCAACTTGCGAGCGATCCGATGCTACGAGAAAGCGGGGTTTGAGAGGCAAGGTACCGTAACCACCCCAGATGGTCCAGCCGTGTACATGGTTCAAACACGCCAGGCATTCGAGCGAACACGCAGTGATGCCTAA

>AY033653 Pseudomonas aeruginosa isolate 96 plasmid pOZ176 class I integron DNA integrase (intI1) gene, partial cds, and aminoglycoside 6'-N-acetyltransferase (aacA4), IMP-9 metallo-beta-lactamase (blaIMP-9), aminoglycoside N (6')-acetyltransferase (aacA4), chloramphenicol acetyltransferase variant (catB8), and OXA-10 beta-lactamase (blaOXA-10) genes, complete cds.

GTGACCAACAGC---AACGATTCCGTCACACTGCGCCTCATGACTGAGCATGACCTTGCGATGCTCTATGAGTGGCTAAATCGATCTCATATCGTCGAGTGGTGGGGCGGAGAAGAAGCACGCCCGACACTTGCTGACGTACAGGAACAGTACTTGCCAAGCGTTTTAGCGCAAGAGTCCGTCACTCCATACATTGCAATGCTGAATGGAGAGCCGATTGGGTATGCCCAGTCGTACGTTGCTCTTGGAAGCGGGGACGGATGGTGGGAAGAAGAAACCGATCCAGGAGTACGCGGAATAGACCAGTCACTGGCGAATGCATCACAACTGGGCAAAGGCTTGGGAACCAAGCTGGTTCGAGCTCTGGTTGAGTTGCTGTTCAATGATCCCGAGGTCACCAAGATCCAAACGGACCCGTCGCCGAGCAACTTGCGAGCGATCCGATGCTACGAGAAAGCGGGGTTTGAGAGGCAAGGTACCGTAACCACCCCAGATGGTCCAGCCGTGTACATGGTTCAAACACGCCAGGCATTCGAGCGAACACGCAGTGATGCCTAA

>AY033653 Pseudomonas aeruginosa isolate 96 plasmid pOZ176 class I integron DNA integrase (intI1) gene, partial cds, and aminoglycoside 6'-N-acetyltransferase (aacA4), IMP-9 metallo-beta-lactamase (blaIMP-9), aminoglycoside N (6')-acetyltransferase (aacA4), chloramphenicol acetyltransferase variant (catB8), and OXA-10 beta-lactamase (blaOXA-10) genes, complete cds.

GTGACCAACAGC---AACGATTCCGTCACACTGCGCCTCATGACTGAGCATGACCTTGCGATGCTCTATGAGTGGCTAAATCGATCTCATATCGTCGAGTGGTGGGGCGGAGAAGAAGCACGCCCGACACTTGCTGACGTACAGGAACAGTACTTGCCAAGCGTTTTAGCGCAAGAGTCCGTCACTCCATACATTGCAATGCTGAATGGAGAGCCGATTGGGTATGCCCAGTCGTACGTTGCTCTTGGAAGCGGGGACGGATGGTGGGAAGAAGAAACCGATCCAGGAGTACGCGGAATAGACCAGTCACTGGCGAATGCATCACAACTGGGCAAAGGCTTGGGAACCAAGCTGGTTCGAGCTCTGGTTGAGTTGCTGTTCAATGATCCCGAGGTCACCAAGATCCAAACGGACCCGTCGCCGAGCAACTTGCGAGCGATCCGATGCTACGAGAAAGCGGGGTTTGAGAGGCAAGGTACCGTAACCACCCCAGATGGTCCAGCCGTGTACATGGTTCAAACACGCCAGGCATTCGAGCGAACACGCAGTGATGCCTAA

>DQ321671 Shigella sonnei plasmid pSS2852 multidrug-resistance class 1 integron, complete sequence.

GTGACCAACAGC---AACGATTCCGTCACACTGCGCCTCATGACTGAGCATGACCTTGCGATGCTCTATGAGTGGCTAAATCGATCTCATATCGTCGAGTGGTGGGGCGGAGAAGAAGCACGCCCGACACTTGCTGACGTACAGGAACAGTACTTGCCAAGCGTTTTAGCGCAAGAGTCCGTCACTCCATACATTGCAATGCTGAATGGAGAGCCGATTGGGTATGCCCAGTCGTACGTTGCTCTTGGAAGCGGGGACGGATGGTGGGAAGAAGAAACCGATCCAGGAGTACGCGGAATAGACCAGTCACTGGCGAATGCATCACAACTGGGCAAAGGCTTGGGAACCAAGCTGGTTCGAGCTCTGGTTGAGTTGCTGTTCAATGATCCCGAGGTCACCAAGATCCAAACGGACCCGTCGCCGAGCAACTTGCGAGCGATCCGATGCTACGAGAAAGCGGGGTTTGAGAGGCAAGGTACCGTAACCACCCCAGATGGTCCAGCCGTGTACATGGTTCAAACACGCCAGGCATTCGAGCGAACACGCAGTGATGCCTAA

>AM087408 Pseudomonas aeruginosa class 1 integron containing blaVIM-2 and aacA4 gene cassettes.

GTGACCAACAGC---AACGATTCCGTCACACTGCGCCTCATGACTGAGCATGACCTTGCGATGCTCTATGAGTGGCTAAATCGATCTCATATCGTCGAGTGGTGGGGCGGAGAAGAAGCACGCCCGACACTTGCTGACGTACAGGAACAGTACTTGCCAAGCGTTTTAGCGCAAGAGTCCGTCACTCCATACATTGCAATGCTGAATGGAGAGCCGATTGGGTATGCCCAGTCGTACGTTGCTCTTGGAAGCGGGGACGGATGGTGGGAAGAAGAAACCGATCCAGGAGTACGCGGAATAGACCAGTCACTGGCGAATGCATCACAACTGGGCAAAGGCTTGGGAACCAAGCTGGTTCGAGCTCTGGTTGAGTTGCTGTTCAATGATCCCGAGGTCACCAAGATCCAAACGGACCCGTCGCCGAGCAACTTGCGAGCGATCCGATGCTACGAGAAAGCGGGGTTTGAGAGGCAAGGTACCGTAACCACCCCAGATGGTCCAGCCGTGTACATGGTTCAAACACGCCAGGCATTCGAGCGAACACGCAGTGATGCCTAA

>AM087404 Pseudomonas aeruginosa class 1 integron containing aacA4 and blaVIM-2 gene cassettes.

GTGACCAACAGC---AACGATTCCGTCACACTGCGCCTCATGACTGAGCATGACCTTGCGATGCTCTATGAGTGGCTAAATCGATCTCATATCGTCGAGTGGTGGGGCGGAGAAGAAGCACGCCCGACACTTGCTGACGTACAGGAACAGTACTTGCCAAGCGTTTTAGCGCAAGAGTCCGTCACTCCATACATTGCAATGCTGAATGGAGAGCCGATTGGGTATGCCCAGTCGTACGTTGCTCTTGGAAGCGGGGACGGATGGTGGGAAGAAGAAACCGATCCAGGAGTACGCGGAATAGACCAGTCACTGGCGAATGCATCACAACTGGGCAAAGGCTTGGGAACCAAGCTGGTTCGAGCTCTGGTTGAGTTGCTGTTCAATGATCCCGAGGTCACCAAGATCCAAACGGACCCGTCGCCGAGCAACTTGCGAGCGATCCGATGCTACGAGAAAGCGGGGTTTGAGAGGCAAGGTACCGTAACCACCCCAGATGGTCCAGCCGTGTACATGGTTCAAACACGCCAGGCATTCGAGCGAACACGCAGTGATGCCTAA

>DQ236170 Pseudomonas aeruginosa PS1 class I integron IntI1 (intI1) gene, partial cds, GES-1 extended-spectrum beta-lactamase (blaGES-1) and aminoglycoside 6'-N-acetyltransferase (aacA4) genes, complete cds, and unknown gene.

GTGACCAACAGC---AACGATTCCGTCACACTGCGCCTCATGACTGAGCATGACCTTGCGATGCTCTATGAGTGGCTAAATCGATCTCATATCGTCGAGTGGTGGGGCGGAGAAGAAGCACGCCCGACACTTGCTGACGTACAGGAACAGTACTTGCCAAGCGTTTTAGCGCAAGAGTCCGTCACTCCATACATTGCAATGCTGAATGGAGAGCCGATTGGGTATGCCCAGTCGTACGTTGCTCTTGGAAGCGGGGACGGATGGTGGGAAGAAGAAACCGATCCAGGAGTACGCGGAATAGACCAGTCACTGGCGAATGCATCACAACTGGGCAAAGGCTTGGGAACCAAGCTGGTTCGAGCTCTGGTTGAGTTGCTGTTCAATGATCCCGAGGTCACCAAGATCCAAACGGACCCGTCGCCGAGCAACTTGCGAGCGATCCGATGCTACGAGAAAGCGGGGTTTGAGAGGCAAGGTACCGTAACCACCCCAGATGGTCCAGCCGTGTACATGGTTCAAACACGCCAGGCATTCGAGCGAACACGCAGTGATGCCTAA

>DQ236171 Pseudomonas aeruginosa PS37 class I integron GES-5 extended-spectrum beta-lactamase (blaGES-5) and aminoglycoside 6'-N-acetyltransferase (aacA4) genes, complete cds.

GTGACCAACAGC---AACGATTCCGTCACACTGCGCCTCATGACTGAGCATGACCTTGCGATGCTCTATGAGTGGCTAAATCGATCTCATATCGTCGAGTGGTGGGGCGGAGAAGAAGCACGCCCGACACTTGCTGACGTACAGGAACAGTACTTGCCAAGCGTTTTAGCGCAAGAGTCCGTCACTCCATACATTGCAATGCTGAATGGAGAGCCGATTGGGTATGCCCAGTCGTACGTTGCTCTTGGAAGCGGGGACGGATGGTGGGAAGAAGAAACCGATCCAGGAGTACGCGGAATAGACCAGTCACTGGCGAATGCATCACAACTGGGCAAAGGCTTGGGAACCAAGCTGGTTCGAGCTCTGGTTGAGTTGCTGTTCAATGATCCCGAGGTCACCAAGATCCAAACGGACCCGTCGCCGAGCAACTTGCGAGCGATCCGATGCTACGAGAAAGCGGGGTTTGAGAGGCAAGGTACCGTAACCACCCCAGATGGTCCAGCCGTGTACATGGTTCAAACACGCCAGGCATTCGAGCGAACACGCAGTGATGCCTAA

>DQ522234 Pseudomonas aeruginosa class I integron DNA integrase (intI1) gene, partial cds, aminoglycoside 2'-O-adenylyltransferase (aadB), mettalo-beta-lactamase VIM-2 (blaVIM-2), and aminoglycoside 6'-acetyltransferase (aacA4) genes, complete cds, and QacEdelta1 multidrug exporter (qacEdelta1) gene, partial cds.

GTGACCAACAGC---AACGATTCCGTCACACTGCGCCTCATGACTGAGCATGACCTTGCGATGCTCTATGAGTGGCTAAATCGATCTCATATCGTCGAGTGGTGGGGCGGAGAAGAAGCACGCCCGACACTTGCTGACGTACAGGAACAGTACTTGCCAAGCGTTTTAGCGCAAGAGTCCGTCACTCCATACATTGCAATGCTGAATGGAGAGCCGATTGGGTATGCCCAGTCGTACGTTGCTCTTGGAAGCGGGGACGGATGGTGGGAAGAAGAAACCGATCCAGGAGTACGCGGAATAGACCAGTCACTGGCGAATGCATCACAACTGGGCAAAGGCTTGGGAACCAAGCTGGTTCGAGCTCTGGTTGAGTTGCTGTTCAATGATCCCGAGGTCACCAAGATCCAAACGGACCCGTCGCCGAGCAACTTGCGAGCGATCCGATGCTACGAGAAAGCGGGGTTTGAGAGGCAAGGTACCGTAACCACCCCAGATGGTCCAGCCGTGTACATGGTTCAAACACGCCAGGCATTCGAGCGAACACGCAGTGATGCCTAA

>EF125009 Acinetobacter baumannii strain YMC 05/10/R155-VIM-2 class I integron, partial sequence.

GTGACCAACAGC---AACGATTCCGTCACACTGCGCCTCATGACTGAGCATGACCTTGCGATGCTCTATGAGTGGCTAAATCGATCTCATATCGTCGAGTGGTGGGGCGGAGAAGAAGCACGCCCGACACTTGCTGACGTACAGGAACAGTACTTGCCAAGCGTTTTAGCGCAAGAGTCCGTCACTCCATACATTGCAATGCTGAATGGAGAGCCGATTGGGTATGCCCAGTCGTACGTTGCTCTTGGAAGCGGGGACGGATGGTGGGAAGAAGAAACCGATCCAGGAGTACGCGGAATAGACCAGTCACTGGCGAATGCATCACAACTGGGCAAAGGCTTGGGAACCAAGCTGGTTCGAGCTCTGGTTGAGTTGCTGTTCAATGATCCCGAGGTCACCAAGATCCAAACGGACCCGTCGCCGAGCAACTTGCGAGCGATCCGATGCTACGAGAAAGCGGGGTTTGAGAGGCAAGGTACCGTAACCACCCCAGATGGTCCAGCCGTGTACATGGTTCAAACACGCCAGGCATTCGAGCGAACACGCAGTGATGCCTAA

>AM180753 Pseudomonas aeruginosa In71 integron.

GTGACCAACAGC---AACGATTCCGTCACACTGCGCCTCATGACTGAGCATGACCTTGCGATGCTCTATGAGTGGCTAAATCGATCTCATATCGTCGAGTGGTGGGGCGGAGAAGAAGCACGCCCGACACTTGCTGACGTACAGGAACAGTACTTGCCAAGCGTTTTAGCGCAAGAGTCCGTCACTCCATACATTGCAATGCTGAATGGAGAGCCGATTGGGTATGCCCAGTCGTACGTTGCTCTTGGAAGCGGGGACGGATGGTGGGAAGAAGAAACCGATCCAGGAGTACGCGGAATAGACCAGTCACTGGCGAATGCATCACAACTGGGCAAAGGCTTGGGAACCAAGCTGGTTCGAGCTCTGGTTGAGTTGCTGTTCAATGATCCCGAGGTCACCAAGATCCAAACGGACCCGTCGCCGAGCAACTTGCGAGCGATCCGATGCTACGAGAAAGCGGGGTTTGAGAGGCAAGGTACCGTAACCACCCCAGATGGTCCAGCCGTGTACATGGTTCAAACACGCCAGGCATTCGAGCGAACACGCAGTGATGCCTAA

>AY648351 Klebsiella pneumoniae plasmid pJHCMW1 mutant aminoglycoside 6'-N-acetyltransferase-IbS124A gene, complete cds.

GTGACCAACAGC---AACGATTCCGTCACACTGCGCCTCATGACTGAGCATGACCTTGCGATGCTCTATGAGTGGCTAAATCGATCTCATATCGTCGAGTGGTGGGGCGGAGAAGAAGCACGCCCGACACTTGCTGACGTACAGGAACAGTACTTGCCAAGCGTTTTAGCGCAAGAGTCCGTCACTCCATACATTGCAATGCTGAATGGAGAGCCGATTGGGTATGCCCAGTCGTACGTTGCTCTTGGAAGCGGGGACGGATGGTGGGAAGAAGAAACCGATCCAGGAGTACGCGGAATAGACCAGTTACTGGCGAATGCAGCACAACTGGGCAAAGGCTTGGGAACCAAGCTGGTTCGAGCTCTGGTTGAGTTGCTGTTCAATGATCCCGAGGTCACCAAGATCCAAACGGACCCGTCGCCGAGCAACTTGCGAGCGATCCGATGCTACGAGAAAGCGGGGTTTGAGAGGCAAGGTACCGTAACCACCCCAGATGGTCCAGCCGTGTACATGGTTCAAACACGCCAGGCATTCGAGCGAACACGCAGTGTTGCCTAA

>AF360376 Klebsiella pneumoniae plasmid pJHC-MW1 mutant AAC6'-IbS156A 6'-N-acetyltransferase gene, complete cds.

GTGACCAACAGC---AACGATTCCGTCACACTGCGCCTCATGACTGAGCATGACCTTGCGATGCTCTATGAGTGGCTAAATCGATCTCATATCGTCGAGTGGTGGGGCGGAGAAGAAGCACGCCCGACACTTGCTGACGTACAGGAACAGTACTTGCCAAGCGTTTTAGCGCAAGAGTCCGTCACTCCATACATTGCAATGCTGAATGGAGAGCCGATTGGGTATGCCCAGTCGTACGTTGCTCTTGGAAGCGGGGACGGATGGTGGGAAGAAGAAACCGATCCAGGAGTACGCGGAATAGACCAGTTACTGGCGAATGCATCACAACTGGGCAAAGGCTTGGGAACCAAGCTGGTTCGAGCTCTGGTTGAGTTGCTGTTCAATGATCCCGAGGTCACCAAGATCCAAACGGACCCGGCACCGAGCAACTTGCGAGCGATCCGATGCTACGAGAAAGCGGGGTTTGAGAGGCAAGGTACCGTAACCACCCCAGATGGTCCAGCCGTGTACATGGTTCAAACACGCCAGGCATTCGAGCGAACACGCAGTGTTGCCTAA

>AF360378 Klebsiella pneumoniae plasmid pJHC-MW1 mutant AAC6'-IbS158A 6'-N-acetyltransferase gene, complete cds.

GTGACCAACAGC---AACGATTCCGTCACACTGCGCCTCATGACTGAGCATGACCTTGCGATGCTCTATGAGTGGCTAAATCGATCTCATATCGTCGAGTGGTGGGGCGGAGAAGAAGCACGCCCGACACTTGCTGACGTACAGGAACAGTACTTGCCAAGCGTTTTAGCGCAAGAGTCCGTCACTCCATACATTGCAATGCTGAATGGAGAGCCGATTGGGTATGCCCAGTCGTACGTTGCTCTTGGAAGCGGGGACGGATGGTGGGAAGAAGAAACCGATCCAGGAGTACGCGGAATAGACCAGTTACTGGCGAATGCATCACAACTGGGCAAAGGCTTGGGAACCAAGCTGGTTCGAGCTCTGGTTGAGTTGCTGTTCAATGATCCCGAGGTCACCAAGATCCAAACGGACCCGTCGCCGGCAAACTTGCGAGCGATCCGATGCTACGAGAAAGCGGGGTTTGAGAGGCAAGGTACCGTAACCACCCCAGATGGTCCAGCCGTGTACATGGTTCAAACACGCCAGGCATTCGAGCGAACACGCAGTGTTGCCTAA

>SMAACTYLA S.marcescens Plasmid pAZ007 acetyltransferase gene.

GTGACCAACAGC---AACGATTCCGTCACACTGCGCCTCATGACTGAGCATGACCTTGCGATGCTCTATGAGTGGCTAAATCGATCTCATATCGTCGAGTGGTGGGGCGGAGAAGAAGCACGCCCGACACTTGCTGACGTACAGGAACAGTACTTGCCAAGCGTTTTAGCGCAAGAGTCCGTCACTCCATACATTGCAATGCTGAATGGAGAGCCGATTGGGTATGCCCAGTCGTACGTTGCTCTTGGAAGCGGGGACGGATGGTGGGAAGAAGAAACCGATCCAGGAGTACGCGGAATAGACCAGTTACTGGCGAATGCATCACAACTGGGCAAAGGCTTGGGAACCAAGCTGGTTCGAGCTCTGGTTGAGTTGCTGTTCAATGATCCCGAGGTCACCAAGATCCAAACGGACCCGTCGCCGAGCAACTTGCGAGCGATCCGATGCTACGAGAAAGCGGGGTTTGAGAGGCAAGGTACCGTAACCACCCCAGATGGTCCAGCCGTGTACATGGTTCAAACACGCCAGGCATTCGAGCGAACACGCAGGTTTGCCTAA

>AY648359 Klebsiella pneumoniae plasmid pJHCMW1 mutant aminoglycoside 6'-N-acetyltransferase-IbT132A gene, complete cds.

GTGACCAACAGC---AACGATTCCGTCACACTGCGCCTCATGACTGAGCATGACCTTGCGATGCTCTATGAGTGGCTAAATCGATCTCATATCGTCGAGTGGTGGGGCGGAGAAGAAGCACGCCCGACACTTGCTGACGTACAGGAACAGTACTTGCCAAGCGTTTTAGCGCAAGAGTCCGTCACTCCATACATTGCAATGCTGAATGGAGAGCCGATTGGGTATGCCCAGTCGTACGTTGCTCTTGGAAGCGGGGACGGATGGTGGGAAGAAGAAACCGATCCAGGAGTACGCGGAATAGACCAGTTACTGGCGAATGCATCACAACTGGGCAAAGGCTTGGGAGCCAAGCTGGTTCGAGCTCTGGTTGAGTTGCTGTTCAATGATCCCGAGGTCACCAAGATCCAAACGGACCCGTCGCCGAGCAACTTGCGAGCGATCCGATGCTACGAGAAAGCGGGGTTTGAGAGGCAAGGTACCGTAACCACCCCAGATGGTCCAGCCGTGTACATGGTTCAAACACGCCAGGCATTCGAGCGAACACGCAGTGTTGCCTAA

>AY648375 Klebsiella pneumoniae plasmid pJHCMW1 mutant aminoglycoside 6'-N-acetyltransferase-IbT149A gene, complete cds.

GTGACCAACAGC---AACGATTCCGTCACACTGCGCCTCATGACTGAGCATGACCTTGCGATGCTCTATGAGTGGCTAAATCGATCTCATATCGTCGAGTGGTGGGGCGGAGAAGAAGCACGCCCGACACTTGCTGACGTACAGGAACAGTACTTGCCAAGCGTTTTAGCGCAAGAGTCCGTCACTCCATACATTGCAATGCTGAATGGAGAGCCGATTGGGTATGCCCAGTCGTACGTTGCTCTTGGAAGCGGGGACGGATGGTGGGAAGAAGAAACCGATCCAGGAGTACGCGGAATAGACCAGTTACTGGCGAATGCATCACAACTGGGCAAAGGCTTGGGAACCAAGCTGGTTCGAGCTCTGGTTGAGTTGCTGTTCAATGATCCCGAGGTCGCCAAGATCCAAACGGACCCGTCGCCGAGCAACTTGCGAGCGATCCGATGCTACGAGAAAGCGGGGTTTGAGAGGCAAGGTACCGTAACCACCCCAGATGGTCCAGCCGTGTACATGGTTCAAACACGCCAGGCATTCGAGCGAACACGCAGTGTTGCCTAA

>AY648366 Klebsiella pneumoniae plasmid pJHCMW1 mutant aminoglycoside 6'-N-acetyltransferase-IbE140A gene, complete cds.

GTGACCAACAGC---AACGATTCCGTCACACTGCGCCTCATGACTGAGCATGACCTTGCGATGCTCTATGAGTGGCTAAATCGATCTCATATCGTCGAGTGGTGGGGCGGAGAAGAAGCACGCCCGACACTTGCTGACGTACAGGAACAGTACTTGCCAAGCGTTTTAGCGCAAGAGTCCGTCACTCCATACATTGCAATGCTGAATGGAGAGCCGATTGGGTATGCCCAGTCGTACGTTGCTCTTGGAAGCGGGGACGGATGGTGGGAAGAAGAAACCGATCCAGGAGTACGCGGAATAGACCAGTTACTGGCGAATGCATCACAACTGGGCAAAGGCTTGGGAACCAAGCTGGTTCGAGCTCTGGTTGCGTTGCTGTTCAATGATCCCGAGGTCACCAAGATCCAAACGGACCCGTCGCCGAGCAACTTGCGAGCGATCCGATGCTACGAGAAAGCGGGGTTTGAGAGGCAAGGTACCGTAACCACCCCAGATGGTCCAGCCGTGTACATGGTTCAAACACGCCAGGCATTCGAGCGAACACGCAGTGTTGCCTAA

>AY648373 Klebsiella pneumoniae plasmid pJHCMW1 mutant aminoglycoside 6'-N-acetyltransferase-IbE147A gene, complete cds.

GTGACCAACAGC---AACGATTCCGTCACACTGCGCCTCATGACTGAGCATGACCTTGCGATGCTCTATGAGTGGCTAAATCGATCTCATATCGTCGAGTGGTGGGGCGGAGAAGAAGCACGCCCGACACTTGCTGACGTACAGGAACAGTACTTGCCAAGCGTTTTAGCGCAAGAGTCCGTCACTCCATACATTGCAATGCTGAATGGAGAGCCGATTGGGTATGCCCAGTCGTACGTTGCTCTTGGAAGCGGGGACGGATGGTGGGAAGAAGAAACCGATCCAGGAGTACGCGGAATAGACCAGTTACTGGCGAATGCATCACAACTGGGCAAAGGCTTGGGAACCAAGCTGGTTCGAGCTCTGGTTGAGTTGCTGTTCAATGATCCCGCGGTCACCAAGATCCAAACGGACCCGTCGCCGAGCAACTTGCGAGCGATCCGATGCTACGAGAAAGCGGGGTTTGAGAGGCAAGGTACCGTAACCACCCCAGATGGTCCAGCCGTGTACATGGTTCAAACACGCCAGGCATTCGAGCGAACACGCAGTGTTGCCTAA

>AF360390 Klebsiella pneumoniae plasmid pJHC-MW1 mutant AAC6'-IbE172A 6'-N-acetyltransferase gene, complete cds.

GTGACCAACAGC---AACGATTCCGTCACACTGCGCCTCATGACTGAGCATGACCTTGCGATGCTCTATGAGTGGCTAAATCGATCTCATATCGTCGAGTGGTGGGGCGGAGAAGAAGCACGCCCGACACTTGCTGACGTACAGGAACAGTACTTGCCAAGCGTTTTAGCGCAAGAGTCCGTCACTCCATACATTGCAATGCTGAATGGAGAGCCGATTGGGTATGCCCAGTCGTACGTTGCTCTTGGAAGCGGGGACGGATGGTGGGAAGAAGAAACCGATCCAGGAGTACGCGGAATAGACCAGTTACTGGCGAATGCATCACAACTGGGCAAAGGCTTGGGAACCAAGCTGGTTCGAGCTCTGGTTGAGTTGCTGTTCAATGATCCCGAGGTCACCAAGATCCAAACGGACCCGTCGCCGAGCAACTTGCGAGCGATCCGATGCTACGAGAAAGCGGGGTTTGCAAGGCAAGGTACCGTAACCACCCCAGATGGTCCAGCCGTGTACATGGTTCAAACACGCCAGGCATTCGAGCGAACACGCAGTGTTGCCTAA

>AF360386 Klebsiella pneumoniae plasmid pJHC-MW1 mutant AAC6'-IbE167A 6'-N-acetyltransferase gene, complete cds.

GTGACCAACAGC---AACGATTCCGTCACACTGCGCCTCATGACTGAGCATGACCTTGCGATGCTCTATGAGTGGCTAAATCGATCTCATATCGTCGAGTGGTGGGGCGGAGAAGAAGCACGCCCGACACTTGCTGACGTACAGGAACAGTACTTGCCAAGCGTTTTAGCGCAAGAGTCCGTCACTCCATACATTGCAATGCTGAATGGAGAGCCGATTGGGTATGCCCAGTCGTACGTTGCTCTTGGAAGCGGGGACGGATGGTGGGAAGAAGAAACCGATCCAGGAGTACGCGGAATAGACCAGTTACTGGCGAATGCATCACAACTGGGCAAAGGCTTGGGAACCAAGCTGGTTCGAGCTCTGGTTGAGTTGCTGTTCAATGATCCCGAGGTCACCAAGATCCAAACGGACCCGTCGCCGAGCAACTTGCGAGCGATCCGATGCTACGCGAAAGCGGGGTTTGAGAGGCAAGGTACCGTAACCACCCCAGATGGTCCAGCCGTGTACATGGTTCAAACACGCCAGGCATTCGAGCGAACACGCAGTGTTGCCTAA

>AY648378 Klebsiella pneumoniae plasmid pJHCMW1 mutant aminoglycoside 6'-N-acetyltransferase-IbQ152A gene, complete cds.

GTGACCAACAGC---AACGATTCCGTCACACTGCGCCTCATGACTGAGCATGACCTTGCGATGCTCTATGAGTGGCTAAATCGATCTCATATCGTCGAGTGGTGGGGCGGAGAAGAAGCACGCCCGACACTTGCTGACGTACAGGAACAGTACTTGCCAAGCGTTTTAGCGCAAGAGTCCGTCACTCCATACATTGCAATGCTGAATGGAGAGCCGATTGGGTATGCCCAGTCGTACGTTGCTCTTGGAAGCGGGGACGGATGGTGGGAAGAAGAAACCGATCCAGGAGTACGCGGAATAGACCAGTTACTGGCGAATGCATCACAACTGGGCAAAGGCTTGGGAACCAAGCTGGTTCGAGCTCTGGTTGAGTTGCTGTTCAATGATCCCGAGGTCACCAAGATCGCAACGGACCCGTCGCCGAGCAACTTGCGAGCGATCCGATGCTACGAGAAAGCGGGGTTTGAGAGGCAAGGTACCGTAACCACCCCAGATGGTCCAGCCGTGTACATGGTTCAAACACGCCAGGCATTCGAGCGAACACGCAGTGTTGCCTAA

>AY648347 Klebsiella pneumoniae plasmid pJHCMW1 mutant aminoglycoside 6'-N-acetyltransferase-IbQ118A gene, complete cds.

GTGACCAACAGC---AACGATTCCGTCACACTGCGCCTCATGACTGAGCATGACCTTGCGATGCTCTATGAGTGGCTAAATCGATCTCATATCGTCGAGTGGTGGGGCGGAGAAGAAGCACGCCCGACACTTGCTGACGTACAGGAACAGTACTTGCCAAGCGTTTTAGCGCAAGAGTCCGTCACTCCATACATTGCAATGCTGAATGGAGAGCCGATTGGGTATGCCCAGTCGTACGTTGCTCTTGGAAGCGGGGACGGATGGTGGGAAGAAGAAACCGATCCAGGAGTACGCGGAATAGACGCCTTACTGGCGAATGCATCACAACTGGGCAAAGGCTTGGGAACCAAGCTGGTTCGAGCTCTGGTTGAGTTGCTGTTCAATGATCCCGAGGTCACCAAGATCCAAACGGACCCGTCGCCGAGCAACTTGCGAGCGATCCGATGCTACGAGAAAGCGGGGTTTGAGAGGCAAGGTACCGTAACCACCCCAGATGGTCCAGCCGTGTACATGGTTCAAACACGCCAGGCATTCGAGCGAACACGCAGTGTTGCCTAA

>AY648352 Klebsiella pneumoniae plasmid pJHCMW1 mutant aminoglycoside 6'-N-acetyltransferase-IbQ125A gene, complete cds.

GTGACCAACAGC---AACGATTCCGTCACACTGCGCCTCATGACTGAGCATGACCTTGCGATGCTCTATGAGTGGCTAAATCGATCTCATATCGTCGAGTGGTGGGGCGGAGAAGAAGCACGCCCGACACTTGCTGACGTACAGGAACAGTACTTGCCAAGCGTTTTAGCGCAAGAGTCCGTCACTCCATACATTGCAATGCTGAATGGAGAGCCGATTGGGTATGCCCAGTCGTACGTTGCTCTTGGAAGCGGGGACGGATGGTGGGAAGAAGAAACCGATCCAGGAGTACGCGGAATAGACCAGTTACTGGCGAATGCATCAGCACTGGGCAAAGGCTTGGGAACCAAGCTGGTTCGAGCTCTGGTTGAGTTGCTGTTCAATGATCCCGAGGTCACCAAGATCCAAACGGACCCGTCGCCGAGCAACTTGCGAGCGATCCGATGCTACGAGAAAGCGGGGTTTGAGAGGCAAGGTACCGTAACCACCCCAGATGGTCCAGCCGTGTACATGGTTCAAACACGCCAGGCATTCGAGCGAACACGCAGTGTTGCCTAA

>AF360392 Klebsiella pneumoniae plasmid pJHC-MW1 mutant AAC6'-IbQ174A 6'-N-acetyltransferase gene, complete cds.

GTGACCAACAGC---AACGATTCCGTCACACTGCGCCTCATGACTGAGCATGACCTTGCGATGCTCTATGAGTGGCTAAATCGATCTCATATCGTCGAGTGGTGGGGCGGAGAAGAAGCACGCCCGACACTTGCTGACGTACAGGAACAGTACTTGCCAAGCGTTTTAGCGCAAGAGTCCGTCACTCCATACATTGCAATGCTGAATGGAGAGCCGATTGGGTATGCCCAGTCGTACGTTGCTCTTGGAAGCGGGGACGGATGGTGGGAAGAAGAAACCGATCCAGGAGTACGCGGAATAGACCAGTTACTGGCGAATGCATCACAACTGGGCAAAGGCTTGGGAACCAAGCTGGTTCGAGCTCTGGTTGAGTTGCTGTTCAATGATCCCGAGGTCACCAAGATCCAAACGGACCCGTCGCCGAGCAACTTGCGAGCGATCCGATGCTACGAGAAAGCGGGGTTTGAGAGGGCAGGTACCGTAACCACCCCAGATGGTCCAGCCGTGTACATGGTTCAAACACGCCAGGCATTCGAGCGAACACGCAGTGTTGCCTAA

>AY648354 Klebsiella pneumoniae plasmid pJHCMW1 mutant aminoglycoside 6'-N-acetyltransferase-IbG127A gene, complete cds.

GTGACCAACAGC---AACGATTCCGTCACACTGCGCCTCATGACTGAGCATGACCTTGCGATGCTCTATGAGTGGCTAAATCGATCTCATATCGTCGAGTGGTGGGGCGGAGAAGAAGCACGCCCGACACTTGCTGACGTACAGGAACAGTACTTGCCAAGCGTTTTAGCGCAAGAGTCCGTCACTCCATACATTGCAATGCTGAATGGAGAGCCGATTGGGTATGCCCAGTCGTACGTTGCTCTTGGAAGCGGGGACGGATGGTGGGAAGAAGAAACCGATCCAGGAGTACGCGGAATAGACCAGTTACTGGCGAATGCATCACAACTGGCCAAAGGCTTGGGAACCAAGCTGGTTCGAGCTCTGGTTGAGTTGCTGTTCAATGATCCCGAGGTCACCAAGATCCAAACGGACCCGTCGCCGAGCAACTTGCGAGCGATCCGATGCTACGAGAAAGCGGGGTTTGAGAGGCAAGGTACCGTAACCACCCCAGATGGTCCAGCCGTGTACATGGTTCAAACACGCCAGGCATTCGAGCGAACACGCAGTGTTGCCTAA

>AY648356 Klebsiella pneumoniae plasmid pJHCMW1 mutant aminoglycoside 6'-N-acetyltransferase-IbG129A gene, complete cds.

GTGACCAACAGC---AACGATTCCGTCACACTGCGCCTCATGACTGAGCATGACCTTGCGATGCTCTATGAGTGGCTAAATCGATCTCATATCGTCGAGTGGTGGGGCGGAGAAGAAGCACGCCCGACACTTGCTGACGTACAGGAACAGTACTTGCCAAGCGTTTTAGCGCAAGAGTCCGTCACTCCATACATTGCAATGCTGAATGGAGAGCCGATTGGGTATGCCCAGTCGTACGTTGCTCTTGGAAGCGGGGACGGATGGTGGGAAGAAGAAACCGATCCAGGAGTACGCGGAATAGACCAGTTACTGGCGAATGCATCACAACTGGGCAAAGCCTTGGGAACCAAGCTGGTTCGAGCTCTGGTTGAGTTGCTGTTCAATGATCCCGAGGTCACCAAGATCCAAACGGACCCGTCGCCGAGCAACTTGCGAGCGATCCGATGCTACGAGAAAGCGGGGTTTGAGAGGCAAGGTACCGTAACCACCCCAGATGGTCCAGCCGTGTACATGGTTCAAACACGCCAGGCATTCGAGCGAACACGCAGTGTTGCCTAA

>AY648344 Klebsiella pneumoniae plasmid pJHCMW1 mutant aminoglycoside 6'-N-acetyltransferase-IbG115A gene, complete cds.

GTGACCAACAGC---AACGATTCCGTCACACTGCGCCTCATGACTGAGCATGACCTTGCGATGCTCTATGAGTGGCTAAATCGATCTCATATCGTCGAGTGGTGGGGCGGAGAAGAAGCACGCCCGACACTTGCTGACGTACAGGAACAGTACTTGCCAAGCGTTTTAGCGCAAGAGTCCGTCACTCCATACATTGCAATGCTGAATGGAGAGCCGATTGGGTATGCCCAGTCGTACGTTGCTCTTGGAAGCGGGGACGGATGGTGGGAAGAAGAAACCGATCCAGGAGTACGCGCAATAGACCAGTTACTGGCGAATGCATCACAACTGGGCAAAGGCTTGGGAACCAAGCTGGTTCGAGCTCTGGTTGAGTTGCTGTTCAATGATCCCGAGGTCACCAAGATCCAAACGGACCCGTCGCCGAGCAACTTGCGAGCGATCCGATGCTACGAGAAAGCGGGGTTTGAGAGGCAAGGTACCGTAACCACCCCAGATGGTCCAGCCGTGTACATGGTTCAAACACGCCAGGCATTCGAGCGAACACGCAGTGTTGCCTAA

>AY648341 Klebsiella pneumoniae plasmid pJHCMW1 mutant aminoglycoside 6'-N-acetyltransferase-IbG112A gene, complete cds.

GTGACCAACAGC---AACGATTCCGTCACACTGCGCCTCATGACTGAGCATGACCTTGCGATGCTCTATGAGTGGCTAAATCGATCTCATATCGTCGAGTGGTGGGGCGGAGAAGAAGCACGCCCGACACTTGCTGACGTACAGGAACAGTACTTGCCAAGCGTTTTAGCGCAAGAGTCCGTCACTCCATACATTGCAATGCTGAATGGAGAGCCGATTGGGTATGCCCAGTCGTACGTTGCTCTTGGAAGCGGGGACGGATGGTGGGAAGAAGAAACCGATCCAGCAGTACGCGGAATAGACCAGTTACTGGCGAATGCATCACAACTGGGCAAAGGCTTGGGAACCAAGCTGGTTCGAGCTCTGGTTGAGTTGCTGTTCAATGATCCCGAGGTCACCAAGATCCAAACGGACCCGTCGCCGAGCAACTTGCGAGCGATCCGATGCTACGAGAAAGCGGGGTTTGAGAGGCAAGGTACCGTAACCACCCCAGATGGTCCAGCCGTGTACATGGTTCAAACACGCCAGGCATTCGAGCGAACACGCAGTGTTGCCTAA

>AY648358 Klebsiella pneumoniae plasmid pJHCMW1 mutant aminoglycoside 6'-N-acetyltransferase-IbG131A gene, complete cds.

GTGACCAACAGC---AACGATTCCGTCACACTGCGCCTCATGACTGAGCATGACCTTGCGATGCTCTATGAGTGGCTAAATCGATCTCATATCGTCGAGTGGTGGGGCGGAGAAGAAGCACGCCCGACACTTGCTGACGTACAGGAACAGTACTTGCCAAGCGTTTTAGCGCAAGAGTCCGTCACTCCATACATTGCAATGCTGAATGGAGAGCCGATTGGGTATGCCCAGTCGTACGTTGCTCTTGGAAGCGGGGACGGATGGTGGGAAGAAGAAACCGATCCAGGAGTACGCGGAATAGACCAGTTACTGGCGAATGCATCACAACTGGGCAAAGGCTTGGCAACCAAGCTGGTTCGAGCTCTGGTTGAGTTGCTGTTCAATGATCCCGAGGTCACCAAGATCCAAACGGACCCGTCGCCGAGCAACTTGCGAGCGATCCGATGCTACGAGAAAGCGGGGTTTGAGAGGCAAGGTACCGTAACCACCCCAGATGGTCCAGCCGTGTACATGGTTCAAACACGCCAGGCATTCGAGCGAACACGCAGTGTTGCCTAA

>AF360388 Klebsiella pneumoniae plasmid pJHC-MW1 mutant AAC6'-IbG170A 6'-N-acetyltransferase gene, complete cds.

GTGACCAACAGC---AACGATTCCGTCACACTGCGCCTCATGACTGAGCATGACCTTGCGATGCTCTATGAGTGGCTAAATCGATCTCATATCGTCGAGTGGTGGGGCGGAGAAGAAGCACGCCCGACACTTGCTGACGTACAGGAACAGTACTTGCCAAGCGTTTTAGCGCAAGAGTCCGTCACTCCATACATTGCAATGCTGAATGGAGAGCCGATTGGGTATGCCCAGTCGTACGTTGCTCTTGGAAGCGGGGACGGATGGTGGGAAGAAGAAACCGATCCAGGAGTACGCGGAATAGACCAGTTACTGGCGAATGCATCACAACTGGGCAAAGGCTTGGGAACCAAGCTGGTTCGAGCTCTGGTTGAGTTGCTGTTCAATGATCCCGAGGTCACCAAGATCCAAACGGACCCGTCGCCGAGCAACTTGCGAGCGATCCGATGCTACGAGAAAGCGGCATTTGAGAGGCAAGGTACCGTAACCACCCCAGATGGTCCAGCCGTGTACATGGTTCAAACACGCCAGGCATTCGAGCGAACACGCAGTGTTGCCTAA

>AF360393 Klebsiella pneumoniae plasmid pJHC-MW1 mutant AAC6'-IbG175A 6'-N-acetyltransferase gene, complete cds.

GTGACCAACAGC---AACGATTCCGTCACACTGCGCCTCATGACTGAGCATGACCTTGCGATGCTCTATGAGTGGCTAAATCGATCTCATATCGTCGAGTGGTGGGGCGGAGAAGAAGCACGCCCGACACTTGCTGACGTACAGGAACAGTACTTGCCAAGCGTTTTAGCGCAAGAGTCCGTCACTCCATACATTGCAATGCTGAATGGAGAGCCGATTGGGTATGCCCAGTCGTACGTTGCTCTTGGAAGCGGGGACGGATGGTGGGAAGAAGAAACCGATCCAGGAGTACGCGGAATAGACCAGTTACTGGCGAATGCATCACAACTGGGCAAAGGCTTGGGAACCAAGCTGGTTCGAGCTCTGGTTGAGTTGCTGTTCAATGATCCCGAGGTCACCAAGATCCAAACGGACCCGTCGCCGAGCAACTTGCGAGCGATCCGATGCTACGAGAAAGCGGGGTTTGAGAGGCAAGCAACCGTAACCACCCCAGATGGTCCAGCCGTGTACATGGTTCAAACACGCCAGGCATTCGAGCGAACACGCAGTGTTGCCTAA

>AY648365 Klebsiella pneumoniae plasmid pJHCMW1 mutant aminoglycoside 6'-N-acetyltransferase-IbV139A gene, complete cds.

GTGACCAACAGC---AACGATTCCGTCACACTGCGCCTCATGACTGAGCATGACCTTGCGATGCTCTATGAGTGGCTAAATCGATCTCATATCGTCGAGTGGTGGGGCGGAGAAGAAGCACGCCCGACACTTGCTGACGTACAGGAACAGTACTTGCCAAGCGTTTTAGCGCAAGAGTCCGTCACTCCATACATTGCAATGCTGAATGGAGAGCCGATTGGGTATGCCCAGTCGTACGTTGCTCTTGGAAGCGGGGACGGATGGTGGGAAGAAGAAACCGATCCAGGAGTACGCGGAATAGACCAGTTACTGGCGAATGCATCACAACTGGGCAAAGGCTTGGGAACCAAGCTGGTTCGAGCTCTGGCTGAGTTGCTGTTCAATGATCCCGAGGTCACCAAGATCCAAACGGACCCGTCGCCGAGCAACTTGCGAGCGATCCGATGCTACGAGAAAGCGGGGTTTGAGAGGCAAGGTACCGTAACCACCCCAGATGGTCCAGCCGTGTACATGGTTCAAACACGCCAGGCATTCGAGCGAACACGCAGTGTTGCCTAA

>AY648342 Klebsiella pneumoniae plasmid pJHCMW1 mutant aminoglycoside 6'-N-acetyltransferase-IbV113A gene, complete cds.

GTGACCAACAGC---AACGATTCCGTCACACTGCGCCTCATGACTGAGCATGACCTTGCGATGCTCTATGAGTGGCTAAATCGATCTCATATCGTCGAGTGGTGGGGCGGAGAAGAAGCACGCCCGACACTTGCTGACGTACAGGAACAGTACTTGCCAAGCGTTTTAGCGCAAGAGTCCGTCACTCCATACATTGCAATGCTGAATGGAGAGCCGATTGGGTATGCCCAGTCGTACGTTGCTCTTGGAAGCGGGGACGGATGGTGGGAAGAAGAAACCGATCCAGGAGCACGCGGAATAGACCAGTTACTGGCGAATGCATCACAACTGGGCAAAGGCTTGGGAACCAAGCTGGTTCGAGCTCTGGTTGAGTTGCTGTTCAATGATCCCGAGGTCACCAAGATCCAAACGGACCCGTCGCCGAGCAACTTGCGAGCGATCCGATGCTACGAGAAAGCGGGGTTTGAGAGGCAAGGTACCGTAACCACCCCAGATGGTCCAGCCGTGTACATGGTTCAAACACGCCAGGCATTCGAGCGAACACGCAGTGTTGCCTAA

>AY648374 Klebsiella pneumoniae plasmid pJHCMW1 mutant aminoglycoside 6'-N-acetyltransferase-IbPV148A gene, complete cds.

GTGACCAACAGC---AACGATTCCGTCACACTGCGCCTCATGACTGAGCATGACCTTGCGATGCTCTATGAGTGGCTAAATCGATCTCATATCGTCGAGTGGTGGGGCGGAGAAGAAGCACGCCCGACACTTGCTGACGTACAGGAACAGTACTTGCCAAGCGTTTTAGCGCAAGAGTCCGTCACTCCATACATTGCAATGCTGAATGGAGAGCCGATTGGGTATGCCCAGTCGTACGTTGCTCTTGGAAGCGGGGACGGATGGTGGGAAGAAGAAACCGATCCAGGAGTACGCGGAATAGACCAGTTACTGGCGAATGCATCACAACTGGGCAAAGGCTTGGGAACCAAGCTGGTTCGAGCTCTGGTTGAGTTGCTGTTCAATGATCCCGAGGCCACCAAGATCCAAACGGACCCGTCGCCGAGCAACTTGCGAGCGATCCGATGCTACGAGAAAGCGGGGTTTGAGAGGCAAGGTACCGTAACCACCCCAGATGGTCCAGCCGTGTACATGGTTCAAACACGCCAGGCATTCGAGCGAACACGCAGTGTTGCCTAA

>AY648362 Klebsiella pneumoniae plasmid pJHCMW1 mutant aminoglycoside 6'-N-acetyltransferase-IbV135A gene, complete cds.

GTGACCAACAGC---AACGATTCCGTCACACTGCGCCTCATGACTGAGCATGACCTTGCGATGCTCTATGAGTGGCTAAATCGATCTCATATCGTCGAGTGGTGGGGCGGAGAAGAAGCACGCCCGACACTTGCTGACGTACAGGAACAGTACTTGCCAAGCGTTTTAGCGCAAGAGTCCGTCACTCCATACATTGCAATGCTGAATGGAGAGCCGATTGGGTATGCCCAGTCGTACGTTGCTCTTGGAAGCGGGGACGGATGGTGGGAAGAAGAAACCGATCCAGGAGTACGCGGAATAGACCAGTTACTGGCGAATGCATCACAACTGGGCAAAGGCTTGGGAACCAAGCTGGCTCGAGCTCTGGTTGAGTTGCTGTTCAATGATCCCGAGGTCACCAAGATCCAAACGGACCCGTCGCCGAGCAACTTGCGAGCGATCCGATGCTACGAGAAAGCGGGGTTTGAGAGGCAAGGTACCGTAACCACCCCAGATGGTCCAGCCGTGTACATGGTTCAAACACGCCAGGCATTCGAGCGAACACGCAGTGTTGCCTAA

>AY648353 Klebsiella pneumoniae plasmid pJHCMW1 mutant aminoglycoside 6'-N-acetyltransferase-IbL126A gene, complete cds.

GTGACCAACAGC---AACGATTCCGTCACACTGCGCCTCATGACTGAGCATGACCTTGCGATGCTCTATGAGTGGCTAAATCGATCTCATATCGTCGAGTGGTGGGGCGGAGAAGAAGCACGCCCGACACTTGCTGACGTACAGGAACAGTACTTGCCAAGCGTTTTAGCGCAAGAGTCCGTCACTCCATACATTGCAATGCTGAATGGAGAGCCGATTGGGTATGCCCAGTCGTACGTTGCTCTTGGAAGCGGGGACGGATGGTGGGAAGAAGAAACCGATCCAGGAGTACGCGGAATAGACCAGTTACTGGCGAATGCATCACAAGCGGGCAAAGGCTTGGGAACCAAGCTGGTTCGAGCTCTGGTTGAGTTGCTGTTCAATGATCCCGAGGTCACCAAGATCCAAACGGACCCGTCGCCGAGCAACTTGCGAGCGATCCGATGCTACGAGAAAGCGGGGTTTGAGAGGCAAGGTACCGTAACCACCCCAGATGGTCCAGCCGTGTACATGGTTCAAACACGCCAGGCATTCGAGCGAACACGCAGTGTTGCCTAA

>AY648364 Klebsiella pneumoniae plasmid pJHCMW1 mutant aminoglycoside 6'-N-acetyltransferase-IbL138A gene, complete cds.

GTGACCAACAGC---AACGATTCCGTCACACTGCGCCTCATGACTGAGCATGACCTTGCGATGCTCTATGAGTGGCTAAATCGATCTCATATCGTCGAGTGGTGGGGCGGAGAAGAAGCACGCCCGACACTTGCTGACGTACAGGAACAGTACTTGCCAAGCGTTTTAGCGCAAGAGTCCGTCACTCCATACATTGCAATGCTGAATGGAGAGCCGATTGGGTATGCCCAGTCGTACGTTGCTCTTGGAAGCGGGGACGGATGGTGGGAAGAAGAAACCGATCCAGGAGTACGCGGAATAGACCAGTTACTGGCGAATGCATCACAACTGGGCAAAGGCTTGGGAACCAAGCTGGTTCGAGCTGCGGTTGAGTTGCTGTTCAATGATCCCGAGGTCACCAAGATCCAAACGGACCCGTCGCCGAGCAACTTGCGAGCGATCCGATGCTACGAGAAAGCGGGGTTTGAGAGGCAAGGTACCGTAACCACCCCAGATGGTCCAGCCGTGTACATGGTTCAAACACGCCAGGCATTCGAGCGAACACGCAGTGTTGCCTAA

>AY648348 Klebsiella pneumoniae plasmid pJHCMW1 mutant aminoglycoside 6'-N-acetyltransferase-IbL119A gene, complete cds.

GTGACCAACAGC---AACGATTCCGTCACACTGCGCCTCATGACTGAGCATGACCTTGCGATGCTCTATGAGTGGCTAAATCGATCTCATATCGTCGAGTGGTGGGGCGGAGAAGAAGCACGCCCGACACTTGCTGACGTACAGGAACAGTACTTGCCAAGCGTTTTAGCGCAAGAGTCCGTCACTCCATACATTGCAATGCTGAATGGAGAGCCGATTGGGTATGCCCAGTCGTACGTTGCTCTTGGAAGCGGGGACGGATGGTGGGAAGAAGAAACCGATCCAGGAGTACGCGGAATAGACCAGGCACTGGCGAATGCATCACAACTGGGCAAAGGCTTGGGAACCAAGCTGGTTCGAGCTCTGGTTGAGTTGCTGTTCAATGATCCCGAGGTCACCAAGATCCAAACGGACCCGTCGCCGAGCAACTTGCGAGCGATCCGATGCTACGAGAAAGCGGGGTTTGAGAGGCAAGGTACCGTAACCACCCCAGATGGTCCAGCCGTGTACATGGTTCAAACACGCCAGGCATTCGAGCGAACACGCAGTGTTGCCTAA

>AY648357 Klebsiella pneumoniae plasmid pJHCMW1 mutant aminoglycoside 6'-N-acetyltransferase-IbL130A gene, complete cds.

GTGACCAACAGC---AACGATTCCGTCACACTGCGCCTCATGACTGAGCATGACCTTGCGATGCTCTATGAGTGGCTAAATCGATCTCATATCGTCGAGTGGTGGGGCGGAGAAGAAGCACGCCCGACACTTGCTGACGTACAGGAACAGTACTTGCCAAGCGTTTTAGCGCAAGAGTCCGTCACTCCATACATTGCAATGCTGAATGGAGAGCCGATTGGGTATGCCCAGTCGTACGTTGCTCTTGGAAGCGGGGACGGATGGTGGGAAGAAGAAACCGATCCAGGAGTACGCGGAATAGACCAGTTACTGGCGAATGCATCACAACTGGGCAAAGGCGCGGGAACCAAGCTGGTTCGAGCTCTGGTTGAGTTGCTGTTCAATGATCCCGAGGTCACCAAGATCCAAACGGACCCGTCGCCGAGCAACTTGCGAGCGATCCGATGCTACGAGAAAGCGGGGTTTGAGAGGCAAGGTACCGTAACCACCCCAGATGGTCCAGCCGTGTACATGGTTCAAACACGCCAGGCATTCGAGCGAACACGCAGTGTTGCCTAA

>AY648367 Klebsiella pneumoniae plasmid pJHCMW1 mutant aminoglycoside 6'-N-acetyltransferase-IbL141A gene, complete cds.

GTGACCAACAGC---AACGATTCCGTCACACTGCGCCTCATGACTGAGCATGACCTTGCGATGCTCTATGAGTGGCTAAATCGATCTCATATCGTCGAGTGGTGGGGCGGAGAAGAAGCACGCCCGACACTTGCTGACGTACAGGAACAGTACTTGCCAAGCGTTTTAGCGCAAGAGTCCGTCACTCCATACATTGCAATGCTGAATGGAGAGCCGATTGGGTATGCCCAGTCGTACGTTGCTCTTGGAAGCGGGGACGGATGGTGGGAAGAAGAAACCGATCCAGGAGTACGCGGAATAGACCAGTTACTGGCGAATGCATCACAACTGGGCAAAGGCTTGGGAACCAAGCTGGTTCGAGCTCTGGTTGAGGCGCTGTTCAATGATCCCGAGGTCACCAAGATCCAAACGGACCCGTCGCCGAGCAACTTGCGAGCGATCCGATGCTACGAGAAAGCGGGGTTTGAGAGGCAAGGTACCGTAACCACCCCAGATGGTCCAGCCGTGTACATGGTTCAAACACGCCAGGCATTCGAGCGAACACGCAGTGTTGCCTAA

>AY648349 Klebsiella pneumoniae plasmid pJHCMW1 mutant aminoglycoside 6'-N-acetyltransferase-IbL120A gene, complete cds.

GTGACCAACAGC---AACGATTCCGTCACACTGCGCCTCATGACTGAGCATGACCTTGCGATGCTCTATGAGTGGCTAAATCGATCTCATATCGTCGAGTGGTGGGGCGGAGAAGAAGCACGCCCGACACTTGCTGACGTACAGGAACAGTACTTGCCAAGCGTTTTAGCGCAAGAGTCCGTCACTCCATACATTGCAATGCTGAATGGAGAGCCGATTGGGTATGCCCAGTCGTACGTTGCTCTTGGAAGCGGGGACGGATGGTGGGAAGAAGAAACCGATCCAGGAGTACGCGGAATAGACCAGTTAGCGGCGAATGCATCACAACTGGGCAAAGGCTTGGGAACCAAGCTGGTTCGAGCTCTGGTTGAGTTGCTGTTCAATGATCCCGAGGTCACCAAGATCCAAACGGACCCGTCGCCGAGCAACTTGCGAGCGATCCGATGCTACGAGAAAGCGGGGTTTGAGAGGCAAGGTACCGTAACCACCCCAGATGGTCCAGCCGTGTACATGGTTCAAACACGCCAGGCATTCGAGCGAACACGCAGTGTTGCCTAA

>AY648368 Klebsiella pneumoniae plasmid pJHCMW1 mutant aminoglycoside 6'-N-acetyltransferase-IbL142A gene, complete cds.

GTGACCAACAGC---AACGATTCCGTCACACTGCGCCTCATGACTGAGCATGACCTTGCGATGCTCTATGAGTGGCTAAATCGATCTCATATCGTCGAGTGGTGGGGCGGAGAAGAAGCACGCCCGACACTTGCTGACGTACAGGAACAGTACTTGCCAAGCGTTTTAGCGCAAGAGTCCGTCACTCCATACATTGCAATGCTGAATGGAGAGCCGATTGGGTATGCCCAGTCGTACGTTGCTCTTGGAAGCGGGGACGGATGGTGGGAAGAAGAAACCGATCCAGGAGTACGCGGAATAGACCAGTTACTGGCGAATGCATCACAACTGGGCAAAGGCTTGGGAACCAAGCTGGTTCGAGCTCTGGTTGAGTTGGCGTTCAATGATCCCGAGGTCACCAAGATCCAAACGGACCCGTCGCCGAGCAACTTGCGAGCGATCCGATGCTACGAGAAAGCGGGGTTTGAGAGGCAAGGTACCGTAACCACCCCAGATGGTCCAGCCGTGTACATGGTTCAAACACGCCAGGCATTCGAGCGAACACGCAGTGTTGCCTAA

>AY648361 Klebsiella pneumoniae plasmid pJHCMW1 mutant aminoglycoside 6'-N-acetyltransferase-IbL134A gene, complete cds.

GTGACCAACAGC---AACGATTCCGTCACACTGCGCCTCATGACTGAGCATGACCTTGCGATGCTCTATGAGTGGCTAAATCGATCTCATATCGTCGAGTGGTGGGGCGGAGAAGAAGCACGCCCGACACTTGCTGACGTACAGGAACAGTACTTGCCAAGCGTTTTAGCGCAAGAGTCCGTCACTCCATACATTGCAATGCTGAATGGAGAGCCGATTGGGTATGCCCAGTCGTACGTTGCTCTTGGAAGCGGGGACGGATGGTGGGAAGAAGAAACCGATCCAGGAGTACGCGGAATAGACCAGTTACTGGCGAATGCATCACAACTGGGCAAAGGCTTGGGAACCAAGGCGGTTCGAGCTCTGGTTGAGTTGCTGTTCAATGATCCCGAGGTCACCAAGATCCAAACGGACCCGTCGCCGAGCAACTTGCGAGCGATCCGATGCTACGAGAAAGCGGGGTTTGAGAGGCAAGGTACCGTAACCACCCCAGATGGTCCAGCCGTGTACATGGTTCAAACACGCCAGGCATTCGAGCGAACACGCAGTGTTGCCTAA

>AJ223604 Pseudomonas aeruginosa integron In 101 DNA.

GTGACCAACAGC---ACCGATTCCGTCACACTGCGCCTCATGACTGAGCATGACCTTGCGATGCTCTATGAGTGGCTAAATCGATCTCATATCGTCGAGTGGTGGGGCGGAGAAGAAGCACGCCCGACACTTGCTGACGTACAGGAACAGTACTTGCCAAGCGTTTTAGCGCAAGAGTCCGTCACTCCATACATTGCAATGCTGAATGGAGAGCCGATTGGGTATGCCCAGTCGTACGTTGCTCTTGGAAGCGGGGACGGATGGTGGGAAGAAGAAACCGATCCAGGAGTACGCGGAATAGACCAGTTACTGGCGAATGCATCACAACTGGGCAAAGGCTTGGGAACCAAGCTGGTTCGAGCTCTGGTTGAGTTGCTGTTCAATGATCCCGAGGTCACCAAGATCCAAACGGACCCGTCGCCGAGCAACTTGCGAGCGATCCGATGCTACGAGAAAGCGGGGTTTGAGAGGCAAGGTACCGTAACCACCCCAGATGGTCCAGCCGTGTACATGGTTCAAACACGCCAGGCATTCGAGCGAACACGCAGTGATGCCTAA

>DQ393784 Pseudomonas aeruginosa strain N2-1 class I integron, partial sequence.

GTGACCAACAGC---ACCGATTCCGTCACACTGCGCCTCATGACTGAGCATGACCTTGCGATGCTCTATGAGTGGCTAAATCGATCTCATATCGTCGAGTGGTGGGGCGGAGAAGAAGCACGCCCGACACTTGCTGACGTACAGGAACAGTACTTGCCAAGCGTTTTAGCGCAAGAGTCCGTCACTCCATACATTGCAATGCTGAATGGAGAGCCGATTGGGTATGCCCAGTCGTACGTTGCTCTTGGAAGCGGGGACGGATGGTGGGAAGAAGAAACCGATCCAGGAGTACGCGGAATAGACCAGTTACTGGCGAATGCATCACAACTGGGCAAAGGCTTGGGAACCAAGCTGGTTCGAGCTCTGGTTGAGTTGCTGTTCAATGATCCCGAGGTCACCAAGATCCAAACGGACCCGTCGCCGAGCAACTTGCGAGCGATCCGATGCTACGAGAAAGCGGGGTTTGAGAGGCAAGGTACCGTAACCACCCCAGATGGTCCAGCCGTGTACATGGTTCAAACACGCCAGGCATTCGAGCGAACACGCAGTGATGCCTAA

>EU090799 Pseudomonas aeruginosa strain 27 class I integron metallo-beta-lactamase VIM-3 (blaVIM-3), putative protein, aminoglycoside-6'-N-acetyltransferase (aacA4), aminoglycoside 2'-O-adenylyltransferase (aadB), and aminoglycoside-6'-N-acetyltransferase (aacA4) genes, complete cds.

GTGACCAACAGC---ACCGATTCCGTCACACTGCGCCTCATGACTGAGCATGACCTTGCGATGCTCTATGAGTGGCTAAATCGATCTCATATCGTCGAGTGGTGGGGCGGAGAAGAAGCACGCCCGACACTTGCTGACGTACAGGAACAGTACTTGCCAAGCGTTTTAGCGCAAGAGTCCGTCACTCCATACATTGCAATGCTGAATGGAGAGCCGATTGGGTATGCCCAGTCGTACGTTGCTCTTGGAAGCGGGGACGGATGGTGGGAAGAAGAAACCGATCCAGGAGTACGCGGAATAGACCAGTTACTGGCGAATGCATCACAACTGGGCAAAGGCTTGGGAACCAAGCTGGTTCGAGCTCTGGTTGAGTTGCTGTTCAATGATCCCGAGGTCACCAAGATCCAAACGGACCCGTCGCCGAGCAACTTGCGAGCGATCCGATGCTACGAGAAAGCGGGGTTTGAGAGGCAAGGTACCGTAACCACCCCAGATGGTCCAGCCGTGTACATGGTTCAAACACGCCAGGCATTCGAGCGAACACGCAGTGATGCCTAA

>DQ393784 Pseudomonas aeruginosa strain N2-1 class I integron, partial sequence.

GTGACCAACAGC---ACCGATTCCGTCACACTGCGCCTCATGACTGAGCATGACCTTGCGATGCTCTATGAGTGGCTAAATCGATCTCATATCGTCGAGTGGTGGGGCGGAGAAGAAGCACGCCCGACACTTGCTGACGTACAGGAACAGTACTTGCCAAGCGTTTTAGCGCAAGAGTCCGTCACTCCATACATTGCAATGCTGAATGGAGAGCCGATTGGGTATGCCCAGTCGTACGTTGCTCTTGGAAGCGGGGACGGATGGTGGGAAGAAGAAACCGATCCAGGAGTACGCGGAATAGACCAGTTACTGGCGAATGCATCACAACTGGGCAAAGGCTTGGGAACCAAGCTGGTTCGAGCTCTGGTTGAGTTGCTGTTCAATGATCCCGAGGTCACCAAGATCCAAACGGACCCGTCGCCGAGCAACTTGCGAGCGATCCGATGCTACGAGAAAGCGGGGTTTGAGAGGCAAGGTACCGTAACCACCCCAGATGGTCCAGCCGTGTACATGGTTCAAACACGCCAGGCATTCGAGCGAACACGCAGTGATGCCTAA

>DQ393784 Pseudomonas aeruginosa strain N2-1 class I integron, partial sequence.

GTGACCAACAGC---ACCGATTCCGTCACACTGCGCCTCATGACTGAGCATGACCTTGCGATGCTCTATGAGTGGCTAAATCGATCTCATATCGTCGAGTGGTGGGGCGGAGAAGAAGCACGCCCGACACTTGCTGACGTACAGGAACAGTACTTGCCAAGCGTTTTAGCGCAAGAGTCCGTCACTCCATACATTGCAATGCTGAATGGAGAGCCGATTGGGTATGCCCAGTCGTACGTTGCTCTTGGAAGCGGGGACGGATGGTGGGAAGAAGAAACCGATCCAGGAGTACGCGGAATAGACCAGTTACTGGCGAATGCATCACAACTGGGCAAAGGCTTGGGAACCAAGCTGGTTCGAGCTCTGGTTGAGTTGCTGTTCAATGATCCCGAGGTCACCAAGATCCAAACGGACCCGTCGCCGAGCAACTTGCGAGCGATCCGATGCTACGAGAAAGCGGGGTTTGAGAGGCAAGGTACCGTAACCACCCCAGATGGTCCAGCCGTGTACATGGTTCAAACACGCCAGGCATTCGAGCGAACACGCAGTGATGCCTAA

>EU090799 Pseudomonas aeruginosa strain 27 class I integron metallo-beta-lactamase VIM-3 (blaVIM-3), putative protein, aminoglycoside-6'-N-acetyltransferase (aacA4), aminoglycoside 2'-O-adenylyltransferase (aadB), and aminoglycoside-6'-N-acetyltransferase (aacA4) genes, complete cds.

GTGACCAACAGC---ACCGATTCCGTCACACTGCGCCTCATGACTGAGCATGACCTTGCGATGCTCTATGAGTGGCTAAATCGATCTCATATCGTCGAGTGGTGGGGCGGAGAAGAAGCACGCCCGACACTTGCTGACGTACAGGAACAGTACTTGCCAAGCGTTTTAGCGCAAGAGTCCGTCACTCCATACATTGCAATGCTGAATGGAGAGCCGATTGGGTATGCCCAGTCGTACGTTGCTCTTGGAAGCGGGGACGGATGGTGGGAAGAAGAAACCGATCCAGGAGTACGCGGAATAGACCAGTTACTGGCGAATGCATCACAACTGGGCAAAGGCTTGGGAACCAAGCTGGTTCGAGCTCTGGTTGAGTTGCTGTTCAATGATCCCGAGGTCACCAAGATCCAAACGGACCCGTCGCCGAGCAACTTGCGAGCGATCCGATGCTACGAGAAAGCGGGGTTTGAGAGGCAAGGTACCGTAACCACCCCAGATGGTCCAGCCGTGTACATGGTTCAAACACGCCAGGCATTCGAGCGAACACGCAGTGATGCCTAA

>AF360380 Klebsiella pneumoniae plasmid pJHC-MW1 mutant AAC6'-IbL160A 6'-N-acetyltransferase gene, complete cds.

GTGACCAACAGC---AACGATTCCGTCACACTGCGCCTCATGACTGAGCATGACCTTGCGATGCTCTATGAGTGGCTAAATCGATCTCATATCGTCGAGTGGTGGGGCGGAGAAGAAGCACGCCCGACACTTGCTGACGTACAGGAACAGTACTTGCCAAGCGTTTTAGCGCAAGAGTCCGTCACTCCATACATTGCAATGCTGAATGGAGAGCCGATTGGGTATGCCCAGTCGTACGTTGCTCTTGGAAGCGGGGACGGATGGTGGGAAGAAGAAACCGATCCAGGAGTACGCGGAATAGACCAGTTACTGGCGAATGCATCACAACTGGGCAAAGGCTTGGGAACCAAGCTGGTTCGAGCTCTGGTTGAGTTGCTGTTCAATGATCCCGAGGTCACCAAGATCCAAACGGACCCGTCGCCGAGCAACGCACGAGCGATCCGATGCTACGAGAAAGCGGGGTTTGAGAGGCAAGGTACCGTAACCACCCCAGATGGTCCAGCCGTGTACATGGTTCAAACACGCCAGGCATTCGAGCGAACACGCAGTGTTGCCTAA

>AF012280 Plasmid pJHC-MW1 transposon Tn1331 mutant aminoglycoside 6'-N-acetyltransferase gene, complete cds.

GTGACCAACAGC---AACGATTCCGTCACACTGCGCCTCATGACTGAGCATGACCTTGCGATGCTCTATGAGTGGCTAAATCGATCTCATATCGTCGAGTGGTGGGGCGGAGAAGAAGCACGCCCGACACTTGCTGACGTACAGGAACAGTACTTGCCAAGCGTTTTAGCGCAAGAGTCCGTCACTCCATGCATTGCAATGCTGAATGGAGAGCCGATTGGGTATGCCCAGTCGTACGTTGCTCTTGGAAGCGGGGACGGATGGTGGGAAGAAGAAACCGATCCAGGAGTACGCGGAATAGACCAGTTACTGGCGAATGCATCACAACTGGGCAAAGGCTTGGGAACCAAGCTGGTTCGAGCTCTGGTTGAGTTGCTGTTCAATGATCCCGAGGTCACCAAGATCCAAACGGACCCGTCGCCGAGCAACTTGCGAGCGATCCGATGCTACGAGAAAGCGGGGTTTGAGAGGCAAGGTACCGTAACCACCCCAGATGGTCCAGCCGTGTACATGGTTCAAACACGCCAGGCATTCGAGCGAACACGCAGTGTTGCCTAA

>DQ836057 Escherichia coli class I integron aminoglycoside 6'-N-acetyltransferase (aacA4), chloramphenicol acetyltransferase (catB3), and dihydrofolate reductase (dfrA1) genes, complete cds.

GTGACCAACAGC---AACGATTCCGTCACACTGCGCCTCATGACTGAGCATGACCTTGCGATGCTCTATGAGTGGCTAAATCGATCTCATATCGTCGAGTGGTGGGGCGGAGAAGAAGCACGCCCGACACTTGCTGACGTACAGGAACAGTACTTGCCAAGCGTTTTAGCGCAAGAGTCCGTCACTCCATACATTGCAATGCTGAATGGAGAGCCGATTGGGTATGCCCAGTCGTACGTTGCTCTTGGAAGCGGGGACGGATGGTGGGAAGAAGAAACCGATCCAGGAGTACGCGGAATAGACCAGTCACTGGCGAATGCATCACAACTGGGCAAAGGCTTGGGAACCAAGCTGGTTCGAGCACTGGTTGAGTTGCTGTTCAATGATCCCGAGGTCACCAAGATCCAAACGGACCCGTCGCCGAGCAACTTGCGAGCGATCCGATGCTACGAGAAAGCGGGGTTTGAGAGGCAAGGTACCGTAACCACCCCAGATGGTCCAGCCGTGTACATGGTTCAAACACGCCAGGCATTCGAGCGAACACGCAGTGATGCCTAA

>EF488369 Escherichia coli clone 1008 class I integron, complete sequence.

GTGACCAACAGC---AACGATTCCGTCACACTGCGCCTCATGACTGAGCATGACCTTGCGATGCTCTATGAGTGGCTAAATCGATCTCATATCGTCGAGTGGTGGGGCGGAGAAGAAGCACGCCCGACACTTGCTGACGTACAGGAACAGTACTTGCCAAGCGTTTTAGCGCAAGAGTCCGTCACTCCATACATTGCAATGCTGAATGGAGAGCCGATTGGGTATGCCCAGTCGTACGTTGCTCTTGGAAGCGGGGACGGATGGTGGGAAGAAGAAACCGATCCAGGAGTACGCGGAATAGACCAGTCACTGGCGAATGCATCACAACTGGGCAAAGGCTTGGGAACCAAGCTGGTTCGAGCACTGGTTGAGTTGCTGTTCAATGATCCCGAGGTCACCAAGATCCAAACGGACCCGTCGCCGAGCAACTTGCGAGCGATCCGATGCTACGAGAAAGCGGGGTTTGAGAGGCAAGGTACCGTAACCACCCCAGATGGTCCAGCCGTGTACATGGTTCAAACACGCCAGGCATTCGAGCGAACACGCAGTGATGCCTAA

>EF516991 Escherichia coli strain 347 class I integron aminoglycoside 6'-N-acetyltransferase (aacA4), chloramphenicol acetyltransferase (catB3), and dihydrofolate reductase (dfrA1) genes, complete cds.

GTGACCAACAGC---AACGATTCCGTCACACTGCGCCTCATGACTGAGCATGACCTTGCGATGCTCTATGAGTGGCTAAATCGATCTCATATCGTCGAGTGGTGGGGCGGAGAAGAAGCACGCCCGACACTTGCTGACGTACAGGAACAGTACTTGCCAAGCGTTTTAGCGCAAGAGTCCGTCACTCCATACATTGCAATGCTGAATGGAGAGCCGATTGGGTATGCCCAGTCGTACGTTGCTCTTGGAAGCGGGGACGGATGGTGGGAAGAAGAAACCGATCCAGGAGTACGCGGAATAGACCAGTCACTGGCGAATGCATCACAACTGGGCAAAGGCTTGGGAACCAAGCTGGTTCGAGCACTGGTTGAGTTGCTGTTCAATGATCCCGAGGTCACCAAGATCCAAACGGACCCGTCGCCGAGCAACTTGCGAGCGATCCGATGCTACGAGAAAGCGGGGTTTGAGAGGCAAGGTACCGTAACCACCCCAGATGGTCCAGCCGTGTACATGGTTCAAACACGCCAGGCATTCGAGCGAACACGCAGTGATGCCTAA

>AF315351 Pseudomonas aeruginosa integron integrase (int1) gene, partial cds; and beta-lactamase OXA-32 (bla oxa-32) and acetyltransferase (aacA4) genes, complete cds.

GTGACCAACAGC---AACGATTCCGTCACACTGCGCCTCATGACTGAGCATGACCTTGCGATGCTCTATGAGTGGCTAAATCGATCTCATATCGTCGAGTGGTGGGGCGGAGAAGAAGCACGCCCGACACTTGCTGACGTACAGGAACAGTACTTGCCAAGCGTTTTAGCGCAAGAGTCCGTCACTCCATACATTGCAATGCTGAATGGAGAGCCGATTGGGTATGCCCAGTCGTACGTTGCTCTTGGAAGCGGGGACGGATGGTGGGAAGAAGAAACCGATCCAGGAGTACGCGGAATAGACCAGTCACTGGCGAATGCATCACAACTGGGCAAAGGCTTGGGAACCAAGCTGGTTCGAGCACTGGTTGAGTTGCTGTTCAATGATCCCGAGGTCACCAAGATCCAAACGGACCCGTCGCCGAGCAACTTGCGAGCGATCCGATGCTACGAGAAAGCGGGGTTTGAGAGGCAAGGTACCGTAACCACCCCAGATGGTCCAGCCGTGTACATGGTTCAAACACGCCAGGCATTCGAGCGAACACGCAGTGATGCCTAA

>AJ243491 Acinetobacter baumannii partial intI1 gene and blaIMP-2, aacA4 and aadA1 genes.

GTGACCAACAGC---AACGATTCCGTCACACTGCGCCTCATGACTGAGCATGACCTTGCGATGCTCTATGAGTGGCTAAATCGATCTCATATCGTCGAGTGGTGGGGCGGAGAAGAAGCACGCCCGACACTTGCTGACGTACAGGAACAGTACTTGCCAAGCGTTTTAGCGCAAGAGTCCGTCACTCCATACATTGCAATGCTGAATGGAGAGCCGATTGGGTATGCCCAGTCGTACGTTGCTCTTGGAAGCGGGGACGGATGGTGGGAAGAAGAAACCGATCCAGGAGTACGCGGAATAGACCAGTCACTGGCGAATGCATCACAACTGGGCAAAGGCTTGGGAACCAAGCTGGTTCGAGCACTGGTTGAGTTGCTGTTCAATGATCCCGAGGTCACCAAGATCCAAACGGACCCGTCGCCGAGCAACTTGCGAGCGATCCGATGCTACGAGAAAGCGGGGTTTGAGAGGCAAGGTACCGTAACCACCCCAGATGGTCCAGCCGTGTACATGGTTCAAACACGCCAGGCATTCGAGCGAACACGCAGTGATGCCTAA

>AF317511 Pseudomonas aeruginosa integron In80 class 1 DNA integrase (intI1) gene, partial cds; and metallo-beta-lactamase (blaVIM-1), aminoglycoside 6'-N-acetyltransferase type II enzyme (aac6'-Ib'), aminoglycoside 6'-N-acetyltransferase type II enzyme (aac6'-Ib'), and OXA-like protein (blaOXA) genes, complete cds.

GTGACCAACAGC---AACGATTCCGTCACACTGCGCCTCATGACTGAGCATGACCTTGCGATGCTCTATGAGTGGCTAAATCGATCTCATATCGTCGAGTGGTGGGGCGGAGAAGAAGCACGCCCGACACTTGCTGACGTACAGGAACAGTACTTGCCAAGCGTTTTAGCGCAAGAGTCCGTCACTCCATACATTGCAATGCTGAATGGAGAGCCGATTGGGTATGCCCAGTCGTACGTTGCTCTTGGAAGCGGGGACGGATGGTGGGAAGAAGAAACCGATCCAGGAGTACGCGGAATAGACCAGTCACTGGCGAATGCATCACAACTGGGCAAAGGCTTGGGAACCAAGCTGGTTCGAGCACTGGTTGAGTTGCTGTTCAATGATCCCGAGGTCACCAAGATCCAAACGGACCCGTCGCCGAGCAACTTGCGAGCGATCCGATGCTACGAGAAAGCGGGGTTTGAGAGGCAAGGTACCGTAACCACCCCAGATGGTCCAGCCGTGTACATGGTTCAAACACGCCAGGCATTCGAGCGAACACGCAGTGATGCCTAA

>AF317511 Pseudomonas aeruginosa integron In80 class 1 DNA integrase (intI1) gene, partial cds; and metallo-beta-lactamase (blaVIM-1), aminoglycoside 6'-N-acetyltransferase type II enzyme (aac6'-Ib'), aminoglycoside 6'-N-acetyltransferase type II enzyme (aac6'-Ib'), and OXA-like protein (blaOXA) genes, complete cds.

GTGACCAACAGC---AACGATTCCGTCACACTGCGCCTCATGACTGAGCATGACCTTGCGATGCTCTATGAGTGGCTAAATCGATCTCATATCGTCGAGTGGTGGGGCGGAGAAGAAGCACGCCCGACACTTGCTGACGTACAGGAACAGTACTTGCCAAGCGTTTTAGCGCAAGAGTCCGTCACTCCATACATTGCAATGCTGAATGGAGAGCCGATTGGGTATGCCCAGTCGTACGTTGCTCTTGGAAGCGGGGACGGATGGTGGGAAGAAGAAACCGATCCAGGAGTACGCGGAATAGACCAGTCACTGGCGAATGCATCACAACTGGGCAAAGGCTTGGGAACCAAGCTGGTTCGAGCACTGGTTGAGTTGCTGTTCAATGATCCCGAGGTCACCAAGATCCAAACGGACCCGTCGCCGAGCAACTTGCGAGCGATCCGATGCTACGAGAAAGCGGGGTTTGAGAGGCAAGGTACCGTAACCACCCCAGATGGTCCAGCCGTGTACATGGTTCAAACACGCCAGGCATTCGAGCGAACACGCAGTGATGCCTAA

>AJ784804 Pseudomonas aeruginosa partial integron, isolate 85-2394.

GTGACCAACAGC---AACGATTCCGTCACACTGCGCCTCATGACTGAGCATGACCTTGCGATGCTCTATGAGTGGCTAAATCGATCTCATATCGTCGAGTGGTGGGGCGGAGAAGAAGCACGCCCGACACTTGCTGACGTACAGGAACAGTACTTGCCAAGCGTTTTAGCGCAAGAGTCCGTCACTCCATACATTGCAATGCTGAATGGAGAGCCGATTGGGTATGCCCAGTCGTACGTTGCTCTTGGAAGCGGGGACGGATGGTGGGAAGAAGAAACCGATCCAGGAGTACGCGGAATAGACCAGTCACTGGCGAATGCATCACAACTGGGCAAAGGCTTGGGAACCAAGCTGGTTCGAGCACTGGTTGAGTTGCTGTTCAATGATCCCGAGGTCACCAAGATCCAAACGGACCCGTCGCCGAGCAACTTGCGAGCGATCCGATGCTACGAGAAAGCGGGGTTTGAGAGGCAAGGTACCGTAACCACCCCAGATGGTCCAGCCGTGTACATGGTTCAAACACGCCAGGCATTCGAGCGAACACGCAGTGATGCCTAA

>AJ784805 Pseudomonas aeruginosa partial integron, isolate 86-10088.

GTGACCAACAGC---AACGATTCCGTCACACTGCGCCTCATGACTGAGCATGACCTTGCGATGCTCTATGAGTGGCTAAATCGATCTCATATCGTCGAGTGGTGGGGCGGAGAAGAAGCACGCCCGACACTTGCTGACGTACAGGAACAGTACTTGCCAAGCGTTTTAGCGCAAGAGTCCGTCACTCCATACATTGCAATGCTGAATGGAGAGCCGATTGGGTATGCCCAGTCGTACGTTGCTCTTGGAAGCGGGGACGGATGGTGGGAAGAAGAAACCGATCCAGGAGTACGCGGAATAGACCAGTCACTGGCGAATGCATCACAACTGGGCAAAGGCTTGGGAACCAAGCTGGTTCGAGCACTGGTTGAGTTGCTGTTCAATGATCCCGAGGTCACCAAGATCCAAACGGACCCGTCGCCGAGCAACTTGCGAGCGATCCGATGCTACGAGAAAGCGGGGTTTGAGAGGCAAGGTACCGTAACCACCCCAGATGGTCCAGCCGTGTACATGGTTCAAACACGCCAGGCATTCGAGCGAACACGCAGTGATGCCTAA

>AJ969233 Pseudomonas aeruginosa Tn5051-like transposon and class 1 integron In70, isolate 66MG.

GTGACCAACAGC---AACGATTCCGTCACACTGCGCCTCATGACTGAGCATGACCTTGCGATGCTCTATGAGTGGCTAAATCGATCTCATATCGTCGAGTGGTGGGGCGGAGAAGAAGCACGCCCGACACTTGCTGACGTACAGGAACAGTACTTGCCAAGCGTTTTAGCGCAAGAGTCCGTCACTCCATACATTGCAATGCTGAATGGAGAGCCGATTGGGTATGCCCAGTCGTACGTTGCTCTTGGAAGCGGGGACGGATGGTGGGAAGAAGAAACCGATCCAGGAGTACGCGGAATAGACCAGTCACTGGCGAATGCATCACAACTGGGCAAAGGCTTGGGAACCAAGCTGGTTCGAGCACTGGTTGAGTTGCTGTTCAATGATCCCGAGGTCACCAAGATCCAAACGGACCCGTCGCCGAGCAACTTGCGAGCGATCCGATGCTACGAGAAAGCGGGGTTTGAGAGGCAAGGTACCGTAACCACCCCAGATGGTCCAGCCGTGTACATGGTTCAAACACGCCAGGCATTCGAGCGAACACGCAGTGATGCCTAA

>AJ969234 Pseudomonas aeruginosa class 1 integron In110, isolate 67MG.

GTGACCAACAGC---AACGATTCCGTCACACTGCGCCTCATGACTGAGCATGACCTTGCGATGCTCTATGAGTGGCTAAATCGATCTCATATCGTCGAGTGGTGGGGCGGAGAAGAAGCACGCCCGACACTTGCTGACGTACAGGAACAGTACTTGCCAAGCGTTTTAGCGCAAGAGTCCGTCACTCCATACATTGCAATGCTGAATGGAGAGCCGATTGGGTATGCCCAGTCGTACGTTGCTCTTGGAAGCGGGGACGGATGGTGGGAAGAAGAAACCGATCCAGGAGTACGCGGAATAGACCAGTCACTGGCGAATGCATCACAACTGGGCAAAGGCTTGGGAACCAAGCTGGTTCGAGCACTGGTTGAGTTGCTGTTCAATGATCCCGAGGTCACCAAGATCCAAACGGACCCGTCGCCGAGCAACTTGCGAGCGATCCGATGCTACGAGAAAGCGGGGTTTGAGAGGCAAGGTACCGTAACCACCCCAGATGGTCCAGCCGTGTACATGGTTCAAACACGCCAGGCATTCGAGCGAACACGCAGTGATGCCTAA

>AJ969235 Pseudomonas aeruginosa class 1 integron In70, isolate 35MG.

GTGACCAACAGC---AACGATTCCGTCACACTGCGCCTCATGACTGAGCATGACCTTGCGATGCTCTATGAGTGGCTAAATCGATCTCATATCGTCGAGTGGTGGGGCGGAGAAGAAGCACGCCCGACACTTGCTGACGTACAGGAACAGTACTTGCCAAGCGTTTTAGCGCAAGAGTCCGTCACTCCATACATTGCAATGCTGAATGGAGAGCCGATTGGGTATGCCCAGTCGTACGTTGCTCTTGGAAGCGGGGACGGATGGTGGGAAGAAGAAACCGATCCAGGAGTACGCGGAATAGACCAGTCACTGGCGAATGCATCACAACTGGGCAAAGGCTTGGGAACCAAGCTGGTTCGAGCACTGGTTGAGTTGCTGTTCAATGATCCCGAGGTCACCAAGATCCAAACGGACCCGTCGCCGAGCAACTTGCGAGCGATCCGATGCTACGAGAAAGCGGGGTTTGAGAGGCAAGGTACCGTAACCACCCCAGATGGTCCAGCCGTGTACATGGTTCAAACACGCCAGGCATTCGAGCGAACACGCAGTGATGCCTAA

>AJ969236 Pseudomonas aeruginosa class 1 integron In70, isolate 17MG.

GTGACCAACAGC---AACGATTCCGTCACACTGCGCCTCATGACTGAGCATGACCTTGCGATGCTCTATGAGTGGCTAAATCGATCTCATATCGTCGAGTGGTGGGGCGGAGAAGAAGCACGCCCGACACTTGCTGACGTACAGGAACAGTACTTGCCAAGCGTTTTAGCGCAAGAGTCCGTCACTCCATACATTGCAATGCTGAATGGAGAGCCGATTGGGTATGCCCAGTCGTACGTTGCTCTTGGAAGCGGGGACGGATGGTGGGAAGAAGAAACCGATCCAGGAGTACGCGGAATAGACCAGTCACTGGCGAATGCATCACAACTGGGCAAAGGCTTGGGAACCAAGCTGGTTCGAGCACTGGTTGAGTTGCTGTTCAATGATCCCGAGGTCACCAAGATCCAAACGGACCCGTCGCCGAGCAACTTGCGAGCGATCCGATGCTACGAGAAAGCGGGGTTTGAGAGGCAAGGTACCGTAACCACCCCAGATGGTCCAGCCGTGTACATGGTTCAAACACGCCAGGCATTCGAGCGAACACGCAGTGATGCCTAA

>AJ969237 Pseudomonas aeruginosa class 1 integron In80, isolate 54MG.

GTGACCAACAGC---AACGATTCCGTCACACTGCGCCTCATGACTGAGCATGACCTTGCGATGCTCTATGAGTGGCTAAATCGATCTCATATCGTCGAGTGGTGGGGCGGAGAAGAAGCACGCCCGACACTTGCTGACGTACAGGAACAGTACTTGCCAAGCGTTTTAGCGCAAGAGTCCGTCACTCCATACATTGCAATGCTGAATGGAGAGCCGATTGGGTATGCCCAGTCGTACGTTGCTCTTGGAAGCGGGGACGGATGGTGGGAAGAAGAAACCGATCCAGGAGTACGCGGAATAGACCAGTCACTGGCGAATGCATCACAACTGGGCAAAGGCTTGGGAACCAAGCTGGTTCGAGCACTGGTTGAGTTGCTGTTCAATGATCCCGAGGTCACCAAGATCCAAACGGACCCGTCGCCGAGCAACTTGCGAGCGATCCGATGCTACGAGAAAGCGGGGTTTGAGAGGCAAGGTACCGTAACCACCCCAGATGGTCCAGCCGTGTACATGGTTCAAACACGCCAGGCATTCGAGCGAACACGCAGTGATGCCTAA

>AJ969237 Pseudomonas aeruginosa class 1 integron In80, isolate 54MG.

GTGACCAACAGC---AACGATTCCGTCACACTGCGCCTCATGACTGAGCATGACCTTGCGATGCTCTATGAGTGGCTAAATCGATCTCATATCGTCGAGTGGTGGGGCGGAGAAGAAGCACGCCCGACACTTGCTGACGTACAGGAACAGTACTTGCCAAGCGTTTTAGCGCAAGAGTCCGTCACTCCATACATTGCAATGCTGAATGGAGAGCCGATTGGGTATGCCCAGTCGTACGTTGCTCTTGGAAGCGGGGACGGATGGTGGGAAGAAGAAACCGATCCAGGAGTACGCGGAATAGACCAGTCACTGGCGAATGCATCACAACTGGGCAAAGGCTTGGGAACCAAGCTGGTTCGAGCACTGGTTGAGTTGCTGTTCAATGATCCCGAGGTCACCAAGATCCAAACGGACCCGTCGCCGAGCAACTTGCGAGCGATCCGATGCTACGAGAAAGCGGGGTTTGAGAGGCAAGGTACCGTAACCACCCCAGATGGTCCAGCCGTGTACATGGTTCAAACACGCCAGGCATTCGAGCGAACACGCAGTGATGCCTAA

>AJ867812 Pseudomonas aeruginosa Tn5051-like transposon and class I integron In110, strain 134MG.

GTGACCAACAGC---AACGATTCCGTCACACTGCGCCTCATGACTGAGCATGACCTTGCGATGCTCTATGAGTGGCTAAATCGATCTCATATCGTCGAGTGGTGGGGCGGAGAAGAAGCACGCCCGACACTTGCTGACGTACAGGAACAGTACTTGCCAAGCGTTTTAGCGCAAGAGTCCGTCACTCCATACATTGCAATGCTGAATGGAGAGCCGATTGGGTATGCCCAGTCGTACGTTGCTCTTGGAAGCGGGGACGGATGGTGGGAAGAAGAAACCGATCCAGGAGTACGCGGAATAGACCAGTCACTGGCGAATGCATCACAACTGGGCAAAGGCTTGGGAACCAAGCTGGTTCGAGCACTGGTTGAGTTGCTGTTCAATGATCCCGAGGTCACCAAGATCCAAACGGACCCGTCGCCGAGCAACTTGCGAGCGATCCGATGCTACGAGAAAGCGGGGTTTGAGAGGCAAGGTACCGTAACCACCCCAGATGGTCCAGCCGTGTACATGGTTCAAACACGCCAGGCATTCGAGCGAACACGCAGTGATGCCTAA

>EF577408 Pseudomonas aeruginosa strain PA-SD2 class I integron IntI1 (intI1), class B carbapenemase VIM-1 (blaVIM-1), AacA4 (aacA4), and AadA1 (aadA1) genes, complete cds.

GTGACCAACAGC---AACGATTCCGTCACACTGCGCCTCATGACTGAGCATGACCTTGCGATGCTCTATGAGTGGCTAAATCGATCTCATATCGTCGAGTGGTGGGGCGGAGAAGAAGCACGCCCGACACTTGCTGACGTACAGGAACAGTACTTGCCAAGCGTTTTAGCGCAAGAGTCCGTCACTCCATACATTGCAATGCTGAATGGAGAGCCGATTGGGTATGCCCAGTCGTACGTTGCTCTTGGAAGCGGGGACGGATGGTGGGAAGAAGAAACCGATCCAGGAGTACGCGGAATAGACCAGTCACTGGCGAATGCATCACAACTGGGCAAAGGCTTGGGAACCAAGCTGGTTCGAGCACTGGTTGAGTTGCTGTTCAATGATCCCGAGGTCACCAAGATCCAAACGGACCCGTCGCCGAGCAACTTGCGAGCGATCCGATGCTACGAGAAAGCGGGGTTTGAGAGGCAAGGTACCGTAACCACCCCAGATGGTCCAGCCGTGTACATGGTTCAAACACGCCAGGCATTCGAGCGAACACGCAGTGATGCCTAA

>AF024602 Pseudomonas aeruginosa integrase (tnpI) gene, partial cds; AacA4 protein (aacA4), beta-lactamase (oxa-20), genes, complete cds; resolvase (tnpR) pseudogene, partial sequence; streptomycin resistance protein A (strA), and streptomycin resistance protein B (strB) genes, complete cds.

GTGACCAACAGC---AACGATTCCGTCACACTGCGCCTCATGACTGAGCATGACCTTGCGATGCTCTATGAGTGGCTAAATCGATCTCATATCGTCGAGTGGTGGGGCGGAGAAGAAGCACGCCCGACACTTGCTGACGTACAGGAACAGTACTTGCCAAGCGTTTTAGCGCAAGAGTCCGTCACTCCATACATTGCAATGCTGAATGGAGAGCCGATTGGGTATGCCCAGTCGTACGTTGCTCTTGGAAGCGGGGACGGATGGTGGGAAGAAGAAACCGATCCAGGAGTACGCGGAATAGACCAGTCACTGGCGAATGCATCACAACTGGGCAAAGGCTTGGGAACCAAGCTGGTTCGAGCACTGGTTGAGTTGCTGTTCAATGATCCCGAGGTCACCAAGATCCAAACGGACCCGTCGCCGAGCAACTTGCGAGCGATCCGATGCTACGAGAAAGCGGGGTTTGAGAGGCAAGGTACCGTAACCACCCCAGATGGTCCAGCCGTGTACATGGTTCAAACACGCCAGGCATTCGAGCGAACACGCAGTGATGCCTAA

>AJ291609 Pseudomonas aeruginosa intI1 gene (partial), blaVIM-1 gene, aacA4 gene, aph gene and aadA1 gene.

GTGACCAACAGC---AACGATTCCGTCACACTGCGCCTCATGACTGAGCATGACCTTGCGATGCTCTATGAGTGGCTAAATCGATCTCATATCGTCGAGTGGTGGGGCGGAGAAGAAGCACGCCCGACACTTGCTGACGTACAGGAACAGTACTTGCCAAGCGTTTTAGCGCAAGAGTCCGTCACTCCATACATTGCAATGCTGAATGGAGAGCCGATTGGGTATGCCCAGTCGTACGTTGCTCTTGGAAGCGGGGACGGATGGTGGGAAGAAGAAACCGATCCAGGAGTACGCGGAATAGACCAGTCACTGGCGAATGCATCACAACTGGGCAAAGGCTTGGGAACCAAGCTGGTTCGAGCACTGGTTGAGTTGCTGTTCAATGATCCCGAGGTCACCAAGATCCAAACGGACCCGTCGCCGAGCAACTTGCGAGCGATCCGATGCTACGAGAAAGCGGGGTTTGAGAGGCAAGGTACCGTAACCACCCCAGATGGTCCAGCCGTGTACATGGTTCAAACACGCCAGGCATTCGAGCGAACACGCAGTGATGCCTAA

>Y18050 Pseudomonas aeruginosa intI1, blaVIM, aacA4, aphA15, aadA1 and qacEdelta1 genes.

GTGACCAACAGC---AACGATTCCGTCACACTGCGCCTCATGACTGAGCATGACCTTGCGATGCTCTATGAGTGGCTAAATCGATCTCATATCGTCGAGTGGTGGGGCGGAGAAGAAGCACGCCCGACACTTGCTGACGTACAGGAACAGTACTTGCCAAGCGTTTTAGCGCAAGAGTCCGTCACTCCATACATTGCAATGCTGAATGGAGAGCCGATTGGGTATGCCCAGTCGTACGTTGCTCTTGGAAGCGGGGACGGATGGTGGGAAGAAGAAACCGATCCAGGAGTACGCGGAATAGACCAGTCACTGGCGAATGCATCACAACTGGGCAAAGGCTTGGGAACCAAGCTGGTTCGAGCACTGGTTGAGTTGCTGTTCAATGATCCCGAGGTCACCAAGATCCAAACGGACCCGTCGCCGAGCAACTTGCGAGCGATCCGATGCTACGAGAAAGCGGGGTTTGAGAGGCAAGGTACCGTAACCACCCCAGATGGTCCAGCCGTGTACATGGTTCAAACACGCCAGGCATTCGAGCGAACACGCAGTGATGCCTAA

>AJ581664 Pseudomonas aeruginosa insertion sequence ISPa7 transposon tnpA gene, intI1 gene, blaVIM gene, aacA4 gene, aphA15 gene, aadA1 gene and qacEdelta1 gene (partial), isolate TS-832035.

GTGACCAACAGC---AACGATTCCGTCACACTGCGCCTCATGACTGAGCATGACCTTGCGATGCTCTATGAGTGGCTAAATCGATCTCATATCGTCGAGTGGTGGGGCGGAGAAGAAGCACGCCCGACACTTGCTGACGTACAGGAACAGTACTTGCCAAGCGTTTTAGCGCAAGAGTCCGTCACTCCATACATTGCAATGCTGAATGGAGAGCCGATTGGGTATGCCCAGTCGTACGTTGCTCTTGGAAGCGGGGACGGATGGTGGGAAGAAGAAACCGATCCAGGAGTACGCGGAATAGACCAGTCACTGGCGAATGCATCACAACTGGGCAAAGGCTTGGGAACCAAGCTGGTTCGAGCACTGGTTGAGTTGCTGTTCAATGATCCCGAGGTCACCAAGATCCAAACGGACCCGTCGCCGAGCAACTTGCGAGCGATCCGATGCTACGAGAAAGCGGGGTTTGAGAGGCAAGGTACCGTAACCACCCCAGATGGTCCAGCCGTGTACATGGTTCAAACACGCCAGGCATTCGAGCGAACACGCAGTGATGCCTAA

>AJ581665 Pseudomonas aeruginosa insertion sequence ISPa7 transposon tnpA gene, intI1 gene, blaVIM gene, aacA4 gene, aphA15 gene, aadA1 gene and qacEdelta1 gene (partial), isolate PPV-108.

GTGACCAACAGC---AACGATTCCGTCACACTGCGCCTCATGACTGAGCATGACCTTGCGATGCTCTATGAGTGGCTAAATCGATCTCATATCGTCGAGTGGTGGGGCGGAGAAGAAGCACGCCCGACACTTGCTGACGTACAGGAACAGTACTTGCCAAGCGTTTTAGCGCAAGAGTCCGTCACTCCATACATTGCAATGCTGAATGGAGAGCCGATTGGGTATGCCCAGTCGTACGTTGCTCTTGGAAGCGGGGACGGATGGTGGGAAGAAGAAACCGATCCAGGAGTACGCGGAATAGACCAGTCACTGGCGAATGCATCACAACTGGGCAAAGGCTTGGGAACCAAGCTGGTTCGAGCACTGGTTGAGTTGCTGTTCAATGATCCCGAGGTCACCAAGATCCAAACGGACCCGTCGCCGAGCAACTTGCGAGCGATCCGATGCTACGAGAAAGCGGGGTTTGAGAGGCAAGGTACCGTAACCACCCCAGATGGTCCAGCCGTGTACATGGTTCAAACACGCCAGGCATTCGAGCGAACACGCAGTGATGCCTAA

>AM183120 Enterobacter cloacae partial integron In71.

GTGACCAACAGC---AACGATTCCGTCACACTGCGCCTCATGACTGAGCATGACCTTGCGATGCTCTATGAGTGGCTAAATCGATCTCATATCGTCGAGTGGTGGGGCGGAGAAGAAGCACGCCCGACACTTGCTGACGTACAGGAACAGTACTTGCCAAGCGTTTTAGCGCAAGAGTCCGTCACTCCATACATTGCAATGCTGAATGGAGAGCCGATTGGGTATGCCCAGTCGTACGTTGCTCTTGGAAGCGGGGACGGATGGTGGGAAGAAGAAACCGATCCAGGAGTACGCGGAATAGACCAGTCACTGGCGAATGCATCACAACTGGGCAAAGGCTTGGGAACCAAGCTGGTTCGAGCACTGGTTGAGTTGCTGTTCAATGATCCCGAGGTCACCAAGATCCAAACGGACCCGTCGCCGAGCAACTTGCGAGCGATCCGATGCTACGAGAAAGCGGGGTTTGAGAGGCAAGGTACCGTAACCACCCCAGATGGTCCAGCCGTGTACATGGTTCAAACACGCCAGGCATTCGAGCGAACACGCAGTGATGCCTAA

>AY648360 Klebsiella pneumoniae plasmid pJHCMW1 mutant aminoglycoside 6'-N-acetyltransferase-IbK133A gene, complete cds.

GTGACCAACAGC---AACGATTCCGTCACACTGCGCCTCATGACTGAGCATGACCTTGCGATGCTCTATGAGTGGCTAAATCGATCTCATATCGTCGAGTGGTGGGGCGGAGAAGAAGCACGCCCGACACTTGCTGACGTACAGGAACAGTACTTGCCAAGCGTTTTAGCGCAAGAGTCCGTCACTCCATACATTGCAATGCTGAATGGAGAGCCGATTGGGTATGCCCAGTCGTACGTTGCTCTTGGAAGCGGGGACGGATGGTGGGAAGAAGAAACCGATCCAGGAGTACGCGGAATAGACCAGTTACTGGCGAATGCATCACAACTGGGCAAAGGCTTGGGAACCGCGCTGGTTCGAGCTCTGGTTGAGTTGCTGTTCAATGATCCCGAGGTCACCAAGATCCAAACGGACCCGTCGCCGAGCAACTTGCGAGCGATCCGATGCTACGAGAAAGCGGGGTTTGAGAGGCAAGGTACCGTAACCACCCCAGATGGTCCAGCCGTGTACATGGTTCAAACACGCCAGGCATTCGAGCGAACACGCAGTGTTGCCTAA

>AY648355 Klebsiella pneumoniae plasmid pJHCMW1 mutant aminoglycoside 6'-N-acetyltransferase-IbK128A gene, complete cds.

GTGACCAACAGC---AACGATTCCGTCACACTGCGCCTCATGACTGAGCATGACCTTGCGATGCTCTATGAGTGGCTAAATCGATCTCATATCGTCGAGTGGTGGGGCGGAGAAGAAGCACGCCCGACACTTGCTGACGTACAGGAACAGTACTTGCCAAGCGTTTTAGCGCAAGAGTCCGTCACTCCATACATTGCAATGCTGAATGGAGAGCCGATTGGGTATGCCCAGTCGTACGTTGCTCTTGGAAGCGGGGACGGATGGTGGGAAGAAGAAACCGATCCAGGAGTACGCGGAATAGACCAGTTACTGGCGAATGCATCACAACTGGGCGCAGGCTTGGGAACCAAGCTGGTTCGAGCTCTGGTTGAGTTGCTGTTCAATGATCCCGAGGTCACCAAGATCCAAACGGACCCGTCGCCGAGCAACTTGCGAGCGATCCGATGCTACGAGAAAGCGGGGTTTGAGAGGCAAGGTACCGTAACCACCCCAGATGGTCCAGCCGTGTACATGGTTCAAACACGCCAGGCATTCGAGCGAACACGCAGTGTTGCCTAA

>AY648376 Klebsiella pneumoniae plasmid pJHCMW1 mutant aminoglycoside 6'-N-acetyltransferase-IbK150A gene, complete cds.

GTGACCAACAGC---AACGATTCCGTCACACTGCGCCTCATGACTGAGCATGACCTTGCGATGCTCTATGAGTGGCTAAATCGATCTCATATCGTCGAGTGGTGGGGCGGAGAAGAAGCACGCCCGACACTTGCTGACGTACAGGAACAGTACTTGCCAAGCGTTTTAGCGCAAGAGTCCGTCACTCCATACATTGCAATGCTGAATGGAGAGCCGATTGGGTATGCCCAGTCGTACGTTGCTCTTGGAAGCGGGGACGGATGGTGGGAAGAAGAAACCGATCCAGGAGTACGCGGAATAGACCAGTTACTGGCGAATGCATCACAACTGGGCAAAGGCTTGGGAACCAAGCTGGTTCGAGCTCTGGTTGAGTTGCTGTTCAATGATCCCGAGGTCACCGCGATCCAAACGGACCCGTCGCCGAGCAACTTGCGAGCGATCCGATGCTACGAGAAAGCGGGGTTTGAGAGGCAAGGTACCGTAACCACCCCAGATGGTCCAGCCGTGTACATGGTTCAAACACGCCAGGCATTCGAGCGAACACGCAGTGTTGCCTAA

>AF360387 Klebsiella pneumoniae plasmid pJHC-MW1 mutant AAC6'-IbK168A 6'-N-acetyltransferase gene, complete cds.

GTGACCAACAGC---AACGATTCCGTCACACTGCGCCTCATGACTGAGCATGACCTTGCGATGCTCTATGAGTGGCTAAATCGATCTCATATCGTCGAGTGGTGGGGCGGAGAAGAAGCACGCCCGACACTTGCTGACGTACAGGAACAGTACTTGCCAAGCGTTTTAGCGCAAGAGTCCGTCACTCCATACATTGCAATGCTGAATGGAGAGCCGATTGGGTATGCCCAGTCGTACGTTGCTCTTGGAAGCGGGGACGGATGGTGGGAAGAAGAAACCGATCCAGGAGTACGCGGAATAGACCAGTTACTGGCGAATGCATCACAACTGGGCAAAGGCTTGGGAACCAAGCTGGTTCGAGCTCTGGTTGAGTTGCTGTTCAATGATCCCGAGGTCACCAAGATCCAAACGGACCCGTCGCCGAGCAACTTGCGAGCGATCCGATGCTACGAGGCAGCGGGGTTTGAGAGGCAAGGTACCGTAACCACCCCAGATGGTCCAGCCGTGTACATGGTTCAAACACGCCAGGCATTCGAGCGAACACGCAGTGTTGCCTAA

>AY648372 Klebsiella pneumoniae plasmid pJHCMW1 mutant aminoglycoside 6'-N-acetyltransferase-IbP146A gene, complete cds.

GTGACCAACAGC---AACGATTCCGTCACACTGCGCCTCATGACTGAGCATGACCTTGCGATGCTCTATGAGTGGCTAAATCGATCTCATATCGTCGAGTGGTGGGGCGGAGAAGAAGCACGCCCGACACTTGCTGACGTACAGGAACAGTACTTGCCAAGCGTTTTAGCGCAAGAGTCCGTCACTCCATACATTGCAATGCTGAATGGAGAGCCGATTGGGTATGCCCAGTCGTACGTTGCTCTTGGAAGCGGGGACGGATGGTGGGAAGAAGAAACCGATCCAGGAGTACGCGGAATAGACCAGTTACTGGCGAATGCATCACAACTGGGCAAAGGCTTGGGAACCAAGCTGGTTCGAGCTCTGGTTGAGTTGCTGTTCAATGATGCCGAGGTCACCAAGATCCAAACGGACCCGTCGCCGAGCAACTTGCGAGCGATCCGATGCTACGAGAAAGCGGGGTTTGAGAGGCAAGGTACCGTAACCACCCCAGATGGTCCAGCCGTGTACATGGTTCAAACACGCCAGGCATTCGAGCGAACACGCAGTGTTGCCTAA

>AF360377 Klebsiella pneumoniae plasmid pJHC-MW1 mutant AAC6'-IbP157A 6'-N-acetyltransferase gene, complete cds.

GTGACCAACAGC---AACGATTCCGTCACACTGCGCCTCATGACTGAGCATGACCTTGCGATGCTCTATGAGTGGCTAAATCGATCTCATATCGTCGAGTGGTGGGGCGGAGAAGAAGCACGCCCGACACTTGCTGACGTACAGGAACAGTACTTGCCAAGCGTTTTAGCGCAAGAGTCCGTCACTCCATACATTGCAATGCTGAATGGAGAGCCGATTGGGTATGCCCAGTCGTACGTTGCTCTTGGAAGCGGGGACGGATGGTGGGAAGAAGAAACCGATCCAGGAGTACGCGGAATAGACCAGTTACTGGCGAATGCATCACAACTGGGCAAAGGCTTGGGAACCAAGCTGGTTCGAGCTCTGGTTGAGTTGCTGTTCAATGATCCCGAGGTCACCAAGATCCAAACGGACCCGTCGGCAAGCAACTTGCGAGCGATCCGATGCTACGAGAAAGCGGGGTTTGAGAGGCAAGGTACCGTAACCACCCCAGATGGTCCAGCCGTGTACATGGTTCAAACACGCCAGGCATTCGAGCGAACACGCAGTGTTGCCTAA

>AF360375 Klebsiella pneumoniae plasmid pJHC-MW1 mutant AAC6'-IbP155A 6'-N-acetyltransferase gene, complete cds.

GTGACCAACAGC---AACGATTCCGTCACACTGCGCCTCATGACTGAGCATGACCTTGCGATGCTCTATGAGTGGCTAAATCGATCTCATATCGTCGAGTGGTGGGGCGGAGAAGAAGCACGCCCGACACTTGCTGACGTACAGGAACAGTACTTGCCAAGCGTTTTAGCGCAAGAGTCCGTCACTCCATACATTGCAATGCTGAATGGAGAGCCGATTGGGTATGCCCAGTCGTACGTTGCTCTTGGAAGCGGGGACGGATGGTGGGAAGAAGAAACCGATCCAGGAGTACGCGGAATAGACCAGTTACTGGCGAATGCATCACAACTGGGCAAAGGCTTGGGAACCAAGCTGGTTCGAGCTCTGGTTGAGTTGCTGTTCAATGATCCCGAGGTCACCAAGATCCAAACGGACGCATCGCCGAGCAACTTGCGAGCGATCCGATGCTACGAGAAAGCGGGGTTTGAGAGGCAAGGTACCGTAACCACCCCAGATGGTCCAGCCGTGTACATGGTTCAAACACGCCAGGCATTCGAGCGAACACGCAGTGTTGCCTAA

>AY648343 Klebsiella pneumoniae plasmid pJHCMW1 mutant aminoglycoside 6'-N-acetyltransferase-IbR114A gene, complete cds.

GTGACCAACAGC---AACGATTCCGTCACACTGCGCCTCATGACTGAGCATGACCTTGCGATGCTCTATGAGTGGCTAAATCGATCTCATATCGTCGAGTGGTGGGGCGGAGAAGAAGCACGCCCGACACTTGCTGACGTACAGGAACAGTACTTGCCAAGCGTTTTAGCGCAAGAGTCCGTCACTCCATACATTGCAATGCTGAATGGAGAGCCGATTGGGTATGCCCAGTCGTACGTTGCTCTTGGAAGCGGGGACGGATGGTGGGAAGAAGAAACCGATCCAGGAGTAGCCGGAATAGACCAGTTACTGGCGAATGCATCACAACTGGGCAAAGGCTTGGGAACCAAGCTGGTTCGAGCTCTGGTTGAGTTGCTGTTCAATGATCCCGAGGTCACCAAGATCCAAACGGACCCGTCGCCGAGCAACTTGCGAGCGATCCGATGCTACGAGAAAGCGGGGTTTGAGAGGCAAGGTACCGTAACCACCCCAGATGGTCCAGCCGTGTACATGGTTCAAACACGCCAGGCATTCGAGCGAACACGCAGTGTTGCCTAA

>AY648363 Klebsiella pneumoniae plasmid pJHCMW1 mutant aminoglycoside 6'-N-acetyltransferase-IbR136A gene, complete cds.

GTGACCAACAGC---AACGATTCCGTCACACTGCGCCTCATGACTGAGCATGACCTTGCGATGCTCTATGAGTGGCTAAATCGATCTCATATCGTCGAGTGGTGGGGCGGAGAAGAAGCACGCCCGACACTTGCTGACGTACAGGAACAGTACTTGCCAAGCGTTTTAGCGCAAGAGTCCGTCACTCCATACATTGCAATGCTGAATGGAGAGCCGATTGGGTATGCCCAGTCGTACGTTGCTCTTGGAAGCGGGGACGGATGGTGGGAAGAAGAAACCGATCCAGGAGTACGCGGAATAGACCAGTTACTGGCGAATGCATCACAACTGGGCAAAGGCTTGGGAACCAAGCTGGTTGCAGCTCTGGTTGAGTTGCTGTTCAATGATCCCGAGGTCACCAAGATCCAAACGGACCCGTCGCCGAGCAACTTGCGAGCGATCCGATGCTACGAGAAAGCGGGGTTTGAGAGGCAAGGTACCGTAACCACCCCAGATGGTCCAGCCGTGTACATGGTTCAAACACGCCAGGCATTCGAGCGAACACGCAGTGTTGCCTAA

>AF360381 Klebsiella pneumoniae plasmid pJHC-MW1 mutant AAC6'-IbR161A 6'-N-acetyltransferase gene, complete cds.

GTGACCAACAGC---AACGATTCCGTCACACTGCGCCTCATGACTGAGCATGACCTTGCGATGCTCTATGAGTGGCTAAATCGATCTCATATCGTCGAGTGGTGGGGCGGAGAAGAAGCACGCCCGACACTTGCTGACGTACAGGAACAGTACTTGCCAAGCGTTTTAGCGCAAGAGTCCGTCACTCCATACATTGCAATGCTGAATGGAGAGCCGATTGGGTATGCCCAGTCGTACGTTGCTCTTGGAAGCGGGGACGGATGGTGGGAAGAAGAAACCGATCCAGGAGTACGCGGAATAGACCAGTTACTGGCGAATGCATCACAACTGGGCAAAGGCTTGGGAACCAAGCTGGTTCGAGCTCTGGTTGAGTTGCTGTTCAATGATCCCGAGGTCACCAAGATCCAAACGGACCCGTCGCCGAGCAACTTGGCAGCGATCCGATGCTACGAGAAAGCGGGGTTTGAGAGGCAAGGTACCGTAACCACCCCAGATGGTCCAGCCGTGTACATGGTTCAAACACGCCAGGCATTCGAGCGAACACGCAGTGTTGCCTAA

>AF360383 Klebsiella pneumoniae plasmid pJHC-MW1 mutant AAC6'-IbR164A 6'-N-acetyltransferase gene, complete cds.

GTGACCAACAGC---AACGATTCCGTCACACTGCGCCTCATGACTGAGCATGACCTTGCGATGCTCTATGAGTGGCTAAATCGATCTCATATCGTCGAGTGGTGGGGCGGAGAAGAAGCACGCCCGACACTTGCTGACGTACAGGAACAGTACTTGCCAAGCGTTTTAGCGCAAGAGTCCGTCACTCCATACATTGCAATGCTGAATGGAGAGCCGATTGGGTATGCCCAGTCGTACGTTGCTCTTGGAAGCGGGGACGGATGGTGGGAAGAAGAAACCGATCCAGGAGTACGCGGAATAGACCAGTTACTGGCGAATGCATCACAACTGGGCAAAGGCTTGGGAACCAAGCTGGTTCGAGCTCTGGTTGAGTTGCTGTTCAATGATCCCGAGGTCACCAAGATCCAAACGGACCCGTCGCCGAGCAACTTGCGAGCGATCGCATGCTACGAGAAAGCGGGGTTTGAGAGGCAAGGTACCGTAACCACCCCAGATGGTCCAGCCGTGTACATGGTTCAAACACGCCAGGCATTCGAGCGAACACGCAGTGTTGCCTAA

>AF360391 Klebsiella pneumoniae plasmid pJHC-MW1 mutant AAC6'-IbR173A 6'-N-acetyltransferase gene, complete cds.

GTGACCAACAGC---AACGATTCCGTCACACTGCGCCTCATGACTGAGCATGACCTTGCGATGCTCTATGAGTGGCTAAATCGATCTCATATCGTCGAGTGGTGGGGCGGAGAAGAAGCACGCCCGACACTTGCTGACGTACAGGAACAGTACTTGCCAAGCGTTTTAGCGCAAGAGTCCGTCACTCCATACATTGCAATGCTGAATGGAGAGCCGATTGGGTATGCCCAGTCGTACGTTGCTCTTGGAAGCGGGGACGGATGGTGGGAAGAAGAAACCGATCCAGGAGTACGCGGAATAGACCAGTTACTGGCGAATGCATCACAACTGGGCAAAGGCTTGGGAACCAAGCTGGTTCGAGCTCTGGTTGAGTTGCTGTTCAATGATCCCGAGGTCACCAAGATCCAAACGGACCCGTCGCCGAGCAACTTGCGAGCGATCCGATGCTACGAGAAAGCGGGGTTTGAGGCACAAGGTACCGTAACCACCCCAGATGGTCCAGCCGTGTACATGGTTCAAACACGCCAGGCATTCGAGCGAACACGCAGTGTTGCCTAA

>AY648371 Klebsiella pneumoniae plasmid pJHCMW1 mutant aminoglycoside 6'-N-acetyltransferase-IbD145A gene, complete cds.

GTGACCAACAGC---AACGATTCCGTCACACTGCGCCTCATGACTGAGCATGACCTTGCGATGCTCTATGAGTGGCTAAATCGATCTCATATCGTCGAGTGGTGGGGCGGAGAAGAAGCACGCCCGACACTTGCTGACGTACAGGAACAGTACTTGCCAAGCGTTTTAGCGCAAGAGTCCGTCACTCCATACATTGCAATGCTGAATGGAGAGCCGATTGGGTATGCCCAGTCGTACGTTGCTCTTGGAAGCGGGGACGGATGGTGGGAAGAAGAAACCGATCCAGGAGTACGCGGAATAGACCAGTTACTGGCGAATGCATCACAACTGGGCAAAGGCTTGGGAACCAAGCTGGTTCGAGCTCTGGTTGAGTTGCTGTTCAATGCTCCCGAGGTCACCAAGATCCAAACGGACCCGTCGCCGAGCAACTTGCGAGCGATCCGATGCTACGAGAAAGCGGGGTTTGAGAGGCAAGGTACCGTAACCACCCCAGATGGTCCAGCCGTGTACATGGTTCAAACACGCCAGGCATTCGAGCGAACACGCAGTGTTGCCTAA

>AY648346 Klebsiella pneumoniae plasmid pJHCMW1 mutant aminoglycoside 6'-N-acetyltransferase-IbD117A gene, complete cds.

GTGACCAACAGC---AACGATTCCGTCACACTGCGCCTCATGACTGAGCATGACCTTGCGATGCTCTATGAGTGGCTAAATCGATCTCATATCGTCGAGTGGTGGGGCGGAGAAGAAGCACGCCCGACACTTGCTGACGTACAGGAACAGTACTTGCCAAGCGTTTTAGCGCAAGAGTCCGTCACTCCATACATTGCAATGCTGAATGGAGAGCCGATTGGGTATGCCCAGTCGTACGTTGCTCTTGGAAGCGGGGACGGATGGTGGGAAGAAGAAACCGATCCAGGAGTACGCGGAATAGCCCAGTTACTGGCGAATGCATCACAACTGGGCAAAGGCTTGGGAACCAAGCTGGTTCGAGCTCTGGTTGAGTTGCTGTTCAATGATCCCGAGGTCACCAAGATCCAAACGGACCCGTCGCCGAGCAACTTGCGAGCGATCCGATGCTACGAGAAAGCGGGGTTTGAGAGGCAAGGTACCGTAACCACCCCAGATGGTCCAGCCGTGTACATGGTTCAAACACGCCAGGCATTCGAGCGAACACGCAGTGTTGCCTAA

>AY648370 Klebsiella pneumoniae plasmid pJHCMW1 mutant aminoglycoside 6'-N-acetyltransferase-IbN144A gene, complete cds.

GTGACCAACAGC---AACGATTCCGTCACACTGCGCCTCATGACTGAGCATGACCTTGCGATGCTCTATGAGTGGCTAAATCGATCTCATATCGTCGAGTGGTGGGGCGGAGAAGAAGCACGCCCGACACTTGCTGACGTACAGGAACAGTACTTGCCAAGCGTTTTAGCGCAAGAGTCCGTCACTCCATACATTGCAATGCTGAATGGAGAGCCGATTGGGTATGCCCAGTCGTACGTTGCTCTTGGAAGCGGGGACGGATGGTGGGAAGAAGAAACCGATCCAGGAGTACGCGGAATAGACCAGTTACTGGCGAATGCATCACAACTGGGCAAAGGCTTGGGAACCAAGCTGGTTCGAGCTCTGGTTGAGTTGCTGTTCGCTGATCCCGAGGTCACCAAGATCCAAACGGACCCGTCGCCGAGCAACTTGCGAGCGATCCGATGCTACGAGAAAGCGGGGTTTGAGAGGCAAGGTACCGTAACCACCCCAGATGGTCCAGCCGTGTACATGGTTCAAACACGCCAGGCATTCGAGCGAACACGCAGTGTTGCCTAA

>AY648350 Klebsiella pneumoniae plasmid pJHCMW1 mutant aminoglycoside 6'-N-acetyltransferase-IbN122A gene, complete cds.

GTGACCAACAGC---AACGATTCCGTCACACTGCGCCTCATGACTGAGCATGACCTTGCGATGCTCTATGAGTGGCTAAATCGATCTCATATCGTCGAGTGGTGGGGCGGAGAAGAAGCACGCCCGACACTTGCTGACGTACAGGAACAGTACTTGCCAAGCGTTTTAGCGCAAGAGTCCGTCACTCCATACATTGCAATGCTGAATGGAGAGCCGATTGGGTATGCCCAGTCGTACGTTGCTCTTGGAAGCGGGGACGGATGGTGGGAAGAAGAAACCGATCCAGGAGTACGCGGAATAGACCAGTTACTGGCGGCTGCATCACAACTGGGCAAAGGCTTGGGAACCAAGCTGGTTCGAGCTCTGGTTGAGTTGCTGTTCAATGATCCCGAGGTCACCAAGATCCAAACGGACCCGTCGCCGAGCAACTTGCGAGCGATCCGATGCTACGAGAAAGCGGGGTTTGAGAGGCAAGGTACCGTAACCACCCCAGATGGTCCAGCCGTGTACATGGTTCAAACACGCCAGGCATTCGAGCGAACACGCAGTGTTGCCTAA

>AF360379 Klebsiella pneumoniae plasmid pJHC-MW1 mutant AAC6'-IbN159A 6'-N-acetyltransferase gene, complete cds.

GTGACCAACAGC---AACGATTCCGTCACACTGCGCCTCATGACTGAGCATGACCTTGCGATGCTCTATGAGTGGCTAAATCGATCTCATATCGTCGAGTGGTGGGGCGGAGAAGAAGCACGCCCGACACTTGCTGACGTACAGGAACAGTACTTGCCAAGCGTTTTAGCGCAAGAGTCCGTCACTCCATACATTGCAATGCTGAATGGAGAGCCGATTGGGTATGCCCAGTCGTACGTTGCTCTTGGAAGCGGGGACGGATGGTGGGAAGAAGAAACCGATCCAGGAGTACGCGGAATAGACCAGTTACTGGCGAATGCATCACAACTGGGCAAAGGCTTGGGAACCAAGCTGGTTCGAGCTCTGGTTGAGTTGCTGTTCAATGATCCCGAGGTCACCAAGATCCAAACGGACCCGTCGCCGAGCGCATTGCGAGCGATCCGATGCTACGAGAAAGCGGGGTTTGAGAGGCAAGGTACCGTAACCACCCCAGATGGTCCAGCCGTGTACATGGTTCAAACACGCCAGGCATTCGAGCGAACACGCAGTGTTGCCTAA

>AY648345 Klebsiella pneumoniae plasmid pJHCMW1 mutant aminoglycoside 6'-N-acetyltransferase-IbI116A gene, complete cds.

GTGACCAACAGC---AACGATTCCGTCACACTGCGCCTCATGACTGAGCATGACCTTGCGATGCTCTATGAGTGGCTAAATCGATCTCATATCGTCGAGTGGTGGGGCGGAGAAGAAGCACGCCCGACACTTGCTGACGTACAGGAACAGTACTTGCCAAGCGTTTTAGCGCAAGAGTCCGTCACTCCATACATTGCAATGCTGAATGGAGAGCCGATTGGGTATGCCCAGTCGTACGTTGCTCTTGGAAGCGGGGACGGATGGTGGGAAGAAGAAACCGATCCAGGAGTACGCGGAGCAGACCAGTTACTGGCGAATGCATCACAACTGGGCAAAGGCTTGGGAACCAAGCTGGTTCGAGCTCTGGTTGAGTTGCTGTTCAATGATCCCGAGGTCACCAAGATCCAAACGGACCCGTCGCCGAGCAACTTGCGAGCGATCCGATGCTACGAGAAAGCGGGGTTTGAGAGGCAAGGTACCGTAACCACCCCAGATGGTCCAGCCGTGTACATGGTTCAAACACGCCAGGCATTCGAGCGAACACGCAGTGTTGCCTAA

>AY648377 Klebsiella pneumoniae plasmid pJHCMW1 mutant aminoglycoside 6'-N-acetyltransferase-IbI151A gene, complete cds.

GTGACCAACAGC---AACGATTCCGTCACACTGCGCCTCATGACTGAGCATGACCTTGCGATGCTCTATGAGTGGCTAAATCGATCTCATATCGTCGAGTGGTGGGGCGGAGAAGAAGCACGCCCGACACTTGCTGACGTACAGGAACAGTACTTGCCAAGCGTTTTAGCGCAAGAGTCCGTCACTCCATACATTGCAATGCTGAATGGAGAGCCGATTGGGTATGCCCAGTCGTACGTTGCTCTTGGAAGCGGGGACGGATGGTGGGAAGAAGAAACCGATCCAGGAGTACGCGGAATAGACCAGTTACTGGCGAATGCATCACAACTGGGCAAAGGCTTGGGAACCAAGCTGGTTCGAGCTCTGGTTGAGTTGCTGTTCAATGATCCCGAGGTCACCAAGGCCCAAACGGACCCGTCGCCGAGCAACTTGCGAGCGATCCGATGCTACGAGAAAGCGGGGTTTGAGAGGCAAGGTACCGTAACCACCCCAGATGGTCCAGCCGTGTACATGGTTCAAACACGCCAGGCATTCGAGCGAACACGCAGTGTTGCCTAA

>AF360382 Klebsiella pneumoniae plasmid pJHC-MW1 mutant AAC6'-IbI163A 6'-N-acetyltransferase gene, complete cds.

GTGACCAACAGC---AACGATTCCGTCACACTGCGCCTCATGACTGAGCATGACCTTGCGATGCTCTATGAGTGGCTAAATCGATCTCATATCGTCGAGTGGTGGGGCGGAGAAGAAGCACGCCCGACACTTGCTGACGTACAGGAACAGTACTTGCCAAGCGTTTTAGCGCAAGAGTCCGTCACTCCATACATTGCAATGCTGAATGGAGAGCCGATTGGGTATGCCCAGTCGTACGTTGCTCTTGGAAGCGGGGACGGATGGTGGGAAGAAGAAACCGATCCAGGAGTACGCGGAATAGACCAGTTACTGGCGAATGCATCACAACTGGGCAAAGGCTTGGGAACCAAGCTGGTTCGAGCTCTGGTTGAGTTGCTGTTCAATGATCCCGAGGTCACCAAGATCCAAACGGACCCGTCGCCGAGCAACTTGCGAGCGGCACGATGCTACGAGAAAGCGGGGTTTGAGAGGCAAGGTACCGTAACCACCCCAGATGGTCCAGCCGTGTACATGGTTCAAACACGCCAGGCATTCGAGCGAACACGCAGTGTTGCCTAA

>AF012279 Plasmid pJHC-MW1 transposon Tn1331 mutant aminoglycoside 6'-N-acetyltransferase gene, complete cds.

GTGACCAACAGC---AACGATTCCGTCACACTGCGCCTCATGACTGAGCATGACCTTGCGATGCTCTATGAGTGGCTAAATCGATCTCATATCGTCGAGTGGTGGGGCGGAGAAGAAGCACGCCCGACACTTGCTGACGTACAGGAACAGTACTTGCCAAGCGTTTTAGCGCAAGAGTCCGTCACTCCATACATTGCAATGCTGAATGGAGAGCCGATTGGGTATGCCCAGTCGTACGTTGCTCTTGGAAGCGGGGACGGATGGTGGGAAGAAGAAACCGATCCAGGAGTACGCGGAATAGACCAGTTACTGGCGAATGCATCACAACTGGGCAAAGGCTTGGGAACCAAGCTGGTTCGAGCTCTGGTTGAGTTGCTGTTCAATGATCCCGAGGTCACCAAGATCCAAACGGACCCGTCGCCGAGCAACTTGCGAGCGATCCGATGCTACGAGAAAGCGGGGCTTGAGAGGCAAGGTACCGTAACCACCCCAGATGGTCCAGCCGTGTACATGGTTCAAACACGCCAGGCATTCGAGCGAACACGCAGTGTTGCCTAA

>AF360385 Klebsiella pneumoniae plasmid pJHC-MW1 mutant AAC6'-IbY166A 6'-N-acetyltransferase gene, complete cds.

GTGACCAACAGC---AACGATTCCGTCACACTGCGCCTCATGACTGAGCATGACCTTGCGATGCTCTATGAGTGGCTAAATCGATCTCATATCGTCGAGTGGTGGGGCGGAGAAGAAGCACGCCCGACACTTGCTGACGTACAGGAACAGTACTTGCCAAGCGTTTTAGCGCAAGAGTCCGTCACTCCATACATTGCAATGCTGAATGGAGAGCCGATTGGGTATGCCCAGTCGTACGTTGCTCTTGGAAGCGGGGACGGATGGTGGGAAGAAGAAACCGATCCAGGAGTACGCGGAATAGACCAGTTACTGGCGAATGCATCACAACTGGGCAAAGGCTTGGGAACCAAGCTGGTTCGAGCTCTGGTTGAGTTGCTGTTCAATGATCCCGAGGTCACCAAGATCCAAACGGACCCGTCGCCGAGCAACTTGCGAGCGATCCGATGCGCTGAGAAAGCGGGGTTTGAGAGGCAAGGTACCGTAACCACCCCAGATGGTCCAGCCGTGTACATGGTTCAAACACGCCAGGCATTCGAGCGAACACGCAGTGTTGCCTAA

>EF375699 Acinetobacter baumannii strain YMC 05/4/P498 class 1 integron DNA integrase (intI1) gene, partial cds, AacA4 (aacA4), metallo-beta-lactamase IMP-1 (blaIMP-1), and OXA-2 beta-lactamase (blaOXA-2) genes, complete cds, and quaternary ammonium compound-resistance protein (qacEdelta1) gene, partial cds.

ATGACCAACAGC---AACGATTCCGTCACACTGCGCCTCATGACTGAGCATGACCTTGCGATGCTCTATGAGTGGCTAAATCGATCTCATATCGTCGAGTGGTGGGGCGGAGAAGAAGCACGCCCGACACTTGCTGACGTACAGGAACAGTACTTGCCAAGCGTTTTAGCGCAAGAGTCCGTCACTCCATACATTGCAATGCTGAATGGAGAGCCGATTGGGTATGCCCAGTCGTACGTTGCTCTTGGAAGCGGGGACGGATGGTGGGAAGAAGAAACCGATCCAGGAGTACGCGGAATAGACCAGTTACTGGCGAATGCATCACAACTGGGCAAAGGCTTGGGAACCAAGCTGGTTCGAGCTCTGGTTGAGTTGCTGTTCAATGATCCCGAGGTCACCAAGATCCAAACGGACCCGTCGCCGAGCAACTTGCGAGCGATCCGATGCTACGAGAAAGCGGGGTTTGAGAGGCAAGGTACCGTAACCACCCCAGATGGTCCAGCCGTGTACATGGTTCAAACACGCCAGGCATTCGAGCGAACACGCAGTGTTGCCTAA

>EU014166 Acinetobacter junii strain YMC 06/10/R1547-IMP-1/VIM-2 class I integron DNA integrase (intI1) gene, partial cds, AACA4 (aacA4), metallo-beta-lactamase IMP-1 (blaIMP-1), and OXA-2 beta-lactamase (blaOXA-2) genes, complete cds, and quaternary ammonium compound-resistance protein (qacEdelta1) gene, partial cds.

ATGACCAACAGC---AACGATTCCGTCACACTGCGCCTCATGACTGAGCATGACCTTGCGATGCTCTATGAGTGGCTAAATCGATCTCATATCGTCGAGTGGTGGGGCGGAGAAGAAGCACGCCCGACACTTGCTGACGTACAGGAACAGTACTTGCCAAGCGTTTTAGCGCAAGAGTCCGTCACTCCATACATTGCAATGCTGAATGGAGAGCCGATTGGGTATGCCCAGTCGTACGTTGCTCTTGGAAGCGGGGACGGATGGTGGGAAGAAGAAACCGATCCAGGAGTACGCGGAATAGACCAGTTACTGGCGAATGCATCACAACTGGGCAAAGGCTTGGGAACCAAGCTGGTTCGAGCTCTGGTTGAGTTGCTGTTCAATGATCCCGAGGTCACCAAGATCCAAACGGACCCGTCGCCGAGCAACTTGCGAGCGATCCGATGCTACGAGAAAGCGGGGTTTGAGAGGCAAGGTACCGTAACCACCCCAGATGGTCCAGCCGTGTACATGGTTCAAACACGCCAGGCATTCGAGCGAACACGCAGTGTTGCCTAA

>AB195796 Pseudomonas aeruginosa class I integron DNA, complete sequence.

GTGACCAACAGC---AACGATTCCGTCACACTGCGCCTCATGACTGAGCATGACCTTGCGATGCTCTATGAGTGGCTAAATCGATCTCATATCGTCGAGTGGTGGGGCGGAGAAGAAGCACGCCCGACACTTGCTGACGTACAGGAACAGTACTTGCCAAGCGTTTTAGCGCAAGAGTCCGTCACTCCATACATTGCAATGCTGAATGGAGAGCCGATTGGGTATGGCCAGTCGTACGTTGCTCTTGGAAGCGGGGACGGATGGTGGGAAGAAGAAACCGATCCAGGAGTACGCGGAATAGACCAGTCACTGGCGAATGCATCACAACTGGGCAAAGGCTTGGGAACCAAGCTGGTTCGAGCACTGGTTGAGTTGCTGTTCAATGATCCCGAGGTCACCAAGATCCAAACGGACCCGTCGCCGAGCAACTTGCGAGCGATCCGATGCTACGAGAAAGCGGGGTTTGAGAGGCAAGGTACCGTAACCACCCCAGATGGTCCAGCCGTGTACATGGTTCAAACACGCCAGGCATTCGAGCGAACACGCAGTGATGCCTAA

>AJ697861 Gram-negative bacterium TR-59 aac6'-Ib gene for aminoglycoside 6-N-acetyltransferase.

GTGACCAACAGC---AACGATTCCGTCACACTGCGCCTCATGACTGAGCATGACCTTGCGATGCTCTATGAGTGGCTAAATCGATCTCATATCGTCGAGTGGTGGGGCGGAGAAGAAGCACGCCCGACACTTGCTGACGTACAGGAACAGTACTTGCCAAGCGTTTTAGCGCAAGAGTCCGTCACTCCATACATTGCAATGCTGAATGGAGAGCCGATTGGGTATGCCCAGTCGTACGTTGCTCTTGGAAGCGGGGACGG--TGTGGGAAGAAGATACCGATCCAGGAGTACGCGGAATAGACCAGTTTATGGCGAATGCATCACAACTGGGCAAAGGCTTGGGAACCAAGTTGGTTCGAGCTCTGGTTGAGTTGCTGTTCAATGATCCCGAGGTCACCAAGATCCAAACGGACCCGTCGCCGAGCAACTTGCGAGCGATCCGATGCTACGAGAAAGCGGGGTTTGAGAGGCAAGGTACCGTAACCACCCCAGATGGTCCAGCCGTGTACATGGTTCAAACACGCCAGGCATTCGAGCGAACACGCAGTGATGCCTAA

>EF368053 Pseudomonas aeruginosa isolate PaTBIZP2 class I integron aminoglycoside 6'-N-acetyltyransferase (aac(6)-Ib), streptomycin 3''-adenyl transferase (aadA2), and quaternary ammonium compound-resistance protein (qacEdelta1) genes, complete cds.

GTGACCAACAGC---AACGATTCCGTCACACTGCGCCTCATGACTGAGCATGACCTTGCGATGCTCTATGAGTGGCTAAATCGATCTCATATCGTCGAGTGGTGGGGCGGAGAAGAAGCACGCCCGACACTTGCTGACGTACAGGAACAGTACTTGCCAAGCGTTTTAGCGCAAGAGTCCGTCACTCCATACATTGCAATGCTGAATGGAGAGCCGATTGGGTATGCCCAGTCGTACGTTGCTCTTGGAAGCGGGGACGGATGGTGGGAAGAAGAAACCGATCCAGGAGTACGCGGAATAGACCAGTCACTGGCGAATGCATCACAACTGGGCAAAGGCTTGGGAACCAAGCTGGTTCGAGCTCTGGTTGAGTTGCTGTTCAATGATCCCGAGGTCACCAAGATCCAAACGGACCCGTCGCCGAGCAACTTGCGAGCGATCCGATGCTACGAGAAAGTGGGGTTTGAGAGGCAAGGTACCGTAACCACCCCAGATGGTCCAGCCGTGTACATGGTTCAAACACGCCAGGCATTCGAGCGAACACGCAGTGATGCCTAA

>AY648369 Klebsiella pneumoniae plasmid pJHCMW1 mutant aminoglycoside 6'-N-acetyltransferase-IbF143A gene, complete cds.

GTGACCAACAGC---AACGATTCCGTCACACTGCGCCTCATGACTGAGCATGACCTTGCGATGCTCTATGAGTGGCTAAATCGATCTCATATCGTCGAGTGGTGGGGCGGAGAAGAAGCACGCCCGACACTTGCTGACGTACAGGAACAGTACTTGCCAAGCGTTTTAGCGCAAGAGTCCGTCACTCCATACATTGCAATGCTGAATGGAGAGCCGATTGGGTATGCCCAGTCGTACGTTGCTCTTGGAAGCGGGGACGGATGGTGGGAAGAAGAAACCGATCCAGGAGTACGCGGAATAGACCAGTTACTGGCGAATGCATCACAACTGGGCAAAGGCTTGGGAACCAAGCTGGTTCGAGCTCTGGTTGAGTTGCTGGCCAATGATCCCGAGGTCACCAAGATCCAAACGGACCCGTCGCCGAGCAACTTGCGAGCGATCCGATGCTACGAGAAAGCGGGGTTTGAGAGGCAAGGTACCGTAACCACCCCAGATGGTCCAGCCGTGTACATGGTTCAAACACGCCAGGCATTCGAGCGAACACGCAGTGTTGCCTAA

>AF360389 Klebsiella pneumoniae plasmid pJHC-MW1 mutant AAC6'-IbF171A 6'-N-acetyltransferase gene, complete cds.

GTGACCAACAGC---AACGATTCCGTCACACTGCGCCTCATGACTGAGCATGACCTTGCGATGCTCTATGAGTGGCTAAATCGATCTCATATCGTCGAGTGGTGGGGCGGAGAAGAAGCACGCCCGACACTTGCTGACGTACAGGAACAGTACTTGCCAAGCGTTTTAGCGCAAGAGTCCGTCACTCCATACATTGCAATGCTGAATGGAGAGCCGATTGGGTATGCCCAGTCGTACGTTGCTCTTGGAAGCGGGGACGGATGGTGGGAAGAAGAAACCGATCCAGGAGTACGCGGAATAGACCAGTTACTGGCGAATGCATCACAACTGGGCAAAGGCTTGGGAACCAAGCTGGTTCGAGCTCTGGTTGAGTTGCTGTTCAATGATCCCGAGGTCACCAAGATCCAAACGGACCCGTCGCCGAGCAACTTGCGAGCGATCCGATGCTACGAGAAAGCGGGGGCAGAGAGGCAAGGTACCGTAACCACCCCAGATGGTCCAGCCGTGTACATGGTTCAAACACGCCAGGCATTCGAGCGAACACGCAGTGTTGCCTAA

>AF360384 Klebsiella pneumoniae plasmid pJHC-MW1 mutant AAC6'-IbC165A 6'-N-acetyltransferase gene, complete cds.

GTGACCAACAGC---AACGATTCCGTCACACTGCGCCTCATGACTGAGCATGACCTTGCGATGCTCTATGAGTGGCTAAATCGATCTCATATCGTCGAGTGGTGGGGCGGAGAAGAAGCACGCCCGACACTTGCTGACGTACAGGAACAGTACTTGCCAAGCGTTTTAGCGCAAGAGTCCGTCACTCCATACATTGCAATGCTGAATGGAGAGCCGATTGGGTATGCCCAGTCGTACGTTGCTCTTGGAAGCGGGGACGGATGGTGGGAAGAAGAAACCGATCCAGGAGTACGCGGAATAGACCAGTTACTGGCGAATGCATCACAACTGGGCAAAGGCTTGGGAACCAAGCTGGTTCGAGCTCTGGTTGAGTTGCTGTTCAATGATCCCGAGGTCACCAAGATCCAAACGGACCCGTCGCCGAGCAACTTGCGAGCGATCCGAGCCTACGAGAAAGCGGGGTTTGAGAGGCAAGGTACCGTAACCACCCCAGATGGTCCAGCCGTGTACATGGTTCAAACACGCCAGGCATTCGAGCGAACACGCAGTGTTGCCTAA

>AY458016 Escherichia coli plasmid pC15-1a, complete sequence.

GTGACCAACAGC---AACGATTCCGTCACACTGCGCCTCATGACTGAGCATGACCTTGCGATGCTCTATGAGTGGCTAAATCGATCTCATATCGTCGAGTGGTGGGGCGGAGAAGAAGCACGCCCGACACTTGCTGACGTACAGGAACAGTACTTGCCAAGCGTTTTAGCGCAAGAGTCCGTCACTCCATACATTGCAATGCTGAATGGAGAGCCGATTGGGTATGCCCAGTCGTACGTTGCTCTTGGAAGCGGGGACGGACGGTGGGAAGAAGAAACCGATCCAGGAGTACGCGGAATAGACCAGTTACTGGCGAATGCATCACAACTGGGCAAAGGCTTGGGAACCAAGCTGGTTCGAGCTCTGGTTGAGTTGCTGTTCAATGATCCCGAGGTCACCAAGATCCAAACGGACCCGTCGCCGAGCAACTTGCGAGCGATCCGATGCTACGAGAAAGCGGGGTTTGAGAGGCAAGGTACCGTAACCACCCCATATGGTCCAGCCGTGTACATGGTTCAAACACGCCAGGCATTCGAGCGAACACGCAGTGATGCCTAA

>DQ303918 Escherichia coli fluroquinolone acetylating aminoglycoside acetyltransferase (aac(6`)-Ib-cr) gene, complete cds.

GTGACCAACAGC---AACGATTCCGTCACACTGCGCCTCATGACTGAGCATGACCTTGCGATGCTCTATGAGTGGCTAAATCGATCTCATATCGTCGAGTGGTGGGGCGGAGAAGAAGCACGCCCGACACTTGCTGACGTACAGGAACAGTACTTGCCAAGCGTTTTAGCGCAAGAGTCCGTCACTCCATACATTGCAATGCTGAATGGAGAGCCGATTGGGTATGCCCAGTCGTACGTTGCTCTTGGAAGCGGGGACGGACGGTGGGAAGAAGAAACCGATCCAGGAGTACGCGGAATAGACCAGTTACTGGCGAATGCATCACAACTGGGCAAAGGCTTGGGAACCAAGCTGGTTCGAGCTCTGGTTGAGTTGCTGTTCAATGATCCCGAGGTCACCAAGATCCAAACGGACCCGTCGCCGAGCAACTTGCGAGCGATCCGATGCTACGAGAAAGCGGGGTTTGAGAGGCAAGGTACCGTAACCACCCCATATGGTCCAGCCGTGTACATGGTTCAAACACGCCAGGCATTCGAGCGAACACGCAGTGATGCCTAA

>EF100892 Klebsiella pneumoniae fluroquinolone acetylating aminoglycoside acetyltransferase (aac(6')-Ib-cr) gene, complete cds.

GTGACCAACAGC---AACGATTCCGTCACACTGCGCCTCATGACTGAGCATGACCTTGCGATGCTCTATGAGTGGCTAAATCGATCTCATATCGTCGAGTGGTGGGGCGGAGAAGAAGCACGCCCGACACTTGCTGACGTACAGGAACAGTACTTGCCAAGCGTTTTAGCGCAAGAGTCCGTCACTCCATACATTGCAATGCTGAATGGAGAGCCGATTGGGTATGCCCAGTCGTACGTTGCTCTTGGAAGCGGGGACGGACGGTGGGAAGAAGAAACCGATCCAGGAGTACGCGGAATAGACCAGTTACTGGCGAATGCATCACAACTGGGCAAAGGCTTGGGAACCAAGCTGGTTCGAGCTCTGGTTGAGTTGCTGTTCAATGATCCCGAGGTCACCAAGATCCAAACGGACCCGTCGCCGAGCAACTTGCGAGCGATCCGATGCTACGAGAAAGCGGGGTTTGAGAGGCAAGGTACCGTAACCACCCCATATGGTCCAGCCGTGTACATGGTTCAAACACGCCAGGCATTCGAGCGAACACGCAGTGATGCCTAA

>AY259086 Escherichia coli plasmid pHSH2 encoded class 1 integron In37, complete sequence.

GTGACCAACAGC---AACGATTCCGTCACACTGCGCCTCATGACTGAGCATGACCTTGCGATGCTCTATGAGTGGCTAAATCGATCTCATATCGTCGAGTGGTGGGGCGGAGAAGAAGCACGCCCGACACTTGCTGACGTACAGGAACAGTACTTGCCAAGCGTTTTAGCGCAAGAGTCCGTCACTCCATACATTGCAATGCTGAATGGAGAGCCGATTGGGTATGCCCAGTCGTACGTTGCTCTTGGAAGCGGGGACGGACGGTGGGAAGAAGAAACCGATCCAGGAGTACGCGGAATAGACCAGTTACTGGCGAATGCATCACAACTGGGCAAAGGCTTGGGAACCAAGCTGGTTCGAGCTCTGGTTGAGTTGCTGTTCAATGATCCCGAGGTCACCAAGATCCAAACGGACCCGTCGCCGAGCAACTTGCGAGCGATCCGATGCTACGAGAAAGCGGGGTTTGAGAGGCAAGGTACCGTAACCACCCCATATGGTCCAGCCGTGTACATGGTTCAAACACGCCAGGCATTCGAGCGAACACGCAGTGATGCCTAA

>EF415651 Escherichia coli strain DR22806 class 1 integron integrase (intI1) gene, partial cds, aminoglycoside acetyltransferase (aac(6')-Ib-cr), OXA-1 beta-lactamase (blaOXA-1), chloramphenicol acetyltransferase (catB3), and rifampin ADP-ribosylating transferase (arr-3) genes, complete cds, and quaternary ammonium compound-resistance protein (qacEdeltal) gene, partial cds.

GTGACCAACAGC---AACGATTCCGTCACACTGCGCCTCATGACTGAGCATGACCTTGCGATGCTCTATGAGTGGCTAAATCGATCTCATATCGTCGAGTGGTGGGGCGGAGAAGAAGCACGCCCGACACTTGCTGACGTACAGGAACAGTACTTGCCAAGCGTTTTAGCGCAAGAGTCCGTCACTCCATACATTGCAATGCTGAATGGAGAGCCGATTGGGTATGCCCAGTCGTACGTTGCTCTTGGAAGCGGGGACGGACGGTGGGAAGAAGAAACCGATCCAGGAGTACGCGGAATAGACCAGTTACTGGCGAATGCATCACAACTGGGCAAAGGCTTGGGAACCAAGCTGGTTCGAGCTCTGGTTGAGTTGCTGTTCAATGATCCCGAGGTCACCAAGATCCAAACGGACCCGTCGCCGAGCAACTTGCGAGCGATCCGATGCTACGAGAAAGCGGGGTTTGAGAGGCAAGGTACCGTAACCACCCCATATGGTCCAGCCGTGTACATGGTTCAAACACGCCAGGCATTCGAGCGAACACGCAGTGATGCCTAA

>AY136758 Salmonella typhimurium plasmid pSTI1 class I integron integrase (intI1) gene, partial cds; aminoglycoside 6-N-acetyltransferase type Ib11 (aac(6')-Ib) gene, complete cds; and unknown gene.

CATATCAACAGC---AACGATTCCGTCACACTGCGCCTCATGACTGAGCATGACCTTGCGATGCTCTATGAGTGGCTAAATCGATCTCATATCGTCGAGTGGTGGGGCGGAGAAGAAGCACGCCCGACACTTGCTGACGTACAGGAACAGTACTTGCCAAGCGTTTTAGCGCAAGAGTCCGTCACTCCATACATTGCAATGCTGAATGGAGAGCCGATTGGGTATGCCCAGTCGTACGTTGCTCTTGGAAGCGGGGACGGATGGTGGGAAGAAGAAACCGATCCAGGAGTACGCGGAATAGACCTGTCACTGGCGAATGCATCACAACTGGGCAAAGGCTTGGGAACCAAGCTGGTTCGAGCTCTGGTTGAGTTGCTGTTCAATGATCCCGAGGTCACCAAGATCCAAACGGACCCGTCGCCGAGCAACTTGCGAGCGATCCGATGCTACGAGAAAGCGGGGTTTGAGAGGCAAGGTACCGTAACCACCCCAGATGGTCCAGCCGTGTACATGGTTCAAACACGCCAGGCATTCGAGCGAACACGCAGTGATGCCTAA

>AF315786 Pseudomonas aeruginosa class I integron integrase (intI1) gene, partial cds; and aminoglycoside acetyltransferase (aac(6')-Ib) and restricted-spectrum beta-lactamase OXA-35 (bla) genes, complete cds.

GTGACCAACAGC---AACGATTCCGTCACACTGCGCCTCATGACTGAGCATGACCTTGCGATGCTCTATGAGTGGCTAAATCGATCTCATATCGTCGAGTGGTGGGGCGGAGAAGAAGCACGCCCGACACTTGCTGACGTACAGGAACAGTACTTGCCAAGCGTTTTAGCGCAAGAGTCCGTCACTCCATACATTGCAATGCTGAATGGAGAGCCGATTGGGTATGCCCAGTCGTACGTTGCTCTTGGAAGCGGGGACGGATGGTGGGAAGAAGAAACCGATCCAGGAGTACGCGGAATAGACCAGTCACTGGCGAATGCATCACAACTGGGCAAAGGCTTGGGAACCAAGCTGGTTCGAGCTCTGGTTGAGTTGCTGTTCAATGATCCCGAGGTCGCCAGGATCCAAACGGACCCGTCGCCGAGCAACTTGCGAGCGATCCGATGCTACGAGAAAGCGGGGTTTGAGAGGCAAGGTACCGTAACCACCCCAGATGGTCCAGCCGTGTACATGGTTCAAACACGCCAGGCATTCGAGCGAACACGCAGTGATGCCTAA

>DQ153217 Klebsiella pneumoniae isolate YMC 03/7/P267 class I integron DNA integrase IntI1 (intI1) gene, partial cds, AACA4 (aac6'-I) and metallo-beta-lactamase VIM-2 (blaVIM-2) genes, complete cds, and unknown genes.

GTGACCAACAGC---AACGATTCCGTCACACTGCGCCTCATGACTGAGCATGACCTTGCGATGCTCTATGAGTGGCTAAATCGATCTCATATCGTCGAGTGGTGGGGCGGAGAAGAAGCACGCCCGACACTTGCTGACGTACAGGAACAGTACTTGCCAAGCGTTTTAGCGCAAGAGTCCGTCACTCCATACATTGCAATGCTGAATGGAGAGCCGATTGGGTATGCCCAGTCGTACGTTGCTCTTGGAAGCGGGGACGGATGGTGGGAAGAAGAAACCGATCCAGGAGTACGCGGAATAGACCTGTCACTGGCGAATGCATCACAACTGGGCAAAGGCTTGGGAACCAAGCTGGTTCGAGCTCTGGTTGAGTTGCTGTTCAATGATCCCGAGGTCACCAAGATCCAAACGGACCCGTCGCCGAGCAACTTGCGAGCGATCCGATGCTACGAGAAAGCGGGGTTTGAGAGGCAAGGTACCGTAACCACCCCAGATGGTCCAGCCGTGTACATGGTTCAAACACGCCAGGCATTCGAGCGAACACGCAGTGTTGCCTAA

>AY219651 Klebsiella pneumoniae transposon class 3 integron IntI3 integrase (intI3), GES-1 extended-spectrum beta-lactamase (blaGES-1), and OXA/AAC(6')-Ib aminoglycoside 6'-N-acetyltransferase (blaOXA/aac(6')-Ib) genes, complete cds; and putative replication protein RepC (repC) gene, partial cds.

ATGAAAACATTT---AACGATTCCGTCACACTGCGCCTCATGACTGAGCATGACCTTGCGATGCTCTATGAGTGGCTAAATCGATCTCATATCGTCGAGTGGTGGGGCGGAGAAGAAGCACGCCCGACACTTGCTGACGTACAGGAACAGTACTTGCCAAGCGTTTTAGCGCAAGAGTCCGTCACTCCATACATTGCAATGCTGAATGGAGAGCCGATTGGGTATGCCCAGTCGTACGTTGCTCTTGGAAGCGGGGACGGATGGTGGGAAGAAGAAACCGATCCAGGAGTACGCGGAATAGACCAGTCACTGGCGAATGCATCACAACTGGGCAAAGGCTTGGGAACCAAGCTGGTTCGAGCTCTGGTTGAGTTGCTGTTCAATGATCCCGAGGTCACCAAGATCCAAACGGACCCGTCGCCGAGCAACTTGCGAGCGATCCGATGCTACGAGAAAGCGGGGTTTGAGAGGCAAGGTACCGTAACCACCCCAGATGGTCCAGCCGTGTACATGGTTCAAACACGCCAGGCATTCGAGCGAACACGCAGTGATGCCTAA

>AY507153 Pseudomonas aeruginosa strain 4122 class I integron, partial sequence.

ATGACCAACAGC---AACGATTCCGTCACACTGCGCCTCATGACTGAGCATGACCTTGCGATGCTCTATGAGTGGCTAAATCGATCTCATATCGTCGAGTGGTGGGGCGGAGAAGAAGCACGCCCGACACTTGCTGACGTACAGGAACAGTACTTGCCAAGCGTTTTAGCGCAAGAGTCCGTCACTCCATACATTGCAATGCTGAATGGAGAGCCGATTGGGTATGCCCAGTCGTACGTTGCTCTTGGAAGCGGGGACGGATGGTGGGAAGAAGAAACCGATCCAGGAGTACGCGGAATAGACCAGTTACTGGCGAATGCATCACAACTGGGCAAAGGCTTGGGAACCAAGCTGGTTCGAGCTCTGGTTGAGTTGCTGTTCAATGATCCCGAGGTCACCAAGATCCAAACGGACCCGTCGCCGAGCAACTTGCGAGCGATCCGATGCTACGAGAAAGCGGGGTTTGAGAGGCAAGGTACCGTAACCACCCCAGATGGTCCAGCCGTGTACATGGTTCAAACACGCCAGGCATTCGAGCGAACACGCAGTGATGCCTAA

>AJ009820 Salmonella typhimurium plasmid pSEM partial intI1 gene, aacC4 and aacC1 genes, ORF X and ORF X', aadA1a, qacEdelta1 and sul1 genes, ORF 5 and ORF 6 and partial tnpA gene.

GTGACCAACAGC---AACGATTCCGTCACACTGCGCCTCATGACTGAGCATGACCTTGCGATGCTCTATGAGTGGCTAAATCGATCTCATATCGTCGAGTGGTGGGGCGGAGAAGAAGCACGCCCGACACTTGCCGACGTACAGGAACAGTACTTGCCAAGCGTTTTAGCGCAAGAGTCCGTCACTCCATACATTGCAATGCTGAATGGAGAGCCGATTGGGTATGCCCAGTCGTACGTTGCTCTTGGAAGCGGGGACGGATGGTGGGAAGAAGAAACCGATCCAGGAGTACGCGGAATAGACCAGTTACTGGCGAATGCATCACAACTGGGCAAAGGCTTGGGAACCAAGCTGGTTCGAGCTCTGGTTGAGTTGCTGTTCAATGATCCCGAGGTCACCAAGATCCAAACGGACCCGTCGCCGAGCAACTTGCGAGCGATCCGATGCTACGAGAAAGCGGGGTTTGAGAGGCAAGGTACCGTAACCACCCCAGATGGTCCAGCCGTGTACATGGTTCAAACACGCCAGGCATTCGAGCGAACACGCGATGATGCCTAA

>AY920928 Pseudomonas aeruginosa transposon Tn5393C TnpA (tnpA) gene, partial cds; and integron In109 integrase (intI1) gene, complete sequence, GES-9 (bla(GES-9)) gene, complete cds, insertion sequence ISPa21, complete sequence, AAC(6')-Ib (aacA4) and AADB (aadB) genes, complete cds, insertion sequence ISPa21, complete sequence, and unknown gene.

GTGACCAACAGC---AACGATTCCGTAACACTGCGCCTCATGACTGAGCATGACCTTGCGATGCTCTATGAGTGGCTAAATCGATCTCATATCGTCGAGTGGTGGGGCGGAGAAGAAGCACGCCCGACACTTGCTGACGTACAGGAACAGTACTTGCCAAGCGTTTTAGCGCAAGAGTCCGTCACTCCATACATTGCAATGCTGAATGGAGAGCCGATTGGGTATGCCCAGTCGTACGTTGCTCTTGGAAGCGGGGACGGATGGTGGGAAGAAGAAACCGATCCAGGAGTACGCGGAATAGACCAGTTACTGGCGAATGCATCACAACTGGGCAAAGGCTTGGGAACCAAGCTGGTTCGAGCTCTGGTTGAGTTGCTGTTCAATGATCCCGAGGTCACCAAGATCCAAACGGACCCGTCGCCGAGCAACTTGCGAGCGATCCGATGCTACGAGAAAGCGGGGTTTGAGAGGCAAGGTACCGTAACCACCCCAGATGGTCCAGCCGTGTACATGGTTCAAACACGCCAGGCATTCGAGCGAACATGCAGTGATGCCTAA

>EF210035 Stenotrophomonas maltophilia class I integron aminoglycoside 6'-N-acetyltransferase Ib cr (aac(6')-Ib-cr) gene, complete cds.

---------------------------------------ATGACTGAGCATGACCTTGCGATGCTCTATGAGTGGCTAAATCGATCTCATATCGTCGAGTGGTGGGGCGGAGAAGAAGCACGCCCGACACTTGCTGACGTACAGGAACAGTACTTGCCAAGCGTTTTAGCGCAAGAGTCCGTCACTCCATACATTGCAATGCTGAATGGAGAGCCGATTGGGTATGCCCAGTCGTACGTTGCTCTTGGAAGCGGGGACGGATGGTGGGAAGAAGAAACCGATCCAGGAGTACGCGGAATAGACCAGTTACTGGCGAATGCATCACAACTGGGCAAAGGCTTGGGAACCAAGCTGGTTCGAGCTCTGGTTGAGTTGCTGTTCAATGATCCCGAGGTCACCAAGATCCAAACGGACCCGTCGCCGAGCAACTTGCGAGCGATCCGATGCTACGAGAAAGCGGGGTTTGAGAGGCAAGGTACCGTAACCACCCCAGATGGTCCAGCCGTGTACATGGTTCAAACACGCCAGGCATTCGAGCGAACACGCATGGATGCCTAA

>EF375621 Klebsiella pneumoniae aminoglycoside acetyltransferase AAC(6')-Ib-Suzhou (aac(6')-Ib-Suzhou) gene, complete cds.

---------------------------------------ATGACTGAGCATGACCTTGCGATGCTCTATGAGTGGCTAAATCGATCTCATATCGTCGAGTGGTGGGGCGGAGAAGAAGCACGCCCGACACTTGCTGACGTACAGGAACAGTACTTGCCAAGCGTTTTAGCGCAAGAGTCCGTCACTCCATACATTGCAATGCTGAATGGAGAGCCGATTGGGTATGCCCAGTCGTACGTTGCTCTTGGAAGCGGGGACGGATGGTGGGAAGAAGAAACCGATCCAGGAGTACGCGGAATAGACCAGTTACTGGCGAATGCATCACAACTGGGCAAAGGCTTGGGAACCAAGCTGGTTCGAGCTCTGGTTGAGTTGCTGTTCAATGATCCCGAGGTCACCAAGATCCAAACGGACCCGTCGCCGAGCAACTTGCGAGCGATCCGATGCTACGAGAAAGCGGGGTTTGAGAGGCAAGGTACCGTAACCACCCCAGATGGTCCAGCCGTGTACATGGTTCAAACACGCCAGGCATTCGAGCGAACACGCATGGATGCCTAA

>EU085533 Enterobacter cloacae strain HZB7687 aminoglycoside acetyltransferase AAC(6')-Ib-Suzhou (aac(6')-Ib-Suzhou) gene, complete cds.

---------------------------------------ATGACTGAGCATGACCTTGCGATGCTCTATGAGTGGCTAAATCGATCTCATATCGTCGAGTGGTGGGGCGGAGAAGAAGCACGCCCGACACTTGCTGACGTACAGGAACAGTACTTGCCAAGCGTTTTAGCGCAAGAGTCCGTCACTCCATACATTGCAATGCTGAATGGAGAGCCGATTGGGTATGCCCAGTCGTACGTTGCTCTTGGAAGCGGGGACGGATGGTGGGAAGAAGAAACCGATCCAGGAGTACGCGGAATAGACCAGTTACTGGCGAATGCATCACAACTGGGCAAAGGCTTGGGAACCAAGCTGGTTCGAGCTCTGGTTGAGTTGCTGTTCAATGATCCCGAGGTCACCAAGATCCAAACGGACCCGTCGCCGAGCAACTTGCGAGCGATCCGATGCTACGAGAAAGCGGGGTTTGAGAGGCAAGGTACCGTAACCACCCCAGATGGTCCAGCCGTGTACATGGTTCAAACACGCCAGGCATTCGAGCGAACACGCATGGATGCCTAA

>AJ278514 Achromobacter xylosoxidans subsp. denitrificans plasmid pAX22, DNA for integron In70.

GTGACCAACAGC---AACGATTCCGTCACACTGCGCCTCATGACTGAGCATGACCTTGCGATGCTCTATGAGTGGCTAAATCGATCTCATATCGTCGAGTGGTGGGGCGGAGAAGAAGCACGCCCGACACTTGCTGACGTACAGGAACAGTACTTGCCAAGCGTTTTAGCGCAAGAGTCCGTCACTCCATACATTGCAATGCTGAATGGAGAGCCGATTGGGTATGCCCAGTCGTACGTTGCTCTTGGAAGCGGGGACGGATGGTGGGAAGAAGAAACCGATCCAGGAGTACGCGGAATAGACCAGTCACTGGCGAATGCATCACAACTGGGCAAAGGCTTGGGAACCAAGCTGGTTCGAGCACTGGTTGAGTTGCTGTTCAATGATCCCGAGGTCACCAAGATCCAAACGGACCCGTCGCCGAGCAACTTGCGAGCGATCCGATGCTACGAGAAAGCGGGGTTTGAGAGGCAGGGTACCGTAACCACCCCAGATGGTCCAGCCGTGTACATGGTTCAAACACGCCAGGCATTCGAGCGAACACGCAGTGATGCCTAA

>AJ620678 Pseudomonas aeruginosa intI1 gene (partial), blaGIM-1 gene, aac(6')-1b gene, aadA1 gene (partial), is1394 gene, aadA1 gene (partial), blaOXA-2 gene, qacEdelta1 gene and sul1 gene (partial).

GTGACCAACAGC---AACGATTC-GTCACACTGCGCCTCATGACTGAGCATGACCTTGCGATGCTCTATGAGTGGCTAAATCGATCTCATATCGTCGAGTGGTGGGGCGGAGAAGAAGCACGCCCGACACTTGCTGACGTACAGGAACAGTACTTGCCAAGCGTTTTAGCGCAAGAGTCCGTCACTCCATACATTGCAATGCTGAATGGAGAGCCGATTGGGTATGCCCAGTCGTACGTTGCTCTTGGAAGCGGGGACGGATGGTGGGAAGAAGAAACCGATCCAGGAGTACGCGGAATAGACCAGTCACTGGCGAATGCATCACAACTGGGCAAAGGCTTGGGAACCAAGCTGGTTCGAGCTCTGGTTGAGTTGCTGTTCAATGATCCCGAGGTCACCAAGATCCAAACGGACCCGTCGCCGAGCAACTTGCGAGCGATCCGATGCTACGAGAAAGCGGGGTTTGAGAGGCAAGGTACCGTAACCACCCCAGATGGTCCAGCCGTGTACATGGTTCAAACACGCCAGGCATTCGAGCGAACACGCAGTGATGCCTAA

>AY686225 Achromobacter xylosoxidans class I integron DNA integrase (intI1) gene, partial cds, aminoglycoside 6'-acetyltransferase type I (aacA6'-Ib), aminoglycoside 6'-acetyltransferase type I (aacA6'-Ib), metallo-beta-lactamase VIM-2 (blaVIM-2), and aminoglycoside 6'-acetyltransferase type I (aacA6'-Ib) genes, complete cds, and quaternary ammonium compound-resistance protein (qacEdelta) gene, partial cds.

GTGACCAACAGC---ACCGATTCCGTCACACTGCGCCTCATGACTGAGCATGACCTTGCGATGCTCTATGAGTGGCTAAATCGATCTCATATCGTCGAGTGGTGGGGCGGAGAAGAAGCACGCCCGACACTTGCTGACGTACAGGAACAGTACTTGCCAAGCGTTTTAGCGCAAGAGTCCGTCACACCATACATTGCAATGCTGAATGGAGAGCCGATTGGGTATGCCCAGTCGTACGTTGCTCTTGGAAGCGGGGACGGATGGTGGGAAGAAGAAACCGATCCAGGAGTACGCGGAATAGACCAGTCACTGGCGAATGCATCACAACTGGGCAAAGGCTTGGGAACCAAGCTGGTTCGAGCTCTGGTTGAGTTGCTGTTCAATGATCCCGAGGTCACCAAGATCCAAACGGACCCGTCGCCGAGCAACTTGCGAGCGATCCGATGCTACGAGAAAGCGGGGTTTGAGAGGCAAGGTACCGTAACCACCCCAGATGGTCCAGCCGTGTACATGGTTCAAACACGCCAGGCATTCGAGCGAACACGCAGTGATGCCTAA

>AY686225 Achromobacter xylosoxidans class I integron DNA integrase (intI1) gene, partial cds, aminoglycoside 6'-acetyltransferase type I (aacA6'-Ib), aminoglycoside 6'-acetyltransferase type I (aacA6'-Ib), metallo-beta-lactamase VIM-2 (blaVIM-2), and aminoglycoside 6'-acetyltransferase type I (aacA6'-Ib) genes, complete cds, and quaternary ammonium compound-resistance protein (qacEdelta) gene, partial cds.

GTGACCAACAGC---ACCGATTCCGTCACACTGCGCCTCATGACTGAGCATGACCTTGCGATGCTCTATGAGTGGCTAAATCGATCTCATATCGTCGAGTGGTGGGGCGGAGAAGAAGCACGCCCGACACTTGCTGACGTACAGGAACAGTACTTGCCAAGCGTTTTAGCGCAAGAGTCCGTCACACCATACATTGCAATGCTGAATGGAGAGCCGATTGGGTATGCCCAGTCGTACGTTGCTCTTGGAAGCGGGGACGGATGGTGGGAAGAAGAAACCGATCCAGGAGTACGCGGAATAGACCAGTCACTGGCGAATGCATCACAACTGGGCAAAGGCTTGGGAACCAAGCTGGTTCGAGCTCTGGTTGAGTTGCTGTTCAATGATCCCGAGGTCACCAAGATCCAAACGGACCCGTCGCCGAGCAACTTGCGAGCGATCCGATGCTACGAGAAAGCGGGGTTTGAGAGGCAAGGTACCGTAACCACCCCAGATGGTCCAGCCGTGTACATGGTTCAAACACGCCAGGCATTCGAGCGAACACGCAGTGATGCCTAA

>AY686225 Achromobacter xylosoxidans class I integron DNA integrase (intI1) gene, partial cds, aminoglycoside 6'-acetyltransferase type I (aacA6'-Ib), aminoglycoside 6'-acetyltransferase type I (aacA6'-Ib), metallo-beta-lactamase VIM-2 (blaVIM-2), and aminoglycoside 6'-acetyltransferase type I (aacA6'-Ib) genes, complete cds, and quaternary ammonium compound-resistance protein (qacEdelta) gene, partial cds.

GTGACCAACAGC---ACCGATTCCGTCACACTGCGCCTCATGACTGAGCATGACCTTGCGATGCTCTATGAGTGGCTAAATCGATCTCATATCGTCGAGTGGTGGGGCGGAGAAGAAGCACGCCCGACACTTGCTGACGTACAGGAACAGTACTTGCCAAGCGTTTTAGCGCAAGAGTCCGTCACACCATACATTGCAATGCTGAATGGAGAGCCGATTGGGTATGCCCAGTCGTACGTTGCTCTTGGAAGCGGGGACGGATGGTGGGAAGAAGAAACCGATCCAGGAGTACGCGGAATAGACCAGTCACTGGCGAATGCATCACAACTGGGCAAAGGCTTGGGAACCAAGCTGGTTCGAGCTCTGGTTGAGTTGCTGTTCAATGATCCCGAGGTCACCAAGATCCAAACGGACCCGTCGCCGAGCAACTTGCGAGCGATCCGATGCTACGAGAAAGCGGGGTTTGAGAGGCAAGGTACCGTAACCACCCCAGATGGTCCAGCCGTGTACATGGTTCAAACACGCCAGGCATTCGAGCGAACACGCAGTGATGCCTAA

>AY970968 Escherichia coli plasmid pMVH202 class 1 integron In113 VIM-1 metallo-beta-lactamase (blaVIM-1), aminoglycoside acetyltransferase (aacA4), dihydrofolate reductase (dfrII), aminoglycoside-3'-adenylyltransferase (aadA1), and chloramphenicol acetyltransferase (catB2) genes, complete cds.

-TGACCAACAGC--CAACGATTCCGTCACACTGCGCCTCATGACTGAGCATGACCTTGCGATGCTCTATGAGTGGCTAAATCGATCTCATATCGTCGAGTGGTGGGGCGGAGAAGAAGCACGCCCGACACTTGCTGACGTACAGGAACAGTACTTGCCAAGCGTTTTAGCGCAAGAGTCCGTCACTCCATACATTGCAATGCTGAATGGAGAGCCGATTGGGTATGCCCAGTCGTACGTTGCTCTTGGAAGCGGGGACGGATGGTGGGAAGAAGAAACCGATCCAGGAGTACGCGGAATAGACCAGTCACTGGCGAATGCATCACAACTGGGCAAAGGCTTGGGAACCAAGCTGGTTCGAGCACTGGTTGAGTTGCTGTTCAATGATCCCGAGGTCACCAAGATCCAAACGGACCCGTCGCCGAGCAACTTGCGAGCGATCCGATGCTACGAGAAAGCGGGGTTTGAGAGGCAAGGTACCGTAACCACCCCAGATGGTCCAGCCGTGTACATGGTTCAAACACGCCAGGCATTCGAGCGAACACGCAGTGATGCCTAA

>AY987853 Klebsiella pneumoniae plasmid pMVH202 class 1 integron In113 VIM-1 metallo-beta-lactamase (blaVIM-1), aminoglycoside acetyltransferase (aacA4), dihydrofolate reductase (dfrII), aminoglycoside-3'-adenylyltransferase (aadA1), and chloramphenicol acetyltransferase (catB2) genes, complete cds.

-TGACCAACAGC--CAACGATTCCGTCACACTGCGCCTCATGACTGAGCATGACCTTGCGATGCTCTATGAGTGGCTAAATCGATCTCATATCGTCGAGTGGTGGGGCGGAGAAGAAGCACGCCCGACACTTGCTGACGTACAGGAACAGTACTTGCCAAGCGTTTTAGCGCAAGAGTCCGTCACTCCATACATTGCAATGCTGAATGGAGAGCCGATTGGGTATGCCCAGTCGTACGTTGCTCTTGGAAGCGGGGACGGATGGTGGGAAGAAGAAACCGATCCAGGAGTACGCGGAATAGACCAGTCACTGGCGAATGCATCACAACTGGGCAAAGGCTTGGGAACCAAGCTGGTTCGAGCACTGGTTGAGTTGCTGTTCAATGATCCCGAGGTCACCAAGATCCAAACGGACCCGTCGCCGAGCAACTTGCGAGCGATCCGATGCTACGAGAAAGCGGGGTTTGAGAGGCAAGGTACCGTAACCACCCCAGATGGTCCAGCCGTGTACATGGTTCAAACACGCCAGGCATTCGAGCGAACACGCAGTGATGCCTAA

>EF207717 Pseudomonas aeruginosa strain YMC 05/5/1785 class 1 integron DNA integrase (intI1) gene, partial cds, metallo-beta-lactamase VIM-2 (blaVIM-2), aminoglycoside 6'-N-acetyltransferase type I (aacA6'-Ib), and 3''-adenyl transferase (aadA2) genes, complete cds, and quaternary ammonium compound-resistance protein (qacEdelta1) gene, partial cds.

GTGACCAACAGC--CAACGATT-CGTCACACTGCGCCTCATGACTGAGCATGACCTTGCGATGCTCTATGAGTGGCTAAATCGATCTCATATCGTCGAGTGGTGGGGCGGAGAAGAAGCACGCCCGACACTTGCTGACGTACAGGAACAGTACTTGCCAAGCGTTTTAGCGCAAGAGTCCGTCACTCCATACATTGCAATGCTGAATGGAGAGCCGATTGGGTATGCCCAGTCGTACGTTGCTCTTGGAAGCGGGGACGGATGGTGGGAAGAAGAAACCGATCCAGGAGTACGCGGAATAGACCAGTCACTGGCGAATGCATCACAACTGGGCAAAGGCTTGGGAACCAAGCTGGTTCGAGCTCTGGTTGAGTTGCTGTTCAATGATCCCGAGGTCACCAAGATCCAAACGGACCCGTCGCCGAGCAACTTGCGAGCGATCCGATGCTACGAGAAAGCGGGGTTTGAGAGGCAAGGTACCGTAACCACCCCAGATGGTCCAGCCGTGTACATGGTTCAAACACGCCAGGCATTCGAGCGAACACGCAGTGATGCCTAA

>AJ487034 Salmonella enteritidis class 1 integron conatining aacA4 gene, aadA1 gene and catB2 gene.

GTGACCAACAGC---AACGATTCCGTCACACTGCGCCTCATGACTGAGCATGACCTTGCGATGCTCTATGAGTGGCTAAATCGATCTCATATCGTCGAGTGGTGGGGCGGAGAAGAAGCACGCCCGACACTTGCTGACGTACAGGAACAGTACTTGCCAAGCGTTTTAGCGCAAGAGTCCGTCACTCCATACATTGCAATGCTGAATGGAGAGCCGATTGGGTATGCCCAGTCGTACGTTGCTCTTGGAAACGGGGACGGATGGTGGGAAGAAGAAACCGATCCAGGAGTACGCGGAATAGACCAGTCACTGGCGAATGCATCACAACTGGGCAAAGGCTTGGGAACCAAGCTGGTTCGAGCTCTGGTTGAGTTGCTGTTCAATGATCCCGAGGTCACCAAGATCCAAACGGACCCGTCGCCGAGCAACTTGCGAGCGATCCGATGCTACGAGAAAGCGGGGTTTGAGAGGCAAGGTACCGTAACCACCCCAGATGGTCCAGCCGTGTACATGGTTCAAACACGCCAGGCATTCGAGCGAACACGCAGTGATGCCTAA

>AJ439689 Pseudomonas putida partial pVA304 plasmid containing class 1 integron In110.

GTGACCAACAGC---AACGATTCCGTCACACTGCGCCTCATGACTGAGCATGACCTTGCGATGCTCTATGAGTGGCTAAATCGATCTCATATCGTCGAGTGGTGGGGCGGAGAAGAAGCACGCCCGACACTTGCTGACGTACAGGAACAGTACTTGCCAAGCGTTTTAGCGCAAGAGTCCGTCACTCCATACATTGCAATGCTGAATGGAGAGCCGATTGGGTATGCCCAGTCGTACGTTGCTCTTGGAAGCGGGGACGGATGGTGGGAAGAAGAAACCGATCCAGGAGTACGCGGAATAGACCAGTCACTGGCGAATGCATCACAACTGGGCAAAGGCTTGGGAACCAAGCTGGTTCGAGCACTGGTTGAGTTGCTGTTCAATGATCCCGAGGTCACCAAGATCCAAACGGACCCGTCGCCGAGCAACTTGCGAGCGATCCGATGCTACGAGAAAGCGGGGTTTGAGAGGCAAGGTACCGTAACCCACCCCGATGGTCCAGCCGTGTACATGGTTCAAACACGCCAGGCATTCGAGCGAACACGCAGTGATGCCTAA

>AY574992 Pseudomonas aeruginosa isolate IRPA-2 class I integron, partial sequence.

GTGACCAACAGC---AACGATTCCGTCACACTGCGCCTCATGACTGAGCATGACCTTGCGATGCTCTATGAGTGGCTAAATCGATCTCATATCGTCGAGTGGTGGGGCGGAGAAGAAGCACGCCCGACACTTGCTGACGTACAGGAACAGTACTTGCCAAGCGTTTTAGCGCAAGAGTCCGTCACTCCATACATTGCAATGCTGAATGGAGAGCCGATTGGGTATGCCCAGTCGTACGTTGCTCTTGGAGGCGGGGACGGATGGTGGGAAGAAGAAACCGATCCAGGAGTACGCGGAATAGACCAGTCACTGGCGAATGCATCACAACTGGGCAAAGGCTTGGGAACCAAGCTGGTTCGAGCACTGGTTGAGTTGCTGTTCAATGATCCCGAGGTCACCAAGATCCAAACGGACCCGTCGCCGAGCAACTTGCGAGCGATCCGATGCTACGAGAAAGCGGGGTTTGAGAGGCAAGGTACCGTAACCACCCCAGATGGTCCAGCCGTGTACATGGTTCAAACACGCCAGGCATTCGAGCGAACACGCAGTGATGCCTAA

>EF577407 Pseudomonas aeruginosa strain PA-SL2 class I integron IntI1 (intI1), class B carbapenemase VIM-13 (blaVIM-13), and AacA4 variant (aac) genes, complete cds.

GTGACCAACAGC---ACCGATTCCGTCACACTGCGCCTCATGACTGAGCATGACCTTGCGATGCTCTATGAGTGGCTAAATCGATCTCATATCGTCGAGTGGTGGGGCGGAGAAGAAGCACGCCCGACACTTGCTGACGTACAGGAACAGTACTTGCCAAGCGTTTTAGCGCAAGAGTCCGTCACTCCATACATTGCAATGCTGAATGGAGAGCCGATTGGGTATGCCCAGTCGTACGTTGCTCTTGGAAGCGGGGACGGATGGTGGGAAGAAGAAACCGATCCAGGAGTACGCGGAATAGACCAGTCACTGGCGAATGCATCACAACTGGGCAAAGGCTTGGGAACCAAGCTGGTTCGAACTCTGGTTGAGTTGCTGTTCAATGATCCCGAGGTCACCAAGATCCAAACGGACCCGTCGCCGAGTAACTTGCGAGCGATCCGATGCTACGAGAAAGCGGGGTTTGAGAGGCAAGGTACCGTAACCACCCCAGATGGTCCAGCCGTGTACATGGTTCAAACACGCCAGGCATTCGAGCGAACACGCAGTGATGCCTAA

>EF577409 Pseudomonas putida strain PP-SD1 class I integron IntI1 (intI1), AacA4 variant (aac), and class B carbapenemase VIM-2 (blaVIM-2) genes, complete cds.

GTGACCAACAGC---ACCGATTCCGTCACACTGCGCCTCATGACTGAGCATGACCTTGCGATGCTCTATGAGTGGCTAAATCGATCTCATATCGTCGAGTGGTGGGGCGGAGAAGAAGCACGCCCGACACTTGCTGACGTACAGGAACAGTACTTGCCAAGCGTTTTAGCGCAAGAGTCCGTCACTCCATACATTGCAATGCTGAATGGAGAGCCGATTGGGTATGCCCAGTCGTACGTTGCTCTTGGAAGCGGGGACGGATGGTGGGAAGAAGAAACCGATCCAGGAGTACGCGGAATAGACCAGTCACTGGCGAATGCATCACAACTGGGCAAAGGCTTGGGAACCAAGCTGGTTCGAACTCTGGTTGAGTTGCTGTTCAATGATCCCGAGGTCACCAAGATCCAAACGGACCCGTCGCCGAGTAACTTGCGAGCGATCCGATGCTACGAGAAAGCGGGGTTTGAGAGGCAAGGTACCGTAACCACCCCAGATGGTCCAGCCGTGTACATGGTTCAAACACGCCAGGCATTCGAGCGAACACGCAGTGATGCCTAA

>AF418284 Pseudomonas aeruginosa isolate YMC 95012105 class I integron In107, partial sequence.

GTGACCAACAGC---AACGATTCCGTCACACTGCGCCTCATGACTGAGCATGACCTTGCGATGCTCTATGAGTGGCTAAATCGATCTCATATCGTCGAGTGGTGGGGCGGAGAAGAAGCACGCCCGACACTTGCTGACGTACAGGAACAGTACTTGCCAAGCGTTTTAGCGCAAGAGTCCGTCACTCCATACATTGCAATGCTGAATGGAGAGCCGATTGGGTATGCCCAGTCGTACGTTGCTCTTGGAAGCGGGGACGGATGGTGGGAAGAAGAAACCGATCCAGGAGTACGCGGAATAGACCAGTCACTGGCGAATGCATCACAACTGGGCAAAAGCTTGGGAACCAAGCTGGTTCGAGCTCTGGTTGAGTTGCTGTTCAATGATCCCGAGGTCACCAAGATCCAAACGGACCCGTCGCCGAGCAACTTGCGAGCGATCCGATGCTACGAGAAAGCGGGGTTTGAGAGGCAAGGTACCGTAACCACCCCAGATGGTCCAGCCGTGTACATGGTTCAAACACGCCAGGCATTCGAGCGAACACGCAGTGATGCCTAA

>AY260546 Escherichia coli plasmid pAK33 insertion sequence IS26 transposase (tnpA) gene, complete cds; AphA1-IAB (aphA7) gene, complete cds; insertion sequence IS26 transposase (tnpA) gene, complete cds; transposon class 1 integron In111, complete sequence; and insertion sequence IS6100 transposase (tnpA) gene, complete cds.

GTGACCAACAGC---AACGATTCCGTCACACTGCGCCTCATGACTGAGCATGACCTTGCGATGCTCTATGAGTGGCTAAATCGATCTCATATCGTCGAGTGGTGGGGCGGAGAAGAAGCACGCCCGACACTTGCTGACGTACAGGAACAGTACTTGCCAAGCGTTTTAGCGCAAGAGTCCGTCACTCCATACATTGCAATGCTGAATGGAGAGCCGATTGGGTATGCCCAGTCGTACGTTGCTCTTGGAAGCGGGGACGGATGGTGGGAAGAAGAAACCGATCCAGGAGTACGCGGAATAGACCAGTCACTGGCGAATGCATCACAACTGGGCAAAGGCTTGGGAACCAAGCTGGTTCGAGCTCTGGTTGAGTTGCTGTTCAATGATCCCGAGGTCACCAAGATCCAAACGGACCCGTCGCCGAGCAACTTGCGAGCGATCCGATGCATCGAGAAAGCGGGGTTTGAGAGGCAAGGTACCGTAACCACCCCAGATGGTCCAGCCGTGTACATGGTTCAAACACGCCAGGCATTCGAGCGAACACGCAGTGATGCCTAA

>AF369871 Acinetobacter genomosp. 3 class I integron DNA integrase IntI1 (intI1) gene, partial cds; metallo-beta-lactamase VIM-2 (blaVIM-2), aminoglycoside 6'-acetyltransferase type I (aacA6'-Ib), and aminoglycoside 3'-adenylyltransferase (aadA1) genes, complete cds; quaternary ammonium compound-resistance protein (qacEdelta1) gene, partial cds; and unknown genes.

GTGACCAACAGC---AACGATTCCGTCACACTGCGCCTCATGACTGAGCATGACCTTGCGATGCTCTATGAGTGGCTAAATCGATCTCATATCGTCGAGTGGTGGGGCGGAGAAGAAGCACGCCCGACACTTGCTGACGTACAGGAACAGTACTTGCCAAGCGTTTTAGCGCAAGAGTCCGTCACTCCATACATTGCAATGCTGAATGGAGAGCCGATTGGGTATGCCCAGTCGTACGTTGCTCTTGGAAGCGGGGACGGATGGTGGGAAGAAGAAACCGATCCAGGAGTACGCGGAATAGACCAGTCACTGGCGAATGCATCACAACTGGGCAAAGGCTTGGGAACCAAGCTGGTTCGAGCTCTGGTTGAGTTGCTGTTCAATGATCCCGAGGTCACCAAGATCCAAACGGACCCGTCGCCAAGCAACTTGCGAGCGATCCGATGCTACGAGAAAGCGGGGTTTGAGAGGCAAGGTACCGTAACCACCCCAGATGGTCCAGCCGTGTACATGGTTCAAACACGCCAGGCATTCGAGCGAACACGCAGTGTTGCCTAA

>AY162283 Citrobacter freundii transposon class I integron In38 IntI1 integron integrase (intI1) gene, partial cds, aminoglycoside (6') N-acetyltransferase AAC(6')-II (aacA4), beta-lactamase OXA-4 (bla(OXA-4)), quaternary ammonium compound resistance protein (qacEdelta1), dihydropteroate synthase (sul1), Orf513 (orf513), dihydrofolate reductase type IIIb (dfrA3b), and Orf105 (orf105) genes, complete cds, qacEdelta1 pseudogene, complete sequence, and dihydropteroate synthase (sul1) gene, partial cds.

GTGACCAACAGC---AACGATTCCGTCACACTGCGCCTCATGACTGAGCATGACCTTGCGATGCTCTATGAGTGGCTAAATCGATCTCATATCGTCGAGTGGTGGGGCGGAGAAGAAGCACGCCCGACACTTGCTGACGTACAGGAACAGTACTTGCCAAGCGTTTTAGCGCAAGAGTCCGTCACTCCATACATTGCAATGCTGAATGGAGAGCCGATTGGGTATGCCCAGTCGTACGTTGCTCTTGGAAGCGGGGACGGATGGTGGGAAGAAGAAACCGATCCAGGAGTACGCGGAATAGACCAGTCACTGGCGAATGCATCACAACTGGGCAAAGGCTTGGGAACCAAGCTGGTTCGAGCTCTGGTTGAGTTGCTGTTCAATGATCCCGAGGTCACCAAGATCCAAACGGACCCGTCGCCAAGCAACTTGCGAGCGATCCGATGCTACGAGAAAGCGGGGTTTGAGAGGCAAGGTACCGTAACCACCCCAGATGGTCCAGCCGTGTACATGGTTCAAACACGCCAGGCATTCGAGCGAACACGCAGTGTTGCCTAA

>AF302086 Pseudomonas aeruginosa plasmid pVRP193 integron In72 DNA integrase IntI (intI), 6'-Ib' aminoglycoside acetyltransferase (aacA4), and metallo-beta-lactamase (blaVIM-2) genes, complete cds.

GTGACCAACAGC---AACGATTCCGTCACACTGCGCCTCATGACTGAGCATGACCTTGCGATGCTCTATGAGTGGCTAAATCGATCTCATATCGTCGAGTGGTGGGGCGGAGAAGAAGCACGCCCGACACTTGCTGACGTACAGGAACAGTACTTGCCAAGCGTTTTAGCGCAAGAGTCCGTCACTCCATACATTGCAATGCTGAATGGAGAGCCGATTGGGTATGCCCAGTCGTACGTTGCTCTTGGAAGCGGGGACGGATGGTGGGAAGAAGAAACCGATCCAGGAGTACGCGGAATAGACCAGTCACTGGCGAATGCATCACAACTGGGCAAAGGCTTGGGAACCAAGCTGGTTCGAGCTCTGGTTGAGTTGCTGTTCAATGATCCCGAGGTCACCAAGATCCAAACGGACCCGTCGCCGAGCAACTTGCGAGCGATCCGATGCTACGAGAAAGCGGGGTTTGAGAGGCAAGGTACCGTAACCACCCCAGATGGTCCAGCCGTGTACATGGTTCAAACACGCCAGGCATTCGAGCGAACACGCAGGTTTGCCTAA

>AY139594 Uncultured bacterium plasmid-encoded class I integron gene cassette array aminoglycoside-2''-adenylyltransferase (aadB) and aminoglycoside-6'N-acetyltransferase (aacA4) genes, complete cds.

GTGACCAACAGC---AACGATTCCGTCACACTGCGCCTCATGACTGAGCATGACCTTGCGATGCTCTATGAGCGGCTAAATCGATCTCATATCGTCGAGTGGTGGGGCGGAGAAGAAGCACGCCCGACACTTGCTGACGTACAGGAACAGTACTTGCCAAGCGTTTTAGCGCAAGAGTCCGTCACTCCATACATTGCAATGCTGAATGGAGAGCCGATTGGGTATGCCCAGTCGTACGTTGCTCTTGGAAGCGGGGACGGATGGTGGGAAGAAGAAACCGATCCAGGAGTACGCGGAATAGACCAGTCACTGGCGAATGCATCACAACTGGGCAAAGGCTTGGGAACCAAGCTGGTTCGAGCTCTGGTTGAGTTGCTGTTCAATGATCCCGAGGTCACCAAGATCCAAACGGACCCGTCGCCGAGCAACTTGCGAGCGATCCGATGCTACGAGAAAGCGGGGTTTGAGAGGCAAGGTACCGTAACCACCCCAGATGGTCCAGCCGTGTACATGGTTCAAACACGCCAGGCATTCGAGCGAACACGCAGTGATGCCTAA

>AJ971343 Klebsiella pneumoniae partial pRBDHA plasmid.

GTGACCAACAGC---AACGATTCCGTCACACTGCGCCTCATGACTGAGCATGACCTTGCGATGCTCTATGAGTGGCTAAATCGATCTCATATCGTCGAGTGGTGGGGCGGAGAAGAAGCACGCCCGACACTTGCTGACGTACAGGAACAGTACTTGCCAAGCGTTTTAGCGCAAGAGTCCGTCACTCCATACATTGCAATGCTGAATGGAGAGCCGATTGGGTATGCCCAGTCGTACGTTGCTCTTGGAAGCGGGGACGGAAGGTGGGAAGAAGAAACCGATCCAGGAGTACGCGGAATAGACCAGTTACTGGCGAATGCATCACAACTGGGCAAAGGCTTGGGAACCAAGCTGGTTCGAGCTCTGGTTGAGTTGCTGTTCAATGATCCCGAGGTCACCAAGATCCAAACGGACCCGTCGCCGAGCAACTTGCGAGCGATCCGATGCTACGAGAAAGCGGGGTTTGAGAGGCAAGGTACCGTAACCACCCCATATGGTCCAGCCGTGTACATGGTTCAAACACGCCAGGCATTCGAGCGAACACGCAGTGATGCCTAA

>EF375620 Enterobacter cloacae aminoglycoside acetyltransferase AAC(6')-Ib-cr (aac(6')-Ib-cr) gene, complete cds.

---------------------------------------ATGACTGAGCATGACCTTGCGATGCTCTATGAGTGGCTAAATCGATCTCATATCGTCGAGTGGTGGGGCGGAGAAGAAGCACGCCCGACACTTGCTGACGTACAGGAACAGTACTTGCCAAGCGTTTTAGCGCAAGAGTCCGTCACTCCATACATTGCAATGCTGAATGGAGAGCCGATTGGGTATGCCCAGTCGTACGTTGCTCTTGGAAGCGGGGACGGACGGTGGGAAGAAGAAACCGATCCAGGAGTACGCGGAATAGACCAGTTACTGGCGAATGCATCACAACTGGGCAAAGGCTTGGGAACCAAGCTGGTTCGAGCTCTGGTTGAGTTGCTGTTCAATGATCCCGAGGTCACCAAGATCCAAACGGACCCGTCGCCGAGCAACTTGCGAGCGATCCGATGCTACGAGAAAGCGGGGTTTGAGAGGCAAGGTACCGTAACCACCCCATATGGTCCAGCCGTGTACATGGTTCAAACACGCCAGGCATTCGAGCGAACACGCAGTGATGCCTAA

>EF465463 Klebsiella pneumoniae fluoroquinolone acetylating aminoglycoside acetyltransferase gene, partial cds.

---------------------------------------ATGACTGAGCATGACCTTGCGATGCTCTATGAGTGGCTAAATCGATCTCATATCGTCGAGTGGTGGGGCGGAGAAGAAGCACGCCCGACACTTGCTGACGTACAGGAACAGTACTTGCCAAGCGTTTTAGCGCAAGAGTCCGTCACTCCATACATTGCAATGCTGAATGGAGAGCCGATTGGGTATGCCCAGTCGTACGTTGCTCTTGGAAGCGGGGACGGACGGTGGGAAGAAGAAACCGATCCAGGAGTACGCGGAATAGACCAGTTACTGGCGAATGCATCACAACTGGGCAAAGGCTTGGGAACCAAGCTGGTTCGAGCTCTGGTTGAGTTGCTGTTCAATGATCCCGAGGTCACCAAGATCCAAACGGACCCGTCGCCGAGCAACTTGCGAGCGATCCGATGCTACGAGAAAGCGGGGTTTGAGAGGCAAGGTACCGTAACCACCCCATATGGTCCAGCCGTGTACATGGTTCAAACACGCCAGGCATTCGAGCGAACACGCAGTGATGCCTAA

>EU077612 Escherichia coli plasmid pGD008 AAC(6')-Ib-cr (aac(6')-Ib-cr) gene, partial cds.

---------------------------------------ATGACTGAGCATGACCTTGCGATGCTCTATGAGTGGCTAAATCGATCTCATATCGTCGAGTGGTGGGGCGGAGAAGAAGCACGCCCGACACTTGCTGACGTACAGGAACAGTACTTGCCAAGCGTTTTAGCGCAAGAGTCCGTCACTCCATACATTGCAATGCTGAATGGAGAGCCGATTGGGTATGCCCAGTCGTACGTTGCTCTTGGAAGCGGGGACGGAAGGTGGGAAGAAGAAACCGATCCAGGAGTACGCGGAATAGACCAGTTACTGGCGAATGCATCACAACTGGGCAAAGGCTTGGGAACCAAGCTGGTTCGAGCTCTGGTTGAGTTGCTGTTCAATGATCCCGAGGTCACCAAGATCCAAACGGACCCGTCGCCGAGCAACTTGCGAGCGATCCGATGCTACGAGAAAGCGGGGTTTGAGAGGCAAGGTACCGTAACCACCCCATATGGTCCAGCCGTGTACATGGTTCAAACACGCCAGGCATTCGAGCGAACACGCAGTGATGCCTAA

>AJ295229 Pseudomonas aeruginosa intI1 gene (partial), blaVIM-2 gene, aac(6')-Ib' gene, aadA1 gene, oxa2 gene and ORFD.

GTGACCAACAGC---AACGATTCCGTCACACTGCGCCTCATGACTGAGCATGACCTTGCGATGCTCTATGAGTGGCTAAATCGATCTCATATCGTCGAGTGGTGGGGCGGAGAAGAAGCACGCCCGACACTTGCTGACGTACAGGAACAGTACTTGCCAAGCGTTTTAGCGCAAGAGTTCGTCACTCCATACATTGCAATGCTGAATGGAGAGCCGATTGGGTATGCCCAGTCGTACGTTGCTCTTGGAAGCGGGGACGGATGGTGGGAAGAAGAAACCGATCCAGGAGTACGCGGAATAGACCAGTCACTGGCGAATGCATCACAACTGGGCAAAGGCTTGGGAACCAAGCTGGTTCGAGCTCTGGTTGAGTTGCTGTTCAATGATCCCGAGGTCACCAAGATCCAAACGGACCCGTCGCCGAGCAACTTGCGAGCGATCCGATGCTACGAGAAAGCGGGGTTTGAGAGGCAAGGTACCGTAACCACCCCAGATGGTCCAGCCGTGTACATGGTTCAAACACGCCAGGCATTCGAGCGAACACGCAGGTTTGCCTAA

>AY557339 Acinetobacter baumannii strain 1-43 class I integron Intw1 aminoglycoside 6'-N-acetyltransferase (aacA4), chloramphenicol acetyltransferase (catB8), and aminoglycoside 3'-adenyltransferase (aadA1) genes, complete cds.

GTGACCAACAGC---AACGATTCCGTCACACTGCGCCTCATGACTGAGCATGACCTTGCGATGCTCTATGAGTGGCTAAATCGATCTCATATCGTCGAGTGGTGGGGCGGAGAAGAAGCACGCCCGACACTTGCTGACGTACAGGAACAGTACTTGCCAAGCGTTTTAGCGCAAGAGTCCGTCACTCCATACATTGCAATGCTGAATGGAGAGCCGATTGGGTATGCCCAGTCGTACGTTGCTCTTGGAAGCGGGGACAGATGGTGGGAAGAAGAAACCGATCCAGGAGTACGCGGAATAGACCAGTCACTGGCGAATGCATCACAACTGGGCAAAGGCTTGGGAACCAAGCTGGTTCGAGCTCTGGTTGAGTTGCTGTTCAATGATCCCGAGGTCACCAAGATCCAAACGGACCCGTCGCCGAGCAACTTGCGAGCGATCCGATGCTACGAGAAAGCGGGGTTTGAGAGGCAAGGTACCGTAACCACCCCAGATGGTCCAGCCGTGTACATGGTTCAAACACGCCAGGCATTCGAGCGAACACGCAGTGTTGCCTAA

>AJ878850 Pseudomonas aeruginosa class 1 integron, intI1 gene (partial), aacA4 gene and catB10 gene.

ATGACCACTACC---ATTAGCTTCGTCACGCTGCGCCTCATGACCGAGCACGACCTTCCGATGCTCCATGACTGGCTAAATCGGCCTCACATCGTCGAGTGGTGGGGCGGAGAAGAAGCACGCCCGACACTTGCTGACGTACAGGAACAGTACTTGCCAAGCGTTTTAGCGCAAGAGTCCGTCACTCCATACATTGCAATGCTGAATGGAGAGCCGATTGGGTATGCCCAGTCGTACGTTGCTCTTGGAAGCGGGGACGGATGGTGGGAAGAAGAAACCGATCCAGGAGTACGCGGAATAGACCAGTCACTGGCGAATGCATCACAACTGGGCAAAGGCTTGGGAACCAAGCTGGTTCGAGCTCTGGTTGAGTTGCTGTTCAATGATCCCGAGGTCACCAAGATCCAAACGGACCCGTCGCCGAGCAACTTGCGAGCGATCCGATGCTACGAGAAAGCGGGGTTTGAGAGGCAAGGTACCGTAACCACCCCAGATGGTCCAGCCGTGTACATGGTTCAAACACGCCAGGCATTCGAGCGAACACGCAGTGTTGCCTAA

>EF501990 Shigella flexneri 3b strain NK2788 ciprofloxacin modifying enzyme gene, partial cds.

GTGACCAACAGC---AACGATTCCGTCACACTGCGCCTCATGACTGAGCATGACCTTGCGATGCTCTATGAGTGGCTAAATCGATCTCATATCGTCGAGTGGTGGGGCGGAGAAGAAGCACGCCCGACACTTGCTGACGTACAGGAACAGTACTTGCCAAGCGTTTTAGCGCAAGAGTCCGTCACTCCATACATTGCAATGCTGAATGGAGAGCCGATTGGGTATGCCCAGTCGTACGTTGCTCTTGGAAGCGGGGACGGACGGTGGGAAGAAGAAACCGATCCAGGAGTACGCGGAATAGACCAGTTACTGGCGAATGCATCACAACTGGGCAAAGGCTTGGGAACCAAGCTGGTTCGAGCTCTGGTTGAGTTGCTGTTCAATGATCCCGAGGTCACCAAGATCCAAACGGACCCGTCGCCGAGCAACTTGCGAGCGATCCGATGCTACGAGAAAGCGGGGTTTGAGAGGCAAGGTACCGTAACCACCCCATATGGTCCAGCCGTGT

>EF501991 Escherichia coli strain E4 ciprofloxacin modifying enzyme gene, partial cds.

GTGACCAACAGC---AACGATTCCGTCACACTGCGCCTCATGACTGAGCATGACCTTGCGATGCTCTATGAGTGGCTAAATCGATCTCATATCGTCGAGTGGTGGGGCGGAGAAGAAGCACGCCCGACACTTGCTGACGTACAGGAACAGTACTTGCCAAGCGTTTTAGCGCAAGAGTCCGTCACTCCATACATTGCAATGCTGAATGGAGAGCCGATTGGGTATGCCCAGTCGTACGTTGCTCTTGGAAGCGGGGACGGACGGTGGGAAGAAGAAACCGATCCAGGAGTACGCGGAATAGACCAGTTACTGGCGAATGCATCACAACTGGGCAAAGGCTTGGGAACCAAGCTGGTTCGAGCTCTGGTTGAGTTGCTGTTCAATGATCCCGAGGTCACCAAGATCCAAACGGACCCGTCGCCGAGCAACTTGCGAGCGATCCGATGCTACGAGAAAGCGGGGTTTGAGAGGCAAGGTACCGTAACCACCCCATATGGTCCAGCCGTGT

>EF501992 Escherichia coli strain AV193 ciprofloxacin modifying enzyme gene, partial cds.

GTGACCAACAGC---AACGATTCCGTCACACTGCGCCTCATGACTGAGCATGACCTTGCGATGCTCTATGAGTGGCTAAATCGATCTCATATCGTCGAGTGGTGGGGCGGAGAAGAAGCACGCCCGACACTTGCTGACGTACAGGAACAGTACTTGCCAAGCGTTTTAGCGCAAGAGTCCGTCACTCCATACATTGCAATGCTGAATGGAGAGCCGATTGGGTATGCCCAGTCGTACGTTGCTCTTGGAAGCGGGGACGGACGGTGGGAAGAAGAAACCGATCCAGGAGTACGCGGAATAGACCAGTTACTGGCGAATGCATCACAACTGGGCAAAGGCTTGGGAACCAAGCTGGTTCGAGCTCTGGTTGAGTTGCTGTTCAATGATCCCGAGGTCACCAAGATCCAAACGGACCCGTCGCCGAGCAACTTGCGAGCGATCCGATGCTACGAGAAAGCGGGGTTTGAGAGGCAAGGTACCGTAACCACCCCATATGGTCCAGCCGTGT

>EF501993 Shigella boydii strain G24371 ciprofloxacin modifying enzyme gene, partial cds.

GTGACCAACAGC---AACGATTCCGTCACACTGCGCCTCATGACTGAGCATGACCTTGCGATGCTCTATGAGTGGCTAAATCGATCTCATATCGTCGAGTGGTGGGGCGGAGAAGAAGCACGCCCGACACTTGCTGACGTACAGGAACAGTACTTGCCAAGCGTTTTAGCGCAAGAGTCCGTCACTCCATACATTGCAATGCTGAATGGAGAGCCGATTGGGTATGCCCAGTCGTACGTTGCTCTTGGAAGCGGGGACGGACGGTGGGAAGAAGAAACCGATCCAGGAGTACGCGGAATAGACCAGTTACTGGCGAATGCATCACAACTGGGCAAAGGCTTGGGAACCAAGCTGGTTCGAGCTCTGGTTGAGTTGCTGTTCAATGATCCCGAGGTCACCAAGATCCAAACGGACCCGTCGCCGAGCAACTTGCGAGCGATCCGATGCTACGAGAAAGCGGGGTTTGAGAGGCAAGGTACCGTAACCACCCCATATGGTCCAGCCGTGT

>EF514363 Uncultured bacterium clone Eaac18 aminoglycoside acetyltransferase AAC(6')-Ib (aac(6')-Ib) gene, partial cds.

---------------------------------------------------------GCGATGCTCTATGAGTGGCTAAATCGATCTCATATCGTCGAGTGGTGGGGCGGAGAAGAAGCACGCCCGACACTTGCCGACGTACAGGAACAGTACTTGCCAAGCGTTTTAGCGCAAGAGTCCGTCACTCCATACATTGCAATGCTGAATGGAGAGCCGATTGGGTATGCCCAGTCGTACGTTGCTCTTGGAAGCGGGGACGGATGGTGGGAAGAAGAAACCGATCCAGGAGTACGCGGAATAGACCAGTCACTGGCGAATGCATCACAACTGGGCAAAGGCTTGGGAACCAAGCTGGTTCGAGCTCTGGTTGAGTTGCTGTTCAATGATCCCGAGGTCACCAAGATCCAAACGGACCCGTCGCCGAGCAACTTGCGAGCGATCCGATGCTACGAGAAAGCGGGGTTTGAGAGGCAAGGTACCGTAACCACCCCAGATGGTCCAGCCGTGTACATGGTTCAAACACGCCAGGCATTCGAG

>EF514365 Uncultured bacterium clone Laac21 aminoglycoside acetyltransferase AAC(6')-Ib (aac(6')-Ib) gene, partial cds.

---------------------------------------------------------GCGATGCTCTATGAGTGGCTAAATCGATCTCATATCGTCGAGTGGTGGGGCGGAGAAGAAGCACGCCCGACACTTGCTGACGTACAGGAACAGTACTTGCCAAGCGTTTTAGCGCAAGAGTCCGTCACTCCATACATTGCAATGCTGAATGGAGAGCCGATTGGGTATGCCCAGTCGTACGTTGCTCTTGGAAGCGGGGACGGATGGTGGGAAGAAGAAACCGATCCAGGAGTACGCGGAATAGACCAGTCACTGGCGAATGCATCACAACTGGGCAAAGGCTTGGGAACCAAGCTGGTTCGAGCTCTGGTTGAGTTGCTGTTCAATGATCCCGAGGTCACCAAGATCCAAACGGACCCGTCGCCGAGCAACTTGCGAGCGATCCGATGCTACGAGAAAGCGGGGTTTGAGAGGCAAGGTACCGTAACCACCCCAGATGGTCCAGCCGTGTACATGGTTCAAACACGCCAGGCATTCGAGA

>EF514367 Uncultured bacterium clone 2Aaac8 aminoglycoside acetyltransferase AAC(6')-Ib (aac(6')-Ib) gene, partial cds.

---------------------------------------------------------GCGATGCTCTATGAGTGGCTAAATCGATCTCATATCGTCGAGTGGTGGGGCGGAGAAGAAGCACGCCCGACACTTGCTGACGTACAGGAACAGTACTTGCCAAGCGTTTTAGCGCAAGAGTCCGTCACTCCATACATTGCAATGCTGAATGGAGAGCCGATTGGGTATGCCCAGTCGTACGTTGCTCTTGGAAGCGGGGACGGATGGTGGGAAGAAGAAACCGATCCAGGAGTACGCGGAATAGACCAGTCACTGGCGAATGCATCACAACTGGGCAAAGGCTTGGGAACCAAGCTGGTTCGAGCTCTGGTTGAGTTGCTGTTCAATGATCCCGAGGTCACCAAGATCCAAACGGACCCGTCGCCGAGCAACTTGCGAGCGATCCGATGCTACGAGAAAGCGGGGTTTGAGAGGCAAGGTACCGTAACCACCCCAGATGGTCCAGCCGTGTACATGGTTCAAACACGCCAGGCATTCGAGA

>EF514360 Uncultured bacterium clone Eaac1 aminoglycoside acetyltransferase AAC(6')-Ib (aac(6')-Ib) gene, partial cds.

---------------------------------------------------------GCGATGCTCTATGAGTGGCTAAATCGATCTCATATCGTCGAGTGGTGGGGCGGAGAAGAAGCACGCCCGACACTTGCTGACGTACAGGAACAGTACTTGCCAAGCGTTTTAGCGCAAGAGTCCGTCACTCCATACATTGCAATGCTGAATGGAGAGCCGATTGGGTATGCCCAGTCGTACGTTGCTCTTGGAAGCGGGGACGGATGGTGGGAAGAAGAAACCGATCCAGGAGTACGCGGAATAGACCAGTCACTGGCGAATGCATCACGACTGGGCAAAGGCTTGGGAACCAAGCTGGTTCGAGCTCTGGTTGAGTTGCTGTTCAATGATCCCGAGGTCACCAAGATCCAAACGGACCCGTCGCCGAGCAACTTGCGAGCGATCCGATGCTACGAGAAAGCGGGGTTTGAGAGGCAAGGTACCGTAACCACCCCAGATGGTCCAGCCGTGTACATGGTTCAAACACGCCAGGCATTCGAG

>EF443074 Klebsiella pneumoniae CZ2987 fluoroquinolone acetylating aminoglycoside acetyltransferase (acc(6')-Ib-Cr) gene, partial cds.

GTGACCAACAGC---AACGATTCCGTCACACTGCGCCTCATGACTGAGCATGACCTTGCGATGCTCTATGAGTGGCTAAATCGATCTCATATCGTCGAGTGGTGGGGCGGAGAAGAAGCACGCCCGACACTTGCTGACGTACAGGAACAGTACTTGCCAAGCGTTTTAGCGCAAGAGTCCGTCACTCCATACATTGCAATGCTGAATGGAGAGCCGATTGGGTATGCCCAGTCGTACGTTGCTCTTGGAAGCGGGGACGGAAGGTGGGAAGAAGAAACCGATCCAGGAGTACGCGGAATAGACCAGTTACTGGCGAATGCATCACAACTGGGCAAAGGCTTGGGAACCAAGCTGGTTCGAGCTCTGGTTGAGTTGCTGTTCAATGATCCCGAGGTCACCAAGATCCAAACGGACCCGTCGCCGAGCAACTTGCGAGCGATCCGATGCTACGAGAAAGCGGGGTTTGAGAGGCAAGGT

>EF529518 Enterobacter cloacae AAC(6')-Ib-cr gene, partial cds.

---------------------------------------------------------GCGATGCTCTATGAGTGGCTAAATCGATCTCATATCGTCGAGTGGTGGGGCGGAGAAGAAGCACGCCCGACACTTGCTGACGTACAGGAACAGTACTTGCCAAGCGTTTTAGCGCAAGAGTCCGTCACTCCATACATTGCAATGCTGAATGGAGAGCCGATTGGGTATGCCCAGTCGTACGTTGCTCTTGGAAGCGGGGACGGACGGTGGGAAGAAGAAACCGATCCAGGAGTACGCGGAATAGACCAGTTACTGGCGAATGCATCACAACTGGGCAAAGGCTTGGGAACCAAGCTGGTTCGAGCTCTGGTTGAGTTGCTGTTCAATGATCCCGAGGTCACCAAGATCCAAACGGACCCGTCGCCGAGCAACTTGCGAGCGATCCGATGCTACGAGAAAGCGGGGTTTGAGAGGCAAGGTACCGTAACCACCCCATATGGTCCAGCCGTGTACATGGTTCAAACACGCCAGGCATTCGAG

>EF514366 Uncultured bacterium clone 2Aaac1 aminoglycoside acetyltransferase AAC(6')-Ib (aac(6')-Ib) gene, partial cds.

---------------------------------------------------------GCGATGCTCTATGAGTGGCTAAATCGATCTCATATCGTCGAGTGGTGGGGCGGAGAAGAAGCACGCCCGACACTTGCTGACGTACAGGAACAGTACTTGCCAAGCGTTTTAGCGCAAGAGTCCGTCACTCCATACATTGCAATGCTGAATGGAGAGCCGATTGGGTATGCCCAGTCGTACGTTGCTCTTGGAAGCGGGGACGGATGGTGGGAAGAAGAAACCGATCCAGGAGTACGCGGAATAGGCCAGTCACTGGCGAATGCATCACAACTGGGCAAAGGCTTGGGAGCCAAGCTGGTTCGAGCTCTGGTTGAGTTGCTGTTCAATGATCCCGAGGTCACCAAGATCCAAACGGACCCGTCGCCGAGCAACTTGCGAGCGATCCGATGCTACGAGAAAGCGGGGTTTGAGAGGCAAGGTACCGTAACCACCCCAGATGGTCCAGCCGTGTACATGGTTCAAACACGCCAGGCATTCGAGA

>EF514361 Uncultured bacterium clone Eaac6 aminoglycoside acetyltransferase AAC(6')-Ib (aac(6')-Ib) gene, partial cds.

---------------------------------------------------------GCGATGCTCTATGAGTGGCTAAATCGATCTCATATCGTCGAGTGGTGGGGCGGAGAAGAAGCACGCCCGACACTTGCTGACGTACAGGAACAGTACTTGCCAAGCGTTTTAGCGCAAGAGTCCGTCACTCCATACATTGCAATGCTGAATGGAGAGCCGATTGGGTATGCCCAGTCGTACGTTGCTCTTGGAAGCGGGGACGGATGGTGGGAAGAAGAAACCGATCCAGGAGTACGCGGAATAGACCAGTCACTGGCGAATGCATCACAACTGGGCAAAGGCTTGGGAACCAAGCTGGTTCGAGCTCTGGTTGAGTTGCTGTTCAATGATCCCGAGGTCACCAAGATCCAAACGGACCCGTCGCCGAGCAACTTGCGAGCGATCCGATGCTACGAGAAAGCGGGGTTTGAGTGGCAAGGTACCGTAACCACCCCAGATGGTCCAGCCGTGTACATGGTTCAAACACGCCAGGCATTCGAG

>EF590261 Acinetobacter baumannii strain SZ9 aminoglycoside-modifying enzyme (aacA4) gene, partial cds.

-------------------------------------------------------------------------------TCGGATCTCATATCGTCGAGTGGTGGGGCGGAGAAGAAGCACGCCCGACACTTGCTGACGTACAGGAACAGTACTTGCCAAGCGTTTTAGCGCAAGAGTCCGTCACTCCATACATTGCAATGCTGAATGGAGAGCCGATTGGGTATGCCCAGTCGTACGTTGCTCTTGGAAGCGGGGACGGATGGTGGGAAGAAGAAACCGATCCAGGAGTACGCGGAATAGACCAGTCACTGGCGAATGCATCACAACTGGGCAAAGGCTTGGGAACCAAGCTGGTTCGAGCTCTGGTTGAGTTGCTGTTCAATGATCCCGAGGTCACCAAGATCCAAACGGACCCGTCGCCGAGCAACTTGCGAGCGATCCGATGCTACGAGAAAGCGGGGTTTGAGAGGCAAGGTACCGTAACCACCCCAGATGGTCCAGCCGTGTACATGGTTCAAACACGCCAGGCATTCGAGCGAACACGCAGTGATGCCTAA

>AM283490 Acinetobacter baumannii partial class 1 Integron In86, isolate 48-501A.

ATGACCACTACC---ATTAGCTTCGTCACGCTGCGCCTCATGACCGAGCACGACCTTCCGATGCTCCATGACTGGCTAAATCGGCCTCACATCGTTGAGTGGTGGGGCGGAGAAGAAACACGTCCAACACTTGCTGAAGTGCTGGAGCAATACCTACCAAGCGCCCTGGCGAAAGAGTCCGTCACTCCCTACATCGCAATGCTGGATGAAGAACCGATTGGGTACGCTCAGTCGTACATTGCACTCGGAAGCGGTGACGGATGGTGGGAAGACGAAACCGATCCAGGAGTACGCGGAATAGACCAGTCTCTGGCGAATCCATCGCAGCTGGGCAAGGGCTTGGGAACCAAGCTCGTTTGCGCGCTCGTTGAGATGCTGTTCAAAGACGCTGAGGTAACCAAGATCCAAACGGACCCGTCGCCGAACAACTTACGCGCAATCCGGTGCTACGAGAAGGCGGGTTTTGTGGCGCAAAGAACCATAAACACCCCAGATGGACCGGCCGTATACATGGTTCAAACACGTCAGGCGTTCGAGCAGGCGCGCAGTGCTGTCTAA

>AJ640197 Acinetobacter baumannii partial class 1 integron In86.

ATGACCACTACC---ATTAGCTTCGTCACGCTGCGCCTCATGACCGAGCACGACCTTCCGATGCTCCATGACTGGCTAAATCGGCCTCACATCGTTGAGTGGTGGGGCGGAGAAGAAACACGTCCAACACTTGCTGAAGTGCTGGAGCAATACCTACCAAGCGCCCTGGCGAAAGAGTCCGTCACTCCCTACATCGCAATGCTGGATGAAGAACCGATTGGGTACGCTCAGTCGTACATTGCACTCGGAAGCGGTGACGGATGGTGGGAAGACGAAACCGATCCAGGAGTACGCGGAATAGACCAGTCTCTGGCGAATCCATCGCAGCTGGGCAAGGGCTTGGGAACCAAGCTCGTTTGCGCGCTCGTTGAGATGCTGTTCAAAGACGCTGAGGTAACCAAGATCCAAACGGACCCGTCGCCGAACAACTTACGCGCAATCCGGTGCTACGAGAAGGCGGGTTTTGTGGCGCAAAGAACCATAAACACCCCAGATGGACCGGCCGTATACATGGTTCAAACACGTCAGGCGTTCGAGCAGGCGCGCAGTGCTGTCTAA

>AM283489 Pseudomonas putida partial class 1 Integron In86, isolate 48-12348A.

ATGACCACTACC---ATTAGCTTCGTCACGCTGCGCCTCATGACCGAGCACGACCTTCCGATGCTCCATGACTGGCTAAATCGGCCTCACATCGTTGAGTGGTGGGGCGGAGAAGAAACACGTCCAACACTTGCTGAAGTGCTGGAGCAATACCTACCAAGCGCCCTGGCGAAAGAGTCCGTCACTCCCTACATCGCAATGCTGGATGAAGAACCGATTGGGTACGCTCAGTCGTACATTGCACTCGGAAGCGGTGACGGATGGTGGGAAGACGAAACCGATCCAGGAGTACGCGGAATAGACCAGTCTCTGGCGAATCCATCGCAGCTGGGCAAGGGCTTGGGAACCAAGCTCGTTTGCGCGCTCGTTGAGATGCTGTTCAAAGACGCTGAGGTAACCAAGATCCAAACGGACCCGTCGCCGAACAACTTACGCGCAATCCGGTGCTACGAGAAGGCGGGTTTTGTGGCGCAAAGAACCATAAACACCCCAGATGGACCGGCCGTATACATGGTTCAAACACGTCAGGCGTTCGAGCAGGCGCGCAGTGCTGTCTAA

>EF571008 Escherichia coli strain CQ4 aminoglycoside acetyltransferase AAC(6')-Ib-cr (aac(6')-Ib-cr) gene, partial cds.

---------------------------------------------------------GCGATGCTCTATGAGTGGCTAAATCGATCTCATATCGTCGAGTGGTGGGGCGGAGAAGAAGCACGCCCGACACTTGCTGACGTACAGGAACAGTACTTGCCAAGCGTTTTAGCGCAAGAGTCCGTCACTCCATACATTGCAATGCTGAATGGAGAGCCGATTGGGTATGCCCAGTCGTACGTTGCTCTTGGAAGCGGGGACGGACGGTGGGAAGAAGAAACCGATCCAGGAGTACGCGGAATAGACCAGTTACTGGCGAATGCATCACAACTGGGCAAAGGCTTGGGAACCAAGCTGGTTCGAGCTCTGGTTGAGTTGCTGTTCAATGATCCCGAGGTCACCAAGATCCAAACGGACCCGTCGCCGAGCAACTTGCGAGCGATCCGATGCTACGAGAAAGCGGGGTTTGAGAGGCAAGGTACCGTAACCACCCCATATGGTCCAGCCGTGTACATGGTTCAA

>AY444814 Pseudomonas aeruginosa integron In78 TnpA (tnpA), Int1 (int1), AacA8 (aacA8), Oxa-2a (oxa-2), AacA7 (aacA7), AadA6 (aadA6), QacEdelta1 (qacEdelta1), and Sul1 (sul1) genes, complete cds, and unknown genes.

ATGACATCCAGC---ATTAGTTTCGTCAAGCTGCGCCTCATGACCGAGCAAGACCTTCCGATGCTCCATGAGTGGCTAAACCGGCCTCACATCGTTGAGTGGTGGGGCGGAGAAGAAGCACGTCCAACACTTGCTGAAGTGCAGGAGCAATACCTGCCAAGCGCCTTGGCGAGAGAGTCCGTCACTCCCTACATCGCAATGCTGGATGAAGAACCGATTGGGTACGCTCAGTCGTACGTTGCACTCGGAAGCGGTGGCGGATGGTGGGAAGACGAAACGGATCCAGGAGTACGCGGAATTGACCAGTCCCTGGCAAATCCATCGCAGCTGGGAAAGGGGCTAGGAACCAAGCTTGTTCGCGCGCTCGTTGAGATGCTGTTCAAAGACGCTAAGGTAACCAATATCCAAACGGACCCGTCGCCGAACAACTTGCGCGCAATCCGGTGCTACGAGAAGGCGGGGTTTGTGACGCAAAGAACCATAACCACCCCAGACGGGCCGGCTGTGTACATGGTTCAAACACGTCAGGCGTTCGAGCAGGCGCGCAGTGCTGCCTAA

>DQ357197 Pseudomonas aeruginosa MB197 class I integron VIM-4 (blaVIM-4), Aac(6')-Ib-like (aacA8), OXA-2 (blaOXA-2), and AacA7 (aacA7) genes, complete cds, and unknown gene.

ATGACATCCAGC---ATTAGTTTCGTCAAGCTGCGCCTCATGACCGAGCAAGACCTTCCGATGCTCCATGAGTGGCTAAACCGGCCTCACATCGTTGAGTGGTGGGGCGGAGAAGAAGCACGTCCAACACTTGCTGAAGTGCAGGAGCAATACCTGCCAAGCGCCTTGGCGAGAGAGTCCGTCACTCCCTACATCGCAATGCTGGATGAAGAACCGATTGGGTACGCTCAGTCGTACGTTGCACTCGGAAGCGGTGGCGGATGGTGGGAAGACGAAACGGATCCAGGAGTACGCGGAATTGACCAGTCCCTGGCAAATCCATCGCAGCTGGGAAAGGGGCTAGGAACCAAGCTTGTTCGCGCGCTCGTTGAGATGCTGTTCAAAGACGCTAAGGTAACCAATATCCAAACGGACCCGTCGCCGAACAACTTGCGCGCAATCCGGTGCTACGAGAAGGCGGGGTTTGTGACGCAAAGAACCATAACCACCCCAGACGGGCCGGCTGTGTACATGGTTCAAACACGTCAGGCGTTCGAGCAGGCGCGCAGTGCTGCCTAA

>EF067855 Escherichia coli plasmid E107 6-N-aminglycoside acetyltransferase (aac(6')-Ib) gene, partial cds.

---------------------------------------------------------------------------------------------GTCGAGTGGTGGGGCGGAGAAGAAGCACGCCCGACACTTGCTGACGTACAGGAACAGTACTTGCCAAGCGTTTTAGCGCAAGAGTCCGTCACTCCATACATTGCAATGCTGAATGGAGAGCCGATTGGGTATGCCCAGTCGTACGTTGCTCTTGGAAGCGGGGACGGAAGGTGGGAAGAAGAAACCGATCCAGGAGTACGCGGAATAGACCAGTTACTGGCGAATGCATCACAACTGGGCAAAGGCTTGGGAACCAAGCTGGTTCGAGCTCTGGTTGAGTTGCTGTTCAATGATCCCGAGGTCACCAAGATCCAAACGGACCCGTCGCCGAGCAACTTGCGAGCGATCCGATGCTACGAGAAAGCGGGGTTTGAGAGGCAAGGTACCGTAACCACCCCATATGGTCCAGCCGTGTACATGGTTCAAACACGCCAGGCATTCGA

>EF514364 Uncultured bacterium clone Laac20 aminoglycoside acetyltransferase AAC(6')-Ib (aac(6')-Ib) gene, partial cds.

---------------------------------------------------------GCGATGCTCTATGAGTGGCTAAACCGGCCTCACATCGTTGAGTGGTGGGGCGGAGAAGAAGCACGTCCAACACTTGCTGAAGTGCAGGAGCAATACCTGCCAAGCGCCTTGGCGAGAGAGTCCGTCACTCCCTACATCGCAATGCTGGATGAAGAACCGATTGGGTACGCTCAGTCGTACGTTGCACTCGGAAGCGGTGACGGATGGTGGGAAGACGAAACCGATCCAGGAGTACGCGGAATTGACCAGTCCCTGGCAAATCCATCGCAGCTGGGAAAGGGGCTAGGAACCAAGCTTGTTCGCGCGCTCGTTGAGATGCTGTTCAAAGACGCTAAGGTAACCAATATCCAAACGGACCCGTCGCCGAACAACTTGCGTGCAATCCGGTGCTACGAGAAGGCGGGGTCTGTGACGCAAAGAACCATAACCACGCCAGACGGGCCGGCTGTGTACATGGTTCAAACACGCCAGGCATTCGAGA

>EF514368 Uncultured bacterium clone 2Aaac16 aminoglycoside acetyltransferase AAC(6')-Ib (aac(6')-Ib) gene, partial cds.

---------------------------------------------------------GCGATGCTCTATGAGTGGCTAAATCGGCCTCACATCGTTGAGTGGTGGGGCGGAGAAGAAACACGTCCAACACGTGCTGAAGTGCTGGAGCAATACCTACCAAGTGCCCTGGCGAAAGAGTCCGTCACTCCCTACATCGCAATGCTGGATGAAGAACCGATTGGGTACGCTCAGTCGTACATTGCACTCGGAAGCGGTGGCGGATGGTGGGAAGACGAAACCGATCCAGGAGTACGCGGAATAGACCAGTCTCTGGCGAATCCATCGCAGCTGGGCAAGGGCTTGGGAACCAAGCTCGTTCGCGCGCTCGTTGAGATGCTGTTTAAAGACCTTGAGGTAACCAAGATCCAAACGGACCCGTCGCCGAACAACTTACGCGCAATCCGGTGCTACGAGAAGGCGGGTTTTGTGGCGCAAAGAACCATAAACACCCCAGATGGACCGGCCGTATACATGGTTCAAACACGCCAGGCATTCGAGA

>EU081298 Enterobacter aerogenes strain Dublin 1 AAC(6')-Ib-cr gene, partial cds.

------------------------------------------------------------------------------------------------------------------GAAGCACGCCCGACACTTGCTGACGTACAGGAACAGTACTTGCCAAGCGTTTTAGCGCAAGAGTCCGTCACTCCATACATTGCAATGCTGAATGGAGAGCCGATTGGGTATGCCCAGTCGTACGTTGCTCTTGGAAGCGGGGACGGAAGGTGGGAAGAAGAAACCGATCCAGGAGTACGCGGAATAGACCAGTTACTGGCGAATGCATCACAACTGGGCAAAGGCTTGGGAACCAAGCTGGTTCGAGCTCTGGTTGAGTTGCTGTTCAATGATCCCGAGGTCACCAAGATCCAAACGGACCCGTCGCCGAGCAACTTGCGAGCGATCCGATGCTACGAGAAAGCGGGGTTTGAGAGGCAAGGTACCGTAACCACCCCATATGGTCCAGCCGTGTACATGGTTCAAACACGCCAGGCATTCGAG

>AJ937775 Yersinia sp. TR 63 partial aac6'-Ib gene for aminoglycoside 6-N-acetyltransferase.

GTGACCAACAGC---AACGATTCCGTCACACTGCGCCTCATGACTGAGCATGACCTTGCGATGCTCTATGAGTGGCTAAATCGATCTCATATCGTCGAGTGGTGGGGCGGAGAAGAAGCACGCCCGACACTTGCTGACGTACAGGAACAGTACTTGCCAAGCGTTTTAGCGCAAGAGTCCGTCACTCCATACATTGCAATGCTGAATGGAGAGCCGATTGGGTATGCCCAGTCGTACGTTGCTCTTGGAAGCGGGGACGGATGGTGGGAAGAAGAAACCGATCCAGGAGTACGCGGAATAGACCAGTTACTGGCGAATGCATCACAACTGGGCAAAGGCTTGGGAACCAAGCTGGTTCGAGCTCTGGTTGAGTTGCTGTTCAATGATCCCGAGGTCACC

>AJ938159 Providencia sp. TR 58 aac(6') gene for aminoglycoside 6'-N- acetyl transferase.

---------------------------------------------------------------------------------------------------------GGCGGAGAAGAAGCACGCCCGACACTTGCTGACGTACAGGAACAGTACTTGCCAAGCGATTTAGCGCAAGAGTCCGTCACTCCATACATTGCAATGCTGAATGGAGAGCCGATTGGGTATGCCCAGTCGTACGTTGCTCTTGGAAGCGGGGACGGATGGTGGGAAGAAGAAACCGATCCAGGAGTACGCGGAATAGACCAGTTACTGGCGAATGCATCACAACTGGGCAAAGGCTTGGGAACCAAGCTGGTTCGAGCTCTGGTTGAGTTGCTGTTCAATGATCCCGAGGTCACCAAGATCCAAACGGACCCGTCGCCGAGCAACTTGCGAGCGATCCGATGCTACGAGAAAGCGGGGTTTGAGAGGCAAGGTACCGTAACCACCCCAGATGGTCCAGCCGTGTACATGGTTCAAACACGCCAGGCATTCGAGCGAACACGCAGTGATGCCTATCCCTTCCATCGAGGGGGACGTCCAAGGGCTGGCGCCCTTGGCGGCCCCTCATGTCAAACGCTAGATGCACTAAGCACATAA

>AY553609 Escherichia coli aminoglycoside 6'-N-acetyltransferase gene, partial cds.

------------------------------------------------------------------------------------------------------------------GAAGCACGCCCGACACTTGCTGACGTACAGGAACAGTACTTGCCAAGCGTTTTAGCGCAAGAGTCCGTCACTCCATACATTGCAATGCTGAATGGAGAGCCGATTGGGTATGCCCAGTCGTACGTTGCTCTTGGAAGCGGGGACGGATGGTGGGAAGAAGAAACCGATCCAGGAGTACGCGGAATAGACCAGTTACTGGCGAATGCATCACAACTGGGCAAAGGCTTGGGAACCAAGCTGGTTCGAGCTCTGGTTGAGTTGCTGTTCAATGATCCCGAGGTCACCAAGATCCAAACGGACCCGTCGCCGAGCAACTTGCGAGCGATCCGATGCTACGAGAAAGCG

>AY536063 Acinetobacter baumannii 6'-N-acetyltransferase (aacA4) gene, partial cds.

---------------------------------------------------------------------------------------------------------------GAAGAAGCACGCCCGACACTTGCTGACGTACAGGAACAGTACTTGCCAAGCGTTTTAGCGCAAGAGTCCGTCACTCCATACATTGCAATGCTGAATGGAGAGCCGATTGGGTATGCCCAGTCGTACGTTGCTCTTGGAAGCGGGGACGGATGGTGGGAAGAAGAAACCGATCCAGGAGTACGCGGAATAGACCAGTCACTGGCGAATGCATCACAACTGGGCAAAGGCTTGGGAACCAAGCTGGTTCGAGCTCTGGTTGAGTTGCTGTTCAATGATCCCGAGGTCACCAAGATCCAAACGGACCCGTCGCCGAGCAACTTGCGAGCGATCCGATGCTACGAGAAAGCG

>AY675227 Klebsiella pneumoniae strain KpnW02 6'-N-acetyltransferase (aacA4) gene, partial cds.

---------------------------------------------------------------------------------------------------------------GAAGAAGCACGCCCGACACTTGCTGACGTACAGGAACAGTACTTGCCAAGCGTTTTAGCGCAAGAGTCCGTCACTCCATACATTGCAATGCTGAATGGAGAGCCGATTGGGTATGCCCAGTCGTACGTTGCTCTTGGAAGCGGGGACGGATGGTGGGAAGAAGAAACCGATCCAGGAGTACGCGGAATAGACCAGTCACTGGCGAATGCATCACAACTGGGCAAAGGCTTGGGAACCAAGCTGGTTCGAGCTCTGGTTGAGTTGCTGTTCAATGATCCCGAGGTCACCAAGATCCAAACGGACCCGTCGCCGAGCAACTTGCGAGCGATCCGATGCTACGAGAAAGCG

>AY912486 Klebsiella pneumoniae aminoglycoside 6'-N-acetyltransferase (aacA4) gene, partial cds.

---------------------------------------------------------------------------------------------------------------GAAGAAGCACGCCCGACACTTGCTGACGTACAGGAACAGTACTTGCCAAGCGTTTTAGCGCAAGAGTCCGTCACTCCATACATTGCAATGCTGAATGGAGAGCCGATTGGGTATGCCCAGTCGTACGTTGCTCTTGGAAGCGGGGACGGATGGTGGGAAGAAGAAACCGATCCAGGAGTACGCGGAATAGACCAGTCACTGGCGAATGCATCACAACTGGGCAAAGGCTTGGGAACCAAGCTGGTTCGAGCTCTGGTTGAGTTGCTGTTCAATGATCCCGAGGTCACCAAGATCCAAACGGACCCGTCGCCGAGCAACTTGCGAGCGATCCGATGCTACGAGAAAGCG

>AY663803 Enterobacter cloacae 6'-N-acetyltransferase (aacA4) gene, partial cds.

------------------------------------------------------------------------------------------------------------------GAAGCACGCCCGACACTTGCTGACGTACAGGAACAGTACTTGCCAAGCGTTTTAGCGCAAGAGTCCGTCACTCCATACATTGCAATGCTGAATGGAGAGCCGATTGGGTATGCCCAGTCGTACGTTGCTCTTGGAAGCGGGGACGGATGGTGGGAAGAAGAAACCGATCCAGGAGTACGCGGAATAGACCAGTCACTGGCGAATGCATCACAACTGGGCAAAGGCTTGGGAACCAAGCTGGTTCGAGCTCTGGTTGAGTTGCTGTTCAATGATCCCGAGGTCACCAAGATCCAAACGGACCCGTCGCCGAGCAACTTGCGAGCGATCCGATGCTACGAGAAAGCG

>EF542813 Escherichia coli strain DU38480/05 plasmid AAC(6')-Ib-cr (aac(6')-Ib-cr) gene, partial cds.

---------------------------------------------------------------------------------------------------------------------------------------------------------------------------------------------------------------------CCGATTGGGTATGCCCAGTCGTACGTTGCTCTTGGAAGCGGGGACGGACGGTGGGAAGAAGAAACCGATCCAGGAGTACGCGGAATAGACCAGTTACTGGCGAATGCATCACAACTGGGCAAAGGCTTGGGAACCAAGCTGGTTCGAGCTCTGGTTGAGTTGCTGTTCAATGATCCCGAGGTCACCAAGATCCAAACGGACCCGTCGCCGAGCAACTTGCGAGCGATCCGATGCTACGAGAAAGCGGGGTTTGAGAGGCAAGGTACCGTAACCACCCCATATGGTCCAGCCGTGTACATGGTTCAAACACGCCAGGCATTCGAG

>EF542812 Klebsiella pneumoniae strain DU33062/05 plasmid AAC(6')-Ib-cr (aac(6')-Ib-cr) gene, partial cds.

----------------------------------------------------------------------------------------------------------------------------------------------------------------------------------------------------------------------CGATTGGGTATGCCCAGTCGTACGTTGCTCTTGGAAGCGGGGACGGACGGTGGGAAGAAGAAACCGATCCAGGAGTACGCGGAATAGACCAGTTACTGGCGAATGCATCACAACTGGGCAAAGGCTTGGGAACCAAGCTGGTTCGAGCTCTGGTTGAGTTGCTGTTCAATGATCCCGAGGTCACCAAGATCCAAACGGACCCGTCGCCGAGCAACTTGCGAGCGATCCGATGCTACGAGAAAGCGGGGTTTGAGAGGCAAGGTACCGTAACCACCCCATATGGTCCAGCCGTGTACATGGTTCAAACACGC

>D50438 Serratia marcescens DNA for integrase, metallo-bata-lactamase, aminoglycoside acetyltransferase, complete cds.

GTGACCAACAGC---ACCGATTCCGTCACACTGCGCCTCATGACTGAGCATGACCTTGCGATGCTCTATGAGTGGCTAAATCGATCTCATATCGTCGAGTGGTGGGGCGGAGAAGAAGCACGCCCGACACTTGCTGACGTACAGGAACAGTACTTGCCAAGCGTTTTAGCGCAAGAGTCCGTCACTCCATACATTGCAATGCTGAATGGAGAGCCGATTGGGTATGCCCAGTCGTACGTTGCTCTTGGAAGCGGGGACGGATGGTGGGAAGAAGAAACCGATCCAGGAGTACGCGGAATAGACCAGTCACTGGCGAATGCATCACAACTGGGCAAAGGCTTGGGAACCAAGCTGGTTCGAGCT

>PLPPSASPR Plasmid pSa DNA region containing the Sp-R gene of pSa.

-------------------------------------------------------------------------------------------------------------------------------------------------------------------------------------------------------------------------------------AGTCGTACGTTGCTCTTGGAA-CGGGGACG-ATGGTGGGAAGAAGAAACCGATCCAGGAGTACGCGGAATAGACCAGTCACTGGCGAATGCATCACAACTGGGCAAAGGCTTGGGAACCAAGCTGGTTCGAGCTCTGGTTGAGTTGCTGTTCAATGATCCCGAGGTCACCAAGATCCAAACGGACCCGTCGCCGAGCAACTTGCGAGCGATCCGATGCTACGAGAAAGCGGGGTTTGAGAGGCAAGGTACCGTAACCACCCCAGATGGTCCAGCCGTGTACATGGTTCAAACACGCCAGGCATTCGAGCGAACACGCAGTGATGCCTAA

>AF139865 Synthetic construct plasmid pJHCMW1 6'-N-acetyltransferase AAC(6')-IbCP301 (aac(6')-Ib) gene, partial cds.

------------------------------------------------------------------------------------------------------------------------------------------------------------------------------------------------------------------------------------------------------------------------TGGGAAGAAGAAACCGATCCAGGAGTACGCGGAATAGACCAGTTACTGGCGAATGCATCACAACTGGGCAAAGGCTTGGGAACCAAGCTGGTTCGAGCTCTGGTTGAGTTGCTGTTCAATGATCCCGAGGTCACCAAGATCCAAACGGACCCGTCGCCGAGCAACTTGCGAGCGATCCGATGCTACGAGAAAGCGGGGTGGGAGAGGCAAGGTACCGTAACCACCCCAGATGGTCCAGCCGTG

>AF139871 Synthetic construct plasmid pJHCMW1 6'-N-acetyltransferase AAC(6')-IbSSF8 (aac(6')-Ib) gene, partial cds.

---------------------------------------------------------------------------------------------------------------------------------------------------------------------------------------------------------------------------------------------------------------------------------------------------------------------------------------------------------------------CGAGCTCTGGTTGAGTTGCTGTTCAATGATCCCGAGGTCACCAAGATCCAAACGGACCCGTCGCCGAGCAACTTGCGAGCGATCCGATGCTACGAGAAAGCGGGGAAAGAGAGGCAAGGTACCGTAACCACCCCAGATGGTCCAGCCGTGTACATGGTTCAAACACGCCAGGCATTCGAGCGAACACGCAGTGTTGCCTAA

>AF139870 Synthetic construct plasmid pJHCMW1 6'-N-acetyltransferase AAC(6')-IbSSF2 (aac(6')-Ib) gene, partial cds.

---------------------------------------------------------------------------------------------------------------------------------------------------------------------------------------------------------------------------------------------------------------------------------------------------------------------------------------------------------------------------------GAGTTGCTGTTCAATGATCCCGAGGTCACCAAGATCCAAACGGACCCGTCGCCGAGCAACTTGCGAGCGATCCGATGCTACGAGAAAGCGGGGAACGAGAGGCAAGGTACCGTAACCACCCCAGATGGTCCAGCCGTGTACATGGTTCAAACACGCCAGGCATTCGAGCGAACACGCAGTGTTGCCTAA

>AF139869 Synthetic construct plasmid pJHCMW1 6'-N-acetyltransferase AAC(6')-IbCP262 (aac(6')-Ib) gene, partial cds.

---------------------------------------------------------------------------------------------------------------------------------------------------------------------------------------------------------------------------------------------------------------------------------------------------------------------------------------------------------------------------------GAGTTGCTGTTCAATGATCCCGAGGTCACCAAGATCCAAACGGACCCGTCGCCGAGCAACTTGCGAGCGATCCGATGCTACGAGAAAGCGGGGTCCGAGAGGCAAGGTACCGTAACCACCCCAGATGGTCCAGCCGTGTACATGGTTCAAACACGCCAGGCATTCGAGCGAACACGCAGTGTTGCCTAA

>AF139868 Synthetic construct plasmid pJHCMW1 6'-N-acetyltransferase AAC(6')-IbCP210 (aac(6')-Ib) gene, partial cds.

---------------------------------------------------------------------------------------------------------------------------------------------------------------------------------------------------------------------------------------------------------------------------------------------------------------------------------------------------------------------------------------CTGTTCAATGATCCCGAGGTCACCAAGATCCAAACGGACCCGTCGCCGAGCAACTTGCGAGCGATCCGATGCTACGAGAAAGCGGGGGGGGAGAGGCAAGGTACCGTAACCACCCCAGATGGTCCAGCCGTGTACATGGTTCAAACACGCCAGGCATTCGAGCGAACACGCAGTGTTGCCTAA

>AF139867 Synthetic construct plasmid pJHCMW1 6'-N-acetyltransferase AAC(6')-IbCP224 (aac(6')-Ib) gene, partial cds.

------------------------------------------------------------------------------------------------------------------------------------------------------------------------------------------------------------------------------------------------------------------------------------------------------------------------------------------------------------------------------------------------GATCCCGAGGTCACCAAGATCCAAACGGACCCGTCGCCGAGCAACTTGCGAGCGATCCGATGCTACGAGAAAGCGGGGTATGAGAGGCAAGGTACCGTAACCACCCCAGATGGTCCAGCCGTGTACATGGTTCAAACACGCCAGGCATTCGAGCGAACACGCAGTGTTGCCTAA

>AF139866 Synthetic construct plasmid pJHCMW1 6'-N-acetyltransferase AAC(6')-IbCP220 (aac(6')-Ib) gene, partial cds.

------------------------------------------------------------------------------------------------------------------------------------------------------------------------------------------------------------------------------------------------------------------------------------------------------------------------------------------------------------------------------------------------GATCCCGAGGTCACCAAGATCCAAACGGACCCGTCGCCGAGCAACTTGCGAGCGATCCGATGCTACGAGAAAGCGGGGATCGAGAGGCAAGGTACCGTAACCACCCCAGATGGTCCAGCCGTGTACATGGTTCAAACACGCCAGGCATTCGAGCGAACACGCAGTGTTGCCTAA

>AF139864 Synthetic construct plasmid pJHCMW1 6'-N-acetyltransferase AAC(6')-IbCP213 (aac(6')-Ib) gene, partial cds.

---------------------------------------------------------------------------------------------------------------------------------------------------------------------------------------------------------------------------------------------------------------------------------------------------------------------------------------------------------------------------------------------------CCCGAGGTCACCAAGATCCAAACGGACCCGTCGCCGAGCAACTTGCGAGCGATCCGATGCTACGAGAAAGCGGGGATGGAGAGGCAAGGTACCGTAACCACCCCAGATGGTCCAGCCGTGTACATGGTTCAAACACGCCAGGCATTCGAGCGAACACGCAGTGTTGCCTAA

>X68227 E.coli plasmid pSa aadA2 gene for streptomycin resistance.

------------------------------------------------------------------------------------------------------------------------------------------------------------------------------------------------------------------------------------------------------------------------------------------------------------------------------------------------------------------------------------------------------------------------------------------------------------------------------------------GGTACCGTAACCACCCCAGATGGTCCAGCCGTGTACATGGTTCAAACACGCCAGGCATTCGAGCGAACACGCAGTGATGCCTAA

>AJ550807 Pseudomonas aeruginosa partial Tn21-like transposon and partial Tn402-like transposon.

GTGACCAACAGC---AACGATTCCGTCACACTGCGCCTCATGACTGAGCATGACCTTGCGATGCTCTATGAGTGGCTAAATCGATC
