# Supplementary material for: Synonymous Genes Explore Different Evolutionary Landscapes
Source: PLoS Genet. 2008 Nov 14;4(11):e1000256. doi: 10.1371/journal.pgen.1000256 (PMC2575237; doi:10.1371/journal.pgen.1000256)
Supplement: Text S1 — Modeling of the relationship between protein space exploration and library size. (0.10 MB DOC) [file pgen.1000256.s007.doc]

# Text S1: Modeling of the relationship between protein space exploration and library size

Let us assume that the sequence is *L* nucleotides long and that any modification in a fraction *f* of its positions is not lethal (*i.e.* leads to properly folded proteins [1]).

The probability that a sequence codes for a properly folded proteins after *m* independent mutations is:

*(1)*

The denominator is the total number of mutants bearing *m* mutations, while the numerator is the number of combinations in which these mutations does not adversely affect protein function. Assuming the sequence length *L* is much larger than the number of introduced mutations *m* (*L >> m*), this equation simplifies into:

*(2)*

which is consistent with several studies [1, 2].

Let us now consider a given target optimal genotype that is *k* mutations away from the reference one. Among sequences with *m* mutations, the probability that the *k* desired mutations are present is:

*(3)*

The probability that a sequence with *m* mutations encodes a properly folded protein and contains the *k* desired mutations directly stems from equation (2) and (3):

*(4)*

If we assume, as usual, that a library is composed of sequences with a Poisson distributed number of mutations with mean X, then the probability to find the target sequence coding for a properly folded protein is:

*(5)*

which simplifies into:

*(6)*

The inverse of *(6)* is the mean library size required to generate one target clone.

Deriving equation *(6)* with respect to *X* gives the optimal mean mutation rate respective to targets *k* mutations away

*(7)*

The graph below displays the inverses of equation *(6)* for target variants at *k=1* (red), *k=2* (orange) and *k=3* (yellow) mutations away from the template. We assumed a standard bacterial gene length (*L=1000*) and a conservative proportion of non-deleterious mutations at the DNA level (*f=3/4*, corresponding to 1/3 of lethal aa substitutions [3]). Numbers on the left side scale are obtained by calculating the inverse of equation *(6)* for X equal to X_opt_ from equation *(7).*


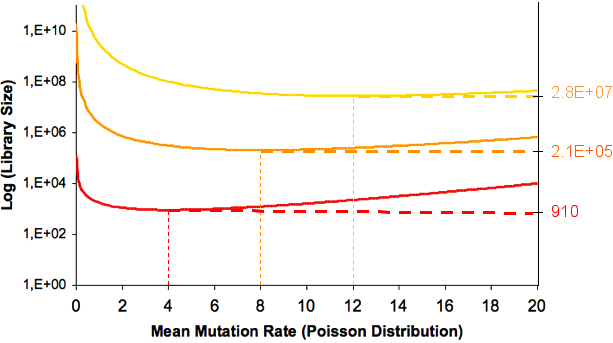


The increase in required size between a library covering a mutational distance *k+i* and one targeting *k* mutations is:

*(8)*

The larger the mean number of mutations, the higher the chance to recover a target further away. However, optimal mutation rate for error-prone PCR derived libraries are predicted to be rather low, even when subtle advantages of high mutation rate are taken into account [2].

The following graph displays equation *(8)* for *i=1* (red) and  *i=2* (orange). A substantial increase in library size is required to fully explore possibilities, even with a somewhat high mutation rate of 4 mutations on average per gene (dotted line). As the occurrence of several mutations in the same codon is very rare using error-prone PCR, these curves can be interpreted as lower bounds to the increase in library size necessary to obtain a 2 or 3 mutations in the same codon instead of 1.


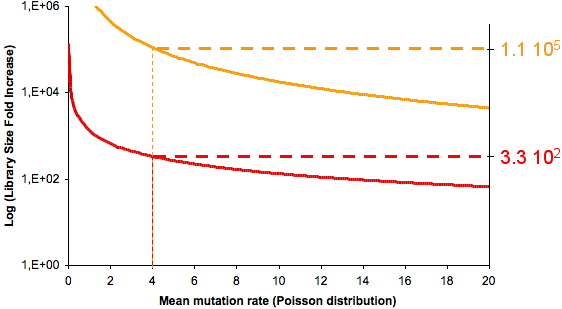


The overall picture could have been worse if we had assumed a cumulative effect of mutations: due to negative epistasis neutral mutations may become deleterious when they accumulate [4].

1. Bloom JD, Silberg JJ, Wilke CO, Drummond DA, Adami C, Arnold FH (2005) Thermodynamic prediction of protein neutrality. PNAS :606–611.

2. Drummond DA, Iverson BL, Georgiou G, Arnold FH (2005) Why High-error-rate Random Mutagenesis Libraries are Enriched in Functional and Improved Proteins. J. Mol. Biology 350: 806-816.

3. Guo HH, Choe J, Loeb LA (2004) Protein tolerance to random amino acid change. PNAS 101: 9205-9210.

4. Bershtein S, Segal M, Bekerman R, Tokuriki N, Tawfik DS (2006) Robustness-epistasis link shapes the fitness landscape of a randomly drifting protein. Nature 444: 929.
